# Residue-Specific Incorporation of Noncanonical Amino Acids in Auxotrophic Hosts: *Quo Vadis?*

**DOI:** 10.1021/acs.chemrev.4c00280

**Published:** 2025-05-16

**Authors:** Žana Marin, Claudia Lacombe, Simindokht Rostami, Arshia Arasteh Kani, Andrea Borgonovo, Monika Cserjan-Puschmann, Jürgen Mairhofer, Gerald Striedner, Birgit Wiltschi

**Affiliations:** 1 Department of Biotechnology and Food Sciences, Institute of Bioprocess Science and Engineering, 27270 BOKU University, Muthgasse 18, 1190 Vienna, Austria; 2 298883 acib - Austrian Centre of Industrial Biotechnology, Muthgasse 18, 1190 Vienna, Austria; 3 enGenes Biotech GmbH, Muthgasse 11/2, 1190 Vienna, Austria

## Abstract

The residue-specific incorporation of noncanonical amino acids in auxotrophic hosts allows the global exchange of a canonical amino acid with its noncanonical analog. Noncanonical amino acids are not encoded by the standard genetic code, but they carry unique side chain chemistries, e.g., to perform bioorthogonal conjugation reactions or to manipulate the physicochemical properties of a protein such as folding and stability. The method was introduced nearly 70 years ago and is still in widespread use because of its simplicity and robustness. In our study, we review the trends in the field during the last two decades. We give an overview of the application of the method for artificial post-translational protein modifications and the selective functionalization and directed immobilization of proteins. We highlight the trends in the use of noncanonical amino acids for the analysis of nascent proteomes and the engineering of enzymes and biomaterials, and the progress in the biosynthesis of amino acid analogs. We also discuss the challenges for the scale-up of the technique.

## Graphical abstract

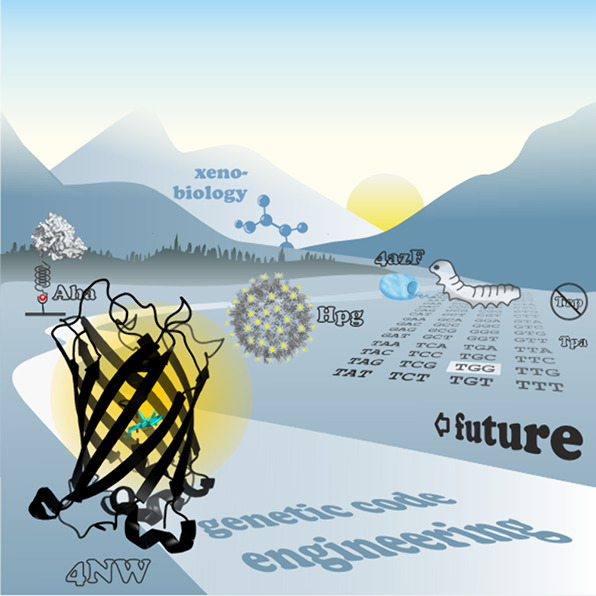

## 1 Introduction

The standard genetic code (SGC) admits only 20 l-α-amino acids to ribosomal translation. Structurally, they consist of an amino group that is connected to a carboxy group *via* the C^α^ atom, which carries one of the 20 different side chains (Figure 1). The 20 amino acids prescribed by the SGC are often called *proteinogenic* or *natural*. However, since many more amino acids can be incorporated into proteins by ribosomal translation with an expanded genetic code (see below) and because many of these amino acids also occur in nature,^1^ the term *canonical* most precisely denotes the protein building blocks prescribed by the SGC.

**Figure 1:**
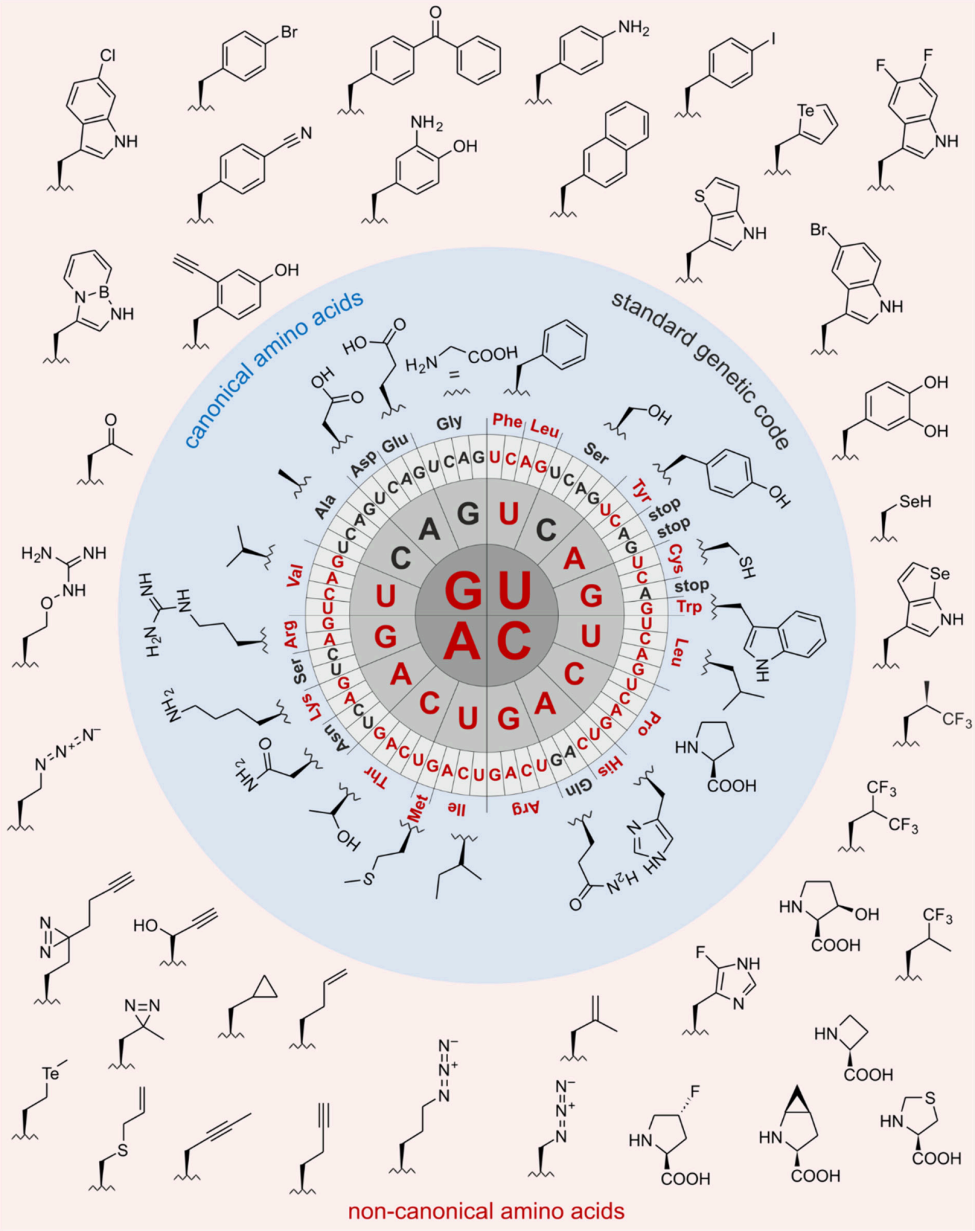
Side chain chemistries of canonical and noncanonical amino acids. The canonical amino acids whose analog incorporation is reviewed in this study are highlighted in red together with their coding units in the standard genetic code. The amino acids are shown in the three-letter code. An illustrative selection of noncanonical amino acids for residue-specific incorporation is shown in the pale-red box.

In addition to the canonical amino acids (cAAs), nature uses the so-called 21st and 22nd amino acids l-selenocysteine (Sec, U) and l-pyrrolysine (Pyl, O) for protein biosynthesis. However, they rather represent exemptions to the rule because they occur in a limited set of proteins (Sec)^2,3^ or in a limited set of organisms (Pyl).^4,5^

The side chain chemistries of the cAAs are modest from an organic chemist’s point of view (Figure 1). To add alternative chemical moieties, the SGC must be engineered or expanded with noncanonical amino acids (ncAAs). As the designation indicates, these amino acids are not encoded by the SGC, nevertheless, they can be introduced into translation and accommodated in proteins under tightly controlled conditions (see Section 2). The side chain chemistries of the ncAAs are vast (a selection is shown in Figure 1) and they include reactive groups such as alkene, alkyne, azide, cyano, nitro, borono, keto, and aldehyde moieties or halogens that are absent from the cAAs.

All 64 coding units of the SGC have a specific meaning. Three codons designate translation stop signals and the remaining 61 codons are assigned to the 20 cAAs (Figure 1). Consequently, a cAA can be encoded by more than one codon, that means the SGC is degenerated. To admit a new amino acid to ribosomal translation for genetic incorporation into a protein, the meaning of at least one codon must be changed or new codons must be generated. The routine methods for ncAA incorporation (detailed below) transiently change the meaning of sense codons or stop codons, predominantly the TAG amber stop. Four-base (quadruplet) codons in combination with a quadruplet-decoding ribosome were also used as coding units for ncAAs.^6^ The degeneracy of the SGC in combination with *de novo* synthesis of whole genomes allows the replacement of selected sense or stop codons by synonymous codons, which “frees” the replaced codons for their assignment to ncAAs. This approach was used successfully to generate organisms with a compressed and expanded genetic code.^7^ In addition, several types of unnatural base pairs have been devised and were shown to be functional *in vitro* ^8^ and *in vivo*,^9^ but they are not yet broadly applied *in vivo* (recently reviewed by Gerecht, et al.^10^).

Two main strategies have been used for the genetic incorporation of ncAAs. To engineer the SGC, a specific cAA is exchanged for a suitable ncAA at all its occurrences in a protein. This approach is called *residue-specific incorporation*. Alternatively, an ncAA can be incorporated *at one or several specific positions* in a protein *in addition to the other cAAs*, which leads to an expansion of the SGC. The approaches are complementary, and they allow us to tailor proteins with genetically encoded noncanonical functions.

The expansion of the SGC has traditionally focused on the UAG amber stop codon (TAG on DNA) to encode an ncAA, but opal (UGA; TGA on DNA)^11^ and ochre (UAA; TAA on DNA)^12^ stop codons as well as quadruplet codons^6^ have also been recoded. To decode an in-frame stop codon (aka “stop codon suppression”, SCS; Figure 2), an appropriate suppressor tRNA is selectively charged with the ncAA. Usually, a mutant aminoacyl-tRNA synthetase (AARS) that accepts the ncAA as a substrate is employed for the aminoacylation of the ncAA. The suppressor tRNA and the mutant AARS must be orthogonal in the host organism (“orthogonal pair”), i.e., they must not interact with the AARS or the tRNAs of the host. Technically, the host must be equipped with an expression construct for the orthogonal pair and the target gene, which is mutated such that an in-frame stop codon encodes the ncAA at the desired position. The site-specific incorporation of ncAAs using orthogonal pairs is beyond the scope of this work, and interested readers are referred to complementary reviews summarizing orthogonal translation systems for the site-specific incorporation of ncAAs^13–15^ and the applications of the technique.^16–18^

**Figure 2:**
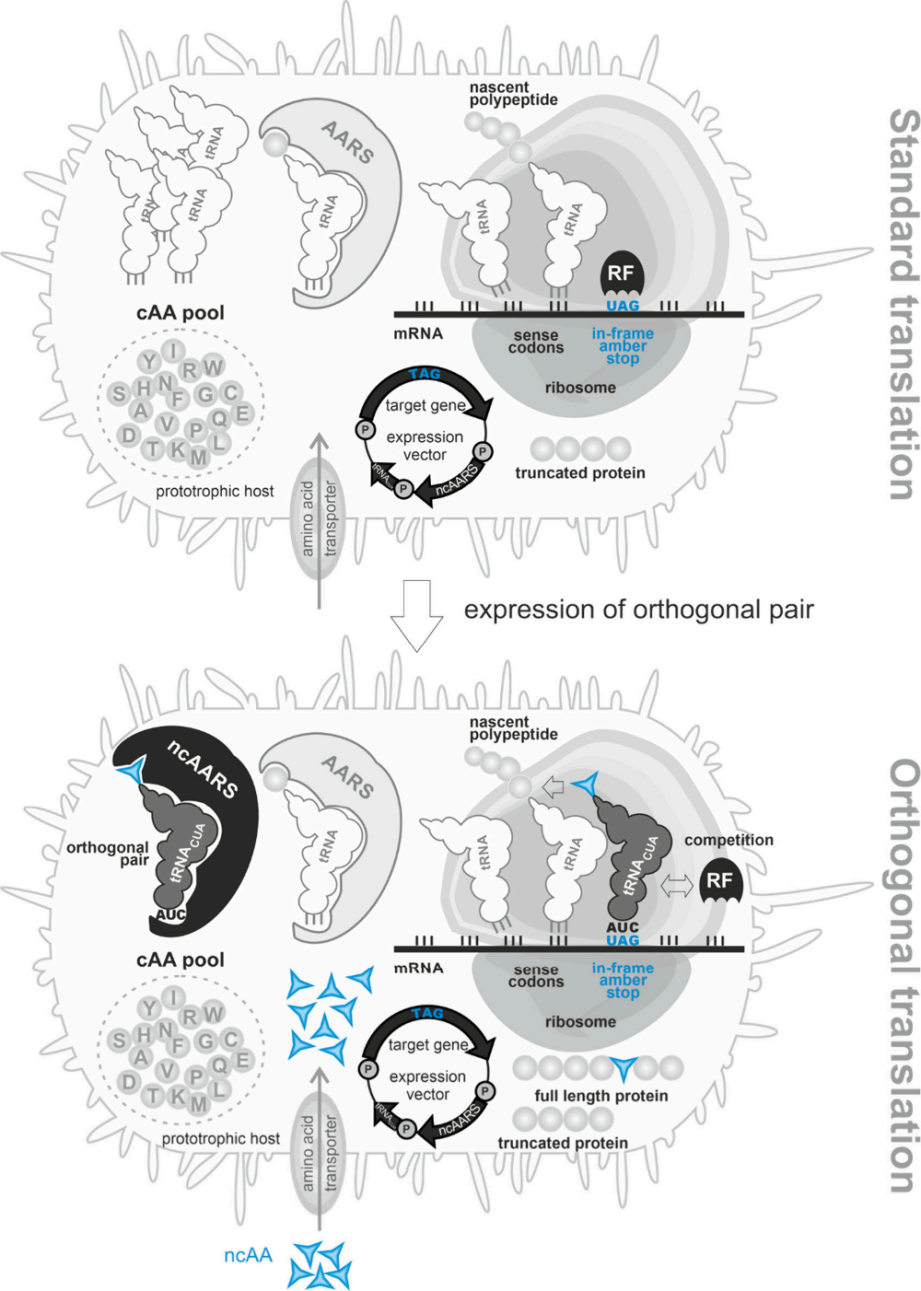
Expansion of the genetic code: Site-specific incorporation of noncanonical amino acids by amber stop codon suppression. A recombinant target gene is designed such that an in-frame amber stop codon defines the position of the ncAA incorporation. Under standard expression conditions (top), the in-frame amber stop codon is decoded by a release factor (RF) that triggers the termination of ribosomal translation. To allow the incorporation of an ncAA (bottom), the recombinant host expresses a mutant aminoacyl-tRNA synthetase that recognizes the ncAA (ncAARS) and its cognate suppressor tRNA_CUA_. The ncAA is supplied in the medium and taken up into the cell. The ncAARS charges the ncAA onto the tRNA_CUA_. The ncAARS/tRNA_CUA_ pair is orthogonal as it does not interact with the host AARSs and tRNAs. The charged ncAA-tRNA_CUA_ is delivered to the ribosome where the ncAA is inserted into the nascent polypeptide in response to the in-frame amber stop codon. The ncAA-tRNA_CUA_ competes with the RF to decode the amber stop. The more efficiently the ncAA-charged tRNA_CUA_ outcompetes the RF the higher the level of produced full-length alloprotein. When the ncAA incorporation fails, a truncated version of the target protein is formed. Since the ncAA is transiently added to the standard genetic code in addition to the 20 cAAs, a prototrophic host can be used.

Here, we focus on the residue-specific incorporation of ncAAs. Many ncAAs are structural and/or chemical analogs of the cAAs. It has been known for more than 60 years, that analogs e.g., of Met, Trp, Phe and Arg can be incorporated throughout the proteome of bacterial hosts.^19^ These cAA analogs can act as surrogates for the cognate substrates of the AARSs despite the high accuracy of aminoacylation.^20^ However, usually they are much worse substrates than the cAAs.^21–25^ Consequently, to be charged (efficiently) onto the tRNA(s) the ncAA must be present at much higher concentration in the cell than the corresponding cAA. A straightforward strategy to minimize the intracellular concentration of free cAA consists in the use of a host strain that is auxotrophic for it. Since the auxotroph lacks the ability to biosynthesize this cAA, it will be unable to grow in minimal medium unless it is supplemented with appropriate amounts of it. Hence, the intracellular cAA level can be controlled by the cAA concentration in the medium. Auxotrophic Escherichia coli strains have been used extensively for the residue-specific incorporation of ncAAs. Amino acid auxotrophs of yeast and Lactococcus lactis are suitable for this approach as well. Among others, Met and Trp are essential amino acids of mammalian cells,^26^ which have been exploited, e.g., for the proteome-wide incorporation of reactive Met analogs.

Once the cells are deprived of the cAA, they can be supplemented with the ncAA. Synthetic ncAAs are usually added to the medium, it is important that they are soluble, efficiently enter the cells and stably accumulate inside them (Figure 3). Some ncAAs can be biosynthesized for residue-specific incorporation (Section 5.2). If the compound resembles its canonical counterpart, the corresponding AARS will accept it as a substrate and charge it onto its cognate tRNA(s). Although AARSs govern the fidelity of ribosomal translation, they have a remarkable substrate tolerance as reviewed very recently by Hartmann.^27^ In most cases, the AARSs and tRNAs of the host are hijacked for the incorporation of the ncAA. Occasionally, wildtype or mutant AARSs, e.g., with an expanded substrate scope or inactivated editing function have been overexpressed to improve the incorporation of an ncAA.

**Figure 3:**
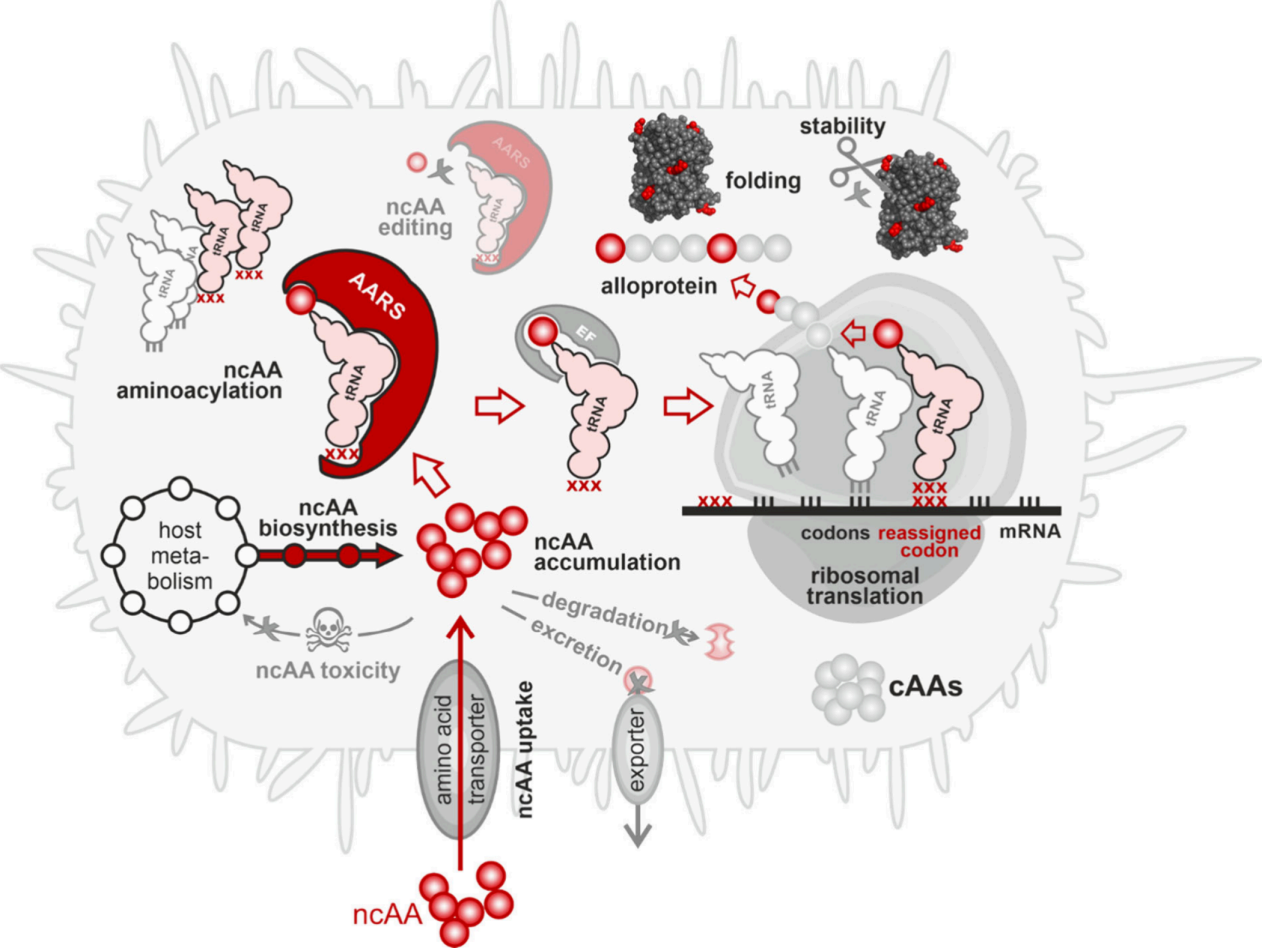
Journey of a noncanonical amino acid into a polypeptide. The cell takes up the ncAA from its environment or biosynthesizes it. The ncAA should not interfere with the host metabolism nor act as a toxin. To accumulate, it must not be degraded nor excreted by the cell. If its intracellular concentration reaches an adequate level, an aminoacyl-tRNA synthetase (AARS) activates it and charges it onto a tRNA. Discrimination against the ncAA by the editing function of some AARS prevents the downstream steps and should be avoided. An elongation factor delivers the ncAA-charged tRNA to the ribosome, where it participates in ribosomal translation. Peptide bonding stably incorporates the ncAA into the nascent polypeptide. The resulting alloprotein must be able to fold correctly and should not be degraded, e.g., by proteolytic enzymes.

The ncAA is incorporated *instead of* a certain cAA. In other words, the genetic information of a given target gene is “interpreted” with an alternative genetic code. While single as well as multiple positions can be exchanged efficiently, for a directed insertion, those positions where the ncAA should not be incorporated must be mutagenized. Whether this is a suitable approach depends on the stability and the function of the resulting mutant.

In 2004, Budisa published an extensive overview on genetic code engineering,^28^ and in 2006 a book-length review followed.^29^ Since then, many reviews have shed light on various aspects of the engineering of proteins with ncAAs with an increasing emphasis on the site-specific techniques. Our review covers the last two decades from 2004 until today. We have explored the trends in genetic code engineering during this period, specifically the residue-specific incorporation of ncAAs using auxotrophic hosts. Section 2 discusses the steps it takes for an ncAA to enter a polypeptide and briefly introduces the experimental details of genetic code engineering. In Section 3, we give an overview of the classic achievements in this field with respect to functionalization of proteins with reactive handles, the tuning of fluorescence properties, protein folding and stability as well as function. Examples for the application of ncAAs for analytics conclude Section 3. Section 4 describes developments beyond the classics, such as strategies to facilitate and improve the incorporation of ncAAs into demanding proteins, approaches to introduce two ncAAs into the same polypeptide as well as to addict the host to an ncAA. We review how the properties of protein-based biomaterials can be tuned with ncAAs, and we give an update on the exploration of proteomes using ncAAs to probe newly synthesized proteins. Section 5 focuses on the future challenges of the field. These include the uptake of ncAAs into cells and the analysis of their cellular fate, their biosynthesis as well as pitfalls of their accidental incorporation. We scrutinize the challenges of scalable bioprocesses for the production of alloproteins. In the final section of Section 5, we look at futuristic trends in the field. Given the tremendous development of the field in the past decade, the literature cited in this manuscript is far from exhaustive. We apologize to all authors whose works we were unable to include.

The following topics are out of the scope of this work: the site-specific incorporation of ncAAs using orthogonal translation systems, which includes the use of artificially auxotrophic cells that are ncAA dependent; the incorporation of ncAAs into hosts whose amino acid biosynthesis pathways are blocked by inhibitors; and sense codon reassignment using cell-free expression systems.

In this work, we use a couple of terms that warrant definition for the sake of clarity: NcAAs are incorporated into *target proteins*. The terms *variant, congener*, and *alloprotein* designate a protein that contains one or several ncAAs. The corresponding protein without ncAAs is the *parent protein* or *parent* for short. In contrast, a mutant is a protein whose genetic information was changed (as opposed to the wildtype). A parent protein can be a wildtype or a mutant. The *incorporation/labeling efficiency* indicates how much alloprotein is produced in comparison to the parent, which is set to 100%. *Quantitative replacement/incorporation* indicates that only fully labeled protein was detectable, e.g., by mass spectrometry or amino acid analysis, while parent protein or incompletely labeled proteins were not detected. For comprehensibility, we use the full names of amino acids or their three-letter codes in the text, but mutants are indicated by the one-letter code for brevity. For instance, *Ec*PheRS^A294G^ designates the alanine to glycine mutant at position 294 (A294G) of the phenylalanyl-tRNA synthetase (PheRS) of E. coli (*Ec*). Auxotrophic strains that were used in the cited literature are listed in Table 1, and the variant proteins mentioned in the text are listed in Table 2; ncAA structures are shown in Figures 7 and 9–14.

**Table 1:** Amino Acid Auxotrophic Hosts for the Residue-Specific Incorporation of Different ncAAs[Table-fn t1fn0] ^,^ ^a^

organism	name	auxotrophy	relevant genotype	strain ref(s)	used-by ref(s)
E. coli	4TUB93	Trp	Δ*trpLEDC* Δ*tnaA* ALE for 4FW	^311^	^311^
E. coli	5TUB83	Trp	Δ*trpLEDC* Δ*tnaA* ALE for 5FW	^311^	^311^
E. coli	AF-IQ	Phe	Δ*pheA*	^261^,^554^	^63^,^67^,^86^,^91^,^105^,^113^,^265^
E. coli	AIV-IQ	Ile, Val	*ilvD691*	^108^	^97^,^109^
E. coli	AMF-IQ	Met, Phe	Δ*metE* Δ*pheA*	^182^	^182^
E. coli	AT2471	Tyr	*tyrA4*	^555^	^115^,^125^,^148^
E. coli	B/r WP2 (ATCC 49980, CGSC #5378)	Trp	*trpE65*(Oc)	^556^,^557^	^80^,^93^,^115^,^119^,^120^,^127^,^138^
E. coli	B834(DE3)	Met	*metE*	^558^,^559^	^58^,^59^,^64^,^83^,^93^,^104^,^107^,^117^,^118^,^130–133^ ,^135^,^136^,^143,145^,^180^,^453^
E. coli	BL21(DE3) (Δ*argH*Δ*trpC*Δ*hisB*)	Arg, Trp, His	Δ*argH* Δ*trpC* Δ*hisB*	^77^	^77^
E. coli	BL21(DE3)Δ*tyrApheA*	Phe, Tyr	Δ*tyrA* Δ*pheA*	^51^	^51^
E. coli	B834(DE3)pLysS	Met	*metE*	^558^,^560^	^47–49^
E. coli	BL21(DE3)pLysS (KC1325)	Pro	*proB*1658::Tn*10*	^561^	^122^,^217^,^219^,^304^
E. coli	BL21(DE3)*cysE51*	Cys	*selB*::kan *cysE51*	^238^	^235–237^
E. coli	BW25113 *tyrA*::kan^R^	Tyr	*tyrA*::kan^R^	^56^	^56^
E. coli	BWEC44	Pro	Δ*proC*	^129^	^129^
E. coli	BWEC47	Trp	Δ*trpC*	^142^	^142^,^144^
E. coli	C600 ΔtrpE	Trp	Δ*trpE*	^309^	^306^,^309^
E. coli	CAG18455	Trp	*trpC83*::Tn*10*	^562^	^250^
E. coli	CAG18491	Met	*metE3079*::Tn*10*	^563^	^114,115,123,125,209^
E. coli	CAG18515	Pro	*proAB3096*::Tn*10kan*	^563^	^34^,^66^,^74^,^82^,^92^,^115^,^123^,^125^,^221^
E. coli	CY1507729 (aka MD33)	Trp	Δ(*trpEA*)*2*	^564^,^565^	^89^
E. coli	DG30	Phe	*tyrB507*	^566^	^115^,^125^
E. coli	DG99	Pro	*proC*::Tn*10*	^34^	^34^
E. coli	DH10B	Leu	Δ*(araA-leu)7697*	^567^	^300^
E. coli	DH10B metE	Met	*metE*	^112^	^112^,^270^
E. coli	DL39(DE3)	Phe, Tyr	*tyrB507*	^482^	^94^
E. coli	DL41	Met	*metA28*	^241^	^75^,^347^
E. coli	DSM 1563	Leu	*leu* ^*–*^	DSM 1563	^209^
E. coli	ER2566/Trp82	Trp	*trpB*	^568^	^79^
E. coli	K10-F6Δ	Phe	*pheS13 pheA18*::Tn*10*	^259^,^271^	^278^
E. coli	JE5630	Met Pro Trp	*metA* or *B proC trp*	NBRP JE5630	^128^
E. coli	JE7094	Arg	*argG*	NBRP JE7094	^146^
E. coli	JE7345	Pro Met Trp	*metA* or *B proC trp*	NBRP JE7345	^124^,^128^
E. coli	JM109	Pro	Δ(*lac*-*proAB*)	^569^	^218^
E. coli	JM83	Pro	Δ(*lac*-*proAB*)	^569^	^76^,^93^,^121^,^126^,^139^
E. coli	JW2581	Tyr	Δ*tyrA763*::kan	^570^	^56^,^60–62^ ,^65^,^68–70^ ,^110^,^111^,^147^
E. coli	KS32	Pro	Δ*proA putA1*::Tn*5*	^74^	^74^
E. coli	LAM1000	Leu	Δ*(araA-leu)7697*	^571^,^572^	^87^,^88^,^95^,^100^,^101^,^103^,^331^,^366^
E. coli	M15MA metG*	Met	*metE*	^573^	^573^
E. coli	M15MA/pREP4^b^	Met	*metE*	^269^	^58^,^85^,^98^,^99^,^102^,^189^,^269^,^573^
E. coli	MC1061 metG*	Met	*metE*	^574^	^574^
E. coli	MDS15 (B834(DE3) descendant)	Met	Δ*metE* Δ*metA780*	^140^	^140^
E. coli	ME5355 (strain AN92)	Phe Trp Tyr	*pheA1 tyrA4 trp-401 aroB351*	^575^, NBRP ME5355	^134^
E. coli	MG1655 Δ*proBA*::frt Δ*proC*::frt (DE3)	Pro	Δ*proBA*::frt Δ*proC*::frt	n.i.	^90^
E. coli	MPC390	Leu Phe	*leuB6*(Am) *pheA18*::Tn*10*	CGSC #7494	^273^
E. coli	MT20	Trp	*ΔtrpLEDCBA* [pSTB7 *trpBA*]	^202^	^202^
E. coli	MT21(DE3)	Trp	*ΔtrpLEDCBA* [pSTB7 *trpBA*]	^81^	^81^
E. coli	NK6024	Phe	*pheA18*::Tn*10*	^576^	^250^
E. coli	RF11 (C43(DE3) descendant)	Met	*metA*	^577^	^50^
E. coli	SB3930	His	Δ*hisB463*	CGSC #4930	^275^
E. coli	TUB00	Trp	Δ*trpLEDC* Δ*tnaA*	^311^	^311^,^316^,^318^
E. coli	TUB170	Trp	Δ*trpLEDC* Δ*tnaA* ALE for [3,2]Tpa	^316^	^316^
E. coli	UMM5	Pro	*putA1*::Tn*5 proC*24	^578^	^34^
E. coli	UTH780	His	*hisC780*	^579^	^72^
E. coli	W3110TrpA33(DE3)/pLysS	Trp	*trpA33*	^580^	^198^
B. subtilis	QB928	Trp	*aroI906 trpC*_*2*_	^581^	^305^,^307^,^308^
L. lactis	NZ9000	Met	*met* ^*–*^	^582^	^71^,^256^
L. lactis	PA1002	Trp	Δ*trpBA*	^73^	^73^,^96^
P. pastoris (aka K. phaffii)	X33 Δ*aro1*	Phe, Trp, Tyr	*aro1*::*URA3*	^583^	^155^
S. cerevisiae	Y03254	Met	*met15*Δ0	EUROFAN Y03254	^154^

a Amino acid auxotrophic strains are available from public strain banks such as the Coli Genetic Stock Center at Yale (CGSC) (recently relocated to the E. coli Genetic Resource Center, https://ecgrc.net/), the Keio Collection (https://shigen.nig.ac.jp/ecoli/strain/resource/keioCollection/about) or Addgene (https://www.addgene.org/) for Escherichia coli or the EUROSCARF collection (http://www.euroscarf.de) for Saccharomyces cerevisiae and Pichia pastoris strains from the Pichia Pool collection.^552,553^

b ALE, adaptive laboratory evolution; n.i., not indicated.

c The Lee group^58,189^ used E. coli strain M15A, which might actually be M15MA.

**Table 2:** Residue-Specifically Labeled Target Proteins[Table-fn t2fn1]

target protein	relevant residues	ncAA(s)	variant protein	titer	effect
4-OT	2 Pro	4cFP	4-OT[4cFP]	15–30 mg/L^129^	tuning enzyme activity: replacement of the catalytic N-terminal Pro residue of 4-OT or 4-OT^P34E^ leads to loss of Michael-type activity^129^
	2 Pro	4tFP	4-OT[4tFP]	15–30 mg/L^129^	ditto^129^
	2 Pro	dhP	4-OT[dhP]	15–30 mg/L^129^	ditto^129^
	2 Pro	Thz	4-OT[Thz]	15–30 mg/L^129^	ditto^129^
4RepCT	3 Met	Aha	4RepCT[Aha]	n.a.	spider silk mimic with reactive azide handles, e.g., to attach fluorophores and the antibiotic levofloxacin^75^ or the cell-adhesion RGD motif;^347^ the antibiotic was conjugated *via* an acid-labile linker to sustainably release it over several days^75^
ψ-b*	15 Leu	Tfl	ψ-b*[Tfl]	n.a.	Tfl incorporation inefficient^209^
	1 Met	Aha	ψ-b*[Aha]	5 mg/L^133^	reactive azide handle for artificial glycosylation,^133^ or dendronylation^131^
				7.36 mg/L^131^	
	1 Met	bioAha	ψ-b*[bioAha]	5.6 mg/L^130^	reactive azide handle for fluorescent labeling by CuAAC; SPI of bioAha; biosynthesis of Aha by MetY with supplementation of *O*-acetyl-l-homoserine and NaN_3_ ^130^
	1 Met	Cpa	ψ-b*[Cpa]	n.a.	incorporation of Cpa at N-terminal AUG codon^114^
	1 Met	Hpg	ψ-b*[Hpg]	8 mg/L^178^	reactive alkyne handle for protein iodination^178^
	1 Pro	4cFP	ψ-b*[4cFP]	n.a.	4cFP improved the refolding^218^
	1 Pro	4tFP	ψ-b*[4tFP]	n.a.	no effect^218^
	1 Pro	4dFP	ψ-b*[4dFP]	n.a.	no effect^218^
	3 Trp	4mW	ψ-b*[4mW]	n.a.	ψ-b*[4mW] behaved as the parent^138^
	3 Trp	4NW	ψ-b*[4NW]	n.a.	4NW destabilized the protein;^138^ PDB 2HXX
	3 Trp	5NW	ψ-b*[5NW]	n.a.	5NW destabilized the protein^138^
	1 Met, 1 Pro, 3 Trp	Hpg, 4cFP, 4AW	ψ-b*[Hpg 4cFP 4AW]	5 mg/L^128^	multi-ncAA SPI; improved stability, blue fluorescence, reactive alkyne handle^128^
ψ-b**	2 Met	Aha	ψ-b**[Aha]	5.2 mg/L^131^	reactive azide handle for dendronylation^131^
	2 Met	bioAha	ψ-b**[bioAha]	1.5 mg/L^140^	reactive azide handle for dendronylation; SPI of bioAha; biosynthesis of Aha by by MetX and MetY with NaN_3_ supplementation^140^
ψ-b*4M	4 Met	Cpa	ψ-b*4M[Cpa]	n.a.	Cpa incorporated at internal and N-terminal AUG codons^114^
	4 Met	Hpg	ψ-b*4M[Hpg]	n.a.	reactive azide handle for artificial glycosylation, used in multivalent binding studies with lectin^117^
ω-TA	31 Pro	4cFP	ω-TA[4cFP]	n.a.	insoluble variant^304^
	31 Pro	4tFP	ω-TA[4tFP]	n.a.	increased thermostability and solvent tolerance^304^
ω-TA R23am	31 Pro	Dopa	ω-TA R23Dopa	n.a.	directed immobilization on chitosan and polystyrene beads; catalytic activity survived 10 rounds of reuse^304^
	31 Pro	Dopa, 4tFP	ω-TA[4tFP] R23Dopa	n.a.	SPI combined with SCS; increased thermostability and solvent tolerance; directed immobilization on chitosan and polystyrene beads; catalytic activity survived 10 rounds of reuse^304^
A_CS5_-(E_5_)_3_	15 Phe	4azF	A_CS5_-(E_5_)_3_[4azF]	66 mg/L^91^	artificial extracellular matrix protein: thin biofilms of controlled elastic moduli for adhesion of mammalian cells^91^
A_RGD_-(E_5_)_3_	15 Phe	4azF	A_RGD_-(E_5_)_3_[4azF]	40 mg/L^67^	artificial extracellular matrix protein: photolithographic biomaterial for patterned adhesion of mammalian cells^67^
AlaDH	11 Tyr	bio2FY	AlaDH[bio2FY]	5.2 mg/L^110^	improved stability; SPI of biosynthesized 2FY; biosynthesis of 2FY from 3-fluorophenol, pyruvate and ammonia by *Cf*TPL^110^
anxA5	35 Leu	Tfl	anxA5[Tfl]	0.1 mg/L^209^	Tfl incorporation inefficient^209^
	1 Trp	bio4AW	anxA5[bio4AW]	17 mg/L^127^	intrinsically blue fluorescent protein; λ_ex max_ red-shifted by 105 nm relative anxA5 parent^127^
	1 Trp	bio5AW	anxA5[bio5AW]	12 mg/L^127^	intrinsically blue fluorescent protein; λ_ex max_ red-shifted by 96 nm relative anxA5 parent^127^
	1 Trp	bio6AW	anxA5[bio6AW]	n.a.	incorporation incomplete^127^
	1 Trp	bio7AW	anxA5[bio7AW]	n.a.	lower fluorescence intensity due to easy quenching and less red-shifted λ_ex max_ than the other variants^127^
AprA	1 Met	Dfm	AprA[Dfm]	16 mg/L^107^	incorporation of ^19^F biophysical probe into the active center causes minimal catalytic or structural alteration^107^
blockCE_5_	6 Phe	4FF	blockCE_5_[4FF]	3.87 mg/L^113^	tuning biomaterial properties: supramolecular assembly at lower temperature than parent; robust elastic network formation at elevated temperatures^113^
blockCE_2_	11 Leu	Tfl	blockCE_2_[Tfl]	30 mg/L^331^	tunable biomaterial with theranostic properties: self-assembly into thermoresponsive micelles for drug delivery and ^19^F MRI/MRS^331^
blockE_5_C	6 Phe	4FF	blockE_5_C[4FF]	4.44 mg/L^113^	tuning biomaterial properties: robust elastic network formation at elevated temperatures^113^
blockE_5_CE_5_	11 Phe	4FF	blockE_5_CE_5_[4FF]	5.68 mg/L^113^	tuning biomaterial properties: conformation deviates from parent at elevated temperatures and becomes beta-rich; enhanced cooperativity during supramolecular assembly in comparison to parent; robust elastic network formation at elevated temperatures^113^
*Bm*Silk	4 Met	Aha	*Bm*Silk[Aha]	–*	silk with reactive handle, e.g., for CuAAC with biotin-PEG_4_-alkyne; Aha is toxic for the silkworm larvae^336^
	4 Met	Hag	*Bm*Silk[Hag]	–*	silk with reactive handle^336^
	4 Met	Hpg	*Bm*Silk[Hpg]	–*	silk with reactive handle, e.g., for CuAAC with biotin-PEG_3_-azide^336^
	35 Phe	4azF	*Bm*Silk[4azF]	–*	silk with reactive handle, e.g., threads, films and porous sponges of *Bm*Silk[4azF] were bioorthogonally conjugated to fluorophores, biotin and DBCO-modified GFP^354^ or cross-linked with a bifunctional (DBCO)_2_-PEG_4_ linker;^359^ the photolabile arylazide of 4azF can be exploited for photopatterning;^354^ low-level 4azF incorporation by the mutant *Bm*PheRS^A450G349^ was improved with the mutants *Bm*PheRS^αT407A354^ and *Bm*PheRS^αF432V356^; 1000 larvae expressing *Bm*PheRS^αF432V^ produced 160 g of azido-labeled silk^357^
	35 Phe	4BrF	*Bm*Silk[4BrF]	–*	brominated silk^349^
	35 Phe	4ClF	*Bm*Silk[4ClF]	–*	chlorinated silk^349^
	35 Phe	4yF	*Bm*Silk[4yF]	–*	silk with photostable reactive handle, e.g., for CuAAC with biotin-PEG_3_-azide and azide-fluorophore^362^
	271 Tyr	3azY	*Bm*Silk[3azY]	–*	silk with photostable reactive handle, e.g., for SpAAC with DBCO-fluorophore^363^
BRD4(D1)	7 Tyr	bio2FY	BRD4(D1)[bio2FY]	n.a.	^19^F NMR; SPI of biosynthesized 2FY; biosynthesis of 2FY from 3-fluorophenol, pyruvate and ammonia by *Cf*TPL; low incorporation efficiency^94^
	7 Tyr	bio3FY	BRD4(D1)[bio3FY]	n.a.	^19^F NMR; SPI of biosynthesized 3FY; biosynthesis of 3FY from 2-fluorophenol, pyruvate and ammonia by *Cf*TPL; low incorporation efficiency^94^
*bzip*INL	4 Ile	5tFI	*bzip*INL[5tFI]	30 mg/L^97^	coiled-coil dimer of improved stability^97^
*bzip*VNL	4 Val	(2S,3R)Tfv	*bzip*VNL[(2S,3R)Tfv]	27 mg/L^97^	ditto^97^
C	7 Leu	Tfl	C[Tfl]	n.a.	coiled-coil protein with improved thermostability, self-assembles into fibers that can bind curcumin in a metal-dependent manner^366^
CalB	5 Met	Aha	CalB[Aha]	2 mg/L^47^	surface-exposed reactive azide handle for coupling to dansyl-alkyne and alkyne-PEG5000 ^47^
	11 Phe	4FF	CalB[4FF]	17 mg/L^155^	SPI in P. pastoris; tuning of catalytic activity: shelf life noticeably prolonged^155^
	5 Trp	5FW	CalB[5FW]	34 mg/L^155^	SPI in P. pastoris; tuning of catalytic activity: shelf life unaltered^155^
	9 Tyr	3FY	CalB[3FY]	26 mg/L^155^	SPI in P. pastoris; tuning of catalytic activity: shelf life prolonged^155^
CapA^G4M^	1 Met	Aha	CapA^G4^[Aha]	n.a.	incorporation in ring domain; variant protein not produced^116^
	1 Met	Hpg	CapA^G4^[Hpg]	n.a.	ditto^116^
CapA^A10M^	1 Met	Aha	CapA^A10^[Aha]	0.102 mg/L^116^	incorporation in loop domain; ∼40% of corresponding parent protein^116^
	1 Met	Hpg	CapA^A10^[Hpg]	n.a.	incorporation in loop domain; variant protein not produced^116^
CapA^G17M^	1 Met	Aha	CapA^G17^[Aha]	0.032 mg/L^116^	incorporation in tail domain; ∼10% of corresponding parent protein^116^
	1 Met	Hpg	CapA^G17^[Hpg]	0.023 mg/L^116^	incorporation in tail domain; ∼6% of corresponding parent protein^116^
CAT	13 Leu	Tfl	CAT[Tfl]	3.5 mg/L^95^	tuning enzyme properties: decreased thermo- and chemostability^95^
CATL2-A1	13 Leu	Tfl	CATL2-A1[Tfl]	n.a.	adaptation of the protein structure to ncAA incorporation: directed evolution of CAT to fit Tfl into the structure^87^
*Cf*TPL	23 Tyr	bio2FY	*Cf*TPL[bio2FY]	n.a.	accidental incorporation; SPI of biosynthesized 2FY; biosynthesis of 2FY from 3-fluorophenol, pyruvate and ammonia by *Cf*TPL^94^
	23 Tyr	bio3FY	*Cf*TPL[bio3FY]	n.a.	accidental incorporation; SPI of biosynthesized 3FY; biosynthesis of 3FY from 2-fluorophenol, pyruvate and ammonia by *Cf*TPL^94^
comp	2 Trp	7AW	comp[7AW]	0.05 mg/L^79^	therapeutic peptide; not active^79^
	2 Trp	6FW	comp[6FW]	1.2 mg/L^79^	therapeutic peptide with 3-fold increased complement inhibitory activity relative to parent protein^79^
	2 Trp	5HW	comp[5HW]	0.2 mg/L^79^	therapeutic peptide; 28-fold less active than parent protein^79^
CTB	1 Met	Aha	CTB[Aha]	n.a.	neuronal tracer; conjugated to biotin-fluorescent streptavidin complex for fluorescence microscopy or CdSe/ZnS core/shell nanoparticles for electron microscopy^180^
dodecin	1 Trp	4AW	dodecin[4AW]	n.a.	monitoring and manipulation of the electron-transfer-process during the photocycle of dodecin;^141^ PDB 4B2M
	1 Trp	4FW	dodecin[4NW]	n.a.	ditto,^141^ PDB 4B2K
ECFP	2 Trp	[2,3]Sep	ECFP[[2,3]Sep]	10–30 mg/L^120^	Se-containing fluorophore nonfluorescent^120^
	2 Trp	[3,2]Sep	ECFP[[3,2]Sep]	10–30 mg/L^120^	ditto^120^
	2 Trp	[2,3]Tpa	ECFP[[2,3]Tpa]	10–30 mg/L^120^	sulfur-containing fluorophore nonfluorescent^120^
	2 Trp	[3,2]Tpa	ECFP[[3,2]Tpa]	10–30 mg/L^120^	ditto^120^
	2 Trp	4AW	ECFP[4AW]	n.a.	largely insoluble; λ_ex max_ red-shifted to 483 nm (+7 nm) and 505 nm (+5 nm) relative to the ECFP parent^124^
	2 Trp	7AW	ECFP[7AW]	n.a.	greatly insoluble; λ_ex max_ blue-shifted to 470 nm (−6 nm) and 485 nm (−15 nm) relative to ECFP parent^124^
	2 Trp	4FW	ECFP[4FW]	10–30 mg/L^120^	fluorination blue-shifted and diminished fluorescence;^120^ PDB 1RM9
	2 Trp	5FW	ECFP[5FW]	10–30 mg/L^120^	ditto^120^
	2 Trp	6FW	ECFP[6FW]	10–30 mg/L^120^	ditto^120^
	2 Trp	7FW	ECFP[7FW]	10–30 mg/L^120^	ditto^120^
	2 Trp	4mW	ECFP[4mW]	10–30 mg/L^120^	methylated fluorophore slightly red-shifted, intensity unchanged^120^
ECFP-C	5 Met	bioAha	ECFP-C[bioAha]	2.6 mg/L^140^	SPI of biosynthesized Aha inefficient; biosynthesis of Aha by MetXY with NaN_3_ supplementation^140^
ECFP-N	6 Met	bioAha	ECFP-N[bioAha]	36 mg/L^140^	reactive azide handle for dendronylation; SPI of biosynthesized Aha; biosynthesis of Aha by by MetXY with NaN_3_ supplementation^140^
ECFP-anxA5	3 Trp	4NW	ECFP-anxA5[4NW]	n.a.	gold fluorescent apoptosis detection tool; highly thermostable and resistant to oligomerization and aggregation; low background fluorescence, compatible with other intracellular strains^80^
EGFP	7 Arg	Can	EGFP[Can]	0.1 mg/L^146^	low titer due to cytotoxicity of Can^146^
	15 Leu	Tfl	EGFP[Tfl]	n.a.	Tfl incorporation inefficient^209^
	6 Met	Cpa	EGFP[Cpa]	n.a.	incorporation of Cpa at internal AUG codons, potential incorporation at N-terminal AUG codon and excision by E. coli MetAP^114^
	10 Pro	4cFP	EGFP[4cFP]	10–30 mg/L^216^	enhanced folding;^216^ PDB 2Q6P
	10 Pro	4tFP	EGFP[4tFP]	10–30 mg/L^216^	insoluble^216^
	1 Trp	[2,3]Sep	EGFP[[2,3]Sep]	10–30 mg/L^120^	fluorescence properties similar to parent EGFP;^120^ PDB 1RMP
	1 Trp	[3,2]Sep	EGFP[[3,2]Sep]	10–30 mg/L^120^	ditto;^120^ PDB 1RMM
	1 Trp	[2,3]Tpa	EGFP[[2,3]Tpa]	10–30 mg/L^120^	ditto^120^
	1 Trp	[3,2]Tpa	EGFP[[3,2]Tpa]	10–30 mg/L^120^	fluorescence properties ∼25% improved relative to parent EGFP;^120^ PDB 1RMO
	1 Trp	4FW	EGFP[4FW]	10–30 mg/L^120^	fluorescence properties similar to parent EGFP;^120^ complete replacement of Trp by 4FW in strain adapted by ALE^311^
	1 Trp	5FW	EGFP[5FW]	10–30 mg/L^120^	fluorescence properties similar to parent EGFP;^120^ complete replacement of Trp by 5FW in strain adapted by ALE^311^
	1 Trp	6FW	EGFP[6FW]	10–30 mg/L^120^	fluorescence properties similar to parent EGFP^120^
	1 Trp	7FW	EGFP[7FW]	10–30 mg/L^120^	ditto^120^
	1 Trp	4mW	EGFP[4mW]	10–30 mg/L^120^	ditto^120^
	11 Tyr	2FY	EGFP[2FY]	10–30 mg/L^137^	more prone to aggregation than parent^137^
	11 Tyr	3FY	EGFP[3FY]	10–30 mg/L^148^	marginally red-shifted fluorescence; ∼10% lower intensity than parent EGFP;^148^ PDB 1RRX
EGFP^G2A^	6 Met	Cpa	EGFP^G2A^[Cpa]	n.a.	incorporation of Cpa at internal AUG codons, potential incorporation at N-terminal AUG codon and excision by E. coli MetAP^114^
EGFP-2M	2 Met	Tfm	EGFP-2M[Tfm]	n.a.	Tfm incorporation inefficient^209^
EGFP N150am	10 Pro	4cFP	EGFP N150Bpa[4cFP]	0.6 mg/L^123^	SPI combined with SCS; approximately 20% of EGFP parent, the site-specific incorporation of Bpa at position Asn150 counteracted the stability-enhancing effect of global replacement of Pro by 4cFP
elastin	80 Pro	4cFP	elastin[4cFP]	15–50 mg/L^34^	impeded self-assembly^214^
				45–50 mg/L^214^	
	80 Pro	4tFP	elastin[4tFP]	15–50 mg/L^34^	n.a.^34^ enhanced self-assembly^214^
				45–50 mg/L^214^	
	80 Pro	dHP	elastin[dHP]	15–50 mg/L^34^	n.a.^34^
	80 Pro	4cHP	elastin[4cHP]	15–50 mg/L^34^	n.a.^34^
	80 Pro	4tHP	elastin[4tHP]	15–50 mg/L^34^	n.a.^34^
	80 Pro	4dFP	elastin[4dFP]	15–50 mg/L^34^	n.a.^34^
	80 Pro	Aze	elastin[Aze]	15–50 mg/L^34^	Aze incorporated when ProRS expressed from multicopy plasmid^34^
	80 Pro	Thz	elastin[Thz]	15–50 mg/L^34^	Thz incorporated in a Pro auxotrophic, *proC putA* deficient strain when ProRS expressed from multicopy plasmid^34^
	80 Pro	Pip	elastin[Pip]	15–50 mg/L^34^	Pip incorporated when ProRS^C443G^ mutant expressed from multicopy plasmid^34^
	80 Pro	3cFP	elastin[3cFP]	45–50 mg/L^215^	enhanced self-assembly^215^
	80 Pro	3tFP	elastin[3tFP]	45–50 mg/L^215^	impeded self-assembly^215^
ELP	2 Met	Aha	ELP[Aha]	n.a.	reactive azide handle for fluorescence labeling^49^
	2 Met	Hpg	ELP[Hpg]	n.a.	reactive alkyne handle for PEGylation and directed immobilization of the lipase CalB^49^
EYFP	12 Tyr	2FY	EYFP[2FY]	10–30 mg/L^137^	fluorescence intensity increased and less prone to aggregation in comparison to parent^137^
		3FY	EYFP[3FY]	10–30 mg/L^137^	p*K*_a_ 1.3 units lower than that of parent^137^
Fp151	19 Pro	4tHP	Fp151[4tHP]	0.03 mg/L^126^	hydroxylation to mimic hydroxyproline in natural adhesive mussel foot protein; strong chiral bias for the incorporation of the 4-*trans* enantiomer^126^
	19 Pro	4cHP	Fp151[4cHP]	n.d.	not expressed with 4-*cis* enantiomer^126^
	19 Pro	4tFP	Fp151[4tFP]	2 mg/L^126^	fluorination to tune bioglue properties; strong chiral bias for the incorporation of the 4-*trans* enantiomer; ∼7-fold higher expression level than the parent protein^126^
	19 Pro	4cFP	Fp151[4cFP]	n.d.	not expressed with 4-*cis* enantiomer^126^
G_(IgG)_^L21M^	3 Met, 2 Phe	Aha, 4yF	G_(IgG)_^L21M^[Aha,4yF]	2.5 mg/L^182^	stapled peptide; IgG binding 4-fold improved^182^
Gal-1	1 Met	Aha	Gal-1[Aha]	11 mg/L^143^	reactive azide handle for directed modification with small molecule and cross-linking; formation of superlectin^143^
	1 Met	Hpg	Gal-1[Hpg]	11 mg/L^143^	reactive alkyne handle for directed modification with small molecule and cross-linking; formation of superlectin^143^
	1 Trp	bio7AW	Gal-1[bio7AW]	29 mg/L^144^	tuning biophysical properties of binding proteins: drastically reduced interaction with 3′-sulfated oligosaccharides^144^
	1 Trp	bio5FW	Gal-1[bio5FW]	21 mg/L^144^	tuning biophysical properties of binding proteins: only subtle changes in glycan binding profile relative to parent protein^144^
	1 Trp	bio6FW	Gal-1[bio6FW]	20 mg/L^144^	ditto^144^
	1 Trp	bio7FW	Gal-1[bio7FW]	32 mg/L^144^	tuning biophysical properties of binding proteins: strongly reduced interaction with 3′-sulfated oligosaccharides^144^
	1 Trp	bio4HW	Gal-1[bio4HW]	7 mg/L^144^	tuning biophysical properties of binding proteins: glycan binding profile comparable to parent protein^144^
	1 Trp	bio5HW	Gal-1[bio5HW]	10 mg/L^144^	tuning biophysical properties of binding proteins: glycan binding profile comparable to parent protein^144^
	1 Trp	bio4NW	Gal-1[bio4NW]	10 mg/L^144^	tuning biophysical properties of binding proteins: only subtle changes in glycan binding profile relative to parent protein^144^
GFP	6 Met	Hpg	GFP[Hpg]	33 mg/L^303^	SPI combined with SCS; reactive alkyne handle for the modification with azide-PEG^303^
	10 Pro	4cFP	GFP[4cFP]	35 mg/L^303^	SPI combined with SCS; improved folding and stability^303^
GFP11.3.3	15 Leu	Tfl	GFP11.3.3[Tfl]	n.a.	adaptation of the protein structure to ncAA incorporation: directed evolution of GFP to fit Tfl into the structure^300^
GFP K15am	6 Met, 10 Pro	Dopa	GFP K15Dopa	11.7 mg/L^303^	SPI combined with SCS; reactive catechol handle for directed immobilization on chitosan and polystyrene beads^303^
	6 Met, 10 Pro	Dopa, 4cFP	GFP K15Dopa[4cFP]	7.8 mg/L^303^	SPI combined with SCS; improved folding and stability; reactive catechol handle for directed immobilization on chitosan and polystyrene beads^303^
	6 Met, 10 Pro	Dopa, Hpg	GFP K15Dopa[Hpg]	6.5 mg/L^303^	SPI combined with SCS; reactive catechol handle for directed immobilization on chitosan and polystyrene beads; reactive alkyne handle for the modification with azide-PEG^303^
GFP^M1^	1 Met	Hpg	GFP^M1^[Hpg]	n.a.	reactive alkyne handle for attachment of cleavable biotin probes^99^
GFP^M50 M134 M143^	3 Met	Aha	GFP^M50 M134 M143^[Aha]	2.58 mg/L^131^	reactive azide handle for dendronylation^131^
GFP Y66am	5 Met	Aha, Dopa	GFP Y66Dopa[Aha]	3.9 mg/L^118^	SPI combined with SCS^118^
		Hpg, Dopa	GFP Y66Dopa[Hpg]	4.1 mg/L^118^	SPI combined with SCS; slightly red-shifted excitation (509 nm) and emission maxima (517 nm) compared to parent GFP (λ_ex max_ 501 nm; λ_em max_ 513 nm)^118^
GFP-1M	1 Met	bioAha	GFP-1M[bioAha]	9.7 mg/L^140^	SPI of biosynthesized Aha; biosynthesis of Aha by by MetXY with NaN_3_ supplementation^140^
GFP-2M	2 Met	bioAha	GFP-2M[bioAha]	20 mg/L^140^	reactive azide handle for dendronylation; SPI of biosynthesized Aha; biosynthesis of Aha by by MetXY with NaN_3_ supplementation^140^
GFP-HS	8 Tyr	Dopa	GFP-HS[Dopa]	n.a.	tuning fluorescence properties: GFP[Dopa] is a Cu^2+^ sensor; binding of Cu^2+^ ions quenched the fluorescence^60^
	8 Tyr	bioDopa	GFP-HS[bioDopa]	n.a.	λ_ex max_ 33 nm red-shifted by Dopa incorporation in fluorophore; SPI of biosynthesized Dopa; biosynthesis of Dopa from catechol, pyruvate and ammonia by *Cf*TPL^110^
GFPcon	6 Met	Hpg	GFPcon[Hpg]	5.7 mg/L^189^	fluorescence of fast folding GFP^S65G S72A^ mutant GFPcon impaired (GFPcon[Met] 15.6 mg/L)^189^
	6 Met	Nle	GFPcon[Nle]	3.5 mg/L	ditto^189^
GFPhs1	6 Met	Aha	GFPhs1[Aha]	19.7 mg/L^189^	adaptation of the protein structure to ncAA incorporation: GFPhs1 contains 14 mutations in comparison to GFP, accommodates Met analogs better than GFPcon (GFPhs1[Met] 57.8 mg/L)^189^
	6 Met	Eth	GFPhs1[Eth]	25.6 mg/L^189^	ditto^189^
	6 Met	Hpg	GFPhs1[Hpg]	28.8 mg/L^189^	ditto^189^
	6 Met	Mox	GFPhs1[Mox]	9.4 mg/L^189^	ditto^189^
	6 Met	Nle	GFPhs1[Nle]	16.8 mg/L^189^	ditto^189^
GFPhs1-RM	1 Met	Hpg	GFPhs1-RM[Hpg]	n.a.	reactive alkyne handle for the attachment of a cell-penetrating peptide for intracellular delivery^135^
cfGFPhs1-RM(M1)	1 Met	bioAhc	cfGFPhs1-RM[M1bioAhc]	∼10 mg/L^136^	SPI of biosynthesized Ahc; cfGFPhs1-RM(M1) generated by introducing mutations C70S C48S into GFPhs1-RM to remove cysteine residues; Q as the penultimate residue prevents N-terminal residue excision; 57% incorporation efficiency^136^
cfGFPhs1-RM(M134)	1 Met	bioAhc	cfGFPhs1-RM[M134bioAhc]	∼10 mg/L^136^	SPI of biosynthesized Ahc; single internal Met, N-terminal Met excised upon His_6_-tag cleavage; 86% incorporation efficiency
cfGFPhs1-RM(M134 M143)	2 Met	bioAhc	cfGFPhs1-RM(M134bioAhc M143bioAhc)	∼10 mg/L^136^	SPI of biosynthesized Ahc; two internal Met residues, N-terminal Met excised upon His_6_-tag cleavage; 71% incorporation efficiency^136^
GFPhs2	6 Met	Aha	GFPhs2[Aha]	16.2 mg/L^189^	adaptation of the protein structure to ncAA incorporation: GFPhs2 contains 23 mutations in comparison to GFP, accommodates Met analogs better than GFPcon but slightly worse than GFPhs1 (GFPhs2[Met] 56.8 mg/L)^189^
	6 Met	Eth	GFPhs2[Eth]	10.2 mg/L^189^	ditto^189^
	6 Met	Hpg	GFPhs2[Hpg]	18.4 mg/L^189^	ditto^189^
	6 Met	Mox	GFPhs2[Mox]	5.4 mg/L^189^	ditto^189^
	8 Tyr	3FY	GFPhs2[3FY]	38 mg/L^62^	potential pH sensor in the range pH 3–8 ^62^
	8 Tyr	Dopa	GFPhs2[Dopa]	32 mg/L^61^	reactive catechol for conjugation to the aminopolysaccharide chitosan^61^
	8 Tyr	Dopa	GFPhs2[Dopa]	n.a.	selective and reversible biosensor for Al^3+^ ions; senses Al^3+^ in living E. coli cells^111^
GFPmut3.1	6 Met	Hpg	GFPmut3.1[Hpg]	n.a.	contains reactive alkyne handle; correct protein folding hampered by Hpg^58^
	6 Met	Nle	GFPhs2[Nle]	6.2 mg/L^189^	adaptation of the protein matrix to ncAA incorporation: GFPhs2 is GFP with 23 mutations, accommodates Met analogs better than the GFPcon parent but slightly worse than GFPhs1; GFPhs2[Met] 56.8 mg/L^189^
GFPrm_AM	7 Met	Aha	GFPrm_AM[Aha]	n.a.^112^	adaptation of the protein structure to ncAA incorporation: structure of GFPrm_AM optimized to accommodate Met analogs; screening marker for MetRS libraries^112^
	7 Met	Hpg	GFPrm_AM[Hpg]	n.a.^112^	ditto^112^
	7 Met	Nle	GFPrm_AM[Nle]	n.a.^112^	ditto^112^
	7 Met	Pra	GFPrm_AM[Pra]	n.a.	Pra incorporated when PraRS expressed from multicopy plasmid^270^
	7 Met	Tfn	GFPrm_AM[Tfn]	21 mg/L^112^	Tfn incorporated when TfnRS expressed from multicopy plasmid; fluorescence of fluorinated GFPrm_AM comparable to parent protein, Tfn well accommodated by the protein matrix^112^
GroESM2	2 Met	Hpg	GroESM2[Hpg]	n.a.	telechelic protein^59^
Grx^C^	1 Cys	Sec	Grx^C^[Sec]	0.7 mg/L^235,237^	enzyme design: Sec in the active center of glutaredoxin provoked glutathione peroxidase activity^237^
GTL	7 Met	bioAha	GTL[bioAha]	5 mg/L^140^	SPI of biosynthesized Aha; biosynthesis of Aha by by MetXY with NaN_3_ supplementation^140^
H3^M120^	1 Met	Ahc	H3^M120^[Ahc]	2.4 g/L^64^	reactive alkene handle for cross-metathesis with allyl alcohol^64^
HBV	2 Met	Aha	HBV[Aha]	20–30 mg/L^98^	virus-like particle with reactive azide surface handles; 95% incorporation efficiency; ∼25% of the azide handles conjugated to alkyne-fluorophore by CuAAC; potential nanocarrier^98^
HBV^M66S^	1 Met	Aha	HBV^M66S^[Aha]	40–60 mg/L^98^	virus-like particle with reactive azide surface handles; 90% incorporation efficiency; ∼55% of the azide handles conjugated to alkyne-fluorophore by CuAAC; potential nanocarrier^98^
hEGF	1 Met	Aha	hEGF^ψR2^[Aha]	n.a.	tuning of N-terminal residue excision; Aha not cleaved^132^
	1 Met	Aha	hEGF^ψR2G^[Aha]	n.a.	tuning of N-terminal residue excision; Aha impairs cleavage^132^
	1 Met	Hpg	hEGF^ψR2^[Hpg]	n.a.	tuning of N-terminal residue excision; Hpg not cleaved^132^
	1 Met	Hpg	hEGF^ψR2G^[Hpg]	n.a.	tuning of N-terminal residue excision; Hpg inhibits cleavage^132^
	3 Met	Aha	hEGF^τ^[Aha]	n.a.	tuning of N-terminal residue excision; Aha impairs cleavage^132^
	3 Met	Hpg	hEGF^τ^[Hpg]	n.a.	tuning of N-terminal residue excision; Hpg strongly impairs cleavage^132^
hSOD1	1 Met	Hpg	hSOD1[Hpg]	0.05 mg/L^154^	SPI of Hpg in S. cerevisiae inefficient^154^
	1 Met	Nle	hSOD1[Nle]	5 mg/L^154^	SPI in S. cerevisiae; titer increases with the supplementation level of the ncAA^154^
hUb	1 Met	Ahc	hUb[Ahc]	n.a.	reactive alkene handle for cross-metathesis with allyl alcohol^64^
	3 Pro	4cFP	hUb[4cFP]	n.a.	4cFP not incorporated^121^
	3 Pro	4tFP	hUb[4tFP]	14 mg/L^121^	improved stability^121^
hu-MscFv	8 Pro	4cFP	hu-MscFv[4cFP]	n.a.	folding unaltered^219^
	8 Pro	4tFP	hu-MscFv[4tFP]	n.a.	improved folding^219^
IFNβ	1 Met	Aha	IFNβ^2A^[Aha]	n.a.	tuning N-terminal residue excision; complete processing^174^
	1 Met	Aha	IFNβ^2S^[Aha]	n.a.	tuning N-terminal residue excision; incomplete processing^174^
	1 Met	Aha	IFNβ^2G^[Aha]	n.a.	ditto^174^
	1 Met	Aha	IFNβ^2H^[Aha]	n.a.	tuning N-terminal residue excision; no cleavage^174^
	1 Met	Aha	IFNβ^2Q^[Aha]	n.a.	ditto^174^
	1 Met	Aha	IFNβ^2E^[Aha]	n.a.	ditto^174^
	1 Met	Hpg	IFNβ^2A^[Hpg]	n.a.	tuning N-terminal residue excision; complete processing^174^
	1 Met	Hpg	IFNβ^2S^[Hpg]	n.a.	tuning N-terminal residue excision; incomplete processing^174^
	1 Met	Hpg	IFNβ^2G^[Hpg]	n.a.	ditto^174^
	1 Met	Hpg	IFNβ^2H^[Hpg]	n.a.	tuning N-terminal residue excision; no cleavage^174^
	1 Met	Hpg	IFNβ^2Q^[Hpg]	n.a.	ditto^174^
	1 Met	Hpg	IFNβ^2E^[Hpg]	n.a.	ditto^174^
IgG-Fc^M32 M208^	2 Met	Ahc	IgG-Fc^M32 M208^[Ahc]	1 mg/L^64^	SPI in HEK293 cells; reactive alkene handle for cross-metathesis with allyl biotin; incorporation of Ahc only at position Met32 but not at Met208 ^64^
KlenTaq	32 Pro	4cFP	KlenTaq[4cFP]	n.a.	incorporation failed^76^
	32 Pro	4tFP	KlenTaq[4tFP]	0.2–0.5 mg/L^76^	retained fidelity, activity, and sensitivity, some loss in thermostability;^76^ crystallized better than parent; PDB 4DLE^220^
KSI	2 Trp	BNW	KSI[BNW]	n.a.	spectroscopic probe; fluorescence red-shifted relative to parent protein^119^
lichenicidin Bliα	1 Met	Aha	Bliα[Aha]	n.a.	antibacterial activity of Bliα[Aha]/Bliβ equal to parent protein^93^
	1 Met	Eth	Bliα[Eth]	n.a.	antibacterial activity of Bliα[Eth]/Bliβ nearly equal to parent protein^93^
	1 Met	Hpg	Bliα[Hpg]	n.a.	antibacterial activity of Bliα[Hpg]/Bliβ slightly lower than parent protein^93^
	1 Met	Nle	Bliα[Nle]	n.a.	antibacterial activity of Bliα[Nle]/Bliβ nearly equal to parent protein^93^
	2 Pro	4cFP	Bliα[4cFP]	n.a.	n.a.^93^
	2 Pro	4tFP	Bliα[4tFP]	n.a.	n.a.^93^
	2 Pro	4tHP	Bliα[4tHP]	n.a.	n.a.^93^
	2 Pro	Thz	Bliα[Thz]	n.a.	n.a.^93^
lichenicidin Bliβ	1 Trp	[3,2]Tpa	Bliβ[[3,2]Tpa]	n.a.	2-fold less Bliβ[3,2]Tpa than Bliβ parent, antimicrobial activity of both proteins equal^81^
	1 Trp	4FW	Bliβ[4FW]	n.a.	n.a.^93^
	1 Trp	5HW	Bliβ[5HW]	n.a.	n.a.^93^
	1 Trp	7AW	Bliβ[7AW]	n.a.	n.a.^93^
LuGST1-1^C^	1 Cys	Sec	LuGST1-1^C^[Sec]	1.13 mg/L^236^	enzyme design: Sec in the active center of glutathione transferase provokes glutathione peroxidase activity^236^
	1 Cys	Tec	LuGST1-1^C^[Tec]	n.a.	enzyme design: glutathione transferase with a catalytic Tec rivals natural glutathione peroxidase activity^235^
LzipA1	8 Leu	(2*S*,4*R*)Tfl	LzipA1[(2*S*,4*R*)Tfl)]	9 mg/L^88^	increased thermostability^88^
	8 Leu	(2*S*,4*S*)Tfl	LzipA1[(2*S*,4*S*)Tfl)]	18 mg/L^88^	ditto^88^
	8 Leu	Alg	LzipA1[Alg]	n.a.	incorporation of the Met analog by overexpression of the editing deficient mutant *Ec*LeuRS^T252Y 100^
	8 Leu	H4y	LzipA1[H4y]	n.a.	ditto^100^
	8 Leu	Hag	LzipA1[Hag]	n.a.	ditto^100^
	8 Leu	Hil	LzipA1[Hil]	10 mg/L^103^	incorporation of the Leu analog by overexpression of *Ec*LeuRS; increased thermostability^103^
	8 Leu	Hpg	LzipA1[Hpg]	n.a.	incorporation of the Met analog by overexpression of the editing deficient mutant *Ec*LeuRS^T252Y 100^
	8 Leu	Nle	LzipA1[Nle]	n.a.	ditto^100^
	8 Leu	Nva	LzipA1[Nva]	n.a.	ditto^100^
	8 Leu	Onv	LzipA1[Onv]	n.a.	reactive ketone handle for oxime coupling; incorporation by overexpression of the editing deficient mutant *Ec*LeuRS^T252Y 101^
LzipA1^S31 M D34F^	1 Met, 2 Phe	Aha, 4yF	LzipA1^S31 M D34F^[Aha,4yF]	5.8 mg/L^182^	stapled peptide; thermostability greatly improved^182^
LzipA1^D52 M A55F^	1 Met, 2 Phe	Aha, 4yF	LzipA1^D52 M A55F^[Aha,4yF]	7.8 mg/L^182^	ditto^182^
MazF-bs	7 Arg	Can	MazF-bs[Can]	n.a.	endoribonuclease with altered cleavage motif; specifically cleaves the motif U#ACAUA with one extra 3′-A instead of U#ACAU^77^
M200	5 Met	Aha	M200[Aha]	n.a.	random immobilization on biosensor; no sensitivity increase^48^
M200^OmpA^	1 Met	Aha	M200^OmpA^[Aha]	n.a.	directed immobilization on biosensor; 800-fold more sensitive for antigen peptide, and 10 times more sensitive toward FMDV^48^
mDHFR	14 Ile	(2*S*,3*S*)enI	mDHFR[(2*S*,3*S*)enI]	22 mg/L	stereoselective incorporation of the unsaturated Ile analog (2*S*,3*S*)enI^258^
	14 Ile	(2*S*,3*R*)enI	mDHFR[(2*S*,3*R*)enI]	n.a.	protein not expressed^258^
	14 Ile, 14 Val	(2*S*,3*S*)Tfv	mDHFR[(2*S*,3*S*)Tfv]	n.a.	(2*S*,3*S*)Tfv not incorporated^109^
	14 Ile, 14 Val	(2*S*,3*R*)Tfv	mDHFR[(2*S*,3*R*)Tfv]	n.a.	(2*S*,3*R*)Tfv incorporated at Ile codons when *Ec*IleRS was overexpressed or at Val codons when *Ec*ValRS was overexpressed^109^
	8 Met	Anl	mDHFR[Anl]	18.1 mg/L^85^	reactive azide handle; mDHFR[Anl] was produced by expressing the *Ec*MetRS^L13G^ mutant from a multicopy plasmid;^85^
					mutant MetRS^NLL^ improves Anl incorporation even in the presence of Met^102^
	8 Met	Tfn	mDHFR[Tfn]	31 mg/L^112^	efficient incorporation of Tfn with the evolved mutant *Ec*MetRS^SLL112^
mDsRed	12 Tyr	3FY	mDsRed[3FY]	n.a.	λ_ex max_ 12 nm blue-shifted and improved quantum yield relative to parent; analog incorporation confirmed only for the fluorophore by mass analysis of the corresponding proteolytic fragment and by fluorescence spectroscopy^190^
	12 Tyr	3NY	mDsRed[3NY]	n.a.	λ_ex max_ 12 nm red-shifted and improved quantum yield relative to parent; analog incorporation confirmed only for the fluorophore by mass analysis of the corresponding proteolytic fragment and by fluorescence spectroscopy^190^
Mgfp-3	10 Tyr	Dopa	Mgfp-3[Dopa]	3–5 mg/L^147^	
				4 mg/L^78^	bioglue with greatly enhanced surface adhesion in dry and underwater environments and strong water resistance;^147^ with host *Ec*TyrRS;^78^
				∼6 mg/L	co-overexpression of *Mj*DhpRS/*Mj*tRNA_AUA_^Tyr^ ^78^
				∼7 mg/L	co-overexpression of *Ec*TyrRS^78^
Mgfp-5	20 Tyr	Dopa	Mgfp-5[Dopa]	3–5 mg/L^147^	bioglue with greatly enhanced surface adhesion in dry and underwater environments and strong water resistance^147^
mRFP1	12 Pro	4cFP	mRFP1[4cFP]	25 mg/L^122^	insoluble^122^
	12 Pro	4tFP	mRFP1[4tFP]	25 mg/L^122^	soluble but nonfluorescent^122^
mRFP1^P63A^	11 Pro	4tFP	mRFP1^P63A^[4tFP]	32 mg/L^122^	relative to parent protein enhanced thermal stability, enhanced stability toward chemical denaturation with SDS, urea and guanidinium hydrochloride; accelerated fluorophore maturation;^122^ temperature sensor^217^
mini-IGFBP-5	7 Leu	Tfl	mini-IGFBP-5[Tfl]	n.a.	low Tfl incorporation^209^
nisin	2 Met	Aha	nisin[Aha]	n.a.^256^	SPI in L. lactis; higher antimicrobial activity than parent;^71^
				8.3 mg/L	improved cross-expression system; antimicrobial activity 30% higher than parent^256^
	2 Met	Alg	nisin[Alg]	n.a.	SPI in L. lactis; Alg not incorporated^71^
	2 Met	Eth	nisin[Eth]	n.a.^256^	SPI in L. lactis; inefficient production, antimicrobial activity not assessed;^71^
				5 mg/L	improved cross-expression system^256^
	2 Met	Hpg	nisin[Hpg]	n.a.	SPI in L. lactis; higher antimicrobial activity than parent^71^
	2 Met	Nle	nisin[Nle]	n.a.	SPI in L. lactis; inefficient production, antimicrobial activity not assessed;^71^
				5.3 mg/L^256^	improved cross-expression system; antimicrobial activity 5% higher than parent^256^
	2 Met	Nva	nisin[Nva]	n.a.	SPI in L. lactis; Nva not incorporated^71^
	1 Pro	[4,5]cmP	nisin[[4,5]cmP]	n.a.	tuning of antimicrobial activity^90^
	1 Pro	[4,5]tmP	nisin[[4,5]tmP]	n.a.	ditto^90^
	1 Pro	4cFP	nisin[4cFP]	n.a.	ditto^90^
	1 Pro	4tFP	nisin[4tFP]	n.a.	ditto^90^
	1 Pro	4cHP	nisin[4cHP]	n.a.	ditto^90^
	1 Pro	4tHP	nisin[4tHP]	n.a.	ditto^90^
nisin^I1W^	1 Trp	5FW	nisin^I1W^[5FW]	n.a.	SPI in L. lactis; cross-expression system to prevent misincorporation of Trp analogs into PTM enzymes; same antimicrobial activity as parent but 2-fold reduced relative to wild-type nisin^255^
	1 Trp	5HW	nisin^I1W^[5HW]	n.a.	ditto; 4-fold lower antimicrobial activity than wild-type nisin^255^
	1 Trp	5mW	nisin^I1W^[5mW]	n.a.	ditto^255^
nisin^I4W^	1 Trp	5FW	nisin^I4W^[5FW]	n.a.	ditto; 2-fold lower antimicrobial activity than wild-type nisin^255^
	1 Trp	5HW	nisin^I4W^[5HW]	n.a.	ditto^255^
	1 Trp	5mW	nisin^I4W^[5mW]	n.a.	ditto^255^
nisin^M17I^	1 Met	Aha	nisin^M17I^[Aha]	n.a.	SPI in L. lactis; bioorthogonal conjugation with peptide and fluorophore and cross-linked with nisin^M17I^[Hpg] by CuAAC;^71^ improved cross-expression system; antimicrobial activity equals parent^256^
	1 Met	Eth	nisin^M17I^[Eth]	n.a.	SPI in L. lactis; improved cross-expression system; antimicrobial activity lower than parent^256^
	1 Met	Hpg	nisin^M17I^[Hpg]	n.a.	SPI in L. lactis; bioorthogonal conjugation with peptide and fluorophore and cross-linked with nisin^M17I^[Aha] by CuAAC^71^
	1 Met	Nle	nisin^M17I^[Nle]	n.a.	SPI in L. lactis; improved cross-expression system; antimicrobial activity lower than parent^256^
nisin^M17W^	1 Trp	5FW	nisin^M17W^[5FW]	n.a.	SPI in L. lactis; cross-expression system to prevent misincorporation of Trp analogs into PTM enzymes^255^
	1 Trp	5HW	nisin^M17W^[5HW]	n.a.	ditto; same antimicrobial activity as parent but 32-fold reduced relative to wild-type nisin^255^
	1 Trp	5mW	nisin^M17W^[5mW]	n.a.	ditto^255^
nisin^M21V^	1 Met	Aha	nisin^M21V^[Aha]	n.a.	SPI in L. lactis; same antimicrobial activity as parent but outperforms wild-type nisin; bioorthogonal conjugation with peptide and fluorophore and cross-linked with nisin^M21V^[Hpg] by CuAAC;^71^ improved cross-expression system; antimicrobial activity 5% higher than parent^256^
	1 Met	Eth	nisin^M21V^[Eth]	n.a.	SPI in L. lactis; improved cross-expression system; antimicrobial activity 11% higher than parent^256^
	1 Met	Hpg	nisin^M21V^[Hpg]	n.a.	SPI in L. lactis; same antimicrobial activity as parent but outperforms parent; bioorthogonal conjugation with peptide and fluorophore and cross-linked with nisin^M21V^[Aha] by CuAAC^71^
	1 Met	Nle	nisin^M21V^[Nle]	n.a.	SPI in L. lactis; improved cross-expression system; antimicrobial activity equals parent^256^
nisin^V32W^	1 Trp	5FW	nisin^V32W^[5FW]	n.a.	SPI in L. lactis; cross-expression system to prevent misincorporation of Trp analogs into PTM enzymes^255^
	1 Trp	5HW	nisin^V32W^[5HW]	n.a.	ditto^255^
	1 Trp	5mW	nisin^V32W^[5mW]	n.a.	ditto^255^
nisin^I1M M17I M21V^	1 Met	Aha	nisin^I1M M17I M21V^[Aha]	n.a.	SPI in L. lactis; improved cross-expression system; antimicrobial activity lower than parent^256^
	1 Met	Eth	nisin^I1M M17I M21V^[Eth]	n.a.	SPI in L. lactis; improved cross-expression system; antimicrobial activity 64% higher than parent^256^
	1 Met	Nle	nisin^I1M M17I M21V^[Nle]	n.a.	SPI in L. lactis; improved cross-expression system; antimicrobial activity 7% higher than parent^256^
nisin^M17I M21V M35^	1 Met	Aha	nisin^M17I M21V M35^[Aha]	n.a.	SPI in L. lactis; bioorthogonal conjugation with peptide and fluorophore and cross-linked with nisin^M17I M21V M35^[Hpg] by CuAAC;^71^ improved cross-expression system; antimicrobial activity lower than parent^256^
	1 Met	Eth	nisin^M17I M21V M35^[Eth]	n.a.	SPI in L. lactis; improved cross-expression system; antimicrobial activity lower than parent^256^
	1 Met	Hpg	nisin^M17I M21V M35^[Hpg]	n.a.	SPI in L. lactis; bioorthogonal conjugation with peptide and fluorophore and cross-linked with nisin^M17I M21V M35^[Aha] by CuAAC^71^
	1 Met	Nle	nisin^M17I M21V M35^[Nle]	n.a.	SPI in L. lactis; improved cross-expression system; antimicrobial activity lower than parent^256^
nisinΔ^M17I^	1 Met	Aha	nisinΔ^M17I^[Aha]	n.a.	ditto^256^
	1 Met	Eth	nisinΔ^M17I^[Eth]	n.a.	ditto^256^
	1 Met	Nle	nisinΔ^M17I^[Nle]	n.a.	SPI in L. lactis; improved cross-expression system; antimicrobial activity 65% higher than parent^256^
nisinΔ^M21V^	1 Met	Aha	nisinΔ^M21V^[Aha]	n.a.	SPI in L. lactis; improved cross-expression system; antimicrobial activity lower than parent^256^
	1 Met	Eth	nisinΔ^M21V^[Eth]	n.a.	SPI in L. lactis; improved cross-expression system; antimicrobial activity 50% higher than parent^256^
	1 Met	Nle	nisinΔ^M21V^[Nle]	n.a.	SPI in L. lactis; improved cross-expression system; antimicrobial activity lower than parent^256^
Np276^M61^	1 Met	Ahc	Np276^M61^[Ahc]	1.1 mg/L^64^	reactive alkene handle for cross-metathesis with allyl alcohol^64^
OmpC	9 Met	Aha	OmpC[Aha]	n.a.	cell surface display for reactive Met analogs^84,269^
	9 Met	Aza	OmpC[Aza]	n.a.	incorporation of Aza only when E. coli MetRs was overexpressed^84^
	9 Met	Anv	OmpC[Anv]	n.a.	incorporation of Anv only when E. coli MetRs was overexpressed^84^
	9 and 5 Met	Anl	OmpC[Anl]	n.a.	incorporation of Anl only when E. coli MetRS was overexpressed; screening system for MetRS mutants^85^
OPH	5 Tyr	3FY	OPH[3FY]	5.5 mg/L^106^	tuning enzyme properties: pH-optimum of action extended to acidic pH; enhanced thermal stability at alkaline pH^106^
PA6	1 Trp	7AW	PA6[7AW]	n.a.	produced in Trp auxotrophic L. lactis^73^
	1 Trp	5FW	PA6[5FW]	n.a.	ditto^73^
	1 Trp	5HW	PA6[5HW]	n.a.	ditto^73^
	1 Trp	5mW	PA6[5mW]	n.a.	ditto^73^
PA90	5 Trp	7AW	PA90[7AW]	n.a.	ditto^73^
	5 Trp	5FW	PA90[5FW]	n.a.	ditto^73^
	5 Trp	5HW	PA90[5HW]	n.a.	ditto^73^
	5 Trp	5mW	PA90[5mW]	n.a.	produced in Trp auxotrophic L. lactis; first demonstration that 5mW can be incorporated into a recombinant protein by SPI^73^
PapD^R200H^	1 His	2FH	PapD^R200H^[2FH]	n.a.	first successful incorporation of 2FH by SPI^72^
		4FH	PapD^R200H^[4FH]	n.a.	first successful incorporation of 4FH by SPI^72^
PCAF	10 Phe	2FF	PCAF[2FF]	1.6 mg/L^86^	tuning enzyme properties: 2FF disrupts the structure of the enzyme, variant completely inactive^86^
	10 Phe	3FF	PCAF[3FF]	2.85 mg/L^86^	tuning enzyme properties: incorporation of 3FF hardly affects the protein structure, variant specific for native histone substrate^86^
	10 Phe	4FF	PCAF[4FF]	2.6 mg/L^86^	tuning enzyme properties: incorporation of 4FF slightly perturbs the protein structure, variant accepts nonhistone substrate^86^
PsbO	1 Trp	7AW	PsbO[7AW]	n.a.	pH sensor in oxygen-evolving complex of photosystem II^198^
proIns	6 Pro	3cHP	proIns[3cHP]	n.a.	3cHP incorporation at good efficiency when wild-type ProRS was overexpressed^66^
	6 Pro	3tHP	proIns[3tHP]	n.a.	3tHP incorporation at good efficiency when wild-type ProRS was overexpressed^66^
	6 Pro	4cHP	proIns[4cHP]	32 mg/L^82^	tuning biophysical properties of therapeutic proteins: hydroxylation of Pro28B accelerated the release of pharmaceutically active insulin and delayed the onset of fibrillation^82^
	6 Pro	4cmP	proIns[4cmP]	53 mg/L^66^	tuning biophysical properties of therapeutic proteins: methylation of Pro28B accelerated the release of pharmaceutically active insulin without accelerating fibril formation, the stability against physical denaturation was not affected; overexpression of ProRS for efficient incorporation of 4cmP^66^
	6 Pro	4cNP	proIns[4cNP]	n.a.	4cNP incorporation at low efficiency when wild-type ProRS was overexpressed^66^
	6 Pro	4enP	proIns[4enP]	23 mg/L^66^	tuning biophysical properties of therapeutic proteins: accelerated fibril formation; coexpression of ProRS^M157Q^ mutant from multicopy plasmid for efficient incorporation of 4enP^66^
	6 Pro	4oP	proIns[4oP]	n.a.	4oP incorporation at low efficiency when wild-type ProRS was overexpressed^66^
	6 Pro	4tHP	proIns[4tHP]	29 mg/L^82^	tuning biophysical properties of therapeutic proteins: hydroxylation of Pro28B accelerated the release of pharmaceutically active insulin and delayed the onset of fibrillation^82^
	6 Pro	4tmP	proIns[4tmP]	29 mg/L^66^	tuning biophysical properties of therapeutic proteins: methylation of Pro28B accelerated the release of pharmaceutically active insulin without accelerating fibril formation, the stability against physical denaturation was not affected; coexpression of ProRS^C443G^ mutant from multicopy plasmid for efficient incorporation of 4tmP^66^
	6 Pro	Aze	proIns[Aze]	24 mg/L^74^	Aze incorporation only when E. coli ProRS was overexpressed
	6 Pro	dhP	proIns[dhP]	23 mg/L^74^	Dhp incorporation supported by overexpression of E. coli ProRS
	6 Pro	phP	proIns[phP]	n.a.	phP incorporation at very low efficiency when wild-type ProRS was overexpressed^66^
	6 Pro	Pip	proIns[Pip]	21 mg/L^74^	Pip incorporation only when mutant ProRS^C443G^ was overexpressed
	6 Pro	Thz	proIns[Thz]	28 mg/L^74^	Thz incorporation only when E. coli ProRS was overexpressed
PTE	15 Phe	4FF	PTE[4FF]	2 mg/L^63^	tuning enzyme properties: fluorination prevented heat inactivation by stabilizing the interactions across the dimer interface^63^
Pvfp-5	17 Tyr	Dopa	Pvfp-5[Dopa]	10 mg/L^70^	coacervate microdroplets facilitate adhesive thread formation^70^
Q	7 Leu		Q[Tfl]	n.a.	coiled-coil protein with improved thermostability, self-assembles into fibers that can bind curcumin in a metal-dependent manner^366^
Qβ	1 Met	Aha	Qβ[Aha]	50–60 mg/L^98^	virus-like particle with azide reactive handles; low incorporation efficiency (∼10%) but virtually all azide moieties were conjugated to alkyne-fluorophore by CuAAC; potential nanocarrier^98^
Qβ^K16M^	2 Met	Aha	Qβ^K16M^[Aha]	25–35 mg/L^98^	virus-like particle with azide reactive handles; ∼50% incorporation efficiency (occasionally up to >90%) and near quantitative labeling with alkyne-fluorophore by CuAAC; potential nanocarrier^98^
	2 Met	Ahc	Qβ^K16M^[Ahc]	2.9 mg/L^64^	reactive alkene handle for cross-metathesis with allyl alcohol; the mature protein contained only a single Ahc; 80% of the 180 sites on the Qβ VLP were conjugated with allyl alcohol^64^
	2 Met	Hpg	Qβ^K16M^[Hpg]	40 mg/L^98^	VLP with alkyne reactive handles; ∼50% incorporation efficiency; ∼55% of the alkyne handles were conjugated to an azide-fluorophore by CuAAC; potential nanocarrier^98^
Qβ^T93M^	2 Met	Aha	Qβ^T93M^[Aha]	23–35 mg/L^98^	VLP with azide reactive handles; ∼55% incorporation efficiency; only ∼18% of the azide handles were conjugated to an alkyne-fluorophore by CuAAC; potential nanocarrier^98^
rhPrP^C^	9 Met	Mox	rhPrP^C^[Mox]	n.a.	pro-aggregation variant of rhPrP^C145^
	9 Met	Nle	rhPrP^C^[Nle]	n.a.	antiaggregation variant of rhPrP^C145^
resilin	32 Tyr	*R*32	*R*32[Dopa]	1.79 g/L^56^	forms flexible hydrogels by coordinative metal complexation that exhibit superior shaping and self-healing properties; produced at gram amounts in fed-batch fermentation^56^
	32 Tyr	Ri16	Ri16[Dopa]	1.74 g/L^56^	forms mechanically stiff and extremely stretchy hydrogels by coordinative metal complexation and covalent cross-linking that can be stretched over 18 times their original length; produced at gram amounts in fed-batch fermentation^56^
	64 Tyr	Ri32	Ri32[Dopa]	1.32 g/L^56^	forms mechanically stiff and very stretchy hydrogels by coordinative metal complexation and covalent cross-linking; produced at gram amounts in fed-batch fermentation^56^
RSL	1 Met	Aha	RSL[Aha]	n.a.	reactive azide handle for directed modification with small molecule and cross-linking; formation of superlectin^143^
	1 Met	Hpg	RSL[Hpg]	n.a.	ditto, reactive alkyne handle^143^
	7 Trp	bio4FW	RSL[bio4FW]	35–60 mg/L^142^	tuning biophysical properties of binding proteins: stability decreased, glycan binding profile comparable to parent protein; PDB 5O7W^142^
	7 Trp	bio5FW	RSL[bio5FW]	35–60 mg/L^142^	tuning biophysical properties of binding proteins: stability slightly increased, glycan binding profile comparable to parent protein; PDB 5O7V^142^
	7 Trp	bio6FW	RSL[bio6FW]	n.a.	insoluble variant
	7 Trp	bio7FW	RSL[bio7FW]	35–60 mg/L^142^	tuning biophysical properties of binding proteins: stability decreased, weaker binding of blood group B trisaccharide than parent protein; PDB 5O7U^142^
SarZΔ^M21 M60^	2 Met	Ahc	SarZΔ^M21 M60^[Ahc]	0.5 mg/L^64^	reactive alkene handle for cross-metathesis with allyl alcohol, allyl-biotin and allyl-FITC; >95% incorporation efficiency; DNA binding activity retained^64^
SH3	2 Trp	7AW	SH3[7AW]	1.5–2 mg/L^89^	noninvasive optical (fluorescence) probe; conjugation of SH3[7AW] to unlabeled SH2 domain by EPL; physicochemical properties largely retained; fluorescence spectroscopy of 7AW in the presence of Trp^89^
SsβG^M49^	1 Met	Ahc	SsβG^M49^[Ahc]	4.5 mg/L^64^	reactive alkene handle for cross-metathesis with allyl-biotin and allyl-FITC^64^
	1 Met	Sac	SsβG^M49^[Sac]	n.a.	low incorporation efficiency of Sac^83^
SSβG^M43 C439^	1 Met	Aha	SSβG^M43 C439^[Aha]	n.a.	reactive azide and thiol handles for differential artificial post-translational modification^104^
	1 Met	Hpg	SSβG^M43 C439^[Hpg]	n.a.	reactive alkyne and thiol handles for differential artificial post-translational modification^104^
suckerin-12	35 Tyr	Dopa	suckerin-12[Dopa]	25 mg/L^69^	strong underwater adhesive that can form cross-links during maturation^69^
ω-TAST-I	14 Tyr	bio2FY	TAST-I[bio2FY]	9.4 mg/L^110^	improved stability and activity; SPI of biosynthesized 2FY; biosynthesis of 2FY from 3-fluorophenol, pyruvate and ammonia by *Cf*TPL^110^
ω-TAST-II	17 Tyr	bio2FY	TAST-II[bio2FY]	14 mg/L^110^	ditto^110^
T7RNAP	25 Met	Hpg	T7RNAP[Hpg]	n.a.	accidental incorporation of Hpg putatively inactivates the enzyme^58^
tGCN5	10 Phe	2FF	tGCN5[2FF]	88 mg/L^105^	tuning enzyme properties: catalytic efficiency 16-fold lower than parent protein^105^
	10 Phe	3FF	tGCN5[3FF]	181 mg/L^105^	tuning enzyme properties: catalytic efficiency 3-fold lower than parent protein^105^
	10 Phe	4FF	tGCN5[4FF]	91 mg/L^105^	tuning enzyme properties: catalytic efficiency 6-fold lower than parent protein^105^
Trx1P	1 Pro	4cFP	Trx1P[4cFP]	5 mg/L^139^	reduced form of Trx1P[4cFP] stabilized, oxidized form destabilized^139^ PDB 4HU9 accelerated folding^222^
	1 Pro	4tFP	Trx1P[4tFP]	9 mg/L^139^	reduced form of Trx1P[4tFP] stabilized, oxidized form destabilized^139^ PDB 4HUA
	1 Pro	4cmP	Trx1P[4cmP]	n.a.	expression failed most probably because 4cmP favors a C^γ^-*exo* pucker; the single Pro76 in Trx1P preferentially adopts a C^γ^-*endo* pucker in the three-dimensional protein structure^221^
	1 Pro	4tmP	Trx1P[4tmP]	n.a.	4tmP incorporated with overexpression of *Ec*ProRS^C443G^; 4tmP favors the C^γ^-*endo* pucker and might be incorporated into Trx1P because the single Pro76 preferentially adopts a C^γ^-*endo* pucker in the three-dimensional protein structure^221^
TRX-4RepCT	6 Met	Aha	TRX-4RepCT[Aha]	n.a.	soluble spidroin mimic with reactive azide handles, e.g., to attach fluorophores or the cell-adhesion RGD motif; films cast of the material can be sterilized by heat or in 70% (v/v) ethanol^347^
TTL	11 Met	Aha	TTL[Aha]	20 mg/L^125^	tuning enzyme properties: *T*_opt_ = 5 °C and pH_opt_ 1 unit lower than parent^125^
	11 Met	Nle	TTL[Nle]	20 mg/L^125^	tuning enzyme properties: >10-fold more active than the parent protein without heat activation;^125^ 130% activity after treatment with 2-mercaptoethanol^115^
	6 Pro	4cFP	TTL[4cFP]	23 mg/L^125^	tuning enzyme properties: *T*_opt_ 20 °C lower and pH_opt_ 1 unit higher than parent protein;^125^ 4-fold more active than the parent protein in *tert*-butanol; retained 40% activity after treatment with the inhibitor Pefabloc^115^
	6 Pro	4tFP	TTL[4tFP]	29 mg/L^125^	tuning enzyme properties: *T*_opt_ 20 °C lower and pH_opt_ 1 unit higher than parent;^125^ 4-fold more active than the parent protein in *tert*-butanol and nearly 10-fold more active after treatment with the surfactant CHAPS; 120% active after treatment with DTT^115^
	6 Pro	4cHP	TTL[4cHP]	20 mg/L^125^	tuning enzyme properties: *T*_opt_ 20 °C lower and pH_opt_ 1 unit higher than parent;^125^ ∼6-fold more active than the parent protein after treatment with the surfactant CHAPS^115^
	6 Pro	4tHP	TTL[4tHP]	20 mg/L^125^	tuning enzyme properties: *T*_opt_ 15 °C lower and pH_opt_ 1 unit higher than parent protein;^125^ 140% activity after treatment with DTT^115^
	16 Phe	3FF	TTL[3FF]	50 mg/L^125^	tuning enzyme properties: 3FF variant ∼25% more active than the parent protein after heat activation; broadened substrate tolerance;^125^ retained 70% activity after treatment with the denaturant guanidinium hydrochloride^115^
	16 Phe	4FF	TTL[4FF]	67 mg/L^125^	tuning enzyme properties: 4FF variant 60% less active than parent protein after heat activation;^125^ 110% activity after treatment with 2-mercaptoethanol^115^
	2 Trp	7AW	TTL[7AW]	18 mg/L^115^	tuning enzyme properties: *T*_opt_ 5 °C lower than parent protein; 130% activity after treatment with 2-mercaptoethanol; 1.9-fold more active with the denaturant urea than without^115^
	2 Trp	4FW	TTL[4FW]	37 mg/L^115^	tuning enzyme properties: *T*_opt_ 15 °C lower and pH_opt_ 1 unit higher than parent protein^115^
	2 Trp	4NW	TTL[4NW]	42 mg/L^115^	tuning enzyme properties: *T*_opt_ 5 °C lower than parent protein; 110% activity after treatment with 2-mercaptoethanol; retained 70% activity after treatment with the denaturant guanidinium hydrochloride^115^
	7 Tyr	2FY	TTL[2FY]	6 mg/L^125^	enzyme properties not greatly changed in comparison to parent protein^125^
	7 Tyr	3FY	TTL[3FY]	13 mg/L^125^	tuning enzyme properties: 3FY-labeled enzyme inactivated by heat;^125^ ∼7-fold more active than the parent protein after treatment with the surfactant CHAPS; retained 40% activity after treatment with the inhibitor Pefabloc^115^
	16 Phe, 6 Pro, 2 Trp	4FF, 4cFP, 6FW	TTL[4FF 4cFP 6FW]	11.5 mg/L^134^	multi-ncAA SPI; fluorescence slightly red-shifted, *T*_opt_ 10 °C lower than parent, maximal activity 60% of parent^134^
TTL D221am	11 Met	Nle, Bpa	TTL D221Bpa[Nle]	6.4 mg/L^123^	SPI combined with SCS; ∼5% full-length TTL D221Bpa compared to parent protein (100%); activity-enhancing effect of global replacement of Met by Nle retained and combined with photocross reactivity of Bpa^123^
Ubq	1 Met	Ahc	Ubq[Ahc]	n.a.	n.a.^64^
W20LysM	1 Trp	5BrW	W20LysM[5BrW]	n.a.	secreted protein, produced in Trp auxotrophic L. lactis with overexpression of *Ll*TrpS^96^
	1 Trp	5FW	W20LysM[5FW]	n.a.	ditto^96^
	1 Trp	5HW	W20LysM[5HW]	n.a.	ditto^96^
	1 Trp	5mW	W20LysM[5mW]	n.a.	ditto^96^
	1 Trp	[5,6]dFW	W20LysM[[5,6]dFW]	n.a.	ditto^96^
	1 Trp	6BrW	W20LysM[6BrW]	n.a.	ditto^96^
	1 Trp	6ClW	W20LysM[6ClW]	n.a.	ditto^96^
ybbR-E_9_-LPETGG	9 Met	Aha	ybbR-E_9_[Aha]-LPETGG	10–20 mg/L^50^	reactive azide handle for the directed immobilization of a single-domain antibody on ELP E_9_, which served as a multivalent scaffold^50^
ZE-E_5GC_	5 Phe	4azF	ZE-E_5GC_[4azF]	50 mg/L^265^	artificial scaffold for the directed immobilization of target proteins fused to the basic part of the leucine zipper pair as an affinity tag; the reactive azide handle allows the photo-cross-linking to a solid support^265^

a Abbreviations: 4-OT, 4-oxalocrotonate tautomerase; 4RepCT, recombinant minimal spidroin motif; ψ-b*, pseudowild-type barstar P27A C40A C82A; ω-TA, ω-TAST, ω-transaminase from Sphaerobacter thermophillus; A_CS5_-(E_5_)_3_, A_RGD_-(E_5_)_3_, artificial extracellular matrix protein, A cell adhesion domain, E, elastin-like polypeptides; AlaDH, alanine dehydrogenase; ALE, adaptive laboratory evolution; anxA5, human annexin A5; AprA, alkaline protease from Pseudomonas aeruginosa; bio*(ncAA)*, biosynthesized ncAA; blockAE/blockCE/blockEC/blockECE, block polymers of cell-adhesion domains, e.g., CS5 or RGD (A), elastin-like polypeptides (E_2_, E_5_) or coiled-coil domain (C); *Bm*Silk, Bombyx mori silk; BRD4­(D1), bromodomain protein; *bzip*, basic leucine zipper; C, fiber forming coiled-coil protein; CalB, Candida antarctica lipase B; CapA, capistruin (RiPPs); CAT, chloramphenicol acetyltransferase; *Cf*TPL, Citrobacter freundii tyrosine phenol lyase; comp, compstatin, therapeutic peptide; CTB, cholera toxin B subunit; ECFP, enhanced cyan fluorescent protein; EGFP, enhanced green fluorescent protein; elastin, elastin–mimetic polypeptide; ELP, elastin-like protein; EYFP, enhanced yellow fluorescent protein; Fp151, artificial mussel foot protein; G_(IgG)_, IgG binding domain of protein G; Gal-1, human galectin-1; GFP, green fluorescent protein; GroESM2, M1-M86 of the chaperone GroES; Grx^C^, C105S mutant of the glutaredoxin domain of mouse thioredoxin-glutathione reductase; GTL, Geobacillus thermoleovorans lipase; H3, histone H3; HBV, Hepatitis B core antigen; hEGF, human epidermal growth factor; hSOD1, human superoxide dismutase; hUb, human ubiquitin; hu-MscFv, humanized anti-c-Met scFv; IFNβ, human interferon β; IgG-Fc^M32 M20^, Fc region of immunoglobulin G; KlenTaq, KlenTaq DNA polymerase; KSI, ketosteroid isomerase; LuGST1-1^C^, glutathione transferase S9C C86S C200S mutant from *Lucilia cuprina*; LzipA1, synthetic leucine zipper protein A1; MazF-bs, endoribonuclease from Bacillus subtilis; M200, llama heavy-chain antibody; mDHFR, murine dehydrofolate reductase; mDsRed, monomeric DsRed fluorescent protein; Mgfp, adhesive mussel foot protein from Mytilus galloprovincialis; mRFP1, monomeric red fluorescent protein; mini-IGFBP-5, recombinant human insulin-like growth factor binding protein (residues A40-I92); n.a., not assessed; Np276, right-handed β-helix pentapeptide repeat protein; OmpC, outer membrane protein C; OPH, organophosphate hydrolase; PA90, peptidoglycan binding domain of the lactococcal cell-wall-hydrolyzing enzyme, PA6 is part of PA90; PapD^R200H^, single His mutant of the chaperone PapD; PCAF, p300/CBP associated factor, a histone acetyltransferase; pH_opt_, optimal pH; PsbO photosystem II subunit; proIns, proinsulin; PTE, S5 phosphotriesterase from Brevundimonas diminuta; Pvfp, adhesive mussel foot protein from Perna viridis; Q, fiber forming coiled-coil protein; Qβ, bacteriophage; rhPrP^C^, recombinant human cellular prion protein; RSL, Ralstonia solanacearum lectin; SarZ, α-helix bundle DNA-binding protein; SH3, Src homology 3 (SH3) domain; SsβG, TIM-barrel β-glycosidase from Sulfolobus solfataricus; T7RNAP, phage T7 RNA polymerase; tGCN5, histone acetyltransferase from Tetrahymena thermophila; *T*_opt_, optimal temperature; Trx1P, thiol/disulfide oxidoreductase thioredoxin; TRX-4RepCT, recombinant minimal spidroin motif with N-terminal thioredoxin fusion tag; TTL, thermophilic lipase from Thermoanaerobacter thermohydrosulfuricus; Ubq, ubiquitin; W20LysM, tandem protein; ybbR-E_9_-LPETGG, elastin-like polypeptide E_9_ fused to N-terminal ybbR and C-terminal sortase A tag; ZE-E_5CG_, N-terminal fusion of the acidic subunit of a heterodimeric leucine zipper pair with elastin-like polypeptide E_5CG_; −*, the cocoon weights were assessed after incorporation of individual ncAAs and the incorporation efficiencies of the ncAAs were determined (see Section 4.2.2 for details).

## 2 Experimental Setup

An ncAA must pass a number of steps until it can form a building block of a polypeptide in a living cell (Figure 3). The first step of this journey is the uptake into the cell. Already in the early 1960s, Richmond emphasized the importance of an efficient ncAA uptake as a prerequisite for incorporation.^19^ Since then, several hundred ncAAs have been introduced into proteins,^30^ however, little is known about the intracellular uptake mechanisms (discussed in Section 5.1). Alternatively, the biosynthesis of ncAAs (Section 5.2) or their incorporation into proteins produced in cell-free systems^31^ avoids the uptake issue at all.

Next, the ncAA must be stable enough in the intracellular environment such that it accumulates to a level that allows its recognition by the AARS and charging onto the tRNA. It should neither be degraded nor excreted. The intracellular fate of ncAAs aside from their participation in ribosomal translation is even less well studied than their uptake. The notion that they are inert molecules waiting to be charged onto a tRNA is very likely too simplistic. NcAAs can be toxic if they interfere with metabolic pathways, e.g., as receptor agonists or enzyme substrates.^32,33^ Certain proline analogs can be degraded by enzymatic oxidation^34^ (Section 3.3.3) and recent studies have shown that a soil bacterium and the human gut microbiome can metabolize lysine derivatives.^35,36^

An AARS recognizes the ncAA and activates it to charge it onto its cognate tRNA(s). The AARSs of all cAAs except Asn and Gly display an impressive substrate tolerance toward more than 200 ncAAs.^27,37^ Asn and Gly are encoded by a total of six codons (Figure 1), consequently 90% of the sense codons can be reassigned to ncAAs. Some AARSs discriminate against noncognate amino acids by employing editing mechanisms,^38^ which can be mutated to improve the aminoacylation efficiency of ncAAs (Section 4.1.2).

The tRNAs play a major role in the execution of the genetic code. They directly interact with the AARSs and their anticodons base-pair with the corresponding codons on the mRNA. Certain nucleotides and structural features of the tRNA molecule, the so-called identity elements govern the interaction with the AARS and the correct aminoacylation. The anticodon may or may not represent a strong identity element.^39^ The primary tRNA transcripts are processed and the nucleotides are heavily modified. Hypermodification of the nucleotides, particularly around the anticodon effects the fidelity of translation.^40,41^ In contrast to the AARSs, tRNAs have moved into the focus of the ncAA community with some delay. This may be attributed to the intimidating complexities of these molecules that can be tackled now with streamlined analysis methods, such as tRNA Extension (tREX)^42^ and nanopore tRNA sequencing.^43^

The ncAA-charged tRNA is bound by an elongation factor, which delivers the complex to the ribosome. In prokaryotes, a single protein, the EF-Tu interacts with all charged tRNAs with nearly the same overall affinity. This is possible because the tRNAs evolved to compensate for the variable binding of the cAAs. Misaminoacylation of the tRNAs with ncAAs can compromise this delicate balance.^44^ Finally, the ncAA-tRNA must be accommodated in the ribosome to allow the peptidyl transfer. NcAAs for the residue-specific incorporation are structural analogs of their canonical cAAs and thus well tolerated by the translation machinery. This structural analogy also allows the ncAAs to fit into existing protein structures without grossly perturbing their folding and/or stability. This is reflected by the fact that many 3D structures of alloproteins are isomorphous to their parent proteins. However, some analogs such as polyfluorinated ncAAs may perturb the protein structure, which can be engineered to accommodate the unusual side chains (Section 4.1.4).

A host for residue-specific ncAA incorporation must be unable to biosynthesize the corresponding cAA. It will grow only if this cAA is provided externally. On the other hand, the intracellular cAA level must be low to allow the translation of the ncAA. In other words, a codon has dual meaning: During cell growth, the SGC pertains and the cAA is incorporated at its cognate codon(s). For ncAA incorporation, the same codons are decoded by the ncAA. To allow the high-level incorporation of the ncAA with low or at best no “accidental” incorporation of the cAA, both situations must be temporally separated from each other (Figure 4). The expression of a target gene to incorporate the ncAA must be tightly controlled during each phase: It must not occur during growth with the cAA but only during the decoding with the ncAA. The ncAA will be incorporated into any newly synthesized protein, which will eventually lead to the intoxication of the host proteome. To delay this effect, strong inducible expression constructs are usually preferred, e.g., the pET vector/T7 RNA polymerase system^45,46^ in E. coli, to efficiently channel the cellular resources into the target gene expression and attenuate the production of host proteins. However, the “accidental” incorporation of the ncAA into any helper proteins that are coproduced with the target protein, e.g., T7 RNA polymerase (T7RNAP), biosynthesis enzymes or enzymes for post-translational modifications of the target protein, must be considered. To produce functional helper proteins without the ncAA, the expression of their genes should occur during the growth phase in the presence of the cAAs. This can be achieved by the temporal separation of the expression of the helper and target genes^47–50^ (see Section 5.3) or by the use of separately inducible promoters for helper and target genes.^51^

**Figure 4:**
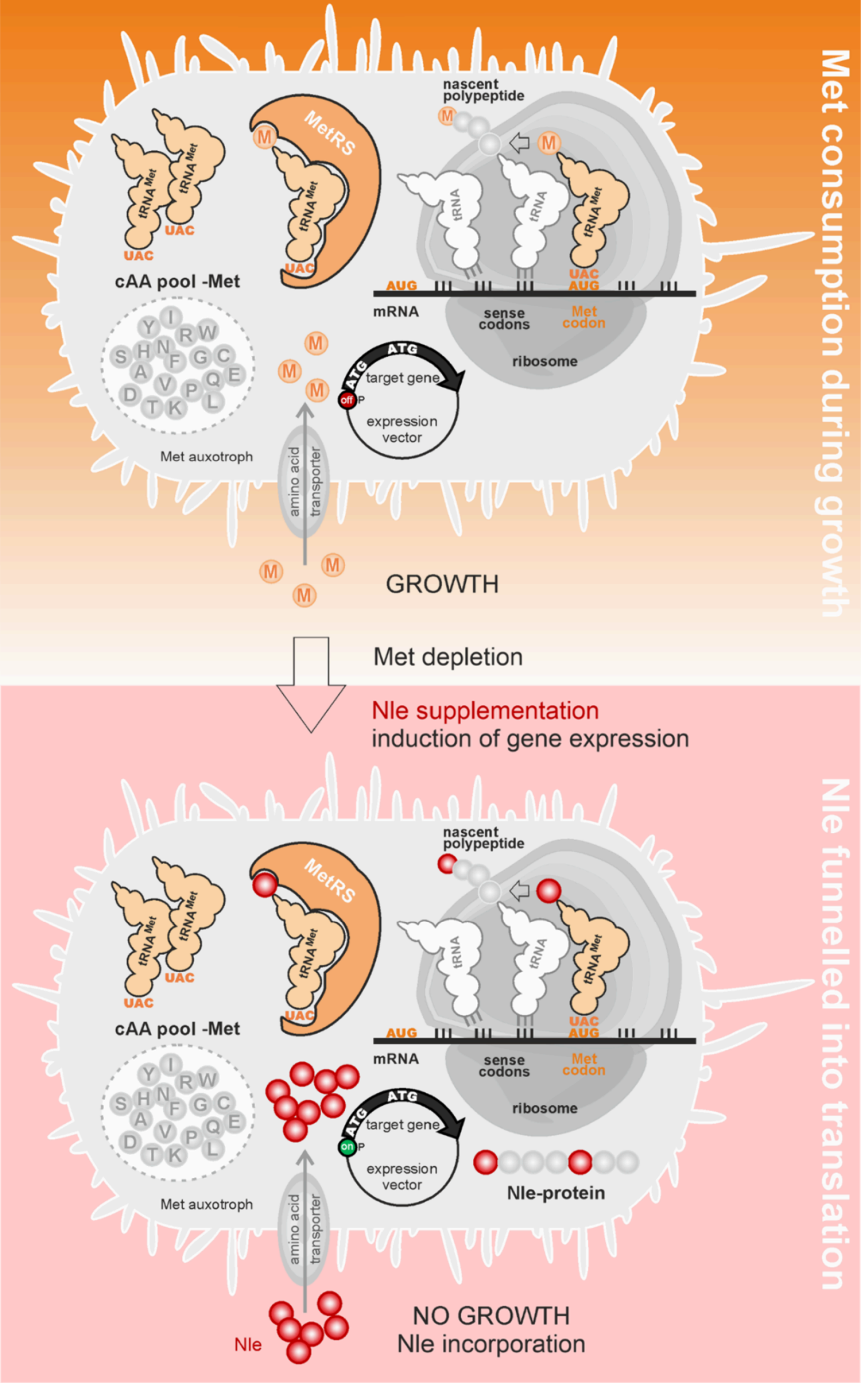
Engineering the genetic code at the example of the residue-specific incorporation of the Met analog norleucine (Nle) in a Met auxotrophic E. coli host by the cAA limitation procedure. During the growth phase in the presence of a limiting amount of Met (top), the target gene expression is turned off. Eventually, the cells will consume the Met in the medium and stop growing. Alternatively, the cells may be grown in medium with unlimited Met, washed and switched to Met-free medium. During the alloprotein production phase (bottom), Nle is supplied in the medium and the target gene expression is turned on. Methionyl-tRNA synthetase (MetRS) accepts Nle as the substrate, charges it onto tRNA_CAU_^Met^ and Nle is incorporated at AUG codons during ribosomal translation.

Two approaches are available for the separation of the growth from the production phase. The *media shift* approach first grows the expression host in medium containing the cAA. Once the cells reach a certain density, they are thoroughly washed to remove the medium containing the cAA and are resuspended in fresh medium without the cAA. To deplete intracellular cAA pools, the cells are usually incubated for a short time without the cAA. Afterward the ncAA is simply added to the medium or the cells are again washed and resuspended in medium containing the ncAA, and the expression of the target gene is turned on. The cells can be grown in complex or minimal medium containing the cAA, while the ncAA incorporation occurs in minimal medium lacking the cAA. Washes may be performed with medium or physiologic saline solution. In any case, the washes must occur under aseptic conditions.

Alternatively, a procedure that we call *cAA limitation* is available (Figure 4). Minimal medium is used throughout the experiment. The cells are inoculated into medium that contains a limiting amount of the cAA. Once the cells have consumed it, they stop growing. The growth stop signals the depletion of the cAA from the medium and that its intracellular level is too low to sustain growth. The medium is supplemented with the ncAA and the target gene expression is turned on (see video protocols for the residue-specific incorporation of Trp and Pro analogs in the literature^52,53^ ).

The latter procedure was introduced by the Budisa group under the term “**S**elective **P**ressure **I**ncorporation” (SPI).^54^ However, the procedure is a “**S**u**P**plementation **I**ncorporation” rather than a “selective pressure incorporation” because the host is not under selective pressure to incorporate the ncAA into the target protein unless this is essential for survival (which is usually not the case for recombinant proteins). Here, we use the acronym SPI in the sense of “supplementation incorporation” because both incorporation techniques, media shift and cAA limitation supplement an auxotrophic host with an ncAA. As such, SPI is well discriminated from the site-specific incorporation of ncAAs by SCS as the initially implemented method does not involve the supplementation of an auxotroph. However, a recent publication describes a host that was evolved to be auxotrophic for an ncAA to improve its incorporation by SCS.^55^ The media shift and cAA limitation procedures both have their pros and cons. For instance, the washing during the media shift takes some time and bears the risk of contamination while host cells subjected to cAA limitation might not be fully depleted for the cAA. The growth limiting concentration of the cAA must be assessed in an upfront titration experiment and it varies with the strain and the medium. Nevertheless, both procedures have been used extensively to incorporate ncAAs residue-specifically in auxotrophic hosts.

Finally, the successful incorporation of the ncAA is analyzed by mass spectrometry, e.g., electrospray ionization mass spectrometry (ESI-MS) or liquid chromatography-tandem mass spectrometry (LC-MS/MS) after proteolytic digest or by Edman degradation and amino acid analysis. The mass analysis of intact variant proteins is rather straightforward, in particular if many positions were not fully exchanged and the protein preparation is heterogeneous, which can complicate the interpretation of LC-MS/MS results.

### 2.1 SPI of ncAAs in Bacteria

E. coli is in widespread use for the SPI of a variety of analogs. Usually, the cells are grown in batch shake flask cultures. Zhu et al. reported a fed-batch fermentation procedure^56^ to introduce a Tyr analog into a recombinant protein (see Section 4.2.3) and Anderhuber and colleagues produced the Met analog l-norleucine in fed-batch fermentation cultures (Section 5.2.2.3).^57^

The media shift procedure^47–50,58–113^ is used more commonly than cAA limitation.^114–148^ The first method might be more common than the latter because it does not require to titrate the host’s limiting cAA concentration.

M9 minimal medium^149^ or New Minimal Medium (NMM)^150^ including the enhanced recipe^136,140^ are mostly used. Alternatives are Minimal Medium containing 50 mg/L of all amino acids,^151^ Andrew’s Magical Medium (AMM),^152^ SelenoMet medium^64,83^ or GMML minimal medium.^119^ M17 or defined medium were used for SPI in L. lactis.^71,73,96^ For the media shift procedure, the medium may contain the cAA whose analog will be incorporated in excess, while a limiting concentration of the cAA is added for the cAA limitation approach. The concentration of the cAA that limits the growth of a selected auxotrophic strain usually at the mid- to late-logarithmic phase in a given medium must be assessed in advance.^153^ Depending on the amino acid requirement of the auxotrophic host strain, the medium may be supplemented with other cAAs.

The cells are routinely grown to the mid- to late logarithmic phase (D_600_ = 0.5–1) and occasionally to higher cell densities.^72,75,104,136,142–144^ At this point during the media shift procedure, helper proteins such T7RNAP^47–50^ or ncAA biosynthesis genes^51^ may be induced before the addition of the ncAA to prevent its accidental incorporation. The cells are then washed 1–3 times with sterile 0.9% (w/v) NaCl solution, phosphate buffered saline (PBS) or minimal medium without cAA and ncAA. Afterward, the washed cells are resuspended in fresh minimal medium and incubated for a short period of time. Usually, 10–15 min to up to 1 h^90^ are adequate to deplete the intracellular pools of the cAA.

If the cAA limitation approach is chosen, the host is grown until it has consumed the limiting cAA and growth stalls. To securely deplete the cAA, the cells are starved for another 30–60 min. Helper proteins may be induced before the anticipated cAA depletion, which is straightforward if different promoters control the expression of the target and helper genes. If the same inducer is used, the induction of the helper genes should be short (e.g., 15 min as for the media shift procedure) to avoid the accumulation of unlabeled parent protein. The cells may be washed before the induction of the target gene expression in the presence of the ncAA.^50^

At this point, both procedures proceed equally, the ncAA is added to the medium and the target gene expression is induced. Standard ncAA supplementations are 0.5–1 mM or 50–100 mg/L but may be lower in case of very rare and/or expensive ncAAs. Higher concentrations up to 8 mM^85^ have been used as well, however, at the chosen concentration, the ncAA should be soluble in the medium and must not be toxic for the cells.

### 2.2 Eukaryotic Cells

#### 2.2.1 Yeast

The media shift approach was used for the incorporation of ncAAs in Saccharomyces cerevisiae and Komagataella phaffii (aka Pichia pastoris). The procedure was similar for both yeasts. Basically, the cells were first grown in synthetic complete medium containing all cAAs or only the essential cAA(s). Afterward, they were starved for 4 or 24 h on synthetic complete medium without the essential cAA to deplete its cellular pool. Finally, they were washed once in sterile water or phosphate buffer and shifted to synthetic complete induction medium without the cAA but containing the ncAA. The ncAAs were used in the range of 100 mg/L or 2 mM.^154,155^

#### 2.2.2 Mammalian Cells

Mammalian cells in culture have a natural requirement for 13 of the 20 cAAs: Arg, Cys, Gln, His, Ile, Leu, Lys, Met, Phe, Thr, Trp, Tyr and Val.^26^ The exact ncAA labeling procedure depends on the cell line yet it usually employs individual steps for growth, cAA depletion and incorporation of the ncAA. For instance, to incorporate Met analogs into the proteome of HEK293T cells, Schiapparelli et al. grew the cells first in DMEM medium containing 20% (v/v) fetal bovine serum (FBS) and 0.2 mM Met. Met depletion occurred for 30 min in HEPES buffered saline supplemented with Ca^2+^, Mg^2+^ and glucose, afterward 4 mM Met analog was added. Alternatively, they skipped the Met depletion and immediately supplemented the cells in buffer with 8 mM Met analog.^156^ Zhang et al. grew mouse embryonic fibroblasts and human hepatoma HepG2 cells in full culture medium containing 10% (v/v) FBS that had been dialyzed to remove the free Met. After washing the cells with PBS, they incubated them in Met-free DMEM medium with 25 μM Met analog.^157^ Wang et al. used a similar procedure for HeLa cells, they found that labeling with 50 μM Met analog in Met- or amino acid-free medium for 2 h was optimal.^158^

To label proteins in Xenopus laevis oocytes with a Met analog, Gupta et al. depleted them for Met by overnight incubation with 4 mM Met analog in Met-free buffer. On the next day, the complementary RNA encoding the target gene was injected into the oocytes and the cells were incubated with the Met analog for 3–4 days.^159^

#### 2.2.3 Insects and Rodents

For the silkworm Bombyx mori, 10 of the 20 cAAs are essential. Among them are Met and Phe. Teramoto and co-workers fed B. mori with a Met-, Phe- or Tyr-reduced diet for the residue-specific incorporation of the corresponding analogs into its silk (Section 4.2.2). Chakrabarti et al. included a 50 μM Met analog in the diet of Drosophila melanogaster.^160^ Met is essential for rodents as well,^161,162^ and Met analogs can be administered by injection.^156,163^

## 3 Classic Applications: Modulation and Analysis of Protein Structure and Function

### 3.1 Functionalization of Proteins with Reactive Handles

NcAAs are well recognized for the installation of reactive bioorthogonal handles (Figure 5) in a protein. NcAAs with azide or alkyne moieties for copper­(I)-catalyzed azide–alkyne [3 + 2] cycloaddition (CuAAC)^164^ or strain-promoted [3 + 2] azide–alkyne cycloaddition (SpAAC) (Figure 5)^165^ have found widespread application. The Met analogs l-azidohomoalanine (Aha), l-azidonorleucine (Anl), l-azidonorvaline (Anv) and l-azidoalanine (Aza) (azide group, Figure 7) as well as l-homopropargylglycine (Hpg), (2*S*)-2-aminooct-7-ynoic acid (Aoa), 2-amino-5-diazirinylnonynoic acid (phAna), l-propargylglycine (Pra) (Figure 7); the Phe analog 4-ethynyl-l-phenylalanine (4yF, Figure 9), and the Thr analog β-ethynyl-l-serine (βeS) (Figure 14) (alkyne group) are suitable ncAAs for these “click chemistry” reactions. Olefin cross-metathesis (OCM) (Figure 5)^83^ and thiol–ene “click chemistry” (TEC) (Figure 5)^166^ require alkene groups, which are present in the Met analogs *S*-allyl-l-homocysteine (Ahc) and *S*-allyl-l-cysteine (Sac) (Figure 7). The keto group of the Leu analog 4-oxo-l-norvaline (Onv) (Figure 13) facilitates oxime coupling (OxC) (Figure 5) with hydroxylamines or hydrazines.^167^ SPI relies on the recognition of the ncAAs by the wild-type host AARSs or in some cases by their mutants (see Section 4.1.2). For obvious reasons, the bioorthogonal groups in the side chains of the ncAAs that are incorporated by SPI cannot be too large. This is the reason why SpAAC works with the azidoamino acids mentioned above but not with an alkyne amino acid analog. Ring-constrained cycloalkynes would simply be too large to fit into the binding pockets of wild-type AARSs. Inverse electron demand Diels–Alder (IEDDA) reactions at alloproteins described in the literature involve large cycloalkene and tetrazine moieties^168^ that cannot be incorporated by SPI either. Nevertheless, ncAAs carrying smaller side chain cycloalkene rings such as methylcyclopropene might be applicable for SPI. More detailed information about the bioorthogonal conjugation reactions and their mechanisms can be found in topical reviews.^169–172^

**Figure 5:**
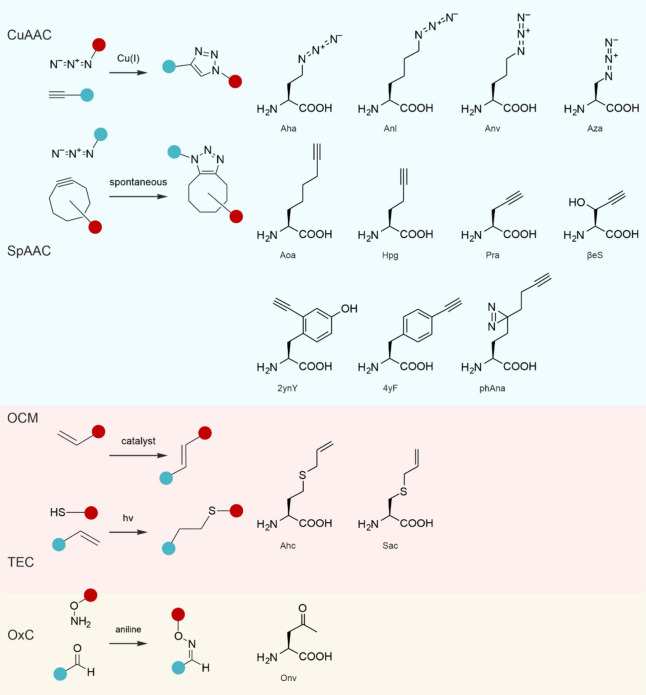
Bioorthogonal conjugation reactions and suitable noncanonical amino acids that can be incorporated by the residue-specific approach. CuAAC, copper­(I)-catalyzed [3 + 2] azide–alkyne cycloaddition; OCM, olefin cross-metathesis; OxC, oxime coupling; SpAAC, strain-promoted [3 + 2] azide–alkyne cycloaddition; TEC, thiol–ene click reaction. Noncanonical amino acids: 2ynY, 2-ethynyl-l-tyrosine; 4yF, 4-ethinyl-l-phenylalanine; Aha, l-azidohomoalanine; Ahc, *S*-allyl-l-homocysteine; Anl, l-azidonorleucine; Anv, l-azidonorvaline; Aoa, (2*S*)-2-aminooct-7-ynoic acid; Aza, l-azidoalanine; Hpg, l-homopropargylglycine; Onv, 4-oxo-l-norvaline; phAna, 2-amino-5-diazirinylnonynoic acid; Pra, l-propargylglycine; Sac, *S*-allyl-l-cysteine; βeS, β-ethynyl-l-serine.

Protein translation starts with Met or rather formylmethionine in prokaryotes, but not all mature proteins carry Met at their N-terminus. The N-terminal Met can be cleaved during translation by the enzyme methionyl aminopeptidase (MetAP, BRENDA:EC3.4.11.18). In general, bulky residues in the penultimate position following Met inhibit its excision while small amino acids do not.^173^ Merkel et al. studied whether the exchange of N-terminal Met with its analogs such as Aha or Hpg (Figure 7) affect their excision.^132^ They incorporated Aha and Hpg into different forms of the human epidermal growth factor (hEGF). hEGF^M22I^ carried a single N-terminal Met which was followed either by Arg (hEGF^ψR2^) or Gly (hEGF^ψR2G^) at the penultimate position. In hEGF^τ^, the N-terminus was extended by 21 amino acids such that the resulting protein contained three Met residues in total and Gly was always the penultimate amino acid. In agreement with the literature,^173^ the bulky Arg at the second position prevented the excision of Met, Aha and Hpg. The first residues of proteins with Gly in the penultimate position, i.e., hEGF^τ^, hEGF^τ^[Aha], hEGF^τ^[Hpg], hEGF^ψR2G^[Aha] and hEGF^ψR2G^[Hpg] (Table 2) were cleaved in the order Met > Aha > Hpg (Figure 6). However, Gly in the second position of hEGF^ψR2G^ interfered with the excision of Met, which contradicted the rule that nonbulky amino acids enhance the cleavage. In summary, reactive Met analogs can block MetAP and Hpg appears to be a stronger inhibitor of MetAP than Aha.^132^ However, this behavior may be sequence dependent. Wang et al.^174^ confirmed the conflicting role of Gly in the penultimate position but found that the penultimate residue controlled the excision of Aha or Hpg rather than the analog itself. They incorporated Aha or Hpg only at the N-terminal position of their model protein human interferon β (IFNβ, Table 2), and the penultimate residue influenced the excision of the residue at the first position in the order Ala > Ser > Gly. As expected, the excision of Aha or Hpg was blocked with His, Gln or Glu at the penultimate position (Figure 6). Taken together, the efficiency of the excision of Aha or Hpg at the ultimate position in a recombinant protein can be controlled by the choice of the penultimate residue.^174^

**Figure 6:**
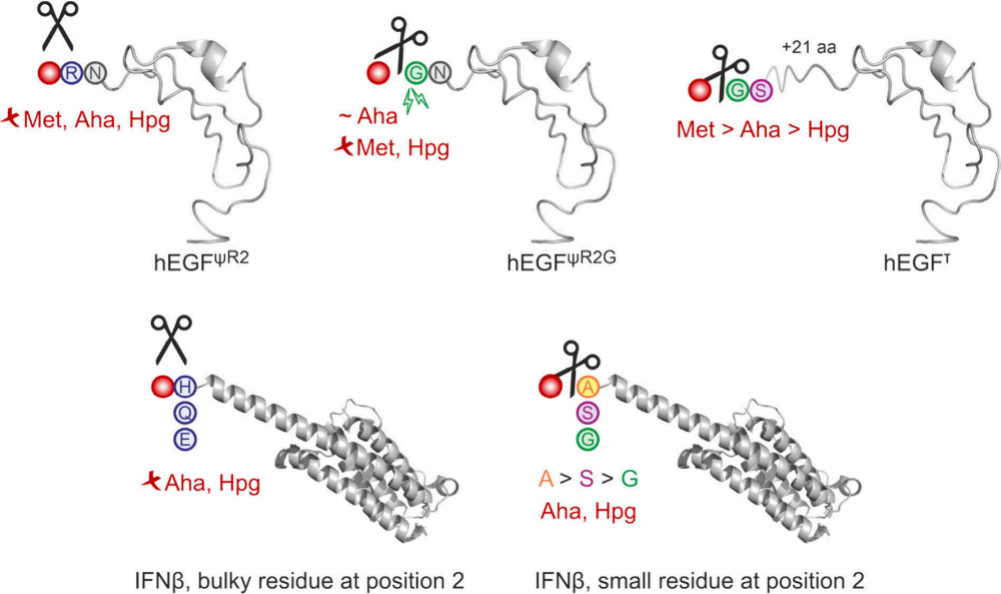
Excision of N-terminal Aha or Hpg is controlled by the penultimate residue. Bulky residues at the second position block the excision of the N-terminal residue by the methionyl aminopeptidase (MetAP, indicated by the scissors), while small residues enhance the cleavage. Despite being the smallest canonical amino acid, Gly at the penultimate position can have a conflicting role. The Budisa group found that the excision efficiency differed with the ncAA^132^ (top panel), while the Tirrell group observed that the penultimate residue controlled the excision rather than the ncAA^174^ (bottom panel).

Ayyadurai et al. prepared a telechelic protein by SPI of Hpg. They used the GroESM2 mutant which comprises amino acids Met1-Met86 of the chaperone GroES from E. coli. The amino acid sequence of this mutant starts and ends with Met, and both Met residues were quantitatively replaced by Hpg. The resulting telechelic GroESM2­[Hpg] (Table 2) could be used in a polymerization reaction with another telechelic protein carrying, e.g., compatible azide groups for CuAAC.^59^

In addition to azide and alkyne groups, which can be employed in bioorthogonal CuAAC or SpAAC reactions, ncAAs bearing alkene side chains for OCM were introduced by SPI. Lin et al. incorporated the Met analog Sac (Figure 7) into a single Met mutant of the TIM-barrel β-glycosidase SsβG^M49^ from Sulfolobus solfataricus, however, the incorporation efficiency was low and OCM was not performed with the isolated SsβG^M49^[Sac] protein (Table 2).^83^ Ten years later, Bhushan et al. confirmed the inefficient incorporation of Sac and turned their attention to another unsaturated Met analog, Ahc (Figure 7).^64^ The expression level of wild-type *Ec*MetRS in the Met auxotrophic E. coli strain B834­(DE3) was sufficient to exchange a single or multiple Met residues of the following proteins with Ahc: Qβ^K16M^ bacteriophage coat protein, histone H3^M120^, right-handed β-helix pentapeptide repeat protein Np276^M61^, S. solfataricus TIM-barrel β-glycosidase SsβG^M49^, ubiquitin (Ubq), α-helix bundle DNA-binding protein SarZ^M4 M43^ and the truncated mutant SarZΔ^M21 M60^ (Table 2). The incorporation efficiency was > 95%. Moreover, they successfully incorporated Ahc into the Fc region of immunoglobulin G (IgG-Fc^M32 M208^, Table 2) in mammalian HEK293T cells. In the eukaryotic cells, only the exchange M32Ahc occurred, Ahc was not inserted at position Met208. The authors successfully coupled the Ahc functionalized proteins with allyl alcohol or an allyl fluorophore by OCM.^64^

**Figure 7:**
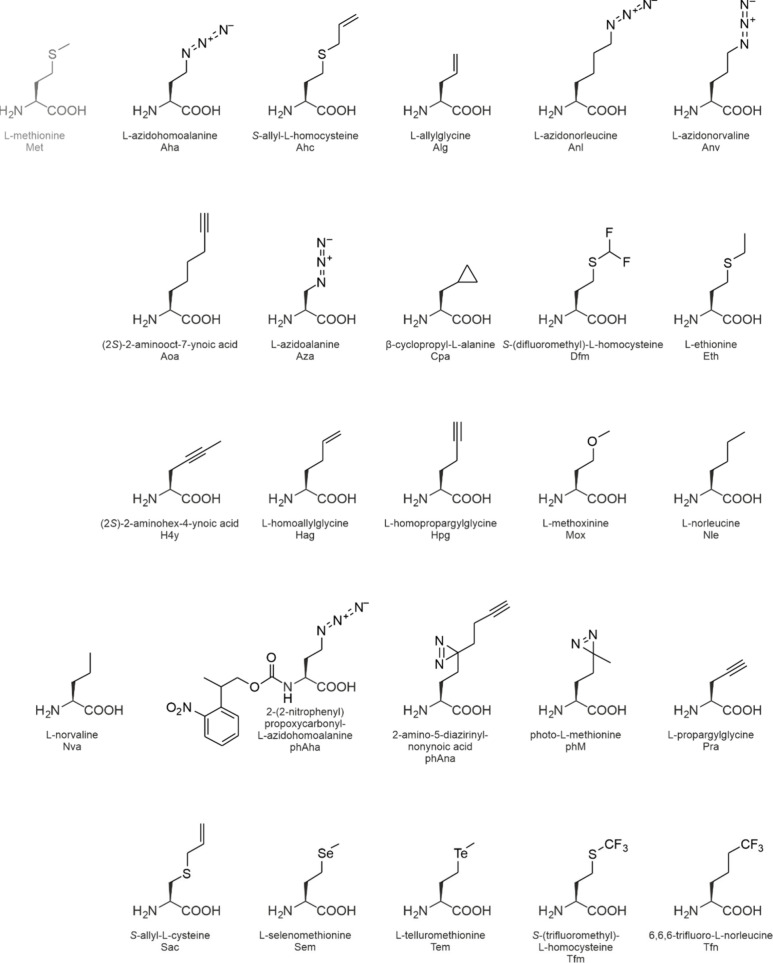
Structures of methionine analogs. The structures are arranged in the alphabetical order of their acronyms.

Eukaryotic expression hosts are preferred over prokaryotic hosts such as E. coli for the recombinant expression of large protein complexes, membrane proteins or proteins with post-translational modifications. To explore the yeast S. cerevisiae as a host to furnish proteins with reactive handles, Wiltschi et al. established a procedure for the SPI of Met analogs.^154^ Auxotrophic yeast strains are readily available from public strain banks such as EUROSCARF (http://www.euroscarf.de/) because auxotrophy markers are common for genome engineering or episomal plasmid maintenance.^175^ They used the Met auxotrophic S. cerevisiae strain Y03254 (Table 1) to introduce the Met analogs Hpg and Nle (Figure 7) into the model protein human superoxide dismutase (hSOD1). The host strain was transformed with a multicopy plasmid encoding hSOD1 and first grown in synthetic complete medium containing Met for 24 h. Then the cells were transferred to synthetic complete starvation medium for 4 h to deplete the intracellular Met pools. Finally, the expression of the target gene hSOD1 occurred for 24 h in induction medium containing Hpg or Nle. hSOD1 contained only a single N-terminal Met residue. The titer of hSOD1­[Hpg] was very low (<2% of the titer of the parent protein) and Hpg was incorporated only at a very low efficiency of 12%. To increase the product titer as well as the incorporation efficiency, they switched to Nle because Hpg would have been too expensive for a titration experiment. Supplementing Nle at twice or even 10 times the standard amino acid concentration (76 mg/L), they achieved increased incorporation efficiencies between 18 and 40%. As well, the titer of hSOD1­[Nle] rose to 5 mg/L. In summary, SPI of Met analogs in S. cerevisiae appears to be less efficient than in E. coli, however, a single study is certainly not sufficient to draw a final conclusion on that matter. Further studies with different model proteins and a more thorough analysis of the fate of the ncAA in the cells would be necessary to validate S. cerevisiae as a host for the SPI of ncAAs.

#### 3.1.1 Artificial Post-translational Modification

The reactive azide and alkyne handles introduced *via* SPI of Aha and Hpg were used to selectively modify the variant proteins. Dong et al. demonstrated the successful iodination of a protein by click chemistry (Figure 8). To this end, they incorporated Hpg into the cysteine-free mutant of barstar (ψ-b*),^176,177^ the inhibitor of the RNase barnase. ψ-b* contains a single N-terminal Met residue, which was exchanged for Hpg with very high efficiency (∼95%). ψ-b*­[Hpg] (Table 2) was produced at 80% of the titer of the parent protein. ψ-b*­[Hpg] was equally thermostable as the parent with Met, which indicated that the Met→Hpg exchange did not grossly affect the protein structure. ψ-b*­[Hpg] could be conjugated nearly quantitatively (>95%) with 2-azido-5-iodobenzoic acid by CuAAC.^178^ In a later study, Ma et al. demonstrated the modification of model proteins containing 1–3 reactive azide handles with dendrimers (Figure 8).^131^ They replaced one N-terminal Met of ψ-b*, two Met residues of the ψ-b*^M1 E47M^ mutant (ψ-b**), and three Met of the green fluorescent protein (GFP) mutant GFP^M50 M134 M143^ with Aha (Table 2). ψ-b*­[Aha] and ψ-b**­[Aha] reached 50% and 40% of the titers of their parent proteins while the GFP mutant amounted to ∼30%. The coupling with alkyne-functionalized oligoglycerol dendrons was quantitative such that no uncoupled protein species were detectable by electrospray ionization mass spectrometry (ESI-MS) analysis.

**Figure 8:**
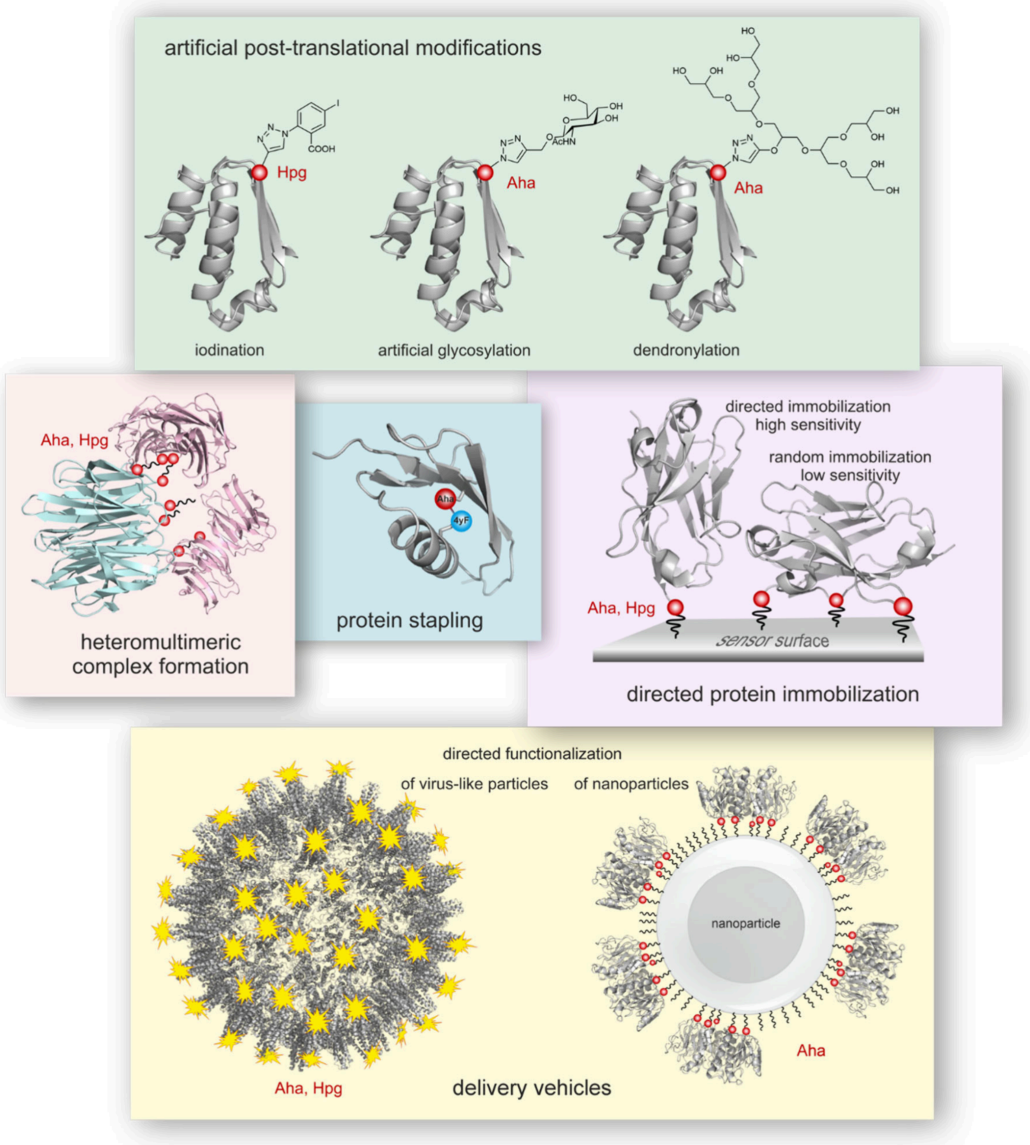
Classic applications of ncAAs to selectively modify proteins include artificial post-translational modifications such as iodination, artificial glycosylation or dendronylation. The covalent conjugation of two proteins with differing functions forms heteromultimeric bifunctional complexes. The bioorthogonal conjugation of two adjacent ncAAs with compatible reactive groups in the same polypeptide introduces stabilizing “staples”. The directed immobilization, for instance of sensor proteins on surfaces greatly improves the sensitivity of the biosensor because the ligand binding sites are maximally exposed. The functionalization of virus-like particles and nanoparticles gives access to new vehicles, e.g., for drug delivery or imaging.

Artificial glycosylation (Figure 8) represents an attractive approach to generate homogeneous protein glycoconjugates. Merkel et al. demonstrated successful artificial glycosylation of ψ-b*­[Aha].^133^ To securely prevent the excision of the N-terminal Aha (see above), they introduced two Lys residues at positions 2 and 3. Indeed, the labeling efficiency was excellent (95%) and the variant was produced at approximately 50% of the parent titer. They employed CuAAC to glycosylate ψ-b*­[Aha] directly at the N-terminus with propargyl-*N*-acetylglucosamine (alkyne-GlcNAc) and propargyl-*N*,*N*′-diacetylchitobiose (alkyne-ChiAc_2_). Both glycans, GlcNAc and ChiAc_2_ occur in natural post-translational protein glycosylation. Due to the quantitative coupling of the alkyne-glycans, homogeneous glycoforms of ψ-b*­[Aha] could be prepared. The lectin wheat germ agglutinin bound ψ-b*­[Aha]-GlcNAc and ψ-b*­[Aha]-ChiAc_2_ with high affinity. Although both artificial glycoforms of ψ-b*­[Aha] were slightly destabilized by the glycoconjugation, their ability to inhibit the RNase barnase was not impaired. The study clearly showed that SPI of ncAAs with reactive side chain groups is an appropriate approach to generate stable and homogeneous artificial glycoforms of a protein. Such artificially glycosylated protein variants might find future application in lectin-directed cell-type-specific protein targeting or lectin affinity chromatography.^133^ In a similar way, Hackenberger and co-workers artificially glycosylated Hpg variants of ψ-b*^M1 K23M E47M K79M^ (ψ-b*4M). ψ-b*4M­[Hpg] (Table 2) contained four alkyne groups that were conjugated to different azido-sugars by CuAAC. The resulting artificial tetra-glycosylated barstar variants were studied in multivalent glycan binding assays with the peanut agglutinin lectin.^117^

Davis and co-workers combined the artificial post-translational modification (PTM) of Aha and Hpg by CuAAC and of Cys residues with glycomethanethiosulfonates (glyco-MTS)^179^ to mimic differential natural PTM.^104^ While CuAAC forms a stable triazole-linkage, glyco-MTS generates a (reducible) disulfide bond. For instance, they used a mutant SSβG containing single Cys and Met residues at predefined positions. The single Met residue of SSβG^M43 C439^ was replaced by Aha using SPI to introduce an azide group in addition to the reactive thiol group of Cys439. SSβG^M43 C439^[Aha] (Table 2) was selectively α-glucosylated at Cys439 by glyco-MTS. Afterward, the Aha-azido group at position 43 was selectively β-galactosylated by CuAAC. Notably, they performed the CuAAC reaction without excess reducing agents to preserve the disulfide-linkage of sugar moiety at Cys439. SSβG^M43 C439^[Hpg] (Table 2) was similarly modified with azido-sugars, and the order of reactions (first glyco-MTS, then CuAAC) could also be reversed. CuAAC-conjugation of SSβG^M43 C439^[Aha] to the trisaccharide siaLacNAc-alkyne or the tetrasaccharide sLe^x^-alkyne in combination with the installation of a sulfotyrosine-mimic at Cys439 by glyco-MTS gave rise to functional mimics of the P-selectin-glycoprotein-ligand-1. The approach was extensively validated with another model protein, with mutants that contained single or multiple attachment sites and with different glycans (for details, see the Supporting Information of ref 104).

#### 3.1.2 Site-Selective Protein Functionalization

The selective functionalization or directed immobilization, for instance of binding proteins, is an important asset for the production of effective drug carriers and sensitive biosensors. The group of Turnbull designed a mutant of the cholera toxin B subunit (CTB) with a single Met residue, which was then exchanged against Aha. CTB binds to ganglioside GM1, which is abundant in motor neurons of the nervous system. CTB­[Aha] (Table 2) was conjugated to alkyne functionalized CdSe/ZnS core/shell nanoparticles (Figure 8) and its uptake into motor neurons traced by electron microscopy *via* silver enhancement.^180^ Nischan et al. demonstrated the intracellular delivery of GFP by the arginine-rich cell-penetrating peptide TAT (TAT).^135^ To this end, they replaced the single N-terminal Met residue of the mutant GFPhs1-RM (a descendant of GFPhs-r5M^181^) with Hpg. Then, they conjugated GFPhs1-RM­[Hpg] (Table 2) with azide-TAT by CuAAC and used confocal laser scan microscopy to trace its delivery to the cytosol and nucleus in HeLa cells.

To ease the recovery and analysis of newly synthesized proteins by bioorthogonal noncanonical amino acid tagging (for details see Section 4.3.1), Szychowski and colleagues labeled the single Met GFP^M1^[Hpg] (Table 2) variant with five different cleavable biotin probes.^99^ The probes were photolabile; cleavable with Na_2_S_2_O_4_ or trifluoroacetic acid; contained an acid-sensitive dialkoxydiphenylsilane (DADPS) motif, which can be cleaved with dilute formic acid solution or a disulfide bridge which is sensitive to reductive cleavage. The probes were azide-labeled such that they could be installed at the N-terminal Hpg residue of GFP^M1^[Hpg] by CuAAC. The release of the biotin moiety was monitored on a Western blot using fluorescence labeled streptavidin. The DAPS-biotin probe afforded the mildest, most specific and most efficient cleavage with 10% formic acid for 0.5 h (or 5% formic acid, 2 h). After the release, only a small, 143 Da protein modification was left behind, which is an asset for proteomic studies.

Tobola et al. used bioorthogonal cross-conjugation to generate so-called “superlectins”.^143^ They replaced the initiator Met residues of human galectin-1 (Gal-1) and Ralstonia solanacearum lectin (RSL) with Aha and Hpg by SPI (Table 2). Then, the azide- and alkyne-functionalized lectins were cross-linked either directly or *via* intervening polyethylene glycol (PEG) linkers carrying compatible reactive groups. The directed modular assembly generated artificial homo- and heteroconjugates (Figure 8), the latter being “superlectins” with mixed glycan specificity. Although only single reactive handles were introduced into the individual lectins, the oligomeric structure of the native proteins complicated a controlled conjugation.^143^

Link and co-workers stabilized the artificial leucine zipper A1 (LzipA1) protein as well as the small globular IgG binding domain of protein G (G_(IgG)_) by installing “staples” (Figure 8), i.e., intramolecular covalent cross-links *via* azide and alkyne handles.^182^ To genetically encode the two compatible reactive handles in the same polypeptide, they introduced a Met and a Phe codon into the leucine zipper helix of LzipA1. The codons were positioned such that after their decoding with Aha and 4yF (Figure 9), the reactive groups would be positioned on the same face of the helix in a a distance appropriate for direct cross-linking. Mutants LzipA1^S31M D34F^ and LzipA1^D52M A55F^ were designed to carry a central or a C-terminal staple, respectively. In G_(IgG)_, Aha inserted at the L21M mutation would be able to staple with 4yF at position Phe47. Using the Met/Phe double auxotrophic E. coli strain AMF-IQ (Table 1), they exchanged the Met and Phe residues of the mutants with Aha and 4yF with very high efficiency. All variants (Table 2) were subjected to CuAAC to form the staples. Stapled LzipA1^S31M D34F^[Aha,4yF] and LzipA1^D52M A55F^[Aha,4yF] showed greatly improved thermostability in comparison to the unstapled variants and G_(IgG)_^L21M^[Aha,4yF] bound IgG with nearly 4-fold increased affinity. Abdeljabbar et al. suggested that the rigid triazole-staples, which they considered more rigid than disulfide bonds, caused a structural restraint, which was responsible for the improved protein properties.^182^

**Figure 9:**
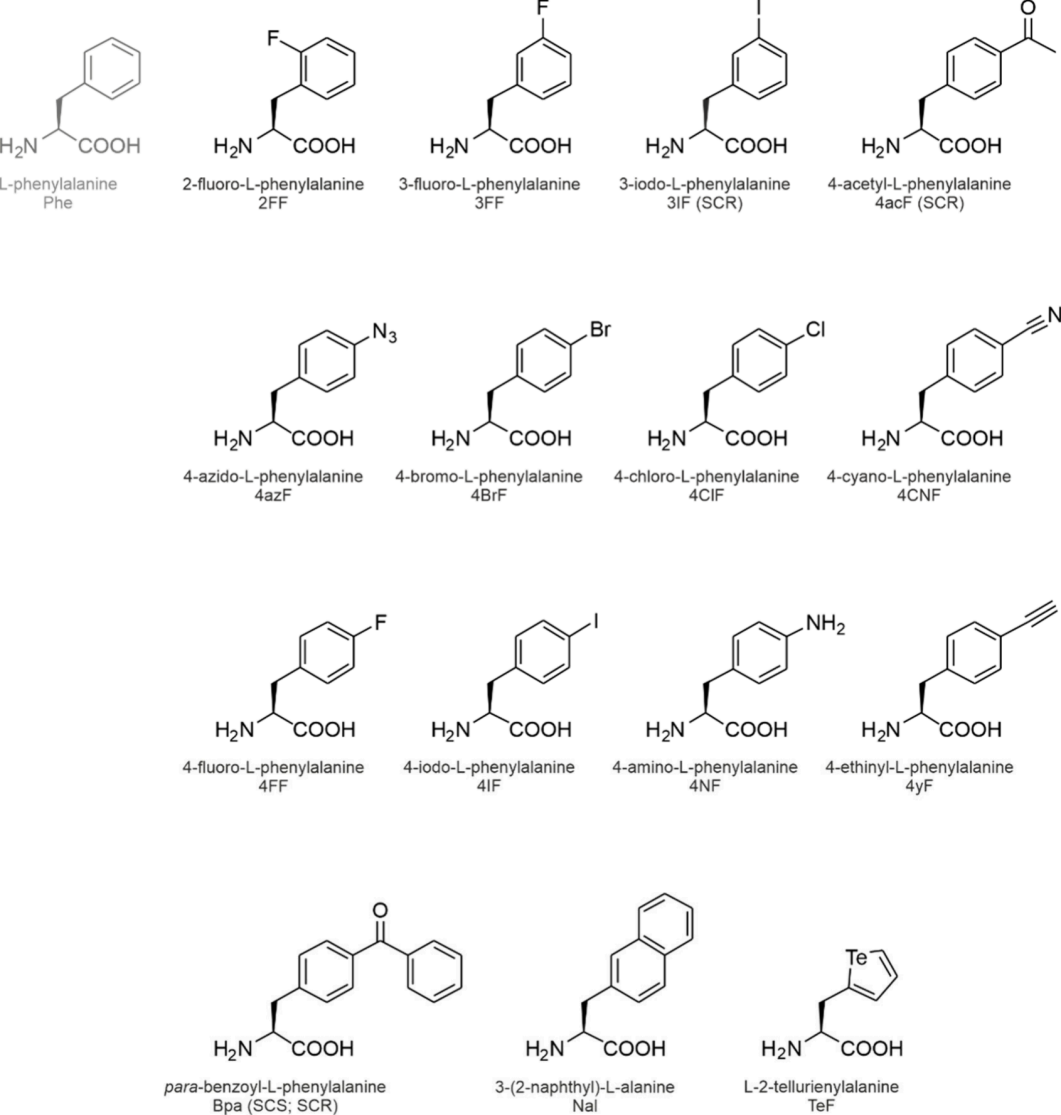
Structures of phenylalanine analogs. The structures are arranged in the alphabetical order of their acronyms. SCR, sense codon reassignment with an orthogonal ncAARS/tRNA pair; SCS, stop codon suppression with an orthogonal ncAARS/tRNA pair.

The SPI of Aha and Hpg carrying reactive azide and alkyne handles was applied to immobilize proteins. Schoffelen et al. labeled Candida antarctica lipase B (CalB, BRENDA:EC3.1.1.3) with Aha using the Met auxotrophic E. coli strain B834­(DE3)­pLysS (Table 1).^47^ The five Met residues of CalB were nearly quantitatively (90%) exchanged for Aha. CalB was expressed with an N-terminal *pelB* signal sequence for export to the periplasm of E. coli. The findings confirmed that the SPI of ncAAs occurs efficiently not only with recombinant proteins produced in the cytosol of E. coli but also when they are secreted. Four of the Aha residues in CalB­[Aha] (Table 2) were buried in its core and one was solvent exposed. After cleavage of the signal sequence, the surface exposed azide group remained available for bioorthogonal conjugation with an alkyne-fluorophore as well as alkyne-PEG5000 by CuAAC. CalB­[Aha] retained its catalytic activity when attached to the polymer although it was slightly reduced in comparison to the parent protein by the Aha incorporation and the click chemistry reagents.^47^

Directed immobilization of affinity proteins such as antibodies can dramatically increase the sensitivity, e.g., of biosensors (Figure 8). Trilling et al. introduced Aha into the llama heavy-chain antibody (VHH) M200 (5 Met) and its single Met mutant M200^OmpA^.^48^ VHH M200 and the mutant are specific for an epitope in the GH loop of the foot-and-mouth disease virus (FMDV). The single Aha of M200^OmpA^[Aha] (Table 2) allowed it to bind in a directed manner to a biosensor surface that had been functionalized with a compatible reactive group for SpAAC. In contrast, M200­[Aha] was randomly immobilized *via* its five Aha residues. The oriented immobilization of M200^OmpA^[Aha] displayed an 800-fold higher sensitivity for an epitope peptide and 10 times higher sensitivity toward the FDMV than the randomly oriented M200­[Aha].

Hepatitis B core antigen (HBV) and bacteriophage Qβ are composed of 240 and 180 units of coat proteins, respectively. The coat proteins can be recombinantly produced in E. coli and self-assemble into virus-like particles (VLPs), which are highly interesting nanocarriers for drug delivery or vaccination.^183^ Due to their multivalency, a reactive handle at a single position on the coat protein facilitates the decoration of the entire VLP with functional molecules (Figure 8). Strable et al. functionalized recombinant HBV and Qβ VLPs with azide- and alkyne-moieties by SPI of Aha and Hpg.^98^ HBV and the mutants Qβ^K16M^ and Qβ^T93M^ contained two Met residues, while the wild-type Qβ sequence and mutant HBV^M66S^ contained only the N-terminal Met. The different variants were produced in the Met auxotrophic E. coli strain M15MA/pREP4 (Table 1) at approximately 50% of the parent protein titer. The incorporation efficiency varied strongly with the incorporation position: HBV and HBV^M66S^ were nearly fully labeled at position Met1 and the first quantitatively at position Met66. In contrast, position Met1 of Qβ was populated only sparsely with Aha (∼10% efficiency; a rare sample achieved ∼90%) and not at all with Hpg. Positions K16M and T93M were fully labeled with Aha and K16 M at 50% with Hpg (Table 2). Strable et al. were able to attach an alkyne-fluorophore to HBV­[Aha] and HBV^M66S^[Aha], however, the VLPs were sensitive to the CuAAC conditions and decomposed easily so that only ∼30% and ∼50% of the available azide moieties on HBV­[Aha] and HBV^M66S^ were labeled with the dye. The Qβ variants were less sensitive to the CuAAC conditions, so that the few azide moieties on Qβ­[Aha] were nearly quantitatively labeled with the alkyne-fluorophore. The remaining variants Qβ^K16M^[Aha], Qβ^K16M^[Hpg] and Qβ^T93M^[Aha] were functionalized with alkyne-biotin, alkyne-/azide-fluorophore, alkyne-functionalized transferrin, an 80 kDa iron transporter or an azide-derivative of selenomethionine with good to very good efficiencies.

Instead of an azide or alkyne function, Ayyadurai et al. exploited the catechol group of the Tyr analog 3,4-dihydroxy-l-phenylalanine (Dopa, Figure 10) to attach GFP to the aminopolysaccharide chitosan.^61^ Oxidation of Dopa with sodium periodate forms a quinone that can react with the nucleophilic primary amines of chitosan to form a covalent adduct.^184^ Eight Tyr residues in GFPhs2 were replaced with Dopa in the Tyr auxotrophic E. coli strain JW2581 (Table 1). GFPhs2­[Dopa] (Table 2) was produced at a comparable titer to the parent protein (94%) and the incorporation of Dopa was excellent (>90% efficiency). In comparison to the parent protein, fluorescence excitation and emission maxima were red-shifted in GFPhs2­[Dopa] (λ_ex max_ 512 nm/λ_em max_ 530 nm vs λ_ex max_ 501 nm/λ_em max_ 511 nm of GFPhs2). GFPhs2­[Dopa] was oxidized with sodium periodate to generate the reactive quinone, which conjugated the protein with chitosan films and hydrogels with high efficiency (∼90%).

**Figure 10:**
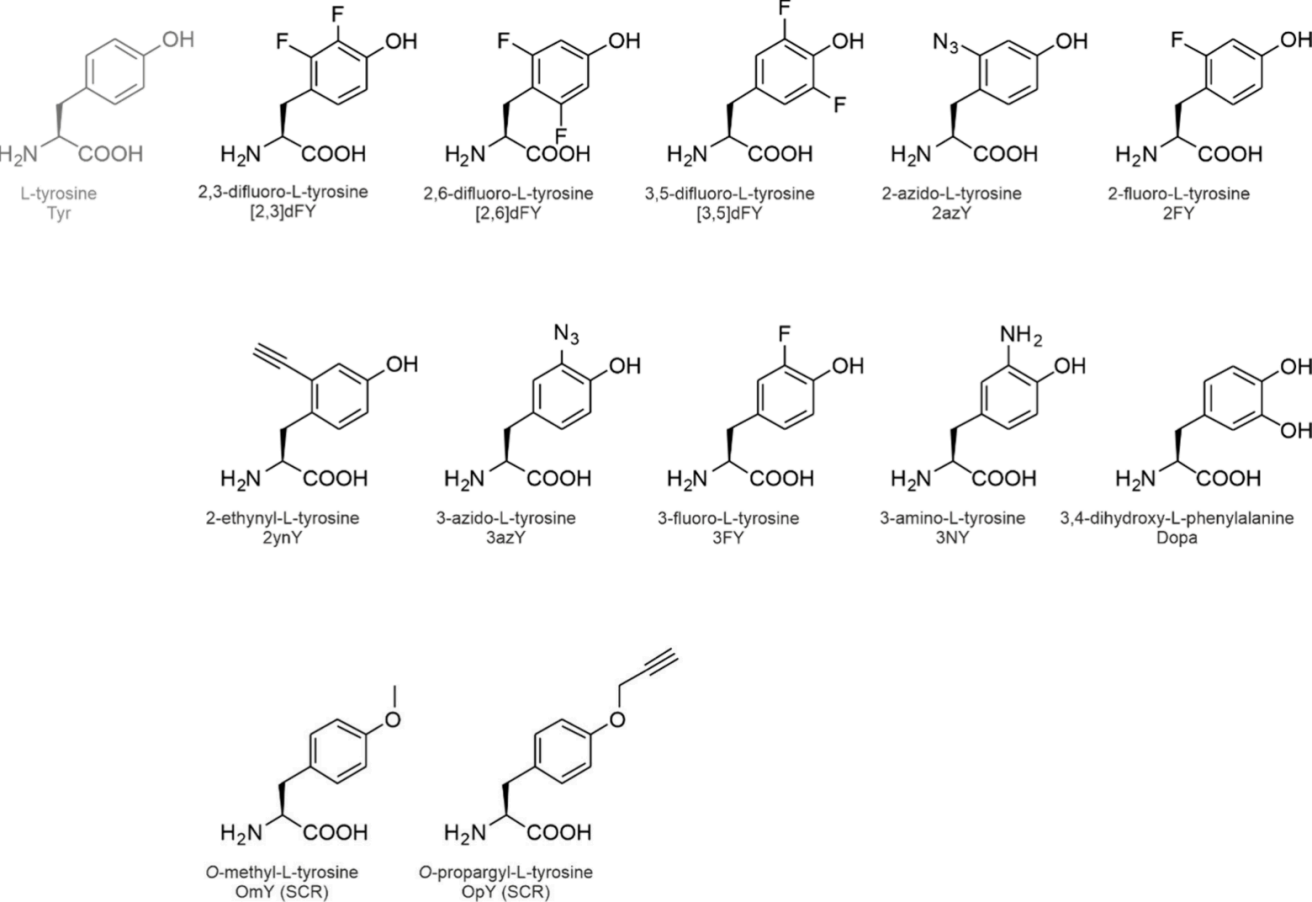
Structures of tyrosine analogs. The structures are arranged in the alphabetical order of their acronyms. SCR, sense codon reassignment with an orthogonal ncAARS/tRNA pair.

### 3.2 Tuning Fluorescence Properties: Fluorescent Biosensors

Next to the installation of reactive handles, ncAAs have been used extensively to modify the fluorescence properties of proteins. A comprehensive review by Budisa and Pal in 2004 summarized the effects of a variety of Trp analogs on the spectral properties of proteins.^185^ Here, we explore the current trends in the field.

Bae et al. quantitatively replaced all eleven Tyr residues of enhanced green fluorescent protein, EGFP (mutant GFP^F64L S65T^ ^186^) with 3-fluoro-l-tyrosine (3FY) (Figure 10) using the Tyr auxotrophic E. coli strain AT2471 (Table 1). This included residue Tyr66 in the fluorophore, which is formed by autocatalytic post-translational rearrangement of residues Ser65-Tyr66-Gly67.^187^ The Tyr→3FY exchange neither dramatically changed the spectral properties nor the structure of EGFP­[3FY] (Table 2). In comparison to the parent protein, its fluorescence emission maximum was red-shifted by 4 nm and the fluorescence intensity was ∼10% lower.^148^

In addition to EGFP, the Budisa group dissected the effects of fluorotyrosines on the spectral properties of enhanced yellow fluorescent protein, EYFP (mutant GFP^S65G V68L S72A T203Y186^; 12 Tyr residues, Tyr66 in fluorophore) and enhanced cyan fluorescent protein, ECFP (mutant GFP^F64L S65T Y66W N146I M153T V163A186^; 10 Tyr residues; Y66W in fluorophore).^137^ They replaced the Tyr residues in all GFP mutants by 2-fluoro-l-tyrosine (2FY) (Figure 10) and 3FY. The absorption maxima of EYFP­[2FY], EGFP­[2FY] and EGFP­[3FY] were blue-shifted by 10, 6, and 3 nm, while EYFP­[3FY] showed a bathochromic shift by 4 nm in comparison to the corresponding parent protein. 2FY caused a blue-shift of the emission maxima of EGFP­[2FY] and EYFP­[2FY] by 6 and 7 nm, and EYFP­[2FY] emitted a higher fluorescence intensity than its parent. In contrast, the incorporation of 3FY red-shifted the fluorescence maxima of EGFP­[3FY] and EYFP­[3FY] by 4 and 6 nm. The spectral properties of ECFP were not changed by the incorporation of any of the fluorotyrosines. Depending on the position of the fluorine substituent in the aromatic ring, the p*K*_a Ph‑OH_ of the phenolic proton in the free amino acids decreases in the order Tyr (p*K*_a Ph‑OH_ = 10.0) > 2FY (p*K*_a Ph‑OH_ = 9.0) > 3FY (p*K*_a Ph‑OH_ = 8.5), i.e., the closer the strong electron withdrawing fluorine the more acidic the phenolic proton.^188^ The p*K*_a_ values of the fluoro-Tyr fluorophores reflected this order, however the declines were low (0.1–0.4 units). The fluorophore of EYFP­[3FY] behaved otherwise, its p*K*_a_ of 5.3 was more than one unit lower compared to the parent (p*K*_a_ = 6.6). The authors identified the fluorination at the *meta* position (3F) of residues Tyr66 (in the fluorophore) and T203Y (near the fluorophore^186^) as a potential cause for this considerable decline of the p*K*_a_ in comparison to the other variant proteins. EGFP­[2FY] was more prone to aggregation than its parent, potentially because it folded less efficiently than the parent or the fluorophore did not mature as efficiently. In contrast, EYFP­[2FY] was less aggregation prone than the parent and this behavior corresponded with the increased fluorescence intensity of this variant (see above).^137^

Nagasundarapandian et al. performed a similar study with GFPhs2 whose eight Tyr residues were exchanged for 3FY. In contrast to EGFP, GFPhs2 is a heavily mutated “superfolder” GFP.^189^ The incorporation of 3FY enhanced the properties of GFPhs2 as the titer of GFPhs2­[3FY] exceeded that of the parent protein by 10% and the relative fluorescence increased by 30%. The fluorescence of GFPhs2­[3FY] linearly correlated with pH in the range of 3–8 and the variant can serve as a pH sensor on that account.^62^

Goulding et al. tuned the spectral properties of monomeric DsRed with 3FY and 3-amino-l-tyrosine (3NY) (Figure 10)^190^ The fluorescence maxima of the variant proteins were shifted by 12 nm each. The two different substituents on the Tyr ring caused opposing effects: mDsRed­[3FY] emitted blue-shifted fluorescence while the fluorescence maximum of mDsRed­[3NY] was red-shifted (Table 2). The opposite effects were most probably caused by the electron donating effect of fluorine on the conjugated electron system of the fluorophore while the amino group exerted an electron withdrawing effect. The fluorescence intensities at λ_ex max_ decreased in the order mDsRed­[3NY] > mDsRed­[3FY] > parent. The variants’ quantum yield was improved compared to the parent and the pH did not affect their fluorescence in the range of 5–11.^190^

Taken together, these studies highlight the potential of Tyr analogs, in particular of fluorotyrosines as tools to subtly tune the spectral properties of fluorescent proteins. This includes the pH sensitivity of the fluorescence, which may be changed depending on the analog and its incorporation position(s), e.g., relative to the fluorophore.

Tryptophan analogs have been used extensively to study the spectral properties of fluorescent proteins.^185^ The Budisa group replaced the single Trp57 residue outside of the fluorophore of EGFP with a palette of Trp analogs.^120^ They exchanged it for fluorinated analogs, i.e., 4-fluoro-l-tryptophan (4FW), 5-fluoro-l-tryptophan (5FW), 6-fluoro-l-tryptophan (6FW) and 7-fluoro-l-tryptophan (7FW); 4-methyl-l-tryptophan (4mW) and four different aromatic ring system variants, i.e., β-(thieno­[3,2-*b*]­pyrrolyl)-l-alanine ([3,2]­Tpa), β-(thieno­[2,3-*b*]­pyrrolyl)-l-alanine ([2,3]­Tpa), β-(selenolo­[3,2-*b*]­pyrrolyl)-l-alanine ([3,2]­Sep) and β-(selenolo­[2,3-*b*]­pyrrolyl)-l-alanine ([2,3]­Sep). The structures of the Trp analogs are shown in Figure 11. The absorbance and fluorescence intensities of variant EGFP­[[3,2]­Tpa] increased by ∼25% relative to parent, otherwise, the spectral properties of most EGFP variants did not deviate noticeably from the parent. According to their 3D structures, the overall fold of EGFP­[[3,2]­Tpa], EGFP­[[3,2]­Sep] and EGFP [[2,3]­Sep] (Table 2) was unchanged compared to EGFP parent.^120^ In contrast, the incorporation of the Tpa and Sep ring analogs into ECFP, which contains a Trp residue in the fluorophore (Trp66) in addition to Trp57, abolished the fluorescence although the proteins appeared to be correctly folded. An electron withdrawing fluorine at Trp66 in the fluorophore blue-shifted the fluorescence and lowered the fluorescence intensities of the fluorotryptophan variants relative to the parent ECFP. In contrast, an electron donating methyl group slightly red-shifted the fluorescence but did not change the intensity (Table 2).^120^

**Figure 11:**
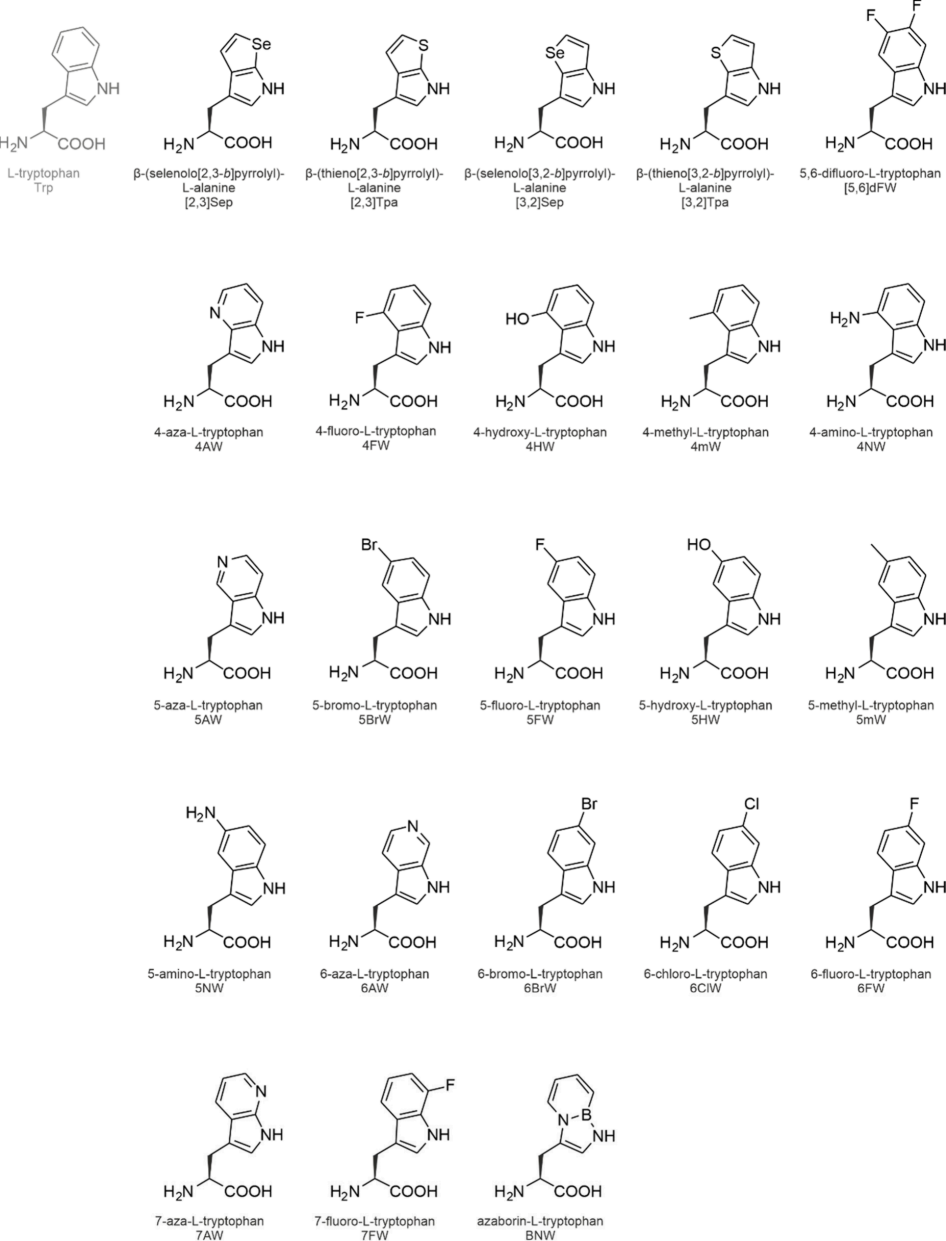
Structures of tryptophan analogs. The structures are arranged in the alphabetical order of their acronyms.

Hoesl et al. used 4-aza-l-tryptophan (4AW) and 7-aza-l-tryptophan (7AW) (Figure 11) to study the maturation of the ECFP fluorophore.^124^ ECFP contains two Trp residues. While the replacement of Trp66 in the fluorophore modulates the fluorescence as outlined above, Trp57 outside of the fluorophore is important for folding. A considerable portion of the ECFP parent protein appeared in the insoluble protein fraction (∼40%), and the incorporation of the azatryptophans enhanced the protein’s insolubility. ECFP­[7AW] (∼90%) was largely insoluble and the majority of ECFP­[4AW] (∼70%) as well (Table 2). Insoluble ECFP­[7AW] consisted of fully labeled yet nonfolded fluorophore-free protein species. The authors attributed the structure destabilizing effect to the more hydrophilic nature of the azatryptophans in comparison to Trp. The fully labeled soluble variant proteins were separated from partially labeled protein species by HPLC, and their spectral properties were analyzed. In relation to the ECFP parent, which emitted fluorescence at two maxima of 476 and 500 nm, ECFP­[4AW] showed red-shifted emission maxima (483 nm, 505 nm), while those of ECFP­[7AW] were blue-shifted (470 nm, 485 nm).^124^ Taken together, the atomic mutation of two Trp residues of ECFP with a single nitrogen atom severely affected the native protein folding and caused a shift in the fluorescence emission of 20 nm. These findings emphasize the power of ncAAs for protein manipulation even if they contain minute alterations in comparison to their canonical counterparts.

ncAAs have been used not only to tune the spectroscopic properties of fluorescence proteins but also as genuine spectral probes. For instance, Kurschus et al. fused ECFP to human annexin A5 (anxA5) and exchanged the three Trp residues of the fusion protein with 4-amino-l-tryptophan (4NW) (Figure 11).^80^ The replacement of Trp57 and Trp66 of ECFP with 4NW led to a substantial red shift of the fluorescence maximum by > 70 nm transforming it from cyan fluorescent to gold fluorescent protein (GdFP). The fluorescence of GdFP resembles DsRed (λ_em max_ 574 nm vs 593 nm, respectively), and the variant is monomeric and highly thermostable.^191^ ECFP-anxA5­[4NW] (Table 2) inherited these traits, it was gold fluorescent and did not show a propensity for oligomerization or aggregation even after prolonged refrigerated storage. The exchange of a single Trp residue in the anxA5 sequence did not interfere with the ability of ECFP-anxA5­[4NW] to detect apoptosis. Its gold fluorescence was stable in the pH range of 5.5–7.0, and the fusion protein variant reliably detected apoptosis in a variety of mammalian cell lines. To reduce the size of the apoptosis reporter, Lepthien et al. endowed anxA5 with autofluorescence in the visible range of the spectrum.^127^ They introduced biosynthesized azatryptophan analogs (see Section 5.2.5.1), which carried the nitrogen atom at position 4, 5, 6, or 7 of the indole ring (Figure 11) at the single Trp187 residue of anxA5. The variants labeled with 4AW and 5-aza-l-tryptophan (5AW) (Figure 11) were produced at the same titer as the parent protein in the Trp auxotrophic E. coli strain ATCC 49980 (Table 1). A combination of fluorescence- and mass analysis facilitated the reliable differentiation between Trp and the aza analogs despite the minute mass difference of 1 Da. 4AW most efficiently replaced Trp (>90% incorporation efficiency), while 7AW and 5AW were less efficiently incorporated (∼80% and ∼70%, respectively). Attempts to fully label anxA5 with 6-aza-l-tryptophan (6AW) (Figure 11) were unsuccessful. The fluorescence maxima of anxA5­[bio4AW] and anxA5­[bio5AW] were dramatically red-shifted by 105 and 96 nm relative to the anxA5 parent (λ_em max_ 318 nm) while they were equally stable. In contrast, the fluorescence intensity of the anxA5­[bio7AW] variant was lower and less red-shifted than the other variants (Table 2). Of the four azatryptophans, 4AW was the most promising and attractive optical probe for proteins due to its small size, the large Stokes shift of 130 nm, the high quantum yield yet low quenching tendency, and its good biocompatibility. For instance, E. coli cells producing anxA5­[bio4AW] emitted bright blue fluorescence. However, excitation and emission wavelengths covering the spectral range between (far) ultraviolet and blue render 4AW inadequate for cell biology applications.

Muir and co-workers exploited 7AW as a unique spectroscopic probe to study the folding of the human multidomain adapter protein c-Crk-I.^89^ The absorption and emission spectra of 7AW are red-shifted in comparison to Trp,^192,193^ which is why it can be selectively excited and observed in the presence of other Trp residues. c-Crk-I consists of Src homology domains 2 and 3 (SH2, SH3) each of which contains two Trp residues. While Trp169 of SH3 is involved in ligand binding, Trp170 plays an important role in the hydrophobic core of the protein. Muir et al. intended to study the biochemical and thermodynamic properties of the c-Crk-I SH3 domain in its native context, which implied the selective incorporation of 7AW in SH3 but not in SH2. SPI does not allow the experimenter to select the site of ncAA incorporation, to circumvent this shortcoming Muir et al. chose to combine SPI with expressed protein ligation (EPL).^194^ They expressed c-Crk-I SH2 as an intein fusion protein in E. coli and performed thiolysis on the purified fusion protein, which liberated SH2 with a C-terminal α-thioester. c-Crk-I SH3 on the other hand, was produced with 7AW in the Trp auxotrophic E. coli strain CY1507729 (Table 1). The expression construct was designed with an N-terminal hexahistidine tag for purification, which was set off from the SH3 sequence by a protease cleavage site. Proteolytic processing not only removed the tag but also generated an N-terminal Cys residue. EPL of SH2-α-thioester and Cys-SH3­[7AW] generated a chimeric c-Crk-I­(SH2-SH3­[7AW]) conjugate where SH2 and SH3­[7AW] were connected by a peptide bond (for mechanistic details of EPL, see ref 194). Trp169 and Trp170 of SH3 were replaced with 7AW at high efficiency (>93% at both positions) such that the spectral properties of the two SH domains of chimeric c-Crk-I­(SH2-SH3­[7AW]) could be exploited individually. c-Crk-I tolerated 7AW in the SH3 domain very well, the analog did not significantly change its stability and ligand binding. The thermodynamic properties of the isolated SH3­[7AW] domain (Table 2) and the c-Crk-I­(SH2-SH3­[7AW]) conjugate were indiscernible, which disfavored a direct interdomain interaction between SH2 and SH3.^89^

The metal-chelating Tyr analog Dopa (Figure 10) is a bidentate that coordinates metal ions *via* its two oxygen atoms. The Yun group exploited this property to engineer GFP with a metal binding site.^60^ They chose GFP-HS as the scaffold, whose sequence is identical to the extra superfolder green fluorescent protein, esGFP,^195^ which contains 8 Tyr residues. Their global replacement by Dopa red-shifted the fluorescence of GFP-HS­[Dopa] (Table 2) relative to its parent. Cu^2+^ ions quenched the fluorescence while other mono-, di- and trivalent metal ions had no effect. The copper ions neither shifted the excitation and emission maxima, nor did they affect the structure or conformation of GFP-HS. To study the mechanism of Cu^2+^ sensing further, they incorporated Dopa into two selected Tyr positions of GFP-HS by SCS using the DHPheRS/tRNA_CUA_^Tyr^ pair derived from *Mj*TyrRS/MjtRNA_CUA_^Tyr^ from Methanocaldococcus jannashii.^196,197^ However, the site-selective exchange of Tyr66 in the fluorophore and Tyr92 outside of the fluorophore by Dopa did not improve the Cu^2+^ sensitivity of the sensor protein.^60^ In a similar approach, Wu et al. turned GFPhs2 into an aluminum ion sensor by the quantitative replacement of its eight Tyr residues with Dopa using the Tyr auxotrophic E. coli strain JW2581 (Table 1).^111^ Binding of Al^3+^ ions but not of other metal ions enhanced the fluorescence of GFPhs2­[Dopa] (Table 2). The variant behaved as a selective and reversible Al^3+^ sensor not only as an isolated protein but also inside E. coli cells.^111^

Offenbacher et al. studied the proton transfer at the oxygen-evolving complex in photosystem II by introducing 7AW as a pH sensitive probe into the single Trp site of the subunit PsbO.^198^ Staudt et al. manipulated electron transfer processes in the photocycle of the riboflavin binding protein dodecin from Halobacterium salinarum by exchanging the single Trp36 residue with 4NW and 4AW (Figure 11). Trp36 plays a crucial role in quenching harmful side-reactions of light-excited riboflavin. It transfers an electron to the excited riboflavin, which generates a charge-separated intermediate state. The subsequent back-transfer of the electron to Trp36 relaxes the riboflavin to the ground state. An exchange of Trp36 by 4NW shortened the lifetime of the charge-separated state to one-fourth while 4AW slightly prolonged it (1.3 times). The electron back-transfer occurred 7-fold faster in dodecin­[4NW] relative to the parent. Despite the remarkable differences in their photocycle, the structures of the parent and variant proteins (Table 2) were highly isomorphous. This finding demonstrates that the different ionization potentials of 4NW and 4AW in place of Trp36 allowed the manipulation of the electron-transfer process with minimal structural perturbation.^141^

Very recently, Boknevitz et al. demonstrated the SPI of an azaborine-Trp analog (BNW) (Figure 11) in the E. coli Trp auxotroph ATCC 49980.^119^ Incorporation of BNW instead of Trp into two positions of ketosteroid isomerase (KSI) red-shifted the fluorescence emission maximum relative to the parent protein (KSI­[BNW], λ_ex max_ 372 nm vs KSI, λ_ex max_ 342 nm; Table 2).^119^

### 3.3 Tuning Folding, Stability, and Function with ncAAs

#### 3.3.1 Modification of Peptides with ncAAs

Ribosomally synthesized and post-translationally modified peptides (RiPPs) are an attractive class of antibacterial agents whose full therapeutic potential has not yet been unlocked. RiPPs are natural products of bacteria, but eukaryotic organisms can also produce them. RiPPs precursors are produced by ribosomal translation and the peptides mature by PTMs such as macrocyclization, dehydration and cyclodehydration, and [4 + 2] cycloaddition as well as lanthionine, sactionine, and lasso peptide formation. Tailoring the (therapeutic) properties of these fascinating compounds by expanding their chemistry with ncAAs appears very promising, particularly in the light of an acute and continued need for new antibiotics.^199–201^

In a joint effort, the groups of Budisa and Süssmuth demonstrated that it is possible to tune the antimicrobial properties of a lantibiotic by the residue-specific incorporation of noncanonical amino acids.^93^ They focused on the lantibiotic lichenicidin from Bacillus licheniformis, which consists of two components, Bliα (1 Met; 2 Pro) and Bliβ (2 Pro; 1 Trp). The single Met in Bliα was exchanged for Aha, Hpg, l-norleucine (Nle) and l-ethionine (Eth; Figure 7). The peptide congeners were purified and mixed in 1:1 molar ratio with the wild-type Bliβ peptide to test the antimicrobial activity of the resulting lichenicidin variants against the indicator strain Micrococcus luteus ATCC 9341. In comparison to the parent Bliα/Bliβ, Bliα­[Aha]/Bliβ was equally active, Bliα­[Nle]/Bliβ and Bliα­[Eth]/Bliβ were nearly as active and Bliα­[Hpg]/Bliβ was slightly less active. Furthermore, Bliα­[Hpg] was successfully conjugated to azido-fluorescein and 1-azido-1-deoxy-β-d-glucopyranoside using CuAAC. Incorporation of (2*S*,4*S*)-4-fluoroproline (*cis*-4-fluoro-l-proline, 4cFP), (2*S*,4*R*)-4-fluoroproline (*trans*-4-fluoro-l-proline, 4tFP), (2*S*,4*R*)-4-hydroxyproline (*trans*-4-hydroxy-l-proline, 4tHP), and (4*R*)-1,3-thiazolidine-4-carboxylic acid (Thz) (Figure 12) at both Pro positions in Bliα was successful, as was the replacement of the single Trp residue in Bliβ with 4FW, 5-hydroxy-l-tryptophan (5HW), and 7AW (Figure 11). The peptide congeners are listed in Table 2. The study by Oldach et al. first successfully demonstrated that RiPPs can be modified with ncAAs.^93^

**Figure 12:**
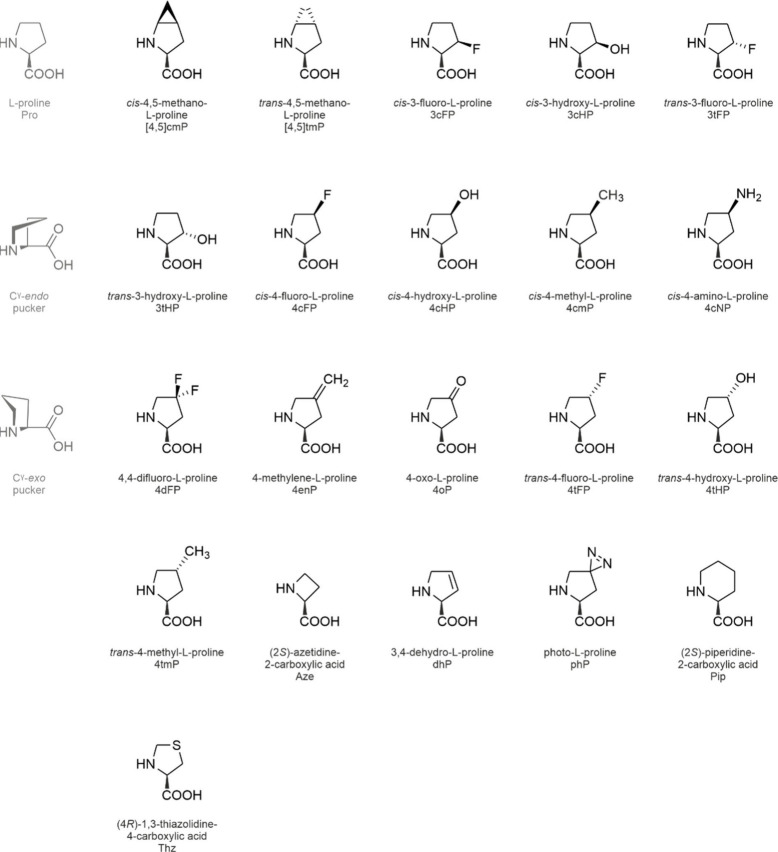
Structures of proline analogs. The structures are arranged in the alphabetical order of their acronyms.

In a follow-up study, Kuthning et al. used the evolutionarily adapted E. coli strain MT21­(DE3) (Table 1; see also Section 4.1.6), which converts the precursor 4*H*-thieno­[3,2-*b*]­pyrrole to [3,2]­Tpa (Figure 11),^202^ to incorporate this Trp analog into lichenicidin Bliβ. The titer of Bliβ­[[3,2]­Tpa] was approximately 2-fold lower than that of the parent protein, which was also lower than previously reported.^203^ Kuthning et al. attributed this finding to a reduced productivity of strain MT21­(DE3). Nevertheless, the antimicrobial activity of Bliα/Bliβ­[[3,2]­Tpa] was comparable to that of Bliα/Bliβ.^81^

The RiPP lantibiotic nisin is naturally produced by the lactic acid bacterium L. lactis. It has been used as a food-preservative because it effectively kills Gram-positive food spoilage bacteria.^204^ Nisin forms five lanthionine rings and the second ring contains a single Pro residue. Nickling et al. exchanged it against cFP, tFP, (2*S*,4*S*)-4-hydroxyproline (*cis*-4-hydroxy-l-proline, 4cHP), 4tHP, *cis*-4,5-methano-l-proline ([4,5]­cmP), and *trans*-4,5-methano-l-proline ([4,5]­tmP) (see the Pro analog structures in Figure 12) by SPI in the Pro auxotrophic E. coli strain MG1655 Δ*proBA*::frt Δ*proC*::frt (DE3) (Table 1).^90^ Nisin­[4tFP], nisin­[4tHP], and nisin­[4,5]­cmP inhibited the growth of a Gram-positive indicator strain to an equal extent as the parent protein.^90^ (Refer to Section 4.1.1.1 for the production of ncAA-labeled RiPPs in L. lactis.)

Lasso peptides adopt an unusual catenane-like structure: They form an N-terminal macrocyclic ring and tie a looplike knot by threading their C-terminal tail through the ring.^201^ Al Toma et al. genetically engineered the class II lasso peptide capistruin from Burkholderia thailandensis E264 to contain single Met residues in its “ring” (CapA^G4M^), “loop” (CapA^A10M^), and “tail” (CapA^G17M^) regions, which were then exchanged against Aha and Hpg. The titers of the Hpg variant proteins ranged from below the quantification limit to 7% of the parent, the Aha congeners reached 40% of the corresponding parent protein at maximum. A possible reason for the low titers of the Aha and Hpg variants could be the accidental incorporation of the ncAAs into T7RNAP (see the discussion of the matter in Section 5.3). As an alternative, reactive Lys derivatives were incorporated by SCS, which resulted in noticeably better protein titers, up to equal as the wild-type protein. The amount sufficed to modify one of the variants with an artificial PTM.^116^

Compstatin (comp) is a small, 13 amino acid therapeutic peptide that inhibits the complement cascade of the immune response.^205^ Trp at position 4 increases the complement inhibiting activity of comp. To assess the effect of Trp analogs on comp’s inhibitory activity, Katragadda and Lambris^79^ expressed the peptide as a self-splicing intein-fusion protein^206^ in the Trp auxotrophic E. coli strain ER2566/Trp82 (Table 1). They exchanged the two Trp residues with 6FW, 5HW and 7AW (Figure 11). The complement inhibitory activity of the variant proteins decreased with the hydrophilicity of the Trp analog. Comp­[6FW] was 3-fold more active than the parent peptide while the activity of comp­[5HW] was reduced by 28-fold and comp­[7AW] was not active at all.

#### 3.3.2 “Teflon” Proteins

Teflon is a perfluorinated polymer that favors fluorine-fluorine interactions but neither interacts with hydrophilic nor lipophilic molecules. This so-called “fluorous effect” makes Teflon an excellent material for nonstick cooking pans. The remarkable quality is absent in natural biopolymers, but it can be added to the structural and functional assets of peptides and proteins by polyfluorination. For example, the fluorous effect may enhance their stability and chemical robustness in biomaterial applications or improve the resilience of antimicrobial peptides against proteolytic degradation.^207,208^ The Budisa group intended to generate “teflon” proteins by incorporation of polyfluorinated Leu and Met analogs into model proteins of a variety of sizes, folds and structures.^209^ They chose anxA5 (35 Leu residues, 36 kDa), which contains predominantly α-helices; the β-sheet protein EGFP (15 Leu residues, 28 kDa); ψ-b* containing α-helices and β-sheets (15 Leu residues, 10 kDa); and recombinant human insulin-like growth factor binding protein (mini-IGFBP-5, residues Ala40-Ile92 including 7 Leu, 8 kDa) consisting of loops and β-sheets. Using the Leu auxotrophic E. coli strain DSM 1563 (Table 1) and the host LeuRS, they sought to exchange the Leu residues of the model proteins by 5,5,5-trifluoro-l-leucine (Tfl) (Figure 13). However, the protein titers of anxA5­[Tfl], EGFP­[Tfl] and ψ-b*­[Tfl] were extremely low (Table 2) and the incorporation efficiencies were marginal (2–7%). Only the Leu residues of mini-IGFBP-5 were exchanged somewhat better (up to 30% efficiency), but the protein titer was ∼100-fold lower than that of the parent protein. Budisa et al. speculated that the Leu→Tfl replacement occurred more efficiently in mini-IGFBP-5­[Tfl] (Table 2) because it was the smallest of the model proteins. The Leu content of the anxA5, EGFP, ψ-b* and mini-IGFBP-5 amino acid sequences ranged from 6% to 17%. To assess whether the inefficient Tfl incorporation originated from putative structural perturbations caused by the Leu→Tfl replacement at multiple positions, Budisa et al. turned to another model protein. They replaced the two Met residues of EGFP-2 M (mutant EGFP^M78L M88L M153T M233K^) by *S*-(trifluoromethyl)-l-homocysteine (l-trifluoromethionine, Tfm) (Figure 7) using the Met auxotrophic E. coli strain CAG18491 (Table 1). However, 10–15% incorporation efficiency in EGFP-2M­[Tfm] (Table 2) was again rather low. Based on their observations the Budisa group attributed the failure to incorporate Tfl and Tfm efficiently to the bulkiness of the ncAAs.^209^ Two years later, Panchenko et al. labeled the enzyme chloramphenicol acetyltransferase (CAT) with Tfl at 80% efficiency (see Section 3.3.5 below).^95^ They referred to Budisa’s 2004 study and hypothesized that a possible reason for their low incorporation efficiencies could have been that Budisa et al. employed the cAA limitation approach with cells which were not fully depleted of Leu and Met. In a follow-up study, the Budisa group took an alternative approach to generate “teflon proteins”. Merkel et al. pushed the limits of protein fluorination by replacing ∼10% of the amino acids of the thermophilic lipase TTL from Thermoanaerobacter thermohydrosulfuricus with their monofluorinated analogs.^134^ They substituted two Trp residues with 6FW, six Pro residues with 4cFP, and 16 Phe residues with 4-fluoro-l-phenylalanine (4FF) (Figure 9). The TTL­[4FF 4cFP 6FW] variant (Table 2) showed highly uniform fluorination at all substitution sites and its secondary structure remained unchanged. However, the multiple fluorination had a substantial impact on the titer, the temperature optimum and the maximum activity of the variant enzyme, which were 75%, 10 °C, and 40% lower in comparison to the parent protein.^134^ Potentially, the structure of TTL could have been engineered to accommodate multiple fluorine atoms without attenuating its performance as, for instance, the Tirrell group demonstrated for different target proteins (see Section 4.1.4).

Montclare et al. assessed the affect of the incorporation of the two Tfl diastereomers (2*S*,4*S*)-5,5,5-trifluoroleucine ((2*S*,4*S*)­Tfl) and (2*S*,4*R*)-5,5,5-trifluoroleucine ((2*S*,4*R*)­Tfl) (Figure 13) on the folding and stability of leucine zipper protein LzipA1 in the Leu auxotrophic E. coli strain LAM1000 (Table 1).^88^ They found that LzipA1­[(2*S*,4*S*)­Tfl)] was produced at a higher titer (18 mg/L) with a better incorporation efficiency of (2*S*,4*S*)­Tfl (91%) than LzipA1­[(2*S*,4*R*)­Tfl)] (9 mg/L, 80%). (2*S*,4*S*)­Tfl was slightly better activated by the *Ec*LeuRS than (2*S*,4*R*)­Tfl, which explained the differential titers and incorporation efficiencies. Both variants were produced at a lower titer than the parent (40% and 20%). The melting temperature of LzipA1­[(2*S*,4*S*)­Tfl)] and LzipA1­[(2*S*,4*R*)­Tfl)] homodimers (Table 2) was 10 °C higher than of the homodimeric parent protein containing Leu, which indicates a substantially increased thermostability of the fluorinated variants. The formation of heterodimers of the two diastereomeric variants increased the thermostability slightly further.^88^ The polyfluorinated Leu analogs improved the thermostability of LzipA1, yet Van Deventer et al. demonstrated that a non-fluorinated Leu analog with increased bulkiness had the same effect. They were able to elevate the melting temperature of LzipA1 by 17 °C relative to the Leu-parent by replacing its eight Leu residues with (2*S*,4*S*)-2-amino-4-methylhexanoic acid (l-homoisoleucine, Hil, Figure 13; LzipA1­[Hil], Table 2).^103^

Son et al. incorporated the fluorinated Val and Ile analogs (2*S*,3*R*)-4,4,4-trifluorovaline ((2S,3R)­Tfv) and (2*S*,3*S*)-5,5,5-trifluoroisoleucine (5tFI) (Figure 13) into basic leucine zipper (*bzip*) peptides derived from the yeast transcription factor GCN4.^97^ In the Ile/Val double auxotrophic E. coli strain AIV-IQ (Table 1), incorporation efficiencies of ∼90% were achieved with both analogs and the variant titers reached > 70% of the parent. The fluorination enhanced the stability of the *bzip*INL­[5tFI] and *bzip*VNL­[(2*S*,3*R*)­Tfv] variants (Table 2). Interestingly, 5tFI had a much more pronounced stabilizing effect than (2S,3R)­Tfv. This is an interesting finding because *bzip*INL and *bzip*VNL contained four Ile or Leu residues at the same positions in their sequences. The fluorination did not affect the DNA binding affinity and specificity of the variants.

#### 3.3.3 Proline Analogs: The Talented Amino Acids with an Edge

Fluorinated proline analogs appear to be particularly useful to manipulate the structure and stability of proteins. Among the cAAs, proline occupies a special place. It is the only cAA where the side chain at C^α^ bonds with the nitrogen of its amino group. The pyrrolidine ring forms a circular loop that directly links to the polypeptide backbone. Consequently, conformational changes in the ring structure reverberate in the polypeptide chain. The five-membered Pro ring preferentially assumes two conformations that are distinguished by the position of the C4 (i.e., C^γ^) atom relative to the average plane of the ring. If the ring adopts a C^γ^-*endo* pucker, the C4 atom protrudes from the ring toward the carboxyl group of Pro while in the C^γ^-*exo* pucker conformation, it protrudes in the opposite direction away from it (Figure 12). Fluorination of the pyrrolidine ring can “freeze” either of the two puckers, for instance, 4cFP is biased toward the C^γ^-*endo* pucker (relative equilibrium population 95%^210^) while 4tFP prefers the C^γ^-*exo* pucker (relative equilibrium population 86%^210^). Moreover, the fluorination of Pro can influence the *trans*/*cis* conformation of peptidyl-prolyl bonds,^211^ which has an impact on the folding and stability of proteins.^212,213^

The Conticello group performed a comprehensive study to assess the effects of Pro analogs on the properties of an elastin–mimetic polypeptide. The protein consisted of highly repetitive (VPGXG)_n_ domains and contained 80 Pro residues, which constituted 20% of the amino acid sequence. First, Kim et al. tackled the quantitative replacement of such a high number of Pro residues with a variety of Pro analogs such as: 4cFP, 4tFP, 4cHP, 4tHP, Thz, (2*S*)-azetidine-2-carboxylic acid (Aze), 3,4-dehydro-l-proline (dhP), 4,4-difluoro-l-proline (4dFP), and (2*S*)-piperidine-2-carboxylic acid (Pip) (the structures of all Pro analogs are shown in Figure 12).^34^ They observed that the host cell physiology strongly affected the incorporation efficiency of the Pro analogs. For optimal analog incorporation, they employed three different host strains in adapted incorporation procedures. The activity of native *Ec*ProRS of the Pro auxotrophic host DG99 (Table 1) was sufficient to incorporate 4cFP and 4tFP. Constitutive overexpression of *Ec*ProRS from a multicopy plasmid in the Pro auxotrophic strain CAG18515 (Table 1) in combination with hyperosmotic shock (600 mM NaCl) worked best to incorporate 4cHP, 4tHP, 4dFP and Aze. The hyperosmotic concentration of sodium chloride in the culture medium induced the up-regulation of the low-affinity proline transporters PutP, ProP, and ProU. The same setup but with constitutive overexpression of the E. coli ProRS C443G mutant (*Ec*ProRS^C443G^) facilitated the incorporation of Pip. To avoid intracellular oxidative degradation of dhP and Thz by the enzymes l-proline dehydrogenase and Δ^1^-pyrroline-5-carboxylate reductase, E. coli strain UMM5 with lesions in the corresponding *putA* and *proC* genes (Table 1) was used to incorporate them. Treatment of the cells with 800 mM sucrose was necessary to optimize the incorporation of Thz. The isolated variants had titers in the range of 15–50 mg/L (Table 2). Although Kim et al. did not analyze the properties of the elastin variants in detail, they observed retarded electrophoretic mobility of elastin­[4tHP] and elastin­[Aze] during sodium dodecylsulfate polyacrylamide gel electrophoresis (SDS-PAGE).

Next, they explored proline analogs to bias the assembly of elastin-like peptide mimics.^214,215^ Using the Pro auxotrophic strain DG99 (Table 1), they incorporated 4cFP and 4tFP^214^ as well as the corresponding C3 diastereomers (2*R*,3*R*)-3-fluoroproline (*cis*-3-fluoro-l-proline, 3cFP) and (2*R*,3*S*)-3-fluoroproline (*trans*-3-fluoro-l-proline, 3tFP) (Figure 12) into the elastin-mimetic polypeptide in shake flask batch cultures.^215^ The variant elastins were produced at titers comparable to the parent protein (Table 2). Type II β-turn formation is crucial for the self-assembly of elastin (see also Section 4.2.1). The structural conformation of the proline side chain ring plays an important role in guiding this process. The conformational bias of fluoroprolines is an excellent tool to dissect these effects. Indeed, Kim et al. observed that 4tFP and 3cFP, which preferentially adopt the C^γ^-*exo* pucker, stabilized the formation of type II β-turns and enhanced the self-assembly of elastin. In contrast, 4cFP and 3tFP prefer the C^γ^-*endo* pucker, thus they destabilized the formation of type II β-turns and impeded the self-assembly of elastin in comparison to Pro.^214,215^

Budisa and co-workers exploited fluoroproline diastereomeres to tune the stability of EGFP.^216^ The global replacement of its 10 Pro residues with 4cFP improved the folding efficiency and speed after thermal denaturation in comparison to the Pro parent while 4tFP drove the protein into insolubility. The comparison of the crystal structures of the EGFP parent and EGFP­[4cFP] (Table 2) revealed that the *cis*-fluorination at C4 preserved the preferred C^γ^-*endo* pyrrolidine ring pucker during thermal denaturation and subsequent refolding, which greatly accelerated and improved the folding process. Moreover, the fluorine atoms engaged in local interactions that substantially stabilized the EGFP­[4cFP] variant.^216^

Deepankumar et al. observed similar improvements of the red fluorescent protein mRFP1 when they exchanged its 12 Pro residues with 4cFP and 4tFP.^122^ In contrast to EGFP­[4cFP], mRFP1­[4cFP] was completely insoluble while the 4tFP variant was soluble but did not fluoresce. To tackle the nonfluorescence of the mRFP1­[4tFP] variant, the authors performed a computational analysis of the mRFP1 structure. It predicted that *trans*-4-fluorination of residue Pro63 would sterically interfere with the mRFP1 fluorophore. Deepankumar et al. generated an mRFP1^P63A^ mutant to prevent this interference and replaced the remaining eleven Pro residues with 4tFP. The P63A mutation slightly improved the protein titer, i.e., 26 mg/L mRFP1^P63A^ vs 25 mg/L mRFP1, and the incorporation of 4tFP raised it to 32 mg/L. The incorporation of 4tFP did not change the fluorescence properties of mRFP1^P63A^[4tFP] in comparison to its mRFP1^P63A^ parent, which experienced a blue-shift of λ_ex max_ by 28 nm and of λ_em max_ by 20 nm due to the P63A mutation. However, the thermal stability of the mRFP1^P63A^[4tFP] variant dramatically improved compared to the parent. It showed a 2–3 times higher stability in the temperature range between 25 and 60 °C. In a follow-up study, the group used the mRFP1^P63A^[4tFP] variant as a temperature sensor.^217^ mRFP1^P63A^[4tFP] was more stable toward chemical denaturation in 5% SDS (3-fold), 8 M urea (2.1-fold) and 6 M guanidinium chloride (1.6-fold) than the parent. As well, the incorporation of 4tFP accelerated its fluorophore maturation. Clearly, the combination of cAA mutagenesis to adapt the protein structure for ncAA incorporation (see Section 4.1.4 for further examples) and the global replacement of Pro with the 4tFP stereoisomer substantially improved the properties of mRFP1.

Opposing effects of fluoroproline stereoisomers were also reported for other proteins containing varying numbers of Pro residues. For instance, replacement of the single Pro residue of ψ-b* with 4cFP improved the refolding after chemical denaturation in urea while 4tFP and 4dFP (Figure 12) did not.^218^ The high-resolution X-ray structure of human ubiquitin shows all three Pro residues adopting a C^γ^-*exo* pucker. 4tFP, which has a bias for the C^γ^-*exo* pucker, improved the protein’s stability while 4cFP, which favors the C^γ^-*endo* pucker, could not be incorporated. With their findings, Crespo and Rubini confirmed the structural preorganization effect of fluoroproline stereoisomers.^121^ Edwardraja et al. replaced eight Pro residues in the humanized anti-c-Met single chain variable fragment (hu-MscFv) with 4cFP and 4tFP.^219^ The expression levels of hu-MscFv­[4cFP] and hu-MscFv­[4tFP] (Table 2) were comparable to the parent protein with Pro, nevertheless, 4tFP improved the folding of the protein while 4cFP did not. hu-MscFv­[4tFP] was more thermostable than the parent protein. At 40 °C, it retained 80% activity while the parent was only 50% as active as without heat treatment. In comparison to the parent, the half-life of the variant’s activity was 4-fold longer at the elevated temperature. Above 50 °C, both proteins were inactive. The authors explained the improved thermostability of hu-MscFv­[4tFP] with favorable local interactions of the fluorine atoms rather than the preorganization of the Pro ring puckers or the *cis*/*trans* isomerization of peptidyl-prolyl bonds. Using the Pro auxotrophic E. coli strain JM83 (Table 1), Marx and co-workers exchanged the 32 Pro residues of the thermophilic KlenTaq DNA polymerase with 4tFP.^76^ Despite the large size of the protein (540 amino acids, 63 kDa) and the high abundance of the Pro residues (6% of the amino acids), they achieved 92% labeling efficiency. LC-MS/MS analysis of the purified KlenTaq­[4tFP] variant revealed that at position Pro701 only Pro occurred while 20 Pro positions were only occupied by 4tFP and at 11 Pro positions, either 4tFP or Pro occurred. KlenTaq­[4tFP] (Table 2) retained the same activity, fidelity and sensitivity as the parent enzyme, however, it lost some thermostability.^76^ The crystal structure of KlenTaq­[4tFP] was very similar to the KlenTaq parent, yet the *trans*-fluorination at C4 of Pro generated a huge network of new noncovalent interactions. It is noteworthy, though, that the KlenTaq­[4tFP] crystallized better than the parent KlenTaq. The authors speculated that this was a result of the conformational homogeneity of the “frozen” 4tFP ring puckers in comparison to the more flexible Pro.^220^ As in most other cases outlined above, the incorporation of the other stereoisomer, 4cFP failed.^76^ The study by Marx and co-workers impressively demonstrated that the biophysical properties such as folding, stability, or crystallization behavior even of a large protein containing many Pro residues can be modulated with fluoroprolines. Nevertheless, the effects of the stereoisomers are difficult to predict because in addition to ring puckering, *cis*/*trans* isomerization of peptidyl-prolyl bonds, an altered structural interaction network as well as surface exposure of fluorine affect the overall biophysical properties of the protein.^76,220^

In addition to *cis*/*trans* fluorination of Pro at C4, methylation at this position can also affect protein properties in an opposing manner. Rubini and co-workers replaced the single Pro76 residue of the thiol/disulfide oxidoreductase thioredoxin mutant, Trx1P^139^ with the stereoisomers (2*S*,4*S*)-methyl-l-proline (*cis*-4-methyl-l-proline, 4cmP) and (2*S*,4*R*)-methyl-l-proline (*trans*-4-methyl-l-proline, 4tmP) (Figure 12) by co-overexpression of the *Ec*ProRS^C443G^ mutant in the E. coli Pro auxotroph CAG18515 (Table 1).^221^ While the incorporation of 4cmP failed, 4tmP could be introduced into Trx1P at 60% efficiency. The finding extends the preorganization effect to proline analogs with C4 substituents other than fluorine: The single Pro76 residue of Trx1P adopts a C^γ^-*endo* pucker. 4cmP and 4tmP prefer C^γ^-*exo* and C^γ^-*endo* puckers, respectively, hence, the successful incorporation of the latter into Trx1P.^221^ Rubini and co-workers generated the single Pro76 mutant of Trx1P to assess the predictability of a single residue exchange. However, contrary to other reports (see above), they observed that the exchange of Pro76 for 4cFP or 4tFP (Figure 12) had the *same* effect on Trx1P. Both stereoisomers stabilized the reduced form of Trx1P and destabilized the oxidized form whereas the disulfide reductase activity was not changed.^139^ While the introduction of 4cFP into Trx1P at Pro76 accelerated the folding kinetics of the protein,^222^ the incorporation of 4dFP at this position did not.^92^ Clearly, stereoismers such as 4cFP and 4tFP do not necessarily have opposing effects and even the outcomes of single cAA→ncAA exchanges are difficult to predict. Nevertheless, fluorinated Pro analogs are excellent molecular tools to modulate the stability and folding behavior of proteins.

Pharmaceutical insulin is a hexameric preparation that slowly dissociates into the pharmaceutically active monomer. The preparation delays the onset of insulin action and insulin fibrils can form during storage, which subdues the insulin’s pharmaceutical efficacy. To accelerate the dissociation without increasing the fibril formation, Tirrell and co-workers introduced hydroxyl groups at the C4-position of Pro28 in the B chain of insulin. They replaced the six Pro residues in pro-insulin (proIns) by SPI of 4cHP and 4tHP (Figure 12) in the Pro auxotrophic E. coli strain CAG18515 (Table 1) that coexpressed *Ec*ProRS from a multicopy plasmid.^82^ The proIns­[cHP] and proIns­[tHP] variants (Table 2) were produced at approximately 60% of the parent titer in shake flask cultures and Pro could be replaced with ∼90% efficiency. The variant proteins were refolded from inclusion bodies, processed into mature insulin by proteolytic treatment with trypsin and carboxypeptidase B and finally purified by HPLC. Mass analysis confirmed the correct proteolytic processing of the insulin. Mature insulin (Ins) contained only a single Pro residue in the B chain (ProB28), which was exchanged against 4cHP or 4tHP. The 3D structures of Ins­[Pro28B4cHP] (T2 dimer, PDB 5HQI; R6 hexamer, PDB 5HRQ) and Ins­[Pro28B4tHP] (T2 dimer, PDB 5HPR; R6 hexamer, PDB 5HPU) were solved and revealed no gross structure perturbations by the hydroxylation of ProB28. Ins­[Pro28B4cHP] and Ins­[Pro28B4tHP] were functional insulin variants as their subcutaneous injection reduced the blood glucose level of diabetic mice. As intended, the hydroxylation of Pro28B at C4 accelerated the dissociation of the insulin hexamer preparation and delayed the onset of insulin fibrillation.^82^

To identify beneficial properties of the Pro residue at position 28B with respect to hexamer dissociation and fibrillation in more detail, the Tirell group focused on Pro analogs with different ring size.^74^ They incorporated Aze, dhP, and Thz (Figure 12) using Pro-auxotrophic E. coli strains that overexpressed wild-type *Ec*ProRS, while the incorporation of Pip (Figure 12) required the overexpression of the *Ec*ProRS^C443G^ mutant. To prevent the degradation of dhP and Thz, strain KS32 (Table 1) was used. All four Pro analogs were incorporated at excellent efficiencies (89% to quantitative replacement) and approximately 40–60% of the parent titers were achieved. However, the unique effect of 4cHP incorporation at Pro28B of insulin with respect to accelerating hexamer dissociation while delaying fibrillation could not be topped by any of the ring-analogs of Pro. This finding supported the hypothesis that a new hydrogen bond of Pro28B4cHP across the insulin dimer interface was at least in part responsible for this desirable effect.

Next, Breunig et al.^66^ engineered insulin with a panel of aliphatic Pro analogs using the approach described above.^74,82^ They fused the N-terminal leader peptide H27R to pro-insulin to enhance its expression.^223^ Again, they equipped their Pro auxotrophic expression host E. coli CAG18515 (Table 1) with a multicopy plasmid expressing wild-type *Ec*ProRS for the incorporation of 4cmP (Figure 12); the *Ec*ProRS^C443G^ mutant to incorporate 4tmP (Figure 12); and the E. coli ProRS M157Q mutant (*Ec*ProRS^M157Q^) for the insertion of 4-methylene-l-proline (4enP) (Figure 12). To enhance the uptake of the Pro analogs, the cells were osmotically shocked with 0.5 M NaCl. The variant proteins proIns­[4tmP] and proIns­[4enP] (Table 2) were produced at titers of 83% and 65% of the parent, while proIns­[4cmP] (Table 2) accumulated to 150% of the parent protein. The incorporation efficiencies of the analogs ranged from 78% (4cmP) over 85% (4tmP) to 93% (4enP). Breunig et al. incorporated further aliphatic Pro analogs (Figure 12) into pro-insulin (variants listed in Table 2), albeit at comparably lower efficiencies than 4cmP, 4tmP, or 4enP: *cis*-3-hydroxy-l-proline (3cHP), 67%; *trans*-3-hydroxy-l-proline (3tHP), 54%; *cis*-4-amino-l-proline (4cNP), 17%; 4-oxo-l-proline (4oP), 15%; photo-l-proline (phP), 4%.^66^

The proIns­[4cmP], proIns­[4tmP] and proIns­[4enP] variants were proteolytically matured and HPLC purified as described above.^82^ The secondary structure of the mature insulin variants Ins­[Pro28B4cmP], Ins­[Pro28B4tmP], and Ins­[Pro28B4enP] was unaffected by the incorporation of the Pro analogs and all three reduced blood glucose levels in diabetic mice. The replacement of ProB28 with 4enP accelerated insulin fibril formation while the C4-methylation of ProB28 accelerated the dissociation of the insulin hexamer without driving fibril formation. Taken together, the works by Tirrell and co-workers impressively demonstrate how proline analogs with subtle molecular changes tune the therapeutically relevant properties of a protein drug.

#### 3.3.4 Further ncAAs to Tune the Stability and Folding of Proteins

Prion diseases are caused by the misfolding of the human cellular prion protein (hPrP^C^), by its aggregation and deposition into β-sheet enriched fibrils in the brain. Misfolded hPrP^C^ initiates the misfolding of other hPrP^C^ molecules in a self-propagating cascade.^224^ Budisa and co-workers intended to assess whether methionine oxidation might be one of the initial events that sets off the self-propagating protein conversion. To selectively arrest the recombinant hPrP^C^ either in the oxidized or the nonoxidized states, they decided to replace the nine Met residues in rhPrP^C^ with the analogs Nle and l-methoxinine (Mox, Figure 7). Mox is more hydrophilic than Met and mimics oxidized Met while Nle is hydrophobic and acts as a “non-oxidizable” Met analog. Both analogs were incorporated at very high efficiencies (Nle at 95% and Mox at 87%) into rhPrP^C^. Indeed, rhPrP^C^[Mox] and rhPrP^C^[Nle] (Table 2) behaved like pro- and antiaggregation variants of rhPrP^C^. rhPrP^C^[Mox] was β-sheet-rich and extremely aggregation-prone while rhPrP^C^[Nle] adopted a predominantly α-helical structure with a lower aggregation propensity than the rhPrP^C^ parent.^145^ This study impressivly demonstrates the utility of Met analogs in the study of protein structure and folding.

The small RNase barnase inhibitor ψ-b* contains three Trp residues of which two (Trp38 and Trp44) are fully or partially solvent exposed and Trp53 is entirely buried inside the hydrophobic core of the protein. To study the effect of ring-substituted Trp analogs on the stability of ψ-b*, Rubini et al. replaced all Trp residues with 4mW, 4NW and 5-amino-l-tryptophan (5NW) (Figure 11).^138^ The ψ-b*­[4NW] and ψ-b*­[5NW] variants were substantially less stable than the parent protein as indicated by their approximately 20 °C lower melting temperature. Both aminotryptophan variants were stable at 17–22 °C, below and above these temperatures the proteins were denatured. The amino substituents reduced the hydrophobicity of Trp, which destabilized the variants. In contrast, the incorporation of 4mW, which is more hydrophobic than Trp, slightly stabilized the b*­[4mW] variant (Table 2) in comparison to the parent protein.^138^

Lepthien et al. went a step further and tuned the physicochemical properties of ψ-b* by the simultaneous replacement of the single Met and Pro residues with Hpg (Figure 7) and 4cFP (Figure 12), respectively, as well as the three tryptophans with 4AW (Figure 11) in the Met, Pro, Trp-triple-auxotrophic E. coli strain JE5630 (Table 1). The resulting ψ-b*­[4cFP Hpg 4AW] variant (Table 2) displayed a combination of the effects of the individual ncAAs. The incorporation of 4AW conferred blue fluorescence, 4cFP increased the stability of the variant and Hpg provided a reactive handle for bioorthogonal conjugation. The effects of the ncAAs balanced each other. The replacement of three hydrophobic Trp residues with the relatively more hydrophilic 4AW substantially destabilized the Hpg/4AW double variant. The incorporation of 4cFP at the single Pro position outweighed the destabilization effect of 4AW and resulted in a highly stable Hpg/4AW/4cFP triple variant.^128^

#### 3.3.5 ncAAs to Redesign the Physicochemical Properties of Enzymes

The chemical diversification of the catalytic residues in the active center of enzymes is a particularly appealing application for ncAAs. SPI of ncAAs has been employed to introduce non-natural chemistry into the active center of enzymes although the site-specific installation by SCS might be preferable in this realm. Two recent reviews excellently compare the benefits of either incorporation technique for the engineering of enzymes with ncAAs.^225,226^ The interpretation of a coding sequence with different genetic codes by SPI can tune the stability, activity and selectivity of enzymes as the examples in this section demonstrate.

Walasek and Honek employed the Met analog *S*-(difluoromethyl)-l-homocysteine (l-difluoromethionine, Dfm) (Figure 7) as an ^19^F biophysical probe to study the alkaline protease AprA from Pseudomonas aeruginosa by ^19^F NMR spectroscopy.^107^ They produced AprA­[Dfm] (Table 2) in the Met auxotrophic E. coli strain B834­(DE3) at a titer of 40% of the parent enzyme. The mature enzyme contains a single Met residue in the active site, which was quantitatively replaced by Dfm. Dfm caused only minimal catalytic and structural alteration.^107^

Organophosphate hydrolase (OPH, aka phosphotriesterase, PTE; BRENDA:EC3.1.8.1) hydrolyzes organophosphorous compounds into phosphoric or alkylphosphonic acid derivatives, which lowers the pH. In an attempt to generate an acid tolerant enzyme, Votchitseva and colleagues exchanged the five Tyr residues of OPH with 3FY (Figure 10) using the Tyr auxotrophic E. coli strain B-2935 (VKM). While the titer of OPH­[3FY] (Table 2) was 10 times lower than that of the parent protein, the Tyr analog was nearly quantitatively (98%) incorporated. The pH optimum of OPH­[3FY] was extended to the acidic range and its thermal stability at alkaline pH was improved. However, OPH­[3FY] showed a lower catalytic performance. In comparison to the parent enzyme, *k*_cat_ decreased 40-fold and *K*_M_ increased 10-fold at the same time.^106^

Baker and Montclare exchanged the 15 Phe residues of the S5 phosphotriesterase from Brevundimonas diminuta (PTE) with 4FF (Figure 9).^63^ PTE is active as a dimer and seven out of its 15 Phe residues lie at the dimer interface. PTE­[4FF] (Table 2) was produced in the Phe auxotrophic E. coli strain AF-IQ (Table 1) at a rather low titer (10% of the parent protein) and the variant was largely insoluble. Nevertheless, Phe was exchanged at an excellent efficiency of ∼90%. While the parent enzyme hydrolyzed organophosphates as well as esters, the fluorination of the Phe residues improved the enzyme’s selectivity and hydrolytic activity on organophosphates but reduced them with esters. It also improved the variant’s thermostability as indicated by the fact that PTE­[4FF] retained substantially higher residual activity after heating > 45 °C than the parent enzyme.^63^

To polyfluorinate the chloramphenicol acetyltransferase (CAT; BRENDA:EC2.3.1.28), Panchenko et al.^95^ replaced its 13 Leu residues with Tfl (Figure 13) by SPI in the Leu auxotrophic E. coli strain LAM1000 (Table 1). Six of the 13 Leu residues form a hydrophobic core in the monomer, and they are not directly involved in substrate binding and catalysis. Tfl was incorporated with an efficiency of 80% and CAT­[Tfl] was produced at 32% of the parent protein titer. While the catalytic activity of CAT­[Tfl] (Table 2) was preserved, the variant enzyme’s thermostability decreased substantially. The parent retained more than 80% of its activity after heating to 60 °C, but CAT­[Tfl] showed only 20% residual activity. Compared to the parent protein, CAT­[Tfl] was more sensitive toward solvents such as DMSO, ethanol and trifluoroethanol as well as against denaturants, e.g., urea. To dissect, which Leu→Tfl replacements contributed to the instability of the variant enzyme, Voloshchuk et al. generated 13 mutants where a single Leu each was mutated to Ile while the other Leu residues remained unchanged.^227^ Again, Tfl was incorporated into all single Leu→Ile mutants by SPI as in the earlier study and the thermostabilities of the CAT^LnI^[Tfl] variants (where n indicates the position of the mutation) were compared to CAT­[Tfl]. Variant CAT^L158I^[Tfl] was approximately 2-fold more thermostable than CAT­[Tfl] while the thermostabilities of CAT^L158I^ and the wild-type CAT were indiscriminate. Tfl was incorporated into CAT^L158I^ and CAT with the same efficiency (82% and 83%, respectively). From these observations, the authors concluded that Tfl at position Leu158 had the most pronounced impact on the thermostability of CAT­[Tfl].

**Figure 13:**
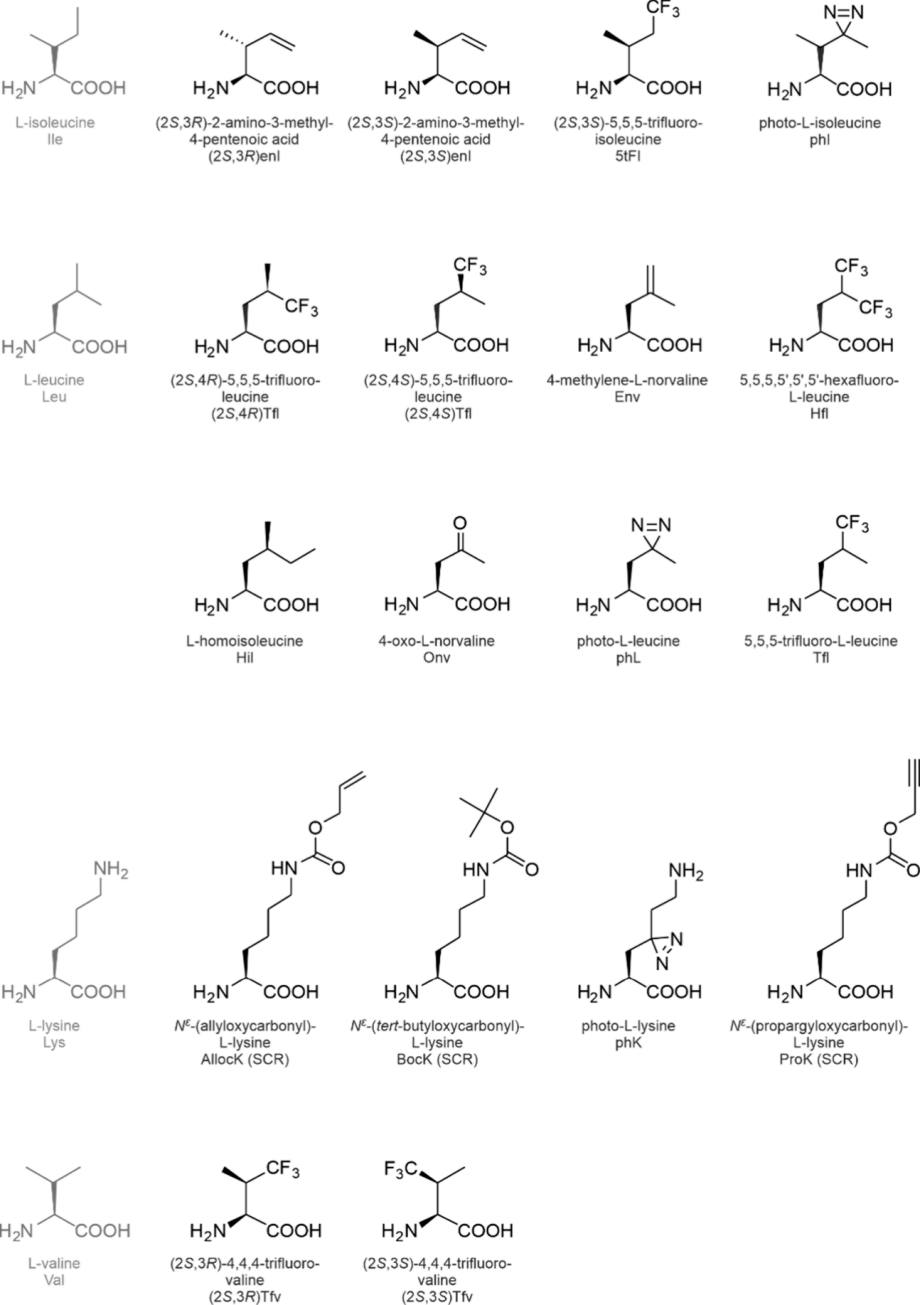
Structures of isoleucine, leucine, lysine, and valine analogs. The structures are arranged in the alphabetical order of their acronyms. SCR, sense codon reassignment with an orthogonal ncAARS/tRNA pair.

Next, Montclare and co-workers explored whether and how the position of a fluorine substituent in Phe could influence enzyme properties. They first tackled the catalytic activity of the histone acetyltransferase (BRENDA:EC2.3.1.48) tGCN5 from Tetrahymena thermophila.^105^ tGCN5 transfers the acetyl group from acetyl-coenzyme A (acetyl-CoA) onto Lys14 of histone H3. Out of 10 Phe residues in total, all except one are buried in the core of tGCN5. Voloshchuk et al. globally replaced them with 2-fluoro-l-phenylalanine (2FF), 3-fluoro-l-phenylalanine (3FF) and 4FF (Figure 9). The variants tGCN5­[2FF] and tGCN5­[4FF] reached approximately half the titer of the parent enzyme and tGCN5­[3FF] (Table 2) was produced at the same level by the Phe auxotrophic E. coli strain AF-IQ (Table 1). The incorporation of all three analogs occurred with an efficiency of ∼85%. The fluorination of Phe destabilized all variants. The destabilization effect was most pronounced for tGCN5­[2FF], whose melting temperature *T*_m_ was 5 °C lower than the parent’s, followed by tGCN5­[3FF] (Δ*T*_m_ −2.5 °C) and finally tGCN5­[4FF] (Δ*T*_m_ −2 °C). Obviously, the steric repulsion increased in the tightly packed protein core when the fluorine substituent on the Phe ring was close to the Cα. All fluorophenylalanine variants showed increased susceptibility to proteolytic digestion with chymotrypsin, which cleaves after Phe.^228^ Again, the effect was least severe for tGCN5­[4FF]. The catalytic activities of tGCN5­[3FF], tGCN5­[4FF] and tGCN5­[2FF] decreased 3-, 6- and 16-fold in comparison to the parent.

In a second study, Mehta et al. focused on tuning the substrate selectivity of a histone acetyltransferase with fluorophenylalanine regioisomers.^86^ p300/CBP associated factor (PCAF) transfers an acetyl group from acetyl-CoA to Lys on its histone substrate H3 or on the nonhistone substrate p53. PCAF contains a total of 10 Phe residues, which occur all outside of its active site. 2FF, 3FF and 4FF were incorporated as described for tGCN5 above. The titers of PCAF­[2FF], PCAF­[3FF], and PCAF­[4FF] (Table 2) were rather low (15%, 27%, and 25% of the parent titer) while the incorporation efficiency reached 73%, 88%, and 81%. PCAF­[2FF] was completely inactive. PCAF­[3FF] specifically acetylated the histone H3 substrate but had no detectable activity with the nonhistone substrate. PCAF­[4FF] acetylated the nonhistone substrate to a higher extent than the parent enzyme, which had a > 250-fold preference for the histone H3. 2FF completely disrupted the structure of PCAF, 4FF caused some structural perturbance and 3FF had only a marginal influence. Clearly, the structural impact of the fluorophenylalanine regioisomers was related to the substrate selectivity: 3FF hardly affected the structure and PCAF­[3FF] acetylated only the histone H3, which reflected the parent’s substrate preference. 4FF opened the structure such that the PCAF­[4FF] variant enzyme was able to accept the alternative nonhistone substrate at the cost of stability and selectivity for the native histone substrate. 2FF unstructured PCAF too much, which resulted in the complete functional loss of PCAF­[2FF].^86^ Taken together, the studies of the Montclare group highlight the impact of subtle positional changes of a single fluorine atom on enzyme structure and function.^86,105^

The Budisa group engineered the thermophilic lipase TTL with 10 different ncAAs.^115,125^ They exchanged the 11 Met residues of the enzyme with Aha and Nle (Figure 7), 6 Pro residues with 4cFP, 4tFP, 4cHP, and 4tHP (Figure 12), 16 Phe residues with 3FF and 4FF (Figure 9), and 7 Tyr residues with 2FY and 3FY (Figure 10) using a variety of properly auxotrophic E. coli strains (CAG18491 (Met auxotroph), CAG18515 (Pro auxotroph), DG30 (Phe auxotroph), and AT2471 (Tyr auxotroph) (for relevant genotypes, see Table 1). The TTL protein matrix accommodated all ncAAs except the Tyr analogs extremely well (all variants are listed in Table 2). TTL­[2FY] and TTL­[3FY] were produced at only 30% and 65% of the parent titer. The TTL variants containing Aha, Nle, 4cHP, and 4tHP analogs occurred at the same level as the parent enzyme. Surprisingly, 4cFP and 4tFP improved the titer of the corresponding variant proteins to 115% and 145% and the variants containing 3FF and 4FF accumulated at a 2.5- and >3-fold higher level than the parent enzyme. The incorporation efficiencies of Aha, Nle, 4cFP, 2FY, and 3FY were good to very good (>50% fully labeled variant), but only partially labeled protein species (<50% fully labeled variant) were found with 4tFP, 4cHP, 4tHP, 3FF, and 4FF. The different ncAAs tuned TLL’s properties in various ways. Originating from a thermophilic organism, TLL requires heat activation for full activity. Nle had the most striking effect as the TTL­[Nle] variant was >10-fold more active than the parent enzyme without heat activation. Obviously, the exchange of 11 Met residues for the more hydrophobic analog Nle increased the overall hydrophobicity of the protein. This became evident by the accelerated migration behavior of the variant on an SDS gel. Recently, Haernvall et al. exploited this trait of TTL­[Nle] to demonstrate that is a potent polyesterase.^229^ In contrast, TTL­[Aha] showed little activity without heat activation, which reflected the parent enzyme’s behavior. TTL­[Nle], TTL­[mFF], and TTL­[oFY] retained the optimal temperature *T*_opt_ of 75 °C of the parent enzyme. All other variants showed lower *T*_opt_, for TTL­[3FY] and TTL­[4FF] it was only at 50 °C, i.e., 25 °C lower than for the parent, which hinted at destabilization of the enzyme. The pH optima of the variant enzymes ranged from pH 7–9, which differed not much from pH 8 of the parent. Again, the position of the fluorine atom in the fluorophenylalanine and fluorotyrosine containing variants caused stunning differential behavior. Thile *T*_opt_ of TTL­[3FY] and TTL­[4FF] was ∼50 °C, TTL­[2FY] and TTL­[3FF] had their temperature optima at ∼70 °C. The activity of heat-activated TTL­[3FF] exceeded that of the parent TTL by ∼25%. In contrast, TTL­[4FF] was not half as active as the parent after heat activation. 3FY inactivated the enzyme while heat-activated TTL­[2FY] was fully active. TTL­[3FF] showed a broader substrate tolerance (C2-C18, optimum C8 > C6) than the parent protein (C8 > C6 ≫ C10). It accepted shorter as well as longer acyl chains and thus combined esterase with lipase behavior. To expand the collection of TLL variants, Acevedo-Rocha et al. exchanged the two Trp residues of TTL with 7AW, 4FW, and 4NW (for structures, see Figure 11; variants are listed in Table 2).^115^ Similar to the other ncAAs, TLL tolerated the Trp analogs very well, and the corresponding variants were obtained at 90%, 185%, and 210% of the parent titer. The authors assessed the tolerance of the various TTL variants toward organic solvents, metal ions, reducing-, alkylating- and denaturing agents as well as inhibitors. DMSO and toluol killed the activity of all TTL enzymes, parent as well as variants. All variants except TTL­[Aha] were more active in *tert*-butanol than the parent, particularly TTL­[4cFP] and TTL­[4tFP] showed a 4-fold improved activity. In general, the variants containing Pro analogs tolerated most solvents very well and Pro fluorination pronounced the effect more than hydroxylation. Trivalent and divalent cations but not K^+^ and Na^+^ impaired the activity of all enzymes. The surfactant CHAPS improved the activity of all variants including the parent, yet TTL­[4tHP] was nearly 10-fold more active than the parent protein followed by TTL­[3FY] (∼7-fold) and TTL­[4cHP] (∼6-fold). While 2-mercaptoethanol and dithiothreitol (DTT) inhibited the parent enzyme, most variants either were not affected or their activity was even slightly improved in DTT, e.g., up to 140% for TTL­[4tHP], 130% for TTL­[7AW] and TTL­[Nle], 110% for TTL­[4NW] and TTL­[4FF], and up to 120% for TTL­[7AW] and TTL­[4tFP]. The alkylating agent 2-iodoacetate reduced the activity of parent to 80%, the variants were either unaffected or up to 1.6-fold more active than without 2-iodoacetate. Parent TTL lost all activity after treatment with guanidinium hydrochloride but TTL­[4NW] and TTL­[3FF] retained 70% of their activity. Urea increased the activity of TTL­[7AW] 1.9-fold while the parent was only 0.9-fold as active as without urea. Finally, some variants even resisted the inhibition by Pefabloc, which efficiently inhibited the parent TTL. TTL­[4cFP] and TTL­[3FY] retained 40% of their activity, while Pefabloc completely inhibited the activity of TTL­[4tFP] and TTL­[2FY].^115^ The comprehensive studies by the Budisa group highlight the power of enzyme engineering with ncAAs. They impressively demonstrate how the interpretation of an enzyme’s genetic information with alternative genetic codes lets us tune its various traits. However, they also poignantly accentuate our current inability to explain most of the effects let alone predict them.

The next example irritatingly exposes this inability. The enzyme 4-oxalocrotonate tautomerase (4-OT, BRENDA:EC5.3.2.6) catalyzes the asymmetric Michael-type addition of various aldehydes to nitrostyrene. It contains two Pro residues, of which the N-terminal catalytic Pro cannot be replaced by classic mutagenesis without destroying the enzyme’s activity. To tune the activity of 4-OT, Lukesch et al. replaced both Pro residues with dhP, 4cFP, 4tFP, and Thz (Figure 12).^129^ The authors co-overexpressed ProRS and MetAP from E. coli to achieve quantitative replacement of both Pro residues with their analogs as well as quantitative excision of the N-terminal Met to expose the catalytic Pro residue. However, all 4-OT variants were inactive, none showed Michael-type activity. Likewise, variants of a 4-OT^P34E^ mutant, in which exclusively the single N-terminal catalytic Pro was exchanged by the Pro analogs were inactive. In comparison, the 4-OT^P34E^ mutant showed Michael-type activity similar to 4-OT. The incorporation of the Pro analogs did not perturb 4-OT’s structure and the NMR structure of 4-OT­[dhP] was virtually unchanged in comparison to parent protein. The most likely explanation for the inactivating effect of the Pro analogs was that they caused changes in the fast dynamics of the 4-OT active site that led to the loss of its Michael-type activity.^129^

The Arg analog l-canavanine (Can) (Figure 14) is a toxic amino acid for humans and many animals^230^ as well as for E. coli cells low on intracellular Arg levels.^231^ Worst et al. incorporated Can into EGFP in the Arg auxotrophic E. coli strain JE7094 (Table 1).^146^ All seven Arg residues were quantitatively replaced by Can, but the titer of EGFP­[Can] (Table 2) was very low. An alternative cell-free protein expression system delivered 2.5 times as much, which clearly highlights the toxicity of the Arg analog. To evade or at least reduce the cytotoxicity of Can and improve its SPI in E. coli, Inouye and co-workers used their single protein production process. When the Arg auxotrophic E. coli strain BL21­(DE3) (Δ*argH*Δ*trpC*Δ*hisB*) reached the midlog phase of growth, they induced the expression of the *mazF* gene encoding the endoribonuclease MazF from E. coli. *Ec*MazF cleaves RNA at ACA motifs, i.e., any cellular RNA containing the recognition motif will be cleaved as soon as the *mazF* gene is induced. Apparently, tRNAs and rRNAs can evade the action of EcMazF because of their elaborate secondary structure and their association with ribosomal proteins, respectively. Without RNAs, E. coli cannot grow yet is metabolically fully active.^232,233^ To exploit this “dormant state” of the host for SPI of Can into their target protein, the endoribonuclease MazF-bs from Bacillus subtilis, Inouye and co-workers designed an ACA-free *mazF*-bs mutant gene. Its expression was induced after the host RNAs had been greatly diminished by the action of the *Ec*MazF. Since the target mRNA was ACA-free and thus immune against the endoribonuclease activity of *Ec*MazF, the resources of the host could be effectively channeled into the production of MazF-bs­[Can] (Table 2). Indeed, seven Arg residues were exchanged against Can at an efficiency of 97%. Moreover, MazF-bs­[Can] was specific for a recognition motif that contained an additional 3′-terminal A (U#ACAUA) and did not cleave the U#ACAU motif of the parent MazF-bs containing Arg.^77^

**Figure 14:**
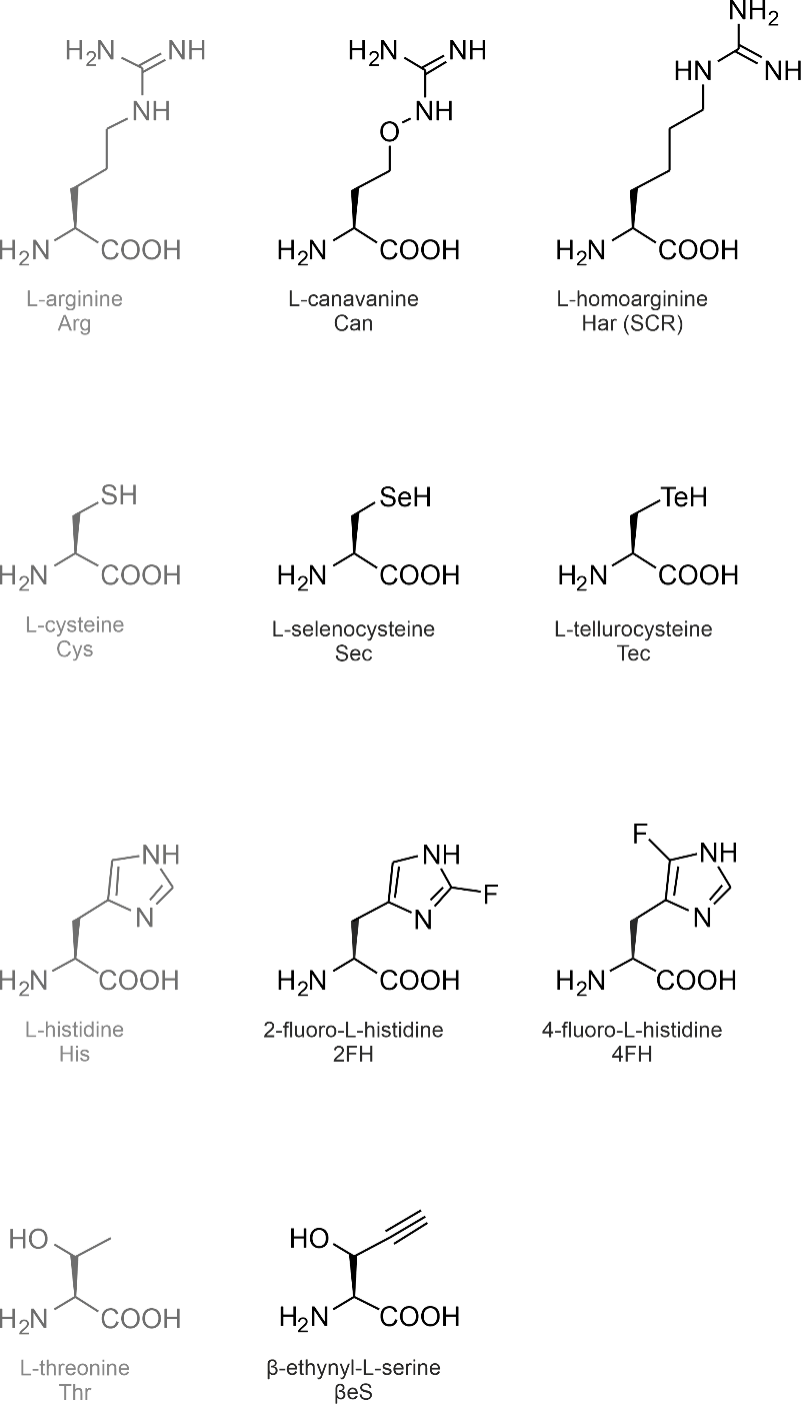
Structures of arginine, cysteine, histidine, and threonine analogs. The structures are arranged in the alphabetical order of their acronyms. SCR, sense codon reassignment with an orthogonal ncAARS/tRNA pair.

Enzyme engineering with ncAA is particularly attractive to design new catalytic function. Shen and co-workers explored chalcogen ncAAs^234^ for this purpose. Glutathione peroxidase (GPx, BRENDA:EC1.11.1.9) is a selenoenzyme that catalyzes the reduction of hydroperoxides. GPx contains a catalytically active Sec (Figure 14) residue and uses glutathione (GSH) as the reducing cosubstrate. Despite the fact that selenoproteins occur in all domains of life,^2^ their recombinant production is complicated because Sec is not a cAA. Noncanonical Sec is encoded by the opal (UGA) stop codon and its insertion at a specific UGA requires a unique sequence element. Frequently, the element’s sequence constraints hamper its accommodation in the coding sequence of a recombinant protein.^3^ Glutathione S-transferase (GST, BRENDA:EC2.5.1.18) resembles glutathione peroxidase with respect to the glutathione binding domain and the catalytic properties and can serve as a scaffold for the introduction of GPx function. To generate a GPx mimic, Yu et al. changed the catalytic Ser9 residue of Lucilia cuprina glutathione transferase (LuGST1-1) to Cys and introduced Cys→Ser mutations at two other positions.^236^ The resulting LuGST1-1^S9C C86S C200S^ mutant (LuGST1-1^C^) contained a single Cys residue in the active site, which allowed them to study exclusively the effect of Sec incorporation on the catalytic activity. Sec was incorporated with 70% efficiency at the single active site Cys by SPI in the Cys auxotrophic E. coli strain BL21­(DE3)*cysE51* (Table 1). Indeed, LuGST1-1^C^[Sec] (Table 2) reduced H_2_O_2_ with GSH and its activity reached 50% of native GPx from rabbit liver.

To enhance the activity of the GPx mimic further, Liu et al. replaced Sec by l-tellurocysteine (Tec) (Figure 14) in LuGST1-1^C^. Tec has a lower redox potential than Sec and is therefore more reducing.^235^ First, the authors confirmed that Tec is a substrate for E. coli cysteinyl-tRNA synthetase. However, it aminoacylated tRNA^Cys^ with Tec substantially less efficiently than with Cys. They employed their earlier described SPI approach^236^ but Tec was highly toxic for the E. coli cells. After the successful adaptation of the SPI conditions, Tec could be incorporated quantitatively at the single Cys of LuGST1-1^C^. As expected, the LuGST1-1^C^[Tec] variant (Table 2) showed a higher activity than LuGST1-1^C^[Sec]. In fact, LuGST1-1^C^[Tec] rivaled the glutathione peroxidase activity of natural GPx.^235^

The Shen group extended their enzyme design by noncanonical chalcogens to the glutaredoxin (Grx) domain of mouse thioredoxin-glutathione reductase.^237^ They engineered the Grx^C105S^ mutant that contained a single Cys48 in the active site (Grx^C^) and incorporated Sec by SPI as reported before.^235,236,238^ Grx^C^[Sec] (Table 2) showed the same peroxidase activity as LuGST1-1^C^[Sec] (see above). The three studies by the Shen group successfully used Grx and GST as a scaffold to generate GPx activity, which strongly suggests that Grx, GST, and GPx originate from the same ancestor. Moreover, they demonstrated that Sec and Tec can complement Cys in generating enzymatic redox activity.

#### 3.3.6 β-Cyclopropyl-l-alanine, a Met Analog with a Cyclic Side Chain

β-Cyclopropyl-l-alanine (Cpa) (Figure 7) is a Met analog containing a cyclopropyl ring in the side chain. In an earlier study, Szostak and co-workers had observed the incorporation of Cpa at internal AUG codons (ATG on DNA) but rather not at the N-terminus in a cell-free expression system.^37^ To test this hypothesis *in vivo*, Acevedo-Rocha et al. assessed the incorporation of Cpa into several different target proteins in the Met auxotrophic E. coli strain CAG18491 (Table 1).^114^ The target proteins TTL (11 Met residues, penultimate residue Q), anxA5 (penultimate residue A), anxA5^A2G^ and anxA5^A2R^ (8 Met residues each) as well as an EGFP^G2R^ mutant (6 Met residues) did not tolerate Cpa and were not produced in the presence of this ncAA. In contrast, five out of six positions of EGFP­[Cpa] (penultimate G) and EGFP^G2A^[Cpa] were labeled with an efficiency of ∼55%, other variants contained four and three Cpa residues. The potential excision of the N-terminal Cpa by the E. coli MetAP might be the reason why fully labeled variants were not observed. ψ-b*, which contains a single N-terminal Met followed by two Lys residues that disfavor N-terminal Met excision by the E. coli MetAP^173^ was expressed with Cpa as was the corresponding ψ-b*4 M mutant containing three additional internal Met positions. Cpa was incorporated with ∼75% efficiency at the single N-terminal position of ψ-b*­[Cpa]. The labeling efficiency of all four positions in ψ-b*4M­[Cpa] was 70% although also partially labeled variants were observed. The *in vivo* study clearly confirmed the incorporation of Cpa not only at internal AUG codons as had been shown earlier *in vitro*, but also at the N-terminus of recombinant proteins. As well, Cpa might be cleaved by the E. coli MetAP.^114^

#### 3.3.7 His Analogs as pH Sensors

Eichler et al. incorporated 2-fluoro-l-histidine (2FH) and 4-fluoro-l-histidine (4FH) into the single His mutant chaperone PapD^R200H^ (Table 2).^72^ While the single His was replaced by 2FH nearly quantitatively, 4FH was incorporated at approximately 50%. The lower incorporation efficiency of the latter may reflect its relatively lower supplementation in the medium due to limited availability. 2FH and 4FH are suitable probes to study the involvement of His in pH dependent processes because their side chain p*K*_a_ is several units lower than that of His.^239^

### 3.4 Application of Seleno and Fluoro Amino Acids for Analytics

The first global exchange of Met by its analog l-selenomethionine (Sem) (Figure 7) in a Met auxotrophic E. coli strain was reported nearly 70 years ago.^240^ The global Met→Sem exchange has become a routine to solve the phase problem in protein crystallography by multiwavelength anomalous diffraction (MAD) since this application was first described by Hendrickson et al. in 1994.^241^ Although nonauxotrophic hosts have been used to incorporate Sem for MAD, which usually results in incomplete labeling, Met auxotrophic expression hosts are particularly useful for quantitative, high efficiency labeling of proteins. Labeling strategies have been devised for E. coli, the yeasts S. cerevisiae and P. pastoris as well as mammalian cells.^242^ Oxidation of Sem enhances the anomalous signal, which is an option for proteins with a low number of Met residues.^243^ Strub et al. introduced Sem and Sec at the same time into two targe proteins in a Cys auxotrophic strain which was inhibited for Met biosynthesis to enhance the anomalous signal.^244^ l-Telluromethionine (Tem) (Figure 7) represents another excellent alternative to Sem and Sec to intensify the anomalous signal. However, the compound is extremely toxic and prone to oxidation, which complicates its incorporation using a Met-auxotrophic expression host.^245^ Another Te analog, the Phe surrogate l-2-tellurienylalanine (TeF) (Figure 9), might be a better option in the future because it is much less toxic than Tem and is very efficiently incorporated into target proteins even in nonauxotrophic hosts (see also Section 4.3.1). Moreover, Phe occurs more abundantly in proteins than Met.^246^

Fluorine is a monoisotopic element with ^19^F being the single naturally occurring stable isotope.^247^ Fluorine is extremely rare in biological samples, which causes virtually no background interference in the application of ^19^F for imaging or spectroscopy.^248,249^ In comparison to ncAAs labeled with other isotopes, many fluoro-ncAAs are commercially available at a reasonable price or can be accessed *via* biosynthesis (see Sections 5.2.4.1 and 5.2.5.1). For these reasons, fluorinated ncAAs are valuable tools for protein ^19^F NMR. Auxotrophic expression hosts for ^19^F-labeled proteins are not absolutely necessary because alternative isotope labeling methods exist.^248^ Nevertheless, Welte et al. demonstrated that the Phe and Trp auxotrophic E. coli hosts NK6024 and CAG18455 (Table 1) facilitated the efficient and controlled fluorination of the Phe and Trp residues of a cold shock protein for ^19^F NMR. In contrast, the standard method to provoke the cellular uptake and incorporation of fluorinated aromatic amino acids by the inhibition of the aromatic amino acid biosynthesis pathway with glyphosate was unsuccessful.^250^ The residue-specific incorporation of 2FF, 3FF, and 4FF (Figure 9) as well as 4FW, 5FW, and 6FW (Figure 11) did not change the target protein’s thermodynamic and structural properties.^251^ Such observations cannot be generalized, though. Acchione and colleagues^252^ globally exchanged all six Trp residues of an scFv with 5FW and performed ^19^F NMR spectroscopy to characterize its antigen binding. In addition, they prepared single Phe mutants at each of the six positions and labeled the remaining five with 5FW to decipher the contribution of fluorine at each individual position. Although the incorporation of 5FW did not cause gross structural changes, the fluorination of selected Trp positions distinctly affected the antigen-binding.^252^

## 4 Developments beyond the Classics

New developments to meet special challenges of genetic code engineering advance the field beyond the classics. NcAAs were successfully incorporated into demanding targets such as secreted proteins and a transmembrane channel. The overexpression of wild-type and engineered aminoacyl-tRNA synthetases (mutAARSs) can improve the incorporation of more distant analogs of the canonical amino acids. The structure of target proteins can be engineered to accommodate such ncAAs that otherwise interfere with folding and/or stability. Breaking the degeneracy of the genetic code unlocks the reassignment of sense codons to ncAAs. Site- (SCS) and residue-specific incorporation (SPI) of two different ncAAs in parallel allow precisely tailored protein modifications. Biomaterials such as elastin-like proteins or silks can be functionalized for non-natural purposes such as drug release. The genetic encoding of post-translational modifications provides access to biomaterials of unprecedented performance. Organisms depending on certain ncAAs by adapted laboratory evolution (ALE) shed light on the reasons why nature uses only a small subset of chemistries for its biological building blocks and how to surmount these limitations.

### 4.1 Supplementation-Based Incorporation Approaches to Meet Special Challenges

#### 4.1.1 Incorporation of ncAAs into Demanding Proteins

E. coli has been the host of choice for the residue-specific incorporation of ncAAs (see Section 3). However, it performs inadequately at expressing membrane proteins, proteins that require post-translational modifications and protein secretion. To overcome these limitations, SPI experiments have been reported for alternative hosts such as the Gram-positive bacterium L. lactis and the yeasts S. cerevisiae and K. phaffii (aka P. pastoris) as well as mammalian cells and even an insect (Figure 15). Eukaryotic hosts are an attractive alternative to E. coli for the recombinant production of large and/or post-translationally modified proteins. Notoriously, K. phaffii secretes proteins into the growth medium with high efficiency.^253^ Noncanonical amino acid tagging has gained enormous momentum for proteomics studies in mammalian cells, and we treat this topic in Section 4.3.1. The residue-specific labeling of mulberry silk in the silkworm Bombyx mori is the subject of Section 4.2.2.

**Figure 15:**
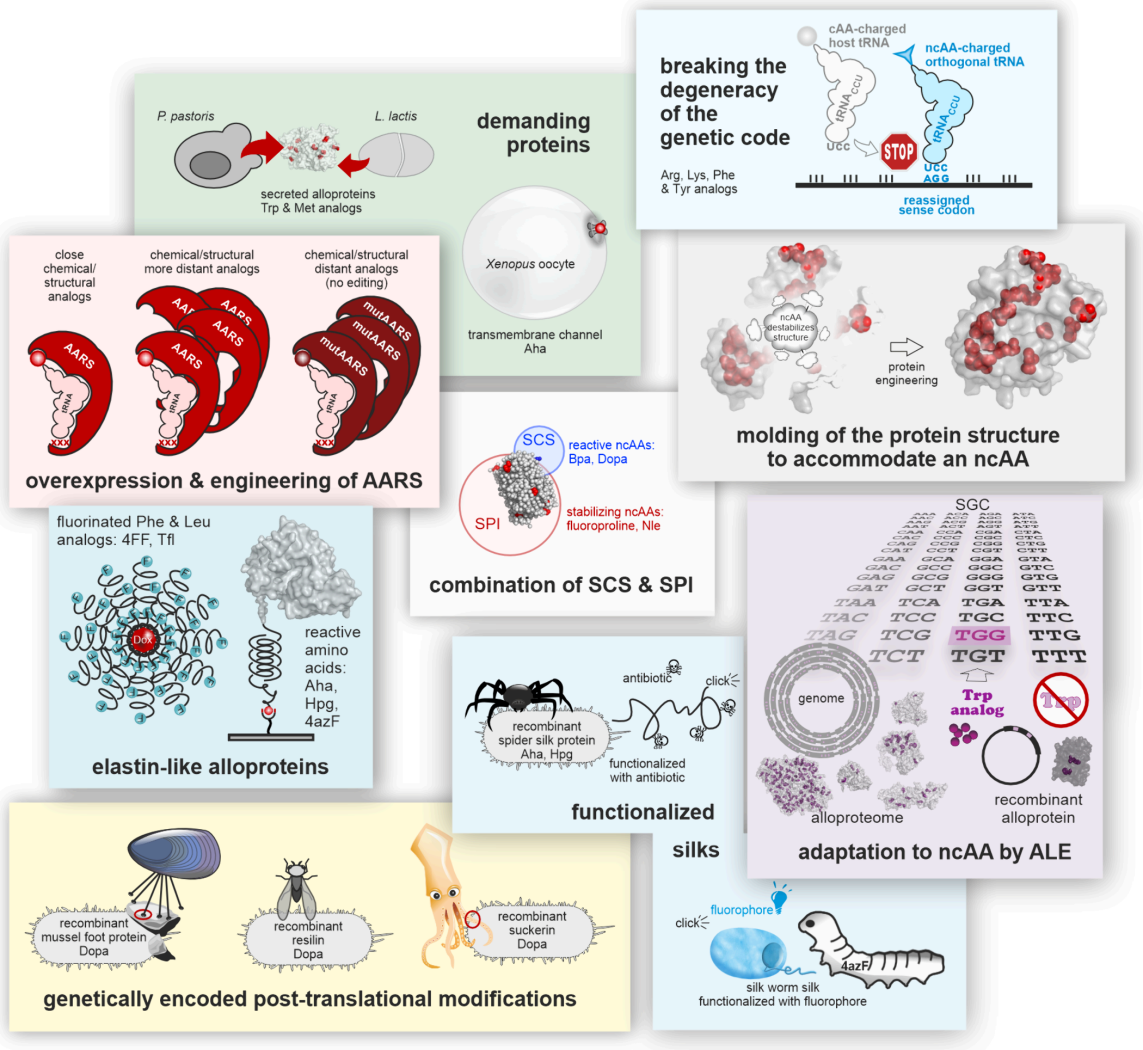
New developments to meet special challenges of genetic code engineering.

##### 4.1.1.1 Secreted Proteins

Lactococcus lactis is a Gram-positive lactic acid bacterium commonly used for the fermentation of food such as yoghurt, sauerkraut or cheese. Lately, it has grown into a Gram-positive recombinant expression host alternative to B. subtilis or E. coli. Its genetic toolbox is well stocked and it secretes the RiPP lantibiotic nisin as well as other bacteriocins into the extracellular environment.^254^ Particularly for RiPPs, the incorporation of noncanonical amino acids could be an attractive asset to tune their therapeutic properties.^200^

An early study by El Khattabi et al. reported the successful SPI of Trp analogs into the recombinant model proteins PA90 and PA6 in an L. lactis Trp auxotroph.^73^ PA90 is the peptidoglycan binding domain of L. lactis’s major autolysin AcmA, it contains five Trp residues. PA6 is a fragment of PA90 containing a single Trp residue. PA90 as well as PA6 were secreted into the medium by the Trp auxotrophic L. lactis strain PA1002 (Table 1), which was supplemented with 5HW, 7AW, 5FW, and 5-methyl-l-tryptophan (5mW) (Figure 11). All variant proteins (Table 2) were produced at comparable titers as the parent proteins with Trp. 7AW and 5FW were incorporated into PA6­[7AW] and PA6­[5FW] at >97% efficiency, while the incorporation of 5HW into PA6­[5HW] was slightly less efficient (89%). To the surprise of the authors, 5mW was introduced into PA6­[5mW] at an efficiency of 92%. This was an unexpected finding because attempts to incorporate this Trp analog into a recombinant protein in E. coli had failed before.^120^ It suggested that TrpRS from L. lactis, *Ll*TrpRS, had a more relaxed substrate specificity than *Ec*TrpRS.^73^

A follow-up study by Petrović et al. confirmed this hypothesis.^96^ They equipped strain PA1002 with a plasmid for the constitutive co-overexpression of *Ll*TrpRS with the model protein. They used the W20LysM tandem protein, which contained a single Trp residue, as the target for Trp analog incorporation. In contrast to *Ll*TrpRS, which was well overexpressed in the cytosol, W20LysM was secreted to the medium to ease its isolation and downstream analysis. The analogs 5FW, 5HW, 5mW, and 5,6-difluoro-l-tryptophan ([5,6]­dFW) were incorporated with excellent efficiencies >94%. The halogenated Trp analogs 5-bromo-l-tryptophan (5BrW), 6-chloro-l-tryptophan (6ClW) and 6-bromo-l-tryptophan (6BrW) (see Trp analog structures in Figure 11), reached ∼90% incorporation efficiency (Table 2). Clearly, the overexpression of *Ll*TrpRS improved the incorporation of Trp analogs with bulky substituents and a platform process for their efficient SPI in L. lactis was devised.^96^

Zhou et al. adapted this process for the production of secreted RiPPs in L. lactis by fine-tuning the expression conditions.^255^ As outlined in Section 3.3.1, RiPPs are first translated at the ribosome and subsequently matured by enzymatic PTMs. To produce a mature ncAA-labeled RiPP it is therefore necessary to coexpress the PTM enzymes together with the gene encoding the RiPP. However, misincorporation of the ncAA into the PTM enzymes could impair their function. To avoid misincorporation, Zhou developed a strategy for the differential expression of the PTM enzymes and the RiPP. They expressed the *nisA* gene encoding nisin in tandem with the *trpRS* gene encoding *Ll*TrpRS under the control of an inducible promoter. The *nisBTC* genes encoding the PTM enzymes were expressed from a differentially inducible promoter. The expression of *nisBTC* was induced first, in the absence of the Trp analog to prevent its misincorporation at any of the 17 Trp residues of NisBTC. After sufficient functional NisBTC was produced, the expression of *nisA*/*trpRS* was initiated with the second inducer (“cross-expression system”, see also the discussion of this matter in Section 5.3). Since *Ll*TrpRS does not contain any Trp residues, it could be coexpressed with nisin because misincorporation of Trp analogs causing a potential inactivation of the enzyme could not occur. Wild-type nisin does not contain any Trp residue, so four single Trp mutants were generated for the SPI of the analogs 5FW, 5HW and 5mW. Nisin^I1W^, nisin^I4W^, and nisin^M17W^ were well expressed but the expression level of the nisin^V32W^ mutant was noticeably lower. All three Trp analogs were incorporated into the mutant nisins, but the protein titers decreased in the order Trp ≫ 5FW > 5HW > 5mW for each mutant. The analog incorporation efficiency into mutants nisin^I1W^, nisin^M17W^, and nisin^V32W^ was generally >97%. However, mutant nisin^I4W^ showed heterogeneous analog incorporation, the efficiency was highest for 5HW (93%), followed by 5FW (89%) and lowest for 5mW (69%). Not all variant proteins could be isolated in sufficient amounts to test their antibiotic activity. All variants that could be purified showed at least 2- to 4-fold lower antibiotic activity than wild-type nisin. Most notably, the antibiotic activities of the nisin^M17W^ parent and the nisin^M17W^[5HW] variant were reduced by 32-fold vis-à-vis the wildtype (Table 2).^255^

Deng et al. expanded the SPI of ncAAs in L. lactis to Met analogs.^71^ They employed the cross-expression system described by Zhou et al.^255^ but without the overexpression of MetRS. In the Met auxotrophic L. lactis strain NZ9000 (Table 1), they first assessed the replacement of the two Met residues at positions 17 and 21 of wild-type nisin with Aha, Hpg, Nle, Eth, l-norvaline (Nva), and l-allylglycine (Alg) (Figure 7). Nisin­[Aha], nisin­[Hpg], nisin­[Nle], and nisin­[Eth] were produced albeit at lower titers than the parent (Table 2). Nva and Alg were not incorporated. Next, they analyzed whether the incorporation efficiency was position-dependent. They generated four nisin single Met mutants, nisin^M17I^, nisin^M21V^, nisin^M17I M21V M35^ (Met residue added at the C-terminus), and nisin^I4M M17I M21V^ carrying the single Met residue close to the N-terminus. The incorporation efficiencies of the Met analogs varied widely, between 50% for nisin^M17I M21V M35^[Nle] and >99.5% for nisin^M17I M21V M35^ containing Aha or Hpg, and they declined in the order Aha > Hpg > Nle > Eth in all peptides except for nisin^M17I M21V M35^, where Aha and Hpg replaced the C-terminal Met equally quantitatively and Eth was more efficiently incorporated than Nle. Apparently, the side chain chemistry, i.e., the acceptance as a substrate for L. lactis MetRS, directed the incorporation efficiency rather than the position in the nisin sequence. The mutant nisin^M21V^ killed the indicator strain Micrococcus flavus more efficiently than wild-type nisin, and its variants nisin^M21V^[Aha] and nisin^M21V^[Hpg] were equally effective (Table 2). Nisin variants with both Met residues exchanged against Aha or Hpg were also more active than the wild-type nisin. The activities of all nisin^M17I^ and nisin^M17I M21V M35^ variants were reduced. As well, the spectrum of the antimicrobial activities was shifted by the Met analogs. The Aha and Hpg variants of nisin^M17I^, nisin^M21V^, and nisin^M17I M21V M35^ were cross-linked *via* their compatible reactive groups by CuAAC. Depending on the cross-link position, the dimeric nisin conjugates retained more or less antimicrobial activity but all were less active than the wildtype. The Aha- and Hpg-labeled nisin variants were also labeled with alkyne- and azide-fluorophores to study their interaction with the indicator strain Entercoccus faecium by fluorescence microscopy. They were all antimicrobially active and localized to the cell membrane. Nisin^M21V^[Aha]-6FAM was the most effective nisin-dye conjugate.^71^ The study by Deng et al. demonstrated first that the SPI of different Met analogs is possible in L. lactis. However, the titers of the variant proteins were quite low.

In a very recent study, Kuipers and co-workers improved titers of the SPI process for Met analogs.^256^ They exchanged the promoters of their cross-expression system such that nisin was expressed from the relatively stronger promoter while *nisBTC* was under control of the tighter yet weaker promoter. This changed the incorporation efficiencies of Aha, Nle and Eth dramatically, Met was quantitatively exchanged by any of them in all tested nisin mutants. As well, they could substantially elevate the protein titers by ∼7-fold in comparison to earlier studies.^71,255^ Nisin­[Aha] reached 88% of the parent protein titer, and nisin­[Nle] and nisin­[Eth] reached ∼55% (Table 2). Using this improved cross-expression system, they incorporated Aha, Nle and Eth into various single Met mutants of nisin and assessed their antimicrobial activity. The activity (% increase relative to the corresponding parent indicated in parentheses) of variants nisin­[Nle] (5%), nisin^M21V^[Aha] (5%), nisin^I1M M17I M21V^[Nle] (7%), and nisin^M21V^[Eth] (11%) increased moderately, while variants nisinΔ^M17I^[Nle] (65%), nisin^I1M M17I M21V^[Eth] (64%), nisinΔ^M21V^[Eth] (50%), and nisin­[Aha] (30%) showed a marked increase in their antimicrobial activities (Table 2). None of the Met analogs improved the activities of nisin^M17I M21V M35^ and nisinΔ^M17I^. NisinΔ were C-terminally truncated nisin mutants comprising amino acids 1–22. For all other tested variants, the activity was unchanged or lower than that of the corresponding parent. Eth and Nle had an activity increasing effect on three proteins and Aha on two. Finally, the variants containing a single Aha residue were artificially lipidated with 1-undecyne by CuAAC. Lipidation increased the antimicrobial activity of the full-length variants at low concentration but not of the C-terminally truncated ones.^256^ In summary, L. lactis has been established as a suitable host for the SPI of Met and Trp analogs into secreted proteins.

The methylotrophic yeast K. phaffii (aka P. pastoris) offers the same assets as S. cerevisiae for recombinant protein production yet it often produces higher recombinant product titers.^257^ It can produce high levels of large proteins and protein complexes as well as membrane proteins, performs post-translational modifications, and even more importantly, it secretes proteins very efficiently into the culture medium. Budisa and co-workers chose Candida antarctica lipase B (CalB) as the secreted model protein and introduced the N74D mutation to remove a nonessential glycosylation site. Glycosylation is often heterogeneous, which complicates the mass analysis of the congeners. They used the strain X33 Δ*aro1*, which is auxotrophic for the aromatic cAAs Phe, Trp, and Tyr (Table 1), for the incorporation of 5FW, 3FY, and 4FF. The fluorinated CalB variants were produced in a range of 30–60% of the parent protein (Table 2). All analogs were incorporated but the labeling was incomplete and highly stochastic. The fluorination of the aromatic residues of CalB prolonged its long-term shelf life.^155^ P. pastoris performed better than S. cerevisiae in terms of product titers and also ncAA incorporation efficiencies although a direct comparison is not truly warranted since different ncAAs and different model proteins were used. Nevertheless, P. pastoris is a very promising eukaryotic candidate to further develop SPI of ncAAs in secreted proteins.

##### 4.1.1.2 Transmembrane Proteins

Gupta and colleagues used Xenopus laevis oocytes as the host for Aha (Figure 7) incorporation to study the structural dynamics of a transmembrane protein.^159^ Shaker Kv is a voltage-sensitive ion channel transmembrane protein on the surface of Xenopus oocytes. Gupta et al. generated a mutant Shaker Kv which exposed only a single Met and Cys residue each at different positions in the extracellular part of the protein channel.^159^ They replaced Met by Aha and labeled it with a DBCO-fluorophore using SpAAC while the Cys residue was simultaneously functionalized with a distinct thiol-reactive maleimide-fluorophore. Aha had been positioned in the voltage-sensing domain of the Shaker Kv and Cys in the pore domain, consequently, their fluorescence-voltage readouts differed radically. The dual fluorescent labeling generated distinguished signals and allowed the authors to study the voltage-dependent dynamics of the ion channel.

#### 4.1.2 Overexpression of Wild-Type and Mutant Aminoacyl-tRNA Synthetases

Many ncAAs are well incorporated by SPI when the corresponding AARSs are expressed at their natural levels in the host. NcAAs whose structure, chemistry or intracellular availability pushes the substrate tolerance of the host AARSs to a limit may need more or less gentle “coaxing” into ribosomal translation. Simply elevating the AARS’s expression levels can suffice, if this is not the case, the amino acid binding pocket can be engineered, or an editing function can be addressed by mutation (Figure 15). In this section, we summarize the measures the community has adopted to coax the SPI of ncAAs. So far, these efforts have not been as elaborate and sophisticated as those employed for the engineering of orthogonal AARS/suppressor tRNA pairs for SCS. Yet, the reassignment of sense codons to ncAAs has requested a great deal of tinkering with the components of the translation apparatus and has been emerging as “the third in the league” with SPI and SCS. The recent review by Hartman provides a comprehensive overview of E. coli AARSs and their mutants for the incorporation of ncAAs by different incorporation methods.^27^

Depending on whether *Ec*IleRS or *Ec*ValRS was overexpressed in the Ile/Leu double auxotrophic E. coli strain AIV-IQ (Table 1), (2*S*,3*R*)­Tfv (see Val and Ile analog structures in Figure 13) was incorporated at Ile or Val codons of murine dehydrofolate reductase (mDHFR). The other stereoisomer, (2*S*,3*S*)-4,4,4-trifluorovaline ((2S,3S)­Tfv) was not accepted as a substrate by either AARS.^109^

*Ec*IleRS accepts (2*S*,3*S*)-2-amino-3-methyl-4-pentenoic acid ((2*S*,3*S*)­enI) as a substrate but not the diastereomer (2*S*,3*R*)-2-amino-3-methyl-4-pentenoic acid ((2*S*,3*R*)­enI). The first could be incorporated into mDHFR (Table 2) with very good efficiency (>70%) even without the overexpression of IleRS but the other diastereomer could not. The terminal double bond of (2*S*,3*S*)­enI might be useful for future coupling reactions, e.g., thiol-ene click chemistry.^258^

The overexpression of the wild-type TrpRS from L. lactis allowed very efficient incorporation of Trp analogs with bulky substituents in the Trp auxotrophic L. lactis strain PA1002 (Table 1 and Section 4.1.1.1).^96,255^

Overexpression of the wild-type *Ec*TyrRS as well as the *Mj*TyrRS^Y32L A67S H70N A167Q^ mutant, aka *Mj*DhpRS from M. jannashii, improved the incorporation of Dopa into mussel adhesive protein.^78^

The Pro analogs 3cHP, 3tHP, 4cNP, 4dFP, 4oP, Aze, dhP, phP, and Thz (Figure 12) require the overexpression of *Ec*ProRS. Pip and 4tmP as well as 4enP (Figure 12) were incorporated when the ProRS mutants C443G (*Ec*ProRS^C443G^) and M157Q (*Ec*ProRS^M157Q^) were coexpressed from a multicopy plasmid.^34,66,74,92,221^

In 1991, Kast and Hennecke devised the E. coli PheRS mutant A294G (*Ec*PheRS^A294G^).^259^ Subsequent studies demonstrated the greatly relaxed substrate specificity of this mutant for Phe analogs with substituents in the *para* position.^260–263^ *Ec*PheRS^A294G^ was extensively employed, for instance, for the proteome-wide incorporation of 4yF (Figure 9)^264^ or the functionalization of protein biomaterials with 4-azido-l-phenylalanine (4azF) (Figure 9).^67,91,265^ As well, it inspired the generation of the analogous mutant *Bm*PheRS^αA450G^ for the incorporation of 4azF into the silk fibroin of B. mori (see Section 4.2.2).^266^

The Tirell group engineered the editing function of LeuRS from E. coli such that the enzyme accepted Met analogs as substrates.^100^ They replaced residue Thr252, which is essential for the editing function of *Ec*LeuRS, with bulkier cAAs. Using the *Ec*LeuRS^T252Y^ mutant in the Leu auxotrophic E. coli strain LAM1000 (Table 1), they successfully exchanged eight Leu residues in the synthetic leucine zipper protein LzipA1 with the following Met analogs: Alg, (2*S*)-2-aminohex-4-ynoic acid (H4y), l-homoallylglycine (Hag), Hpg, Nle, and Nva (Figure 7). However, due to its compromised editing function, *Ec*LeuRS^T252Y^ mis-incorporated Ile, Val and Met at Leu positions. The omission of these cAAs from the expression medium suppressed the background incorporation but it caused incorporation of the Met analogs into Met codons. The Met analogs were supplemented at 320 mg/L and under these conditions they competed efficiently with biosynthesized Met (strain LAM1000 is not Met auxotrophic) as substrates for the host MetRS.^100^ The Tirrell group used the *Ec*LeuRS^T252Y^ mutant also to incorporate the reactive Leu analog Onv (Figure 13) into LzipA1. They showed that the ketone functions installed in LzipA1­[Onv] were accessible for oxime coupling with hydrazine-labeled biotin. The editing function of wild-type *Ec*LeuRS is amazingly selective: It prevented the incorporation of Onv whereas its hydrocarbon isostere 4-methylene-l-norvaline (Env) (Figure 13) passed the editing and was incorporated.^101^

Fluorescence reporter assays are a convenient method to screen orthogonal AARS/suppressor tRNA pairs. Whether stop codon readthrough occurs can be assessed straightforwardly by a simple yes/no output that is detected in a fluorescence plate reader or by flow cytometry.^267,268^ To screen AARS mutants for the incorporation of ncAAs at sense codons the fluorescence assay would have to be robust enough to differentiate subtle differences in fluorescence intensity from mere fluorescence fluctuations. As the readout occurs upon the incorporation of the ncAA as well as cAAs, such an assay could not benefit from a simple yes/no output. It would have to reliably measure the *fluorescence increase* that is caused by the incorporation of the ncAA.

Tirrell and co-workers elegantly solved this predicament. They developed a convenient cell surface display method (Figure 16) to screen MetRS mutants for the incorporation of Met analogs with reactive side chain chemistries.^269^ They chose the outer membrane protein C (OmpC) from E. coli as the reporter. OmpC contains three Met residues, to amplify the readout signal six more Met residues were introduced at exposed loops by site-directed mutagenesis. Incorporated Met analogs such as Aha or Anl that both contain a reactive azide group (Figure 7) would thus be exposed on the cell surface. After conjugation of the azide groups with a detectable tag such as alkyne-biotin, cells that efficiently incorporated the Met analogs could be detected by labeling them with fluorescently labeled streptavidin. Cells displaying an elevated fluorescence readout could be discriminated from less fluorescent or nonfluorescent cells by flow cytometry. The intensity of the fluorescence signal was a direct consequence of the side chain functionality of the ncAA, thus, the incorporation of Met would not provoke a fluorescence signal. In this way, Tirrell and co-workers devised a fluorescence assay that benefits from a yes/no readout for the decoding of Met sense codons with reactive Met analogs.

**Figure 16:**
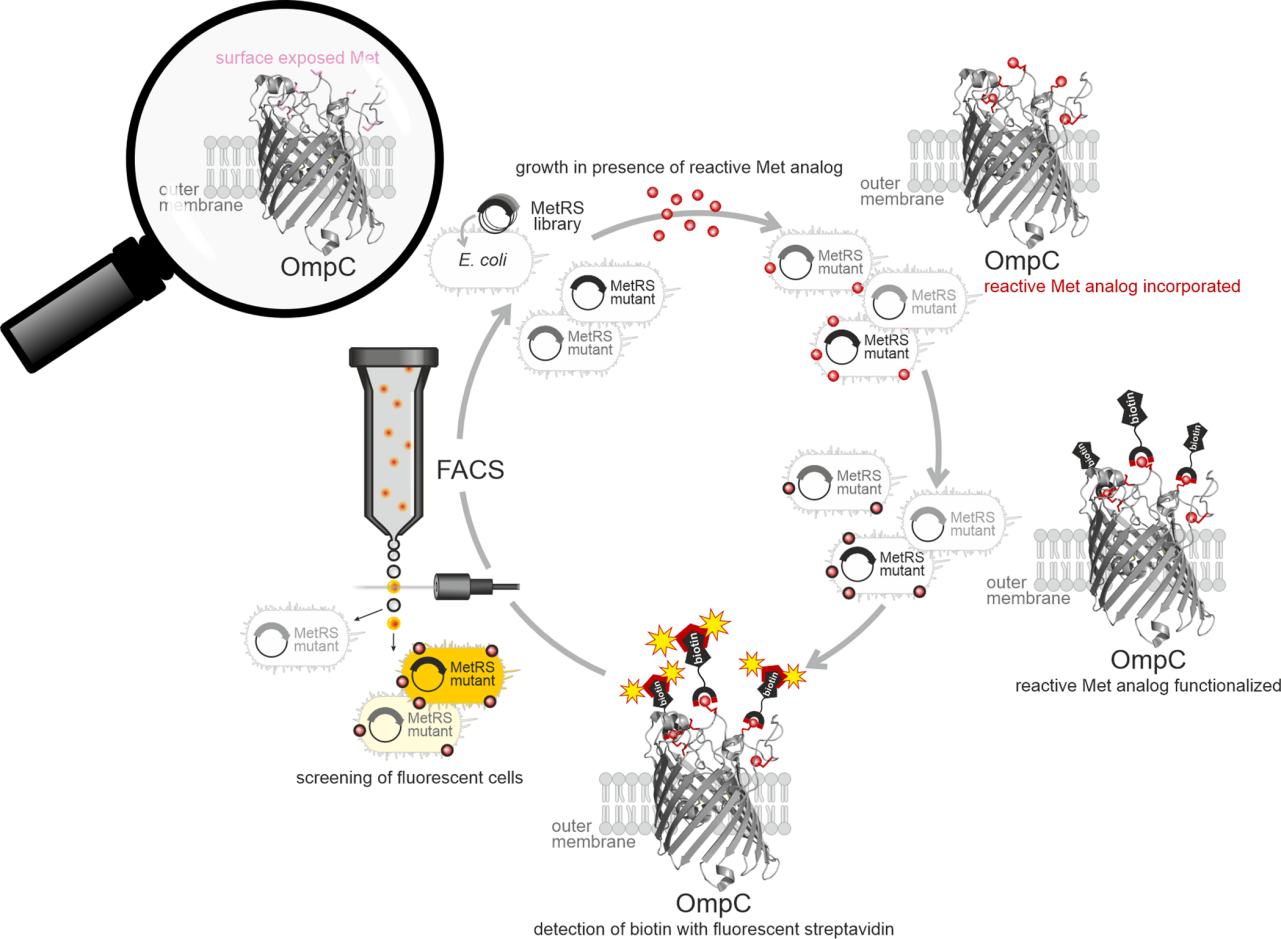
E. coli cell surface display assay to screen MetRS mutants for the incorporation of Met analogs with reactive side chain chemistries. The cells are transformed with a MetRS plasmid library. During growth, the reactive Met analog is incorporated into OmpC, a transmembrane protein in the outer membrane. Surface exposed reactive groups can undergo bioorthogonal conjugation with correspondingly functionalized biotin. Finally, biotinylated cells bind fluorescent streptavidin and can be isolated by fluorescence-activated cell sorting. Isolated mutants can be subjected to another round of screening, e.g., with increased stringency.

Using their new cell surface fluorescence display assay, the Tirrell group assessed the incorporation of a variety of azido-reactive Met analogs with an overexpressed *Ec*MetRS. During their initial study outlined above, they had generated the E. coli strain M15MA, which is a Met auxotrophic descendant of E. coli M15 from Qiagen.^269^ This strain was transformed with a multicopy expression construct for the OmpC^9M^ reporter (mutant OmpC^V50M N88M T187M A230M L271M L306M^) and the pREP4 plasmid encoding the *lacI* repressor to silence the basal expression of the reporter in the resulting E. coli strain M15MA/pREP4 (Table 1). To coexpress *Ec*MetRS, they included a copy of the *metG* gene under its own *metG1*p promoter on the OmpC^9M^ expression vector.^84^ Using the resulting reporter strain, they screened the incorporation of a panel of aliphatic azido-amino acids, Aza, Aha, Anv, and Anl (Figure 7) by fluorescence-activated cell sorting (FACS). As expected, Aha was incorporated without the overexpression of *Ec*MetRS but Aza, Anv and Anl were incorporated at detectable levels only if *Ec*MetRS was overexpressed. The study revealed that the use of CuBr as the catalyst for CuAAC of the alkyne-biotin was essential to detect weak Met analog incorporation.^84^

Next, they applied the cell surface fluorescence display assay to screen an *Ec*MetRS mutant library against Anl (Figure 7).^85^ The best performing candidate *Ec*MetRS^L13G^ still activated Met but with an approximately 300-fold lower efficiency than the wild-type enzyme. As such, it performed with Anl in a comparable manner as wild-type *Ec*MetRS with Aha and Hpg. The model protein mDHFR­[Anl] (Table 2) was produced at a titer corresponding to ∼50% of the parent DHFR and the efficiency of the Anl incorporation was very high (∼95%). Tanrikulu et al. redesigned the *Ec*MetRS library to further enhance the activity of mutant enzymes toward Anl and to improve the discrimination against Met in parallel.^102^ *Ec*MetRS^L13N Y260L H301L^ (*Ec*MetRS^NLL^) emerged as the best performing mutant enzyme from the cell surface display assay. It provoked an almost complete replacement of Met by Anl in the model protein mDHFR and Anl was incorporated at substantial levels even in the presence of Met in the expression medium. *Ec*MetRS^NLL^ has been used extensively for the proteome-wide incorporation of Anl (see Section 4.3.1 below).

The cell surface fluorescence display assay^269^ described above works with reactive ncAAs. To devise a more general screening method for ncAAs that lack reactive side chain chemistry, Yoo and Tirrell turned to GFP as the reporter.^112^ As outlined above, it was essential to generate sufficient sensitivity for the ncAA incorporation under SPI conditions. However, the replacement of the Met residues in GFP led to the loss of its green fluorescence. To render the fluorescence insensitive to the incorporation of Met analogs, they generated a GFP mutant lacking all Met residues in the β-barrel structure. Two Met residues remained, but their exchange would have been too insensitive to reliably measure MetRS activity. Thus, they reintroduced five Met residues into permissive positions in the loop regions connecting the β-sheets such that the resulting GFPrm_AM mutant contained a total of seven Met residues. They confirmed that the newly introduced Met positions permitted the incorporation of Aha, Hpg and Nle. These analogs only modestly reduced the GFP fluorescence, thus, GFPrm_AM was a suitable screening reporter. They applied the redesigned fluorescence assay to isolate an *Ec*MetRS mutant for the incorporation of the nonreactive Met analog 6,6,6-trifluoro-l-norleucine (Tfn, Figure 7). Mutant *Ec*MetRS^L13S Y260L H301L^ (*Ec*MetRS^SLL^) was indeed a TfnRS. It efficiently replaced seven and eight Met residues in GFPrm_AM and mDHFR. GFPrm_AM­[Tfn] was produced at only ∼14% of the parent protein but it fluoresced brightly, which indicates that Tfn was well tolerated by the engineered protein structure. The titer of mDHFR­[Tfn] exceeded that of the parent protein by 50% and Met was replaced nearly quantitatively by Tfn (Table 2).^112^

Truong et al. employed the fluorescence screening assay to isolate an *Ec*MetRS mutant for the incorporation of Pra (Figure 7).^270^ Although the ncAA contains a reactive side chain, the authors decided not to use the cell surface fluorescence display assay.^269^ Pra’s alkyne moiety would have called for CuAAC to install an azide-biotin to attach the fluorescent streptavidin marker, but they did not want to expose the expression host to Cu­(I) during the screening. Truong et al. isolated a PraRS (mutant *Ec*MetRS^L13P A256G P257T Y260Q H301F A331V Δ548E^) that facilitated near quantitative exchange of Met for Pra. They used the PraRS in combination with *Ec*MetRS^NLL^ to selectively label two cell populations with Pra and Anl (see Section 4.3.1 below).^270^

#### 4.1.3 Breaking the Degeneracy of Sense Codons

In 2003, Kwon, Kirshenbaum, and Tirrell demonstrated the first reassignment of a sense codon.^278^ For their approach, they exploited the fact that the genetic code is degenerate, that means that for instance Phe is encoded by two codons, UUU and UUC (TTT and TTC on DNA). In E. coli both codons are read by a single tRNA with the anticodon GAA. tRNA_GAA_^Phe^ perfectly hybridizes with UUC codons by Watson–Crick base-pairs, but to read UUU codons, the first base of the anticodon (G) wobble-base pairs with the third base of the codon (U). Kwon et al. rationalized that, because the G-U wobble base pair was weaker than the Watson–Crick pair A-U, a tRNA_AAA_^Phe^ would Watson–Crick pair with the UUU codon and decode it faster than tRNA_GAA_^Phe^. They generated a mutant tRNA_AAA_^Phe^ from S. cerevisiae and introduced it into E. coli together with the yeast mutant *Sc*PheRS^T415G^, which accepts 3-(2-naphthyl)-l-alanine (Nal) (Figure 9) as the substrate. With this heterologous pair, they were able to decode four UUU codons in their model protein mDHFR preferentially with Nal while the remaining five UUC codons were occupied by Phe. They performed the Nal incorporation experiment in Phe-free minimal medium and used the Phe auxotrophic E. coli host K10-F6Δ (Table 1), which carries the *Ec*PheRS^A294S^ mutant (*pheS13*)^259,271^ that excludes 4FF (Figure 9) from its binding site. The expression of the heterologous *Sc*PheRS^T415G^/*Sc*tRNA_AAA_^Phe^ pair under these conditions biased the decoding of the UUU codon with Nal.

In a follow-up study, Kwon and co-workers exploited the same approach for the reassignment of the Leu UUG codon. In the Phe/Leu double auxotrophic E. coli strain MPC390 (Table 1), they coexpressed yeast *Sc*tRNA_CAA_^Phe^ with the mutant yPheRS_naph (*Sc*PheRS^N412G T415G S418C S437F^), which recognized Nal more efficiently than *Sc*PheRS^T415G^.^272^ Nal-*Sc*tRNA_CAA_Phe competed with Leu-*Ec*tRNA_CAA_^Leu^ for the Leu UUG codon such that a Leu-to-Nal reassignment efficiency of 50% resulted.^273^

These initial experiments clearly indicated that an efficient sense codon reassignment (SCR) (Figure 17) required the careful choice of a suitable sense codon and an orthogonal ncAARS/tRNA pair to decode it, similar as for SCS. For instance, Budisa and co-workers intended to install mutually orthogonal MetRS/tRNA^Met^ pairs from E. coli and the archaeon Sulfolobus acidocaldarius for the differential decoding of initiator and internal AUG codons with Aha and Eth (Figure 7). However, this attempt failed because the bacterial and archaeal MetRS/tRNA^Met^ pairs were not strictly orthogonal.^274^

**Figure 17:**
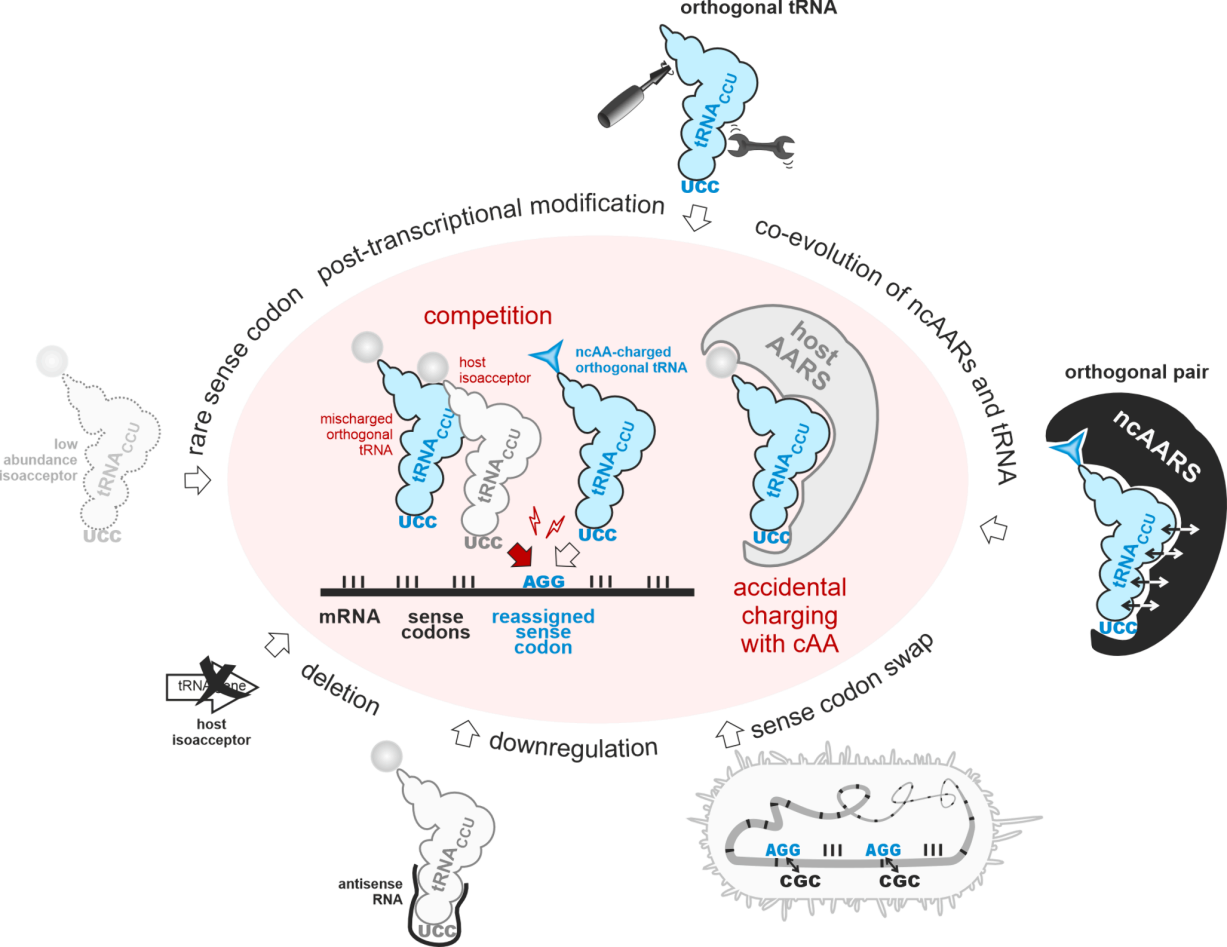
Challenges of sense codon reassignment (pink area) and strategies to meet them (outside circle) at the example of the rare E. coli Arg codon AGG. The orthogonal sense codon decoding tRNA can be accidentally aminoacylated by a host AARS. The mischarged orthogonal tRNA and the synonymous host isoacceptor compete with the ncAA-charged orthogonal tRNA for the sense codon. Possible approaches to evade the competition range from the choice of sense codon over different strategies to moderate the competing host isoacceptor to the engineering of ncAARS/tRNA pairs. See the text for more details.

SCR combines aspects of SCS and SPI of ncAAs. The intention is to “free” individual degenerate sense codons to assign a new meaning (i.e., an ncAA) to them. Degenerate sense codons encoding the same cAA are decoded by synonymous tRNAs, so-called isoacceptors. Decoding of the selected sense codon by a host isoacceptor tRNA *via* wobble base pairing is advantageous. An orthogonal tRNA with a perfectly Watson–Crick base pairing anticodon provides a thermodynamic advantage over a synonymous wobbling host tRNA.^40,273,275–280^ Ideally, the corresponding orthogonal ncAARS recognizes the orthogonal tRNA independently of the anticodon, which is the case, for instance, for pyrrolysyl-tRNA synthetase (PylRS).^281^ Considering these aspects, Söll and co-workers successfully reassigned the frequent Ser AGU codon (*Ec*SerRS does not recognize the anticodon of tRNA^Ser^) to 3-iodo-l-phenylalanine (3IF) (Figure 9) with 65% efficiency using the mutant *Mm*PylRS^N346S C348I^/*Mm*tRNA_ACU_^Pyl^ pair from Methanosarcina mazei. They used a prototrophic E. coli strain and neither downregulated nor deleted the host isoacceptor, which resulted in the accidental incorporation of Ser (33%) and Phe and Thr (1% each) to some extent.^276^

Two major obstacles prevent the quantitative reassignment of sense codons. (i) Sense codon-decoding orthogonal tRNAs compete with the corresponding host tRNAs and (ii) they can be accidentally aminoacylated with cAAs by the host AARSs. Choosing a rare sense codon for reassignment can reduce the competition by the host tRNAs because they occur at low abundance.^282^ For instance, Odoi et al. attempted to incorporate *N* ^ε^-(*tert*-butyloxycarbonyl)-l-lysine (BocK) (Figure 13) with the *Mm*PylRS/*Mm*tRNA_CCU_^Pyl^ pair in response to an AGG codon in the prototrophic E. coli strain BL21­(DE3).^279^ However, despite AGG being the least frequent Arg codon in E. coli, Arg was exclusively incorporated at AGG codons. Most probably, the intracellular level of charged BocK-*Mm*tRNA_CCU_^Pyl^ was too low to compete efficiently with E. coli tRNAs reading Arg AGG codons. The Söll group chose Mycoplasma capricolum as the host for the reassignment of the Arg CGG codon. In this organism, the CGG codon is extremely rare, it occurs only six times in its entire genome. M. capricolum does not encode a dedicated tRNA_CCG_^Arg^ and the CGG codon is only inefficiently decoded by the other Arg isoacceptors.^283^ Nevertheless, reassignment of Arg CGG by the *Mb*PylRS/*Mb*tRNA_CCG_^Pyl^ pair derived from Methanosarcina barkeri was unsuccessful most likely because the host ArgRS cross-charged the recombinant archaeal *Mb*tRNA_CCG_^Pyl^ with Arg.^284^

To avoid competition by the host isoacceptor tRNA and improve the reassignment of the rare AGG codon in E. coli, Liu and co-workers expressed an antisense RNA to downregulate the competing *Ec*tRNA_CCU_^Arg^. However, the downregulation only marginally improved the reassignment of the Arg AGG codon to the lysine derivatives BocK, *N* ^ε^-(allyloxycarbonyl)-l-lysine (AllocK), and *N* ^ε^-(propargyloxycarbonyl)-l-lysine (ProK) (Figure 13) by the *Mb*PylRS/*Mb*tRNA_CCU_^Pyl^ pair from 80% to ∼90%.^285^ To further tone down the competition by Arg-charged host isoacceptors, Lee et al. generated an *argA argW* double knockout of E. coli strain DH10B, which rendered it Arg auxotrophic and eradicated *Ec*tRNA_CCU_^Arg^. Using the *Mj*AzFRS-1/*Mj*tRNA_CCU_^Tyr^ variant pair from M. jannashii (*Mj*AzFRS-1, aka *Mj*TyrRS^Y32T E107N D158P I159L L162Q D286R^),^286^ they achieved the quantitative reassignment of the rare Arg AGG codon to different Phe analogs (4azF, 4-acetyl-l-phenylalanine (4acF), and *p*-benzoyl-l-phenylalanine (Bpa); Figure 9) and one Tyr analog (*O*-propargyl-l-tyrosine, OpY; Figure 10) in complex medium. Multiple AGG codons, also in tandem, were successfully reassigned.^287^ The Sakamoto group took this approach to the next level. They abolished the competition by the E. coli tRNAs that read both rare Arg codons, AGG and AGA. *Ec*tRNA_UCU_^Arg4^ (encoded by *argU*) reads AGA codons by Watson–Crick base pairing and AGG codons by wobble base paring between the third base of the codon and the first base of the anticodon. *Ec*tRNA_CCU_^Arg5^ (encoded by *argW*) reads only AGG codons. Deletion of both *argU* and *argW* genes is lethal in E. coli. However, the authors replaced AGG codons in essential genes with synonymous Arg codons, tightly controlled the expression of recombinant bacteriophage-T4 tRNA_UCU_^T4^ and overexpressed the mutant *Mm*HarRS/*Mm*tRNA_CCU_^Pyl^ pair from Methanosarcina mazei, which remedied the lethality of the *argUW* double knockout. Using this strain, they reassigned the AGG codon to the close Arg analog l-homoarginine (Har) (Figure 14) in a target protein as well es the entire proteome.^288^ In a follow-up study, they used the same strain equipped with two mutually orthogonal ncAARS/tRNA pairs, to engineer a microbial transglutaminase by the site-specific incorporation of two different ncAAs at amber stop and AGG codons.^289^ However, the genome-wide swapping of a sense codon to synonymous codons is not as straightforward as it appears since extensive sense codon swapping had largely deleterious effects in E. coli.^290^ The generation of an E. coli strain with a recoded genome in which the serine codons TCG and TCA, and the amber stop codon TAG, 1.8 × 10^4^ codons in total, were systematically replaced by their synonyms AGC, AGT and TAA required highly sophisticated genome design, synthesis, and assembly strategies.^7^

In addition to the abovementioned approaches, the interaction of the ncAARS with the tRNA and tRNA modifications can be exploited to reassign sense codons. The TyrRS of M. jannashii does not use the anticodon of its cognate *Mj*tRNA_GUA_^Tyr^ as a primary identity element, yet the interaction can be modulated by mutating residues in the anticodon loop. Wang and Tsao exploited this fact to evolve an anticodon binding domain mutant of *Mj*TyrRS for the improved recognition of an Arg AGA decoding *Mj*tRNA_UCU_^Tyr^. Then, they transferred known amino acid binding pocket mutations that make *Mj*TyrRS polyspecific for a variety of Phe and Tyr analogs onto the new *Mj*TyrRS­(AGA) mutant. The resulting *Mj*pCNFRS­(AGA)/*Mj*tRNA_UCU_^Tyr^ pair incorporated two Phe analogs (4-iodo-l-phenylalanine (4IF) and 4acF; Figure 9) and the Tyr analog *O*-methyl-l-tyrosine (OmY) (Figure 10) at a single AGA codon with >90% efficiency.^291^ To reduce the competition by *Ec*tRNA_UCU_^Arg^, they used an E. coli strain that expressed a compromised *argU*10­(Ts) allele. *Ec*tRNA_UCU_^argU10(Ts)^ occurs at lower intracellular levels than wild-type *Ec*tRNA_UCU_^Arg4^, is less efficiently arginylated by *Ec*ArgRS and its interaction with the elongation factor EF-Tu is impaired.^292^

The group of Fisk systematically explored the plasticity of E. coli’s genetic code for SCR.^280,293^ In contrast to Wang and Tsao, they mutagenized both orthogonal components. Co-evolution of the *Mj*tRNA^Tyr^ anticodon loop and the *Mj*TyrRS tRNA anticodon loop binding domain substantially improved the suppression of Lys AAG, Asn AAU, Phe UUU, and His CAU sense codons in the His auxotrophic E. coli strain SB3930 (Table 1).^275^ However, they observed that *Mj*tRNA_AUG_^Tyr^ was post-transcriptionally modified to *Mj*tRNA_IUG_^Tyr^ by the TadA A-to-I deaminase (I, inosine). This post-transcriptional modification of the tRNA resulted in the ambiguous decoding of CAC and CAU His codons. Mutations preventing the tRNA modification in the anticodon loop facilitated the unambiguous reassignment of the His CAU codon.^40^ Taking the same approach, they first optimized *Mj*TyrRS together with *Mj*tRNA_CCU_^Tyr^ for the decoding of rare Arg AGG codons. Then, they transferred the relevant mutations to *Mj*AzFRS^286^ to obtain *Mj*AzFRS­(AGG) $\left({Mj\mathrm{TyrRS‐C3}}^{\begin{array}{l} \mathrm{Y32T}\;\mathrm{E107N}\;\mathrm{D158P}\;\mathrm{I159L}\;\mathrm{L162Q}\;\mathrm{R223G}\\ \mathrm{C231F}\;\mathrm{P232Q}\;\mathrm{H283L}\;\mathrm{P284R}\;\mathrm{M285S}\;\mathrm{D286G} \end{array}}\right)$ ^294^ (*Mj*AzFRS­(AGG)). The resulting optimized *Mj*AzFRS­(AGG)/*Mj*tRNA_CCU_^Tyr‑C3^ pair accomplished the reassignment of the AGG codon to 4azF (Figure 9) at an efficiency of 99%. Interestingly, the pair decoded AGG codons in an Arg auxotrophic *argA argW* double knockout strain worse than in the wild-type background.^294^

The Budisa group took yet another approach to exploit the post-transcriptional modification of tRNA to break the degeneracy of Ile codons. In E. coli and other bacteria, the rare Ile AUA codon is decoded by tRNA_C34AU_^Ile2^. This tRNA carries the same anticodon as tRNA_CAU_^Met^ and can read the AUA codon only after C34 is post-transcriptionally modified with lysine by lysidine-tRNA synthase (TilS), which yields 2-lysyl-cytidine (base code L). If tRNA_CAU_^Ile2^ is not lysylated, it is recognized and charged with Met by MetRS and thus reads Met AUG codons. Some species, such as Mycoplasma mobile possess tRNA_UAU_^Ile3^ to directly decode AUA codons (Fabret et al.^295^ and references therein). Böhlke et al. deleted *tilS* to abolish the decoding of AUA codons by tRNA_LAU_^Ile2^ and were able to rescue the lethal *tilS*::Δ phenotype by the recombinant expression of the *M. mobile* IleRS/tRNA_UAU_^Ile3^ pair. In this way, they freed the AUA codon for proteome-wide reassignment to ncAAs such as Ile analogs.^296^

Taken together, the most promising approaches to reassign sense codons to ncAAs consist in the development of efficient orthogonal ncAARS/tRNA pairs for their decoding. Co-evolved ncAARS/tRNA pairs that outcompete the corresponding host tRNAs and avoid accidental aminoacylation of the orthogonal tRNA by host AARSs are key to success. The competition by cAA-charged host tRNAs appears to be reduced when auxotrophic strains are grown in complex media. Finally, the elimination of selected degenerate sense codons and their decoding tRNAs from the host genome is an exciting concept. Cell-free expression systems allow a more flexible combination of sense codons with orthogonal tRNAs and ncAARSs than live cells and as such represent a hotbed for SCR.^297–299^ A very recent review by Jones and Hartman highlights the latest developments in this emerging research field.^277^

#### 4.1.4 Engineering the Protein Matrix to Accommodate the ncAA

To compensate for the potentially structure destabilizing effects of ncAAs, the protein matrix can be mutated to better accommodate the ncAA (Figure 15). In particular, this approach can be useful if the incorporation of the ncAA occurs at many positions in the protein or when ncAAs with bulky side chains are to be incorporated, or both.

The Tirell group demonstrated the benefit of directed evolution to “mold” a protein structure such that an ncAA fits snugly into it at multiple positions. They evolved GFP to accommodate the polyfluorinated Leu analog Tfl (Figure 13).^300^ The GFP from which they started their directed evolution campaign contained 19 Leu residues. When replaced with Tfl, the protein folded incorrectly and, consequently, it lost its fluorescence. To evolve the protein, they randomized the gene sequence by error-prone PCR^301^ and expressed the mutant genes in the Leu auxotrophic E. coli strain DH10B (Table 1) in minimal Leu-free medium supplemented with Tfl. Fluorescent cells were sorted however, the fluorescence excited by Tfl incorporation hardly exceeded the background autofluorescence of the host cells. The fluorescence levels only increased when the reporter was expressed in the presence of Tfl spiked with a small amount of Leu. The genes isolated from top performing mutants were used to generate a new library which was screened in the presence of Tfl mixed with less Leu and eventually without it. After eleven rounds of directed evolution, the resulting GFP mutant had acquired 18 additional mutations, and the number of Leu decreased from 19 to 15. Tfl was excellently accommodated at 87% efficiency while the expression level as well as the physical and spectroscopic properties of the variant GFP were comparable to the start GFP. The folding not only of the GFP11.3.3­[Tfl] (Table 2) improved but also of the parent protein with Leu. Obviously, directed evolution in the presence of Tfl had developed a “superfolder” GFP that displayed elevated expression levels, improved folding kinetics and an enhanced resistance to chemical denaturation.^300^

Montclare and Tirrell followed a similar directed evolution strategy to adapt chloramphenicol acetyltransferase (CAT) to the global replacement of 13 Leu residues by Tfl.^87^ Similar to GFP, CAT’s thermostability was severely compromised by the Leu→Tfl exchange. They generated a mutant library by error-prone PCR, introduced it into the Leu auxotrophic E. coli host LAM1000 (Table 1) and subjected it to two rounds of directed evolution in the presence of Tfl in Leu-free minimal medium. The thermostability of the best performing variant CATL2-A1­[Tfl] slightly outperformed that of the wild-type CAT. Relative to CAT, the evolved CATL2-A1 sequence contained three amino acid substitutions while the 13 Leu residues remained unchanged. A nonsense-mutation truncated the protein by one residue.^87^ In summary, directed evolution is an excellent strategy to tailor a protein’s structure for the accommodation of an ncAA at multiple positions.

Nagasundarapandian et al. embarked on a semirational approach to engineer GFP for an improved incorporation efficiency of the Met analogs Aha, Hpg, Nle, Mox, and Eth (Figure 7).^189^ Similar to the report by Yoo et al.,^300^ the global replacement of the six Met residues of GFPcon drove the protein into insolubility and reduced its fluorescence. To stabilize GFPcon, the authors introduced 12 mutations into the sequence that had been described in the literature to improve GFP’s folding robustness. Indeed, the resulting mutant GFPhs1 folded faster than GFPcon. GFPhs1 readily incorporated the Met analogs (Table 2) at an efficiency >90% and the titers of the variant proteins reached ∼20–50% of the parent protein with Met. Relative to GFPhs1, GFPhs2 contained nine additional stabilizing mutations described in the literature, however, they did not further improve the protein’s robustness toward Met analog incorporation. This study demonstrated that semirational design is another suitable approach to manipulate the plasticity of a target protein for improved ncAA incorporation.

#### 4.1.5 Combination of SPI and SCS in a Single Experiment

The combination of SPI and SCS in a single experiment can be useful to combine the site-specific installation of a reactive handle, such as a photoreactive or bioorthogonal group with the global manipulation of the physicochemical properties of a protein, e.g., improved stability (Figure 15). Hoesl and Budisa demonstrated this attempt at the example of three model proteins, EGFP, ψ-b* and the thermophilic lipase TTL.^123^ They introduced an amber mutation at position Asn150 in EGFP and installed the photoreactive Phe analog Bpa (Figure 9) at this position. For this, they used the orthogonal BpaRS/*Mj*tRNA_CUA_ pair that had been evolved earlier from Methanocaldococcus jannashii TyrRS/tRNA^Tyr^ by the Schultz group.^302^ At the same time, they replaced the 10 Pro residues of EGFP with 4cFP (Figure 12). They found the fully labeled EGFP N150Bpa­[4cFP] (Table 2) as the main protein species in their protein preparation. However, the stabilizing effect of the full Pro→4cFP replacement reported earlier^216^ was lost in the N150Bpa background. Unfortunately, ψ-b* was not suitable for SCS because a permissive position for the incorporation of Bpa could not be identified. Next, they prepared a D221am amber mutant of TTL and incorporated Bpa at this position using the same orthogonal pair as above. In parallel, the 11 Met residues of the enzyme were exchanged for Nle. Bpa only slightly subdued the activity enhancement that the global Met→Nle exchange provoked (see Section 3.3.5). As the amber readthrough at Asp221 was only partially successful, unlabeled variant protein that was truncated at this position accumulated. TTL D221Bpa­[Nle] (Table 2) carried a C-terminal purification tag which should guarantee the preferential purification of full-length protein. As TTL is a homodimer, however, the truncated variant could heterodimerize with the full-length protein and was copurified. Despite this complication, the preparation of a highly active enzyme (Met→Nle, SPI) containing a photoreactive handle (D221Bpa, SCS) demonstrates very impressively the synergistic effects of a combination of SPI with SCS.^123^

Virtually at the same time, Yun and co-workers furnished GFP with Dopa (Figure 10) and Aha or Hpg (Figure 7) in a combined SCS/SPI experiment.^118^ Using the *Mj*DhpRS/*Mj*tRNA_CUA_ pair described by Alfonta et al.,^197^ they introduced Dopa at position Y66am in the fluorophore of GFP. The five Met residues of GFP Y66am were replaced with Aha or Hpg by SPI at the same time. GFP Y66Dopa­[Hpg] (Table 2) showed slightly red-shifted excitation and emission spectra relative to the wild-type GFP. This was due to the electron donating effect of an additional hydroxyl group at position Y66, which alters the fluorophore chemistry. The alkyne groups of Hpg were selectively conjugated to an azide-fluorophore by CuAAC. The versatility of the combined SCS/SPI experiment was further demonstrated by the production of GFP Y66Dopa­[Aha] under the same conditions. Again, the installation of reactive handles (Met→Aha/Hpg by SPI) was combined with a modulation of the fluorescence properties of the model protein (Y66Dopa, SCS).^118^

Next, the Yun group applied the coupled SPI/SCS method to modify a target protein with multiple functionalities at the same time. Again, they chose GFP as the target protein and introduced the K15am mutation at a solvent-exposed position. The variants GFP­[4cFP], GFP K15Dopa, GFP K15Dopa­[4cFP], GFP­[Hpg] and GFP K15Dopa­[Hpg] were produced by SPI of Hpg or 4cFP in the Met or Pro auxotrophic E. coli strains B834­(DE3) and JM83, respectively. For the site-specific incorporation of Dopa by SCS, the orthogonal *Mj*DhpRS/*Mj*tRNA_CUA_ pair was coexpressed. The individual modifications complemented each other and provoked a synergistic effect: 4cFP increased the stability of GFP, while Dopa allowed the selective immobilization on chitosan or polystyrene beads by periodate oxidation and Hpg facilitated the modification with PEG or a fluorophore *via* CuAAC. Notably, the GFP K15Dopa­[4cFP] retained its excellent folding properties while the dually labeled EGFP N150Bpa­[4cFP] prepared by the Budisa group lost it^123^ (see above).^303^

In a follow-up study, Yun and colleagues made full use of the power of the strategy to stabilize an enzyme and immobilize it onto a support.^304^ They turned their attention to the ω-transaminase (ω-TA) from Sphaerobacter thermophilus. To assess the effect of fluoroprolines on the protein stability, they introduced the Pro analogs 4cFP and 4tFP into ω-TA (13 Pro residues) recombinantly expressed in the Pro auxotrophic E. coli strain BL21­(DE3)­pLysS (KC1325). While ω-TA­[4tFP] was soluble, the incorporation of 4cFP drove the protein into insolubility, thus, 4cFP was not further pursued. Next, they generated the ω-TA R23am mutant for the installation of a reactive handle for directed immobilization. They performed a coupled SPI/SCS experiment with 4tFP and Dopa, whose incorporation was again driven by the *Mj*DhpRS/*Mj*tRNA_CUA_ pair. In comparison to the parent protein or ω-TA R23Dopa, the fluorination of the Pro residues of ω-TA­[4tFP] and ω-TA­[4tFP] R23Dopa increased their thermostability 1.5–2-fold and the fluoro-variants also tolerated a panel of organic solvents better. The Dopa variants ω-TA R23Dopa and ω-TA­[4tFP] R23Dopa were successfully immobilized on chitosan and polystyrene beads by periodate-mediated oxidation, on the latter their catalytic activity survived 10 cycles of reuse.

#### 4.1.6 Adaptive Laboratory Evolution to Addict the Host to an ncAA

ALE is a powerful tool to reshape bacterial proteomes until they integrate ncAAs such that the resulting mutant microorganisms can propagate on them (Figure 15). This has been impressively shown for the proteome-wide incorporation of fluorinated Trp analogs in bacteria.^305–308^ For B. subtilis strain QB928, Trp is essential for growth, the strain does not grow on 4FW, 5FW, or 6FW (Figure 11) as the sole indole amino acid. When strain QB928 was grown on medium containing 4FW instead of Trp, strain LC33 emerged spontaneously by its ability to form colonies on this medium. Strain LC33 grew on Trp as well as 4FW.^305^ 4FW was incorporated at all instances in the proteome and supported cell propagation.^307^ Selection of spontaneous mutants on medium supplemented with fluorotryptophan retrieved the LC33 descendant strain LC62, which propagated on 6FW in addition to Trp and 4FW. The mutant offspring of LC62, strains LC75 and LC79, propagated on 5FW in the presence of decreasing Trp levels. Eventually, strain LC88 emerged, which was able to grow on either Trp, 4FW, 5FW, or 6FW as the sole indole amino acid.^307^ Chemical mutagenesis of LC33 yielded strain HR15, which grew on 4FW *but not* on Trp nor on the other fluorotryptophans.^305^ Taken together, the serial mutations of LC33 through LC88 expanded the genetic code of these strains for fluorinated Trp analogs while the LC33 to HR15 mutation excluded Trp from the genetic code. The selective adaptation reversed the roles of Trp and 4FW: strain QB928 required Trp for growth, while 4FW could not sustain it. In contrast, strain HR15 propagated on 4FW but neglected Trp. These stunning alterations of the genetic code were brought about by only a few rounds of mutagenesis.

The B. subtilis proteome appears to be rather flexible toward the replacement of Trp by the fluorinated analogs. However, this adaptability extends beyond B. subtilis. For instance, the E. coli strain unColi B7-3 propagates on 4FW without Trp incorporation^309^ and bacteriophage Qβ has demonstrated the ability to adapt to 6FW.^310^ Nevertheless, the proteome-wide incorporation of the Trp analogs can lead to malfunction of one or a few essential proteins. The loss of function caused by the replacement of Trp by its analogs in a small collection of such “analog-sensitive” proteins affects cell viability. The genes encoding the analog-sensitive proteins represent a so-called “oligogenic barrier” to genetic code alteration. Their functional expression requires cAAs and since they are essential, their expression ensures that cell viability is maintained through cAAs. At the same time, their analog sensitivity blocks cell propagation on amino acid analogs and the standard genetic code is preserved. To validate the oligogenic barrier concept, Yu et al. analyzed the genomic alterations of the B. subtilis strains thriving on fluorotryptophan in relation to their parent strain QB928. Their findings corroborated the idea that a small number of genes depends on expression with cAAs and therefore preserves the standard genetic code. The cAA and its analogs define which genes form the oligogenic barrier.^308^

The group of Budisa took the proteome-wide incorporation of ncAAs by ALE to the next level.^202,311^ In their first study performed by Hoesl et al.,^202^ they meticulously controlled the availability of Trp for the cells during ALE. They started the ALE experiment with the E. coli K12 W3110 derivative CY15602 Δ*trpEA2* (Coli Genetic Stock Center strain #7679),^312^ which lacked the entire *trpLEDCBA* operon. This strain did not produce any Trp biosynthetic enzymes and, consequently, was unable to biosynthesize Trp or any of its intermediates.^312,313^ Next, they avoided the use of a Trp analog as such because E. coli cells use several transport systems to take up Trp and its analogs from the medium.^313^ They anticipated that the transport systems might be a prime target for mutagenesis during ALE because a block in the Trp analog uptake would alleviate the stress caused by its misincorporation into the proteome of the cells. The Trp precursor indole, however, diffuses into the cell through the cell membrane and can be taken up without a functional transport system.^314^ To ensure efficient turnover of the indole precursor into the corresponding Trp analog (for details, see Section 5.2.5.1), Hoesl et al. equipped the Trp auxotrophic strain CY15602 Δ*trpEA2* with an episomal plasmid for the expression of the Trp synthase from S. typhimurium. As the Trp analog, they chose [3,2]­Tpa (Figure 11) because it is readily accepted by the E. coli TrpRS as a substrate and Trp synthase efficiently produces it from the precursor β-thieno-[3,2-*b*]­pyrrole ([3,2]­Tp).^315^ The initial condition for the ALE experiment consisted of chemically defined NMM^150^ containing 19 cAAs except Trp, which was supplemented with 25 μM [3,2]­Tp and 1 μM indole. Without the minute supplementation with indole, the cells would not grow. The cells were serially passaged to new shake flask cultures which contained a declining concentration of indole while the [3,2]­Tp concentration was kept constant. After 264 passages, E. coli strain MT20 (Table 1) emerged that grew on glucose mineral medium containing [3,2]­Tp but without cAAs or indole added. [3,2]­Tpa replaced Trp in the entire proteome of this strain and recombinantly expressed EGFP (1 Trp residue) was homogeneously labeled with [3,2]­Tpa.^202^

To reveal the mechanism of adaptation, the group repeated the ALE experiment^316^ with a starting strain whose genotype was attuned more finely to the task. They introduced lesions in the genes for Trp biosynthesis (*trpLEDC*) and degradation (*tnaA* gene encoding tryptophanase) into E. coli MG1655 but left the tryptophan synthase encoded by *trpBA* intact. The resulting strain TUB00 ^311^ (Table 1) was able to take up indole or [3,2]­Tp from the medium and convert it to Trp or [3,2]­Tpa intracellularly. This phenotype allowed the supplementation of the medium in each passage with varying ratios of cAAs, indole and [3,2]­Tp to conduct the ALE. First, TUB00 was evolved systematically to grow without indole, afterward the supplementation with commercial cAAs was abolished to avoid potential contamination with traces of Trp. After approximately 1000 generations, the final strain TUB170 (Table 1) was able to grow in minimal medium supplemented with [3,2]­Tp but without added cAAs or indole. A thorough genomic and proteomic analysis of TUB170 as well as single isolates from earlier phases of the ALE experiment revealed that a wide variety of cellular functions had been altered. Most notably, several alterations suppressed the general stress response. In other words, the exchange of Trp by [3,2]­Tpa had heavily stressed the parent strain TUB00 but the adapted strain TUB170 coped well with it.^317^

Agostini et al. drew on Hoesl’s experience to follow up on the ALE of E. coli for the fluorotryptophans 4FW and 5FW.^311^ They biosynthesized 4FW and 5FW (Figure 11) from the corresponding 4- and 5-fluoroindoles with l-serine by the E. coli tryptophan synthase in their Trp auxotrophic E. coli host (TUB00, Table 1). To adapt the host to the biosynthesized fluorotryptophans, they subjected the cells to ALE by serial dilutions. They grew the cells in synthetic minimal medium containing indole and the fluoroindoles in shake flak cultures. In the late exponential phase, they passaged the cells to fresh medium with a lower indole concentration while the concentrations of fluoroindoles, glucose, ammonium and phosphate were kept constant. They analyzed their fluoroindole preparations to rule out any contamination with trace amounts of indole to prevent the accidental, uncontrolled biosynthesis of Trp. Finally, the synthetic minimal medium did not contain any amino acids and was only supplemented with the fluoroindole precursors. The E. coli strains 4TUB93 and 5TUB83 resulting from the ALE procedure were facultative fluorotryptophan/Trp users. The authors performed genomics, proteomics and metabolomics analyses at early, intermediate and late stages of the ALE. They found that only a single out of >20 000 Trp (TGG) codons was mutated during ALE with 5FW. In general, only a limited set of genes was mutated; important examples include ribosomal proteins, RNA polymerase subunits, TrpRS and the Trp repressor TrpR as well as the multidrug efflux pump. Chaperones and proteases were upregulated, and the growth rate was reduced. The metabolome also changed during ALE, and Trp, biotin, and the lipid metabolites experienced the most notable alterations. The cell membrane became permeable to extracellular solutes to facilitate the uptake of the fluoroindoles.^311^

Very recently, Treiber-Kleinke et al. performed a similar ALE experiment to addict E. coli to 6FW and 7FW (Figure 11). While facultative fluorotryptophan/Trp users evolved again, the adaptation to these regioisomers was more challenging for the E. coli cells than the adaptation to 4FW and 5FW.^318^

The study by Budisa and co-workers represents a hallmark for the systematic analysis of the cellular effects of ncAAs during ALE. A brief comment (“first response”) of Zhang and Ellington^319^ discusses their work^311^ in relation to earlier studies on the proteome-wide incorporation of fluorinated Trp analogs.^305,308–310^

### 4.2 Tuning the Properties of Protein-Based Biomaterials

Proteins are genetically encoded amino acid polymers that are assembled from defined building blocks by ribosomal translation. They are monodisperse polyamides and their secondary structure elements are well defined. Although the amino acid side chain chemistry is prescribed by the genetic code, it can be expanded with ncAAs. Being fully biodegradable, proteins blend seamlessly into the natural cycle. These characteristics render proteins an excellent alternative to synthetic polymers for the fabrication of materials. Proteins are also nature’s preferred construction material for the manufacture of functional biomaterials such as hair, fur, silk, horn, bone, teeth, muscles, tendons, and bioadhesives. The proteins constituting these biomaterials, for instance, keratin, collagen, spider- and silkworm-silk fibroins, elastin, suckerin or resilin have inspired and advanced materials science.^320^ ncAAs can augment their appealing natural properties by adding further functionalities not found in nature (Figure 15). ncAAs with bioorthogonal reactive side chain moieties such azide or alkyne groups or fluorinated amino acid analogs allow the precise, genetically encoded manipulation of the proteins’ structural, mechanical and chemical properties and thus of the biomaterials which they constitute. Precisely patterned attachment of drugs, sugars, lipids, nucleic acids, peptides or other polymers holds enormous promise for future applications in tissue engineering, drug delivery, diagnostics, medical therapy, biosensors, nanobiotechnology and textiles.^18^ Here, we focus on the labeling of biomaterials with ncAAs by SPI in auxotrophic organisms. Israeli et al. recently reviewed the multisite incorporation of ncAAs into protein-based biomaterials by genetic code expansion using orthogonal translation systems and genetically recoded organisms.^321^

#### 4.2.1 Functionalization and Fluorination of the Elastic Scaffold Protein Elastin

The structural protein elastin confers elasticity to vertebrate skin, cardiovascular tissue or cartilage among others. Elastin is a protein block polymer consisting of alternating hydrophobic and cross-linking blocks. The hydrophobic block is composed of tetra- to hexapeptides rich in Val, Pro, and Gly residues.^320^ Urry and co-workers showed that polymers of the synthetic Val-Pro-Gly-Val-Pro (VPGVP) pentamer behave similarly to natural elastin. They undergo a phase transition when heated, i.e., they coacervate and form supramolecular filaments.^322^ Elastin-like polypeptides (ELPs) containing the repetitive VPGXG pentamer unit, where X can be any amino acid except proline, have been used to compose stimuli-responsive biomaterials. The nature of the amino acid X as well as the length *n* of (VPGXG)_*n*_ polypentamers modulate the physicochemical properties of these materials (see the recent review by Guo et al.^323^). (VPGXG)_*n*_ polypentamers are readily expressed in recombinant hosts such E. coli.^324^

Teeuwen et al. demonstrated the successful bioorthogonal functionalization of an ELP with fluorophores, a synthetic polymer and an enzyme.^49^ The ELP was composed of 90 VPGXG pentamers fused to an N-terminal purification tag. To introduce reactive handles into the ELP, they replaced two Met residues in the N-terminal domain with Aha and Hpg by SPI in the Met auxotrophic E. coli strain B834­(DE3)­pLysS (Table 1). ELP­[Aha] and ELP­[Hpg] were then conjugated to fluorescent probes carrying compatible alkyne- and azide moieties by CuAAC. To generate an ELP-polymer hybrid, they attached azido-PEG2000 to ELP­[Hpg]. Furthermore, they immobilized azido-functionalized CalB^47^ on ELP­[Hpg] and confirmed that the conjugated enzyme retained its hydrolytic activity.^49^

While Teeuwen et al. modified a soluble ELP, Zhang et al. generated an artificial ELP scaffold for directed protein immobilization.^265^ The artificial peptide scaffold consisted of a surface anchor and a protein capture domain, which were separated by a short peptide linker to avoid steric hindrance. An ELP of the sequence [(VPGVG)_2_­VPG**F**G­(VPGVG)_2_]_5_VPGC (E_5GC_) comprised the surface anchor and the protein capture domain consisted of a parallel heterodimeric leucine zipper pair. The leucine zipper pair was formed of tightly interacting acidic ZE and basic ZR subunits that much more preferentially heterodimerized (∼9 orders of magnitude) than homodimerized.^325^ The ZE subunit was fused to the E_5GC_ and ZR to the target protein as an affinity tag for capture. To immobilize the ZE-E_5GC_ on a solid support, the authors exchanged the five Phe residues in the E_5GC_ sequence with 4azF (Figure 9) by SPI in the Phe auxotrophic E. coli strain AF-IQ (Table 1). Since 4azF is not a substrate for wild-type *Ec*PheRS, they co-overexpressed the mutant *Ec*PheRS^A294G^ with relaxed substrate specificity,^263^ which resulted in 45% incorporation efficiency of 4azF. ZE-E_5GC_[4azF] (Table 2) was then spin-coated onto silanized glass slides and covalently linked to the surface by irradiation of the photoreactive arylazide moieties with UV light. The model proteins GFP and GST carrying a C-terminal ZR affinity tag were spotted onto the ZE-E_5GC_ scaffold. The dimerization of the ZE subunit on the scaffold and the ZR subunit on the proteins promoted their directed immobilization on the solid surface. The approach was so specific that the ZR-tagged model proteins could be immobilized directly from crude cell extracts (Figure 15). Moreover, the leucine zipper interaction was stable under denaturing conditions in 8 M urea and in a pH range from 4 to 8. Due to its specificity and robustness, ZE-E_5GC_ scaffold could be used, for instance, for the high-throughput manufacture of protein arrays.^265^

Carrico et al. used a similar design to generate an artificial extracellular matrix protein.^67^ Instead of a ZE domain, they fused a cell adhesion domain (A; YAVTG­RGD­SPA­SSK­PIA) to the N-terminus of an E_5_ triplicate ((E_5_)_3_, [[(VPGVG)_2_­VPG**F**G­(VPGVG)_2_]_5_­VP]_3_LE). To support the adhesion of mammalian cells, the fibronectin derived adhesion domain contained an RGD motif.^326^ The 15 Phe residues in the fusion protein A_RGD_-(E_5_)_3_ were exchanged for 4azF by SPI as described above for ZE-E_5GC_. A_RGD_-(E_5_)_3_[4azF] was obtained at a titer of 40 mg/L and the incorporation efficiency reached a maximum of 50% when the Phe auxotrophic E. coli expression host AF-IQ was supplemented with 250 mg/L 4azF. To demonstrate cell adhesion to the artificial extracellular matrix protein, A_RGD_-(E_5_)_3_[4azF] protein films were spin-coated onto poly­(ethylene oxide)-coated glass slides and irradiated with UV light through a photomask. Unbound protein in the masked areas was stripped with 6 M guanidinium hydrochloride. Afterward, Rat-1 fibroblasts were deposited on the RGD patterns in serum-free medium, allowed to adhere for several hours and then the slides were rinsed with buffer to remove nonadhering cells. The cells adhered only to the patterns that contained the RGD motif but not outside of them.^67^ To study the elastic properties of the artificial matrix protein in more detail, Nowatzki and co-workers^91^ produced an analogous fusion protein that contained the CS5 binding motif^327^ (GEE­IQI­GHIP­RED­VDYH­LYPG) instead of RGD. Following the same procedure for the exchange of the 15 Phe residues of A_CS5_-(E_5_)_3_ with 4azF as Carrico et al., they obtained a somewhat higher titer (66 mg/L with a supplementation of 250 mg/L 4azF) and a better incorporation efficiency (66%) for A_CS5_-(E_5_)_3_[4azF]. They generated thin films of A_CS5_-(E_5_)_3_[4azF] with a controlled elastic modulus by exposing the films containing the photoreactive 4azF residues to different UV radiation doses. Otherwise, they varied the 4azF content of the protein preparation by supplementing the Phe auxotrophic expression host with selected concentrations of 4azF. The higher the 4azF content or the radiation dose, i.e., the longer the irradiation time, the more cross-links were formed in the protein network. The elastic modulus increased accordingly, indicating that the cross-linking stiffened the biomaterial.

Ta et al. advanced the ELP scaffolding concept further and garnished the oriented immobilization of ELPs with additional functions.^50^ Their ELP was composed of 90 pentapeptide repeats, nine of which contained a single Met residue ([VPG**M**G­(VPGVG)_7_­(VPGEG)_2_]_9_; E_9_). They fused a ybbR and sortase A tag to the N- and C-termini of E_9_. Catalyzed by the enzyme 4′-phosphopantetheinyl transferase (Sfp), the N-terminal ybbR tag binds to coenzyme A (CoA)^328^ while the C-terminal LPETGG tag can be used for sortase A-mediated ligation.^329^ To complete the panel of mutually compatible derivatization options with bioorthogonal chemistry, they replaced the nine Met residues in the E_9_ sequence with Aha. The Met auxotrophic E. coli strain RF11 (Table 1) produced ybbR-E_9_[Aha]-LPETGG at comparable titers as the parent protein (Table 2) and Met was quantitatively replaced with Aha. To generate a biosensor surface, Ta et al. immobilized ybbR-E_9_[Aha]-LPETGG on CoA-coated microwell plates *via* its ybbR tag. They furnished an anti-mCherry single-domain antibody (SdAb) with a DBCO group and attached it to the immobilized ybbR-E_9_[Aha]-LPETGG by SpAAC. In comparison to randomly surface-adsorbed anti-mCherry SdAb, the scaffolded SdAb biosensor detected mCherry faster and with higher sensitivity even in a complex matrix such as human plasma. Finally, they labeled ybbR-E_9_[Aha]-LPETGG with two different fluorophores at the N- and C-termini by the Sfp- and sortase A-catalyzed ligation reactions and attached anti-mCherry SdAb to the azide-moieties by SpAAC. The resulting dual-fluorescence labeled SdAb sensor was then employed to selectively sort cells presenting mCherry on their surface by flow cytometry. This study impressively demonstrates that a combination of genetically encoded bioorthogonal (Aha) and enzymatic tags (ybbR and sortase A tag) can endow multiprotein complexes with precisely positioned functions, designed spacing and controlled valency.

In addition to attaching functional domains such as leucine zippers or ligation tags to ELPs, they can also be combined with other self-assembling polypeptides. Artificial protein block copolymers of distinct alternating self-assembling domains allow the design of entirely new biomaterials. The group of Montclare devised block copolymers consisting of an ELP (E_5_, [(VPGVG)_2_­VPG**F**G­(VPGVG)_2_]_5_­VP) and the coiled-coil domain of cartilage oligomeric matrix protein (C, DLA­PQM­LRE­LQE­TNA­ALQ­DVR­ELL­RQQ­VKE­IT**F**­LKN­TVM­ESD­ASG).^330^ They expressed the diblocks E_5_C and CE_5_ containing 6 Phe residues and a triblock E_5_CE_5_ (11 Phe) with 4FF (Figure 9) in the Phe auxotrophic strain AF-IQ as described earlier.^105^ All variants were produced at ∼5 mg/L and 4FF was incorporated at very high efficiency, 80–90% in blockE_5_C­[4FF], 78–87% in blockCE_5_[4FF], and 89–94% in blockE_5_CE_5_[4FF] (Table 2). The values for the incorporation efficiencies varied depending on whether amino acid analysis or matrix-assisted laser desorption/ionization-time-of-flight (MALDI-TOF) mass spectrometry were employed for the assessment. The fluorinated diblocks’ structure was very similar to that of the parent proteins containing Phe. In contrast, the secondary structure of blockE_5_CE_5_[4FF] resembled the parent at low temperatures but deviated at higher temperatures where it adopted a β-rich conformation. As a result of the varying number and order of the polypeptide blocks, the unmodified CE_5_, E_5_C, and E_5_CE_5_ block copolymers underwent supramolecular transition at different temperatures. In comparison, the blockE_5_C­[4FF] and blockE_5_CE_5_[4FF] proteins transitioned at the same temperature as their corresponding parent proteins, yet the transition of blockE_5_CE_5_[4FF] experienced an enhanced cooperativity. In contrast, fluorination lowered the transition temperature of blockCE_5_[4FF] by 8 °C relative to its parent. The fluorination increased the elastic character of all three fluorinated block variants. In summary, fluorination represents an “atomic instrument” to manipulate structure, temperature responsiveness, supramolecular assembly and mechanical properties of ELPs and their block copolymers.^113^

The same group used fluorinated ELP block copolymer micelles for drug delivery (Figure 15).^331^ They fused a C domain (sequence see above, 7 Leu residues) to a shorter E domain (E_2_, [(VPGVG)_2_­VPGFG­(VPGVG)_2_]_2_­VP) to generate the protein block polymer blockCE_2_. BlockCE_2_ self-assembled into micelles in a concentration- and temperature-dependent manner. To make the micelles visible in ^19^F magnetic resonance imaging and -spectroscopy (^19^F MRI/MRS), Montclare and co-workers replaced the eleven Leu residues in the C block by Tfl (Figure 13). Since E_2_ did not contain Leu residues, the elastin-like domain of the block polymer remained unaltered. They produced ∼30 mg/L blockCE_2_[Tfl] (Table 2) in the Leu auxotrophic E. coli strain LAM1000 (Table 1), which amounted to approximately 60% of the parent protein with Leu. Tfl was incorporated at ∼81% efficiency. Fluorinated blockCE_2_[Tfl] assembled into micelles where the hydrophobic, thermoresponsive non-fluorinated ELP core was surrounded by a corona of fluorinated coiled-coil pentamers. When C domains assemble into coiled-coil homopentamers, a hydrophobic pore is formed that can encapsulate small hydrophobic molecules.^332^ The authors exploited this asset of the C domain to charge the protein block copolymer micelles with the anticancer drug doxorubicin (Figure 15). To administer the drug to cultured human breast cancer cells, they triggered its release under hyperthermic conditions, which induced the thermoresponsive coacervation of the ELP core domains. In addition to its therapeutic effect, blockCE_2_[Tfl] served as an excellent probe to detect tumors in a breast cancer xenograft mouse model by ^19^F MRI/MRS. In summary, blockCE_2_[Tfl] is a promising therapeutic and diagnostic, i.e., a theranostic agent.

#### 4.2.2 Natural and Recombinant Fiber Proteins Redesigned with Bioorthogonal Functional Groups

Silk is a filamentous protein biomaterial that is produced by many terrestrial and aquatic organisms for purposes as diverse as metamorphosis, shelter, reproduction or catching prey.^333,334^ The silk from the mulberry silkworm Bombyx mori and spider dragline silk have received the most attention in biotechnology so far because of their fascinating material properties such as high tensile strength and toughness.^334^ Silks have a highly hierarchical structure (Figure 18). For instance, mulberry raw silk consists of two parallel fibroin monofilaments that are wrapped in an outer layer of sericin, the “silk gum”. Each monofilament is composed of microfibril bundles consisting of fibroin nanofibrils. The nanofibrils are assembled from fibroin molecules that interconnect *via* their termini. The fibroin molecule itself is a heterodimer of the light chain fibroin FibL (26 kDa, hydrophilic) and the 15 times larger heavy chain fibroin FibH (390 kDa), which are connected by a disulfide-bond. The modular primary sequence architecture of FibH determines the mechanical properties of the silk. Hydrophilic, nonrepetitive N- and C-termini flank highly repetitive hydrophobic poly­(GA) domains that self-assemble into β-sheet crystallites. During fibrillation, the β-sheet crystallites are embedded into more amorphous linker regions.^334–336^ The mechanical properties of silk are influenced by the silkworm diet.^337^

**Figure 18:**
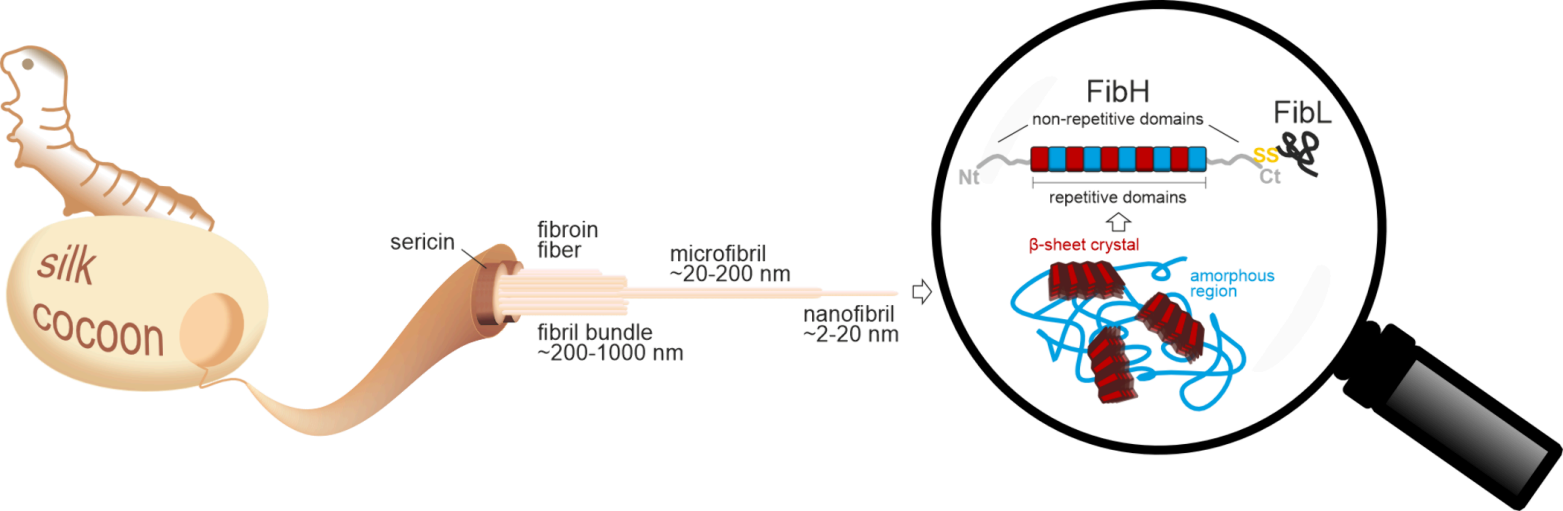
Hierarchical structure of silkworm silk. FibH, heavy chain fibroin; FibL, light chain fibroin; SS, disulfide bond, Nt, amino terminus; Ct, carboxy terminus.

Spider silk proteins (spidroins) have a very similar modular architecture. In the spidroins of spider dragline silk, β-sheet crystallites forming hydrophobic poly­(A) repeats alternate with GGYGPG and GPGQQ motifs that constitute amorphous regions.^334,338^ Their biocompatibility and biodegradability predestines silks not only for biomedical applications, but also for cosmetics, textiles and technical applications.^18,320,339–343^

Harvey et al. first reported the site selective modification of the minimal spidroin motif protein 4RepCT.^75^ Johansson and co-workers had shown before that 4RepCT can be expressed in soluble form in E. coli as a C-terminal fusion to a thioredoxin (TRX) tag.^344^ Proteolytic cleavage of the N-terminal fusion tag provoked spontaneous formation of macroscopic fibers.^345,346^ To introduce reactive azide handles into 4RepCT, Harvey and co-workers expressed the TRX-4RepCT fusion protein with Aha (Figure 7) in the Met auxotrophic E. coli strain DL41 (Table 1). They confirmed the presence and accessibility of the Aha residues in the nonrepetitive C-terminal domain by CuAAC with alkyne-fluorophores. After cleavage of the TRX tag, 4RepCT­[Aha] (Table 2) assembled into fibers. The preassembled fibers were decorated with the antibiotic levofloxacin: levofloxacin was functionalized with an alkyne-functionalized acid-labile linker, which allowed its conjugation with the azide fibers *via* CuAAC (Figure 15). Toxic Cu­(I) ions could be effectively contained by using the Cu­(I) ligand tris­(3-hydroxypropyltriazolylmethyl)­amine (THPTA) in the bioconjugation reaction. Toxic Cu­(II) ions were captured with ethylenediaminetetraacetic acid (EDTA) and washed off the functionalized fibers. After this treatment, the fibers did not contain any detectable traces of copper, which is particularly important for potential medical applications. The acid-labile linker caused a sustainable release of the antibiotic over a period of five days. The slow sustained release could be useful for wound dressings and drug delivery, when the persistent supply of a therapeutic agent is desired.^75^

In a follow-up study,^347^ the same group decorated soluble TRX-4RepCT­[Aha] (Table 2) and 4RepCT­[Aha] fibers with alkyne-functionalized cyclopeptides containing an RGD cell adhesion motif by CuAAC and SpAAC. Soluble RGD-functionalized TRX-4RepCT­[Aha] protein was cast into films, which were seeded with human mesenchymal stem cells. The cells adhered and grew in an RGD-dependent manner. Importantly, the RGD-functionalized TRX-4RepCT­[Aha] films tolerated sterilization in 70% (v/v) ethanol or by autoclaving.^347^

While the assets of spidroins functionalized with ncAAs have just begun to emerge, attempts to label silkworm silk with reactive ncAAs commenced more than a decade ago. Teramoto and co-workers embarked on the endeavor to introduce reactive Phe analogs into silkworm silk by supplying the analogs in the animals’ diet. Basically, they intended to perform an SPI experiment in a living animal. They chose to work with Phe analogs because Phe is an essential amino acid in the silkworm Bombyx mori.^348^ As it is supplied in the diet, its content can be controlled. B. mori PheRS is well studied, and Phe analogs are commercially available. Most importantly, Phe occurs only in the noncrystalline regions of the silk fibroin molecules. Consequently, their replacement by analogs was expected to have a minimal impact on fiber formation.^349^

At first, Teramoto et al. devised a *Bm*PheRS^αA450G^ mutant with relaxed substrate specificity toward Phe analogs with substituents in the *para*-position (C4).^350^ *Bm*PheRS is an (αβ)_2_ heterotetramer and the A450G mutation was introduced into the α-subunit. They gleaned this position from reports in the literature where the E. coli PheRS’s substrate tolerance had been relaxed by introducing the corresponding A294G mutation.^260^ *Bm*PheRS^αA450G^ aminoacylated tRNA^Phe^ with 4FF, 4-chloro-l-phenylalanine (4ClF) and 4-bromo-l-phenylalanine (4BrF), but not with 4IF (Figure 9) *in vitro*.^266^ When they expressed *Bm*PheRS^αA450G^ in ovary-derived cultured B. mori cells (BmN cells), 4ClF was incorporated into the reporter protein EGFP at very low levels (∼5% incorporation efficiency). They were unable to detect the incorporation of 4BrF. The growth medium contained serum and obviously, the included Phe outcompeted the analogs during translation.

Next, Teramoto and Kojima compared the tolerance of several *Bm*PheRS mutants for *para*-substituted Phe analogs.^351^ Of the three examined *Bm*PheRS mutants, *Bm*PheRS^αT407G^ accepted 4ClF, 4BrF, 4IF and 4azF (Figure 9) as substrates for the aminoacylation of tRNA^Phe^ *in vitro*. *Bm*PheRS^αT407A^ showed a comparable substrate profile, but it weakly aminoacylated 4IF and tolerated 4azF less than *Bm*PheRS^αT407G^. *Bm*PheRS^αA450G^ showed the least substrate tolerance aminoacylating 4ClF and 4BrF but 4azF and 4IF only negligibly. 4-Cyano-l-phenylalanine (4CNF) (Figure 9) was a weak substrate for all three mutants and 4-amino-l-phenylalanine (4NF) (Figure 9) was not accepted at all.^351^

The recombinant expression of *Bm*PheRS mutants with relaxed substrate tolerance could result in cytotoxicity caused by the augmented misincorporation of cAAs throughout the proteome. To assess their cytotoxicity, the three *Bm*PheRS mutants were expressed in cultured BmN cells under standard and 9-fold lower Phe supplementation in the medium. Cytotoxicity was judged by the expression level of EGFP as the reporter and it increased with the PheRS’s substrate tolerance. Expression of *Bm*PheRS^αA450G^, the mutant with the lowest substrate tolerance was not toxic for the BmN cells under any Phe condition. While *Bm*PheRS^αT407A^ was cytotoxic at low Phe levels, *Bm*PheRS^αT407G^ drastically decreased the EGFP expression level, independent of the Phe condition. This behavior clearly disqualified the latter mutant for further incorporation experiments, which were conducted with *Bm*PheRS^αA450G^ and *Bm*PheRS^αT407A^. At low Phe supplementation of the BmN cells, the *Bm*PheRS^αA450G^ mutant promoted the incorporation of 4BrF, 4CNF, 4azF and 4IF into EGFP in addition to 4ClF, which had been demonstrated earlier.^350^ 4BrF was also incorporated by the *Bm*PheRS^αT407A^ mutant under standard Phe conditions.^351^

These findings paved the way to the generation of the transgenic B. mori line H01 that expressed *Bm*PheRS^αA450G^ specifically in the posterior silk glands,^349^ where silk fibroin is produced.^352^ The transgenic animals were fed a synthetic diet containing Phe and the analogs 4ClF or 4BrF. 4ClF did not affect larval growth but it reduced the fibroin production in wild-type (∼30% less) and to a higher extent in transgenic animals (∼60% less). Nevertheless, 4ClF partially replaced Phe in the silk fibroin (*Bm*Silk­[4ClF], Table 2). The properties of the 4ClF/Phe silk were largely unaltered in comparison to wild-type silk containing exclusively Phe.^353^ Transgenic B. mori also incorporated 4BrF into their silk (*Bm*Silk­[4BrF], Table 2). The incorporation efficiency was noticeably increased when the Phe content of their diet was reduced to 50% (w/w). However, lowering the Phe in the diet also decreased the silk production. Most notably, 4azF was also incorporated into the silk, albeit in contrast to 4ClF and 4BrF only under drastically reduced Phe conditions. Negligible incorporation of 4azF occurred in silk produced by animals that were fed with 4azF contained in the standard synthetic diet with Phe. When the Phe content was lowered to 20% of the standard, the transgenic larvae grew slowly, and their fibroin production was extremely low. They produced ∼5 mg fibroin/larva, which was seven times less than the output of wild-type B. mori under the same conditions. The incorporation efficiency of 4azF was 1.5%. FibH in the silk fibroin heterodimer contains 28 Phe residues and FibL only seven. If 1.5% of a total of 35 Phe residues in fibroin were replaced by 4azF, on average every second fibroin heterodimer contained a single 4azF molecule. The tradeoff between 4azF incorporation efficiency and larvae growth as well as fibroin output culminated in an optimum dosage of 0.15 mol equivalents of 4azF relative to the standard Phe content of the diet, and a reduction of the Phe content to 30% of the standard. Despite the low incorporation efficiency, the azido-labeled silkworm silk, aka AzidoSilk (*Bm*Silk­[4azF], Table 2) produced by line H01 was successfully functionalized with an alkyne-fluorophore (Figure 15). The azide groups survived the alkali-treatment to remove the sericin gum of the silk but were susceptible to photolysis by light.^349^

To improve the 4azF incorporation, Teramoto and co-workers generated B. mori transgenic line H03 that expressed the *Bm*PheRS^αT407A^ mutant in the posterior silk glands.^354^ As shown in the earlier study outlined above,^351^ this mutant displayed a more relaxed substrate tolerance than *Bm*PheRS^αA450G^ of transgenic line H01. The expression levels of both *Bm*PheRS mutants were comparable in the lines H01 and H03. Both transgenic lines produced less fibroin when fed with 4azF, although the effect was more pronounced in H03. While the incorporation of 4azF was very low in line H01, H03 produced AzidoSilk on a synthetic diet containing standard amounts of Phe and 4azF, or even when fed a commercial mulberry leaf-based diet supplemented with 4azF. However, an increasing 4azF supplementation caused a decline in fibroin production. Titration experiments with 4azF in the commercial diet revealed that 0.5% (w/w) 4azF was the optimal supplement. When reared on this diet, line H03 was able to double the incorporation efficiency of 4azF in comparison to the earlier study.^349^ This means, that the improved transgenic line produced AzidoSilk where each fibroin heterodimer contained on average one 4azF molecule. The mechanical properties of the AzidoSilk from H03 were very similar to wild-type silk. Fibers, films and porous sponges fabricated from this silk were bioorthogonally labeled with fluorophores, biotin and DBCO-functionalized GFP. Employing photomasks, Teramoto et al. were able to control the photolysis of the arylazides in the silk and achieve photolithographic patterning of AzidoSilk films with GFP.^354^

The *Bm*PheRS^αT407G^ and *Bm*PheRS^αT407A A450G^ mutants displayed an even more relaxed substrate specificity than the mutants expressed in lines H01 and H03. However, they mis-charged tRNA^Phe^ with Trp, which led to the misincorporation of Trp at Phe sites. Consequently, the expression of these *Bm*PheRS mutants in transgenic B. mori lines caused a degeneration of the posterior silk glands, the organs for the production of the fibroin and thus a reduction in silk output.^355^ On the other hand, the transgenic B. mori line H06, which expressed the new mutant *Bm*PheRS^αF432V^ emerged as the AzidoSilk production champion.^356^ It combined a substantially improved incorporation efficiency of 6% with a fibroin productivity of 102 mg fibroin/larva, which approached that of the wild type. The excellent performance of the *Bm*PheRS^αF432V^ permitted a 10-fold reduction of the 4azF content in the commercial diet relative to H03.^356^ To upscale the AzidoSilk production, Teramoto and co-workers crossed the transgenic line H06 with the high silk-producing strain Nichi509 × Nichi510. The resulting F1 hybrid H06 × (Nichi509 × Nichi510) produced 1.5-fold more fibroin, yet the incorporation efficiency (6.6%) did not change grossly. The cocoons of 1000 F1 hybrid silkworms yielded 160 g of AzidoSilk raw silk fiber. To demonstrate the availability and accessibility of the azido-moieties for modification, the silk fibers were decorated with green and red fluorescing alkyne-fluorophores using SpAAC.^357^ Interbreeding of transgenic line H06 with the high-silk producer line MCS4 yielded the transgenic high-silk producer line H06-MCS4, which is homozygous for the *Bm*PheRS^αF432V^ mutant. While the cocoon weight increased by 25% relative to H06, the 4azF incorporation efficiency was lowered by ∼20%. Regarding their tensile properties, i.e., maximum strength, maximum strain, Young’s modulus and toughness, AzidoSilk raw silk fibers spun by line H06-MCS4 were equivalent to raw silk fibers from line MCS4.^358^

AzidoSilk produced by transgenic B. mori line H03 with the *Bm*PheRS^αT407A^ mutant^354^ was not very efficiently cross-linked with a bifunctional (DBCO)_2_-PEG_4_ linker, but the cross-linking could be improved by a longer linker molecule.^359^ On the other hand, transgenic line H06 expressing *Bm*PheRS^αF432V^ produced fibroin with more azide groups per fibroin molecule, which improved the cross-linking efficiency as well.^360^ Teramoto and co-workers demonstrated that the PEGylation of silk fibroin films in combination with photopatterning can be used to generate silk-based substrates for controlled cell adhesion.^361^ Films of AzidoSilk from B. mori line H06 were irradiated with 254 nm UV light through photomasks to provoke selective photolithographic decomposition of the arylazide groups. The remaining azide groups were then functionalized by SpAAC with DBCO-functionalized methylated PEG. On such photopatterned surfaces, cultured fibroblasts could only grow in zones free of PEG residues. This finding confirmed that the azido groups of AzidoSilk do not interfere with the biocompatibility of the material, which is an important asset for putative medical applications, e.g., in tissue engineering.^361^

To gain better control over the photolysis of the reactive groups on the silk, Teramoto et al. switched to the analog 4yF (Figure 9), which is more photostable than 4azF. The *Bm*PheRS^αA450G^ and *Bm*PheRS^αT407A^ mutants expressed in the transgenic B. mori lines H01 and H03 had been shown to accept a variety of Phe analogs *in vitro* ^351^ and *in vivo* (see above), but 4yF had not been tested. When fed to wild-type, H01 and H03 larvae, 4yF retarded their growth and severely reduced the fibroin production. Nevertheless, H01 and H03 incorporated 4yF into their silks, line H03 being approximately three times more efficient than line H01. The new AlkyneSilk (*Bm*Silk­[4yF], Table 2) was processed into films, which were successfully labeled with an azide-fluorophore and azide-PEG-biotin by CuAAC. The photostability of the alkyne-groups in the silk material was confirmed by irradiation with UV light at 254 nm for 1.5 min. Under the same conditions, the azide-groups of AzidoSilk photolyzed. The improved photostability of AlkyneSilk would allow its convenient handling under standard lighting conditions, e.g., in a silk reeling factory.^362^

The engineering of silkworm silk is not limited to 4azF and the other Phe analogs listed further above. The Teramoto group has recently drawn their attention to other reactive ncAAs such as the tyrosine analog 3-azido-l-tyrosine (3azY) (Figure 10). Tyr is much more abundant in silk fibroin than Phe. The FibH/FibL heterodimer contains 271 Tyr residues, which constitute ∼5 mol% of the entire sequence. As such, Tyr is roughly seven times more abundant than Phe. Other than Phe, Tyr occurs also in the repetitive domains of the silk fibroin, which form β-sheet crystallites. It may be expected that the replacement of Tyr by its analogs in these domains affects the structure and/or mechanical properties of the protein and possibly also the silk. To test this hypothesis and to increase the frequency of reactive azido groups in the silk, Teramoto et al. generated the transgenic B. mori line H10. This line carries the *Bm*TyrRS^Y36G^ mutant, which was shown to aminoacylate B. mori tRNA^Tyr^ with 3azY. The expression of the mutant *Bm*TyrRS^Y36G^ in the posterior silk glands neither affected larval growth nor fibroin production. The growth of line H10 was independent of the amount of 3azY supplied in its diet, yet the fibroin production decreased with an increasing amount of 3azY. 3azY was incorporated into the silk albeit at low efficiency. Tyr is not an essential amino acid for B. mori,^348^ hence the low incorporation efficiency might be due to the competition of biosynthesized Tyr with 3azY. AzidoSilk­[3azY] (*Bm*Silk­[3azY], Table 2) was sensitive to chemical degumming at alkaline pH and elevated temperature. During the degumming process, the 3azY moieties most probably decomposed and formed highly reactive nitrenes that formed covalent cross-links with nearby residues. This improved the mechanical strength of the silk fibers but rendered them almost insoluble.^363^

Phe residues occur throughout FibH in the domains intervening the β-sheet repeats. In contrast, Met residues are only present in the nonrepetitive N-terminal domain of FibH. FibH contains three Met residues and FibL only one. To avoid potentially adverse effects of the incorporation of a reactive ncAA on fibroin function, Teramoto et al. replaced the four Met residues in silk fibroin with Aha, Hpg and Hag (Figure 7; corresponding *Bm*Silk variants see Table 2).^336^ They showed that *Bm*MetRS aminoacylated tRNA^Met^ efficiently with the three Met analogs *in vitro*. Like Phe, Met is an essential amino acid for B. mori^348^ and its content in the diet had to be lowered for of all three Met analogs to replace the four Met residues in fibroin. As expected, lowering the Met content of the diet lowered the fibroin production. Supplementation of the Met-reduced diet with Hag had the least effect on larval growth and fibroin production. Hpg slowed the larval growth and reduced the fibroin production further, and Aha apparently was toxic for B. mori. Nevertheless, mass analysis of the fibroins produced in the presence of the Met analogs revealed their partial incorporation. Hpg and Hag were incorporated at 23% efficiency and Aha at 13%. On average, each fibroin molecule was labeled with 1 molecule of Hpg or Hag, and Aha was incorporated in every second fibroin molecule. CuAAC of the Hpg- and Aha-labeled silk fibroins with biotin-PEG-azide or -alkyne and the subsequent detection of the biotin-moiety with horse radish peroxidase-labeled streptavidin on a Western blot confirmed the incorporation of both Met analogs in FibH and FibL. The incorporation efficiencies were comparably decent because the Met analogs could have been incorporated into proteins biosynthesized anywhere in the body of the silkworm larvae. In contrast to the Phe analogs, whose incorporation most probably occurred mainly in proteins biosynthesized in the posterior silk glands where the mutant PheRSs were expressed, the Met analogs were substrates of the endogenous *Bm*MetRS. The misincorporation of Hpg, Aha or Hag into proteins other than fibroin not only reduced the availability of the ncAAs for the silk biosynthesis but could also have had toxic side effects on other organs.^336^

In summary, minimal spidroin motif proteins and silkworm fibroins have been successfully labeled with reactive ncAAs in a recombinant and the natural host, respectively (Figure 15). The resulting materials were molded into fibers, films and sponges that could be selectively modified with fluorophores, biotin, PEG or cell adhesion motifs. The enhancement of the fascinating physicochemical and mechanical properties of the silk materials with new functionalities holds great promise for future applications, for instance in tissue engineering or drug delivery.

The SPI of reactive ncAAs such as Aha in E. coli has become an established method to genetically encode the precise localization, spacing or valency of the bioorthogonal groups. To achieve a comparable control in a complex expression host such as the silkworm appears by far more challenging. In a microorganism such as E. coli, a single cell has to cope with the peculiarities of the non-natural building block; in the silkworm the posterior silk gland alone counts more than 500 cells.^364^ Single cells “simply” take up the ncAA from the medium while the silkworm larva consumes it with its food and has to deliver it from the gut to the posterior silk gland where it can participate in the ribosomal translation of the fibroins. Considering the complex process of fibroin biosynthesis in the posterior silk gland^352^ and the fact that ncAAs must accumulate for their efficient charging onto tRNA(s),^365^ the incorporation of 4azF and Aha or Hpg worked amazingly well in B. mori. In this regard, the pioneering works of Teramoto and co-workers represent a hallmark of the systematic improvement of the SPI of reactive ncAAs in living animals.

The Montclare group generated pentameric fluorinated coil-coil proteins that assembled into stable fibers.^366^ The fiber forming coiled-coil proteins Q^367^ and C were derived from the coiled-coil domain of cartilage oligomeric matrix protein. The seven Leu residues in both proteins were replaced with Tfl (Figure 13) at excellent efficiencies (>90%). The fluorinated variant proteins Q­[Tfl] and C­[Tfl] (Table 2) self-assembled into stable fibers, which were able to bind the small molecule curcumin in a metal-dependent manner: Zn^2+^ ions promoted the binding while Ni^2+^ released the small molecule. In contrast, the parent protein containing Leu did not self-assemble into fibers under the same conditions.^366^

#### 4.2.3 Direct Genetic Encoding of Post-translational Modifications in Proteins for Biomaterials

Mussel adhesive proteins (MAPs)^368^ have moved into the focus of material scientists, biotechnologists and synthetic biologists because they demonstrate exciting potential as “wet-adhesive bioglues”. MAP-derived bioadhesives bonding water-rich tissue surfaces promise to satisfy the pressing need for noncytotoxic, biocompatible sealants in bone repair, dental surgery and plastic surgery among other medical applications.^369^

To stay put on their underwater strongholds even in rough surf, mussels secrete adhesive proteins in a plaque region at the tips of their “feet”, the adhesive byssus threads. The MAPs that are involved in the immediate interaction with the solid support are rich in Dopa (Figure 10), which is generated by post-translational hydroxylation of Tyr residues. Dopa plays an important role in the underwater adhesion of the mussel foot, but it is not the sole player in this complex, well-orchestrated process.^184,320^ Dopa contains a reactive catechol group that can be exploited for coordinative cross-linking using Fe^3+^ ions or covalent cross-linking, for instance upon oxidation with NaIO_4_.^184,370^ The introduction of Dopa represents a challenge for the recombinant production of MAPs in prokaryotic hosts such as E. coli that do not perform post-translational modifications. The enzymatic post-translational hydroxylation of the Tyr residues with mushroom tyrosinase is straightforward yet rather inefficient.^371^ The direct genetic encoding of Dopa, for instance by SPI in a Tyr auxotrophic E. coli host has been exploited as an attractive alternative (Figure 15).

In 2011, Ayyadurai et al. first demonstrated the successful residue-specific incorporation of Dopa into GFP by SPI in a Tyr auxotrophic E. coli strain (see Section 3.1.2).^61^ Three years later, the group of Cha applied the approach for the residue-specific incorporation of Dopa into recombinant adhesive mussel foot proteins type 3 (Mgfp-3, 10 Tyr residues) and type 5 (Mgfp-5, 20 Tyr residues) from Mytilus galloprovincialis.^147^ The Dopa variants Mgfp-3­[Dopa] and Mgfp-5­[Dopa] (Table 2) were produced at a titer of 3–5 mg/L and the Dopa content of >90% approached that of the natural MAPs. In comparison, the post-translational enzymatic hydroxylation of Tyr by mushroom tyrosinase was much less efficient (14% for Mgfp-3 and 9% for Mgfp-5). The relatively higher Dopa content allowed Mgfp-3­[Dopa] to stick to hydrophobic surfaces such as polystyrene and polypropylene much more strongly than the enzymatically Tyr-hydroxylated Mgfp-3. The oxidation of the catechol moieties with NaIO_4_ induced strong water resistance in both variants, Mgfp-3­[Dopa] and Mgfp-5­[Dopa]. They adhered to underwater surfaces with a similar strength as the natural MAPs.^147^ This study clearly demonstrated that the SPI of Dopa in E. coli could produce recombinant MAPs with adhesive properties equaling those of the natural proteins. However, despite delivering recombinant MAPs with excellent Dopa contents, the procedure described by Yang et al. suffered from low protein titers. To address this issue, Kwon and co-workers followed a co-overexpression strategy of a mutant TyrRS. The mutant *Mj*TyrRS^Y32L A67S H70N A167Q^, aka *Mj*DhpRS together with the suppressor *Mj*tRNA_CUA_^Tyr^ from Methanocaldococcus jannashii had been devised by Alfonta et al. for the site-specific incorporation of Dopa at an amber stop codon.^197^ Jeong et al. re-engineered the anticodon of the suppressor-tRNA to AUA so that it decoded Tyr codons and co-overexpressed the *Mj*DhpRS/*Mj*tRNA_AUA_^Tyr^ pair together with Mgfp-3 in the Tyr auxotrophic E. coli strain JW2581 (Table 1). The amino acid sequence of Mgfp-3 contained 10 Tyr residues and was identical to that used by Yang et al.^147^ The overexpression of the *Mj*DhpRS/*Mj*tRNA_AUA_^Tyr^ pair only marginally improved the incorporation efficiency to 93% but the Mgfp-3­[Dopa] titer was elevated 1.5-fold to ∼6 mg/L. In comparison, incorporation efficiency (91%) and titer (4 mg/L) were very similar to the earlier report^147^ when they used the endogenous *Ec*TyrRS. However, the overexpression of the wild-type *Ec*TyrRS increased the titer of Mgfp-3­[Dopa] to 7 mg/L and the incorporation efficiency remained very high (90%).^78^ In summary, neither the overexpression of wild-type *Ec*TyrRS nor of *Mj*DhpRS, which had been evolved for the incorporation of Dopa^197^ substantially increased the product titers of Dopa-labeled recombinant MAP. Nevertheless, the *Mj*DhpRS/*Mj*tRNA_AUA_^Tyr^ pair performed better than another orthogonal pair devised for the incorporation of photocaged Dopa at amber UAG codons by the Budisa group.^372^ They employed a release factor 1 (RF1) deficient and amber stop codon reduced descendant of E. coli strain BL21­(DE3)^373^ to produce 6 mg/L Mgfp-5­(TAG_5_→Dopa) and 1 mg/L Mgfp-5­(TAG_10_→Dopa).^372^ Although the Cha and Budisa groups did not produce the exact same proteins, the number of incorporated Dopa residues was the same, and SPI produced approximately 6 times more Dopa-labeled MAP than SCS.

Bilotto et al. and Deepankumar et al. used recombinant Perna viridis mussel foot protein Pvfp-5­[Dopa] (Table 2) to decipher the role of Dopa in comparison to Tyr in underwater adhesion.^65,70^ They quantitatively exchanged all 17 Tyr residues of Pvfp-5 with Dopa by SPI and obtained a Pvfp-5­[Dopa] titer of 10 mg/L, which corresponded to ∼80% of the parent protein with Tyr. To prevent the autoxidation of Dopa during the production of the protein, they added ascorbic acid and DTT to the growth medium and performed the protein isolation in the dark. They found that Dopa did not substantially improve the adhesion of Pvfp-5­[Dopa] to underwater mica in comparison to the parent protein with Tyr.^65^ Nevertheless, Dopa was indispensable for the liquid–liquid phase separation that is necessary to concentrate the adhesive proteins in coacervate microdroplets such that a glue can be formed under water. In contrast, Tyr did not show such behavior.^70^

Dopa is not the only ncAA in mussel foot protein. It also contains 4tHP (Figure 12), *trans*-2,3-*cis*-3,4-dihydroxy-l-proline, l-phosphoserine, and 4-hydroxy-l-arginine.^184^ The Budisa group assessed the incorporation of 4tHP, 4cHP, 4tFP, and 4cFP (Figure 12) into the Pro-rich MAP Fp151. Fp151 consists of the Mgfp-5 sequence from M. galloprovincialis fused to triple Mgfp-1 decapeptide repeats at the N- and C-termini. It contains 19 Pro residues, which were replaced with the analogs listed above in the Pro auxotrophic E. coli strain JM83 (Table 1). Fp151 was only expressed in the presence of 4tHP and 4tFP but not with 4cHP and 4cFP. Similar to other proteins (see Section 3.3.3), Fp151 tolerated only one of the two C4-stereoisomeres. The expression levels slightly increased when *Ec*ProRS was co-overexpressed. Fp151­[4tHP] was produced at a titer of 0.03 mg/L, which corresponded to only 10% of the parent protein. In contrast, strain JM83 produced 2 mg/L of Fp151­[4tFP], which was ∼7-fold more than the parent (Table 2). Although the mass analysis of both variants revealed protein species that were not fully labeled with the Pro analogs, the overall incorporation efficiency of 4tFP amounted to 93%. The authors did not assess the physicochemical properties of their produced Fp151 congeners. Nevertheless, the substantial increase in the titer of Fp151­[4tFP] relative to the parent suggests either enhanced translation of the protein with 4tFP or a substantially improved protein stability.^126^

Suckerins are a family of proteins that constitute the hard sucker ring teeth of squids. They consist of repetitive units composed of a shorter, Ala-, Thr- and His-rich module which is linked *via* Pro residues to a second longer module rich in Gly, Tyr and Leu. Suckerins self-assemble into a thermoplastic semicrystalline biopolymer. Biomaterials made from recombinant suckerins under varying cross-linking conditions can mimic soft tissue, ligament, muscle tissue or even bone (see the recent review by Miserez, Yu, and Mohammadi^320^). Deepankumar et al.^69^ observed extraordinarily strong underwater adhesive properties of suckerin-12, which exceeded those of Mytilus edulis foot protein-5, one of the strongest natural MAPs.^374^ The near quantitative exchange of 35 Tyr residues of suckerin-12 for Dopa (Figure 15) elevated the protein titer by 25% relative to the parent protein (25 mg/L vs 20 mg/L). Although Dopa did not further improve the underwater adhesiveness of suckerin-12, it might serve as a tunable cross-linker.^69^

The elastomeric protein resilin plays a major role where spring-like elasticity is needed in insect locomotion, for instance in the hinge connecting the wing to the thorax or in the jumping mechanism of fleas. Tyrosine accounts for approximately 5% of resilin’s total weight and forms di- and trityrosine cross-links that are essential for the rubbery properties of natural biomaterials consisting of resilin. Its extraordinary extensibility and elasticity are highly interesting properties for the manufacture of functional recombinant protein biomaterials.^320,375^

In their trend-setting study, Zhu et al. fabricated hydrogels from recombinant resilin-like proteins (RLPs) whose cross-linking could be coordinated by the genetic encoding of Dopa (Figure 15).^56^ In the Tyr auxotrophic E. coli strain BW25113 *tyrA*::kan^R^ (Table 1), they expressed the RLPs *R*32, Ri16 and Ri32 in the presence of Dopa. *R*32 contained 32 Tyr residues as it was composed of 32 repeats of the resilin-like amino acid sequence GGR­PSD­S**Y**G­APG­GGN. The sequence was furnished with a second Tyr residue (GGR­PSD­S**Y**G­APG­GGN**Y**) and repeated 16 and 32 times to construct Ri16 (32 Tyr) and Ri32 (64 Tyr). Very importantly, the group devised a fed-batch fermentation procedure for the scalable production of the recombinant Dopa-containing RLPs. The cells were first grown in salt minimal medium containing glucose and cAAs, Tyr was supplied at 200 mg/L. During the growth in the batch phase, the culture was supplemented with three doses of Tyr at 400 mg each. The repeated dosage intended to “avoid metabolic inhibition by excessive supplementation” (verbatim^56^). When the cells had consumed the glucose in the medium, they were fed with a high-glucose feeding solution to adjust the glucose concentration to ∼1 g/L. As soon as the Tyr in the medium was exhausted and the cells stopped growing, the temperature was reduced to 20 °C and gene expression was induced in the presence of 4 mM Dopa. This process yielded >1.3 g/L of each Dopa-labeled RLP (Table 2), which are the highest titers of Dopa-labeled proteins reported to date. The Dopa incorporation efficiencies were high (Ri32­[Dopa], 76%) to very high (*R*32­[Dopa], 80%; Ri16­[Dopa], 85%). *R*32­[Dopa] was produced in soluble form while the RLPs containing two Tyr residues in the repetitive sequence, Ri16­[Dopa] and *R*32­[Dopa] were insoluble. All Dopa-RLPs were purified and used to prepare hydrogels by coordinative complexation with Fe^3+^ ions. The metal coordination rendered all hydrogels extraordinarily stretchy. *R*32­[Dopa] carrying relatively fewer catechol groups produced “looser” and therefore more flexible hydrogels with excellent shaping and self-healing ability. The relatively higher number of catechol groups of Ri16­[Dopa] and Ri32­[Dopa] increased the coordinative cross-linking density and it facilitated covalent Dopa bonding, which endowed hydrogels made from Ri16­[Dopa] and Ri32­[Dopa] with mechanical stiffness.^56^

### 4.3 Proteome-wide Residue-Specific Incorporation of ncAAs

#### 4.3.1 Noncanonical Amino Acid Tagging Methods

Classic pulse-chase experiments have employed radiolabeled amino acids, for instance to study the biosynthesis of single proteins.^376^ Later, stable isotope labeled amino acids have allowed the analysis of the expression of entire proteomes.^377^ Nevertheless, these experimental setups are time-consuming and the identification of miniscule amounts of proteins presents a major challenge. In 2006, the Tirrell group presented a new method that greatly facilitated proteomic labeling and analysis.^378^ Instead of isotope labeled amino acids, they used noncanonical methionine analogs with bioorthogonal reactive groups such as Aha and Hpg to replace Met in the entire proteome of different target organisms. The MetRS in bacteria,^23,25^ yeast,^154,379^ mammalian cells,^378,380^ insect cells,^381^ plants,^382^ and mammals^383^ accept the Met analogs as substrates. Methionine is an essential amino acid for animals and humans,^384,385^ which means that they are naturally auxotrophic for it. In the case of bacteria and yeast, Met auxotrophic strains can be used (see for instance Section 3.1).

The classic setup of the method that Tirrell and co-workers named **B**io**O**rthogonal **N**on-**C**anonical **A**mino acid **T**agging (BONCAT)^378^ (Table 3; Figure 19) is as follows: after an initial growth phase in the presence of Met, the cells are starved for Met. Then they are supplemented with the reactive Met analog, which is Aha in most cases but also Anl and Hpg can be used. In the plant Arabidopsis thaliana, Tivendale et al. found that Aha was not well incorporated, it inhibited plant growth and stimulated the biosynthesis of Met. Hpg did not show these shortcomings and should be preferred over Aha for BONCAT in plants.^386^ If the proteomic effects of a certain stimulus are to be studied, the stimulus is applied at the same time as the ncAA. In the Met starved cells, the reactive Met analog will replace Met in all newly synthesized proteins (NSPs). The bioorthogonal reactive azido-group on Aha and Anl and the alkyne moiety on Hpg (Figure 7) provide unique reactive handles for conjugation of the NSPs, e.g., with an affinity tag such as biotin for pull-down (Figure 19). The affinity tag facilitates the selective enrichment of the NSPs form the proteome such that they can be identified by mass analysis. When fluorescent tags are used to label the NSPs, the approach may be indicated as FUNCAT, short for **F**l**U**orescent **N**on-**C**anonical **A**mino acid **T**agging (*vide infra*).^387^ After the first introduction of BONCAT by Dieterich et al. in 2006,^378^ the method has gained immense traction. At the time of the preparation of this manuscript, a search with the terms “BONCAT” or “FUNCAT” returned roughly 200 publications from the PubMed database (https://pubmed.ncbi.nlm.nih.gov/). On Google Scholar, the term “BONCAT” alone returned more than 1500 documents. Dieterich et al.’s seminal 2006 paper^378^ has been cited more than 850 times (Google Scholar). These impressive numbers highlight the impact of the BONCAT approach. They also demonstrate that SPI of ncAAs has spread out of its immediate specialist community into a broader context within the life sciences during the last 10 years.

**Table 3:** Proteome-wide Residue-Specific Incorporation Methods^a^

acronym	name/type	ncAA(s)	application	example application	ref(s)
BONCAT	bioorthogonal noncanonical amino acid tagging	Aha; Hpg; Anl	identification	identification of NSPs in the proteome of HEK293 cells; cell-selective NSP profiling	^270^,^378^,^381^,^398^,^404^,^412^,^413^,^415^,^584^
FUNCAT	fluorescent noncanonical amino acid tagging	Aha, Hpg; Aha and Hpg	temporal visualization, dynamic visualization, localization	visualization of NSPs in different cellular compartments upon individual stimuli; NSP dynamics in rat hippocampal neurons (reviewed by Hinz et al.^585^)	^380^,^387^,^397^,^398^,^409^
BONCAT-pSILAC	BONCAT combined with pulsed stable isotope labeling with amino acids in cell culture	Aha	identification, quantification	quantitative secretome analysis of mammalian cells against the complex background of growth medium containing serum proteins; protein secretion kinetics in mouse macrophages following lipopolysaccharide stimulation; analysis of proteome dynamics in HeLa cells	^393^,^394^,^417^
BONLAC	BONCAT combined with stable isotope labeling with amino acids	Aha	identification, quantification	identification and quantification of NSPs in multilayered brain tissue slices that were stimulated with brain-derived neurotrophic factor	^418^
QuaNCAT	quantitative noncanonical amino acid tagging	Aha	identification, quantification	quantification of NSPs after T-cell stimulation with phorbol 12-myristate 12-acetate (PMA) and ionomycin drugs that mimic the activation of the cells with antigen	^419^
MITNCAT	multiplex isobaric tagging combined with pSILAC and BONCAT	Aha	identification, quantification	analysis of changes in protein synthesis rates due to unfolded protein response in MCF10a cells after stimulation with tunicamycin and in HeLa cells stimulated with the epidermal growth factor	^420^
BONCAT-DiDBiT	direct detection of biotin-containing tags	Aha	identification	identification of NSPs in HeLa cells and adult rat retina *in vivo* (no stimulus)	^156^
BONCAT-iTRAQ	BONCAT combined with isobaric tags for relative and absolute quantification	Aha	identification, quantification	identification of NSPs during starvation-mediated autophagy in HeLa cells; NSPs induced by inflammatory cytokines in human monocytic THP-1 cells; discrimination of NSPs from biotinylated host proteins	^158^,^395^,^423^
PALM	pulse Aha labeling in mammals	Aha	identification, quantification	comparative analysis of NSP profiles in two mice phenotypes (liver kinase B1 knockout vs wild-type control) by conjugation with “heavy” (^13^C_3_,^15^N)biotin-alkyne or “light” unlabeled biotin-alkyne	^383^
HILAQ	heavy isotope-labeled Aha quantification	(^13^C_4_,^15^N_2_)Aha, Aha	identification, quantification	evaluation of proteome profile of HT22 cells in oxytosis; simplified workflow due to differential labeling with “heavy” (^13^C_4_,^15^N_2_)Aha and “light” Aha	^384^
BONCAT-PhosID	BONCAT with phosphonate handles instead of the biotin tag	Aha	identification, quantification	differential synthesis of interferon-gamma responsive NSPs over time in HeLa and Jurkat cells	^425^
QUAD	quantification of Aha degradation	Aha	quantification (of protein stability rates)	quantification of protein degradation in different mouse tissues	^427^
THRONCAT	threonine-derived noncanonical amino acid tagging	βeS	visualization, quantification	NSPs analyzed in prototrophic E. coli and HeLa cells as well as anti-IgG stimulated Ramos B cells; visualization and quantification of relative protein synthesis rates in specific cell types in D. melanogaster	^428^
laBONCAT	light-activated BONCAT	phAha	visualization	spatiotemporally controlled labeling of NSPs in HeLa cells that were encapsulated in hydrogels (“synthetic HeLa tissue”)	^429^

a NSP, newly synthesized protein.

**Figure 19:**
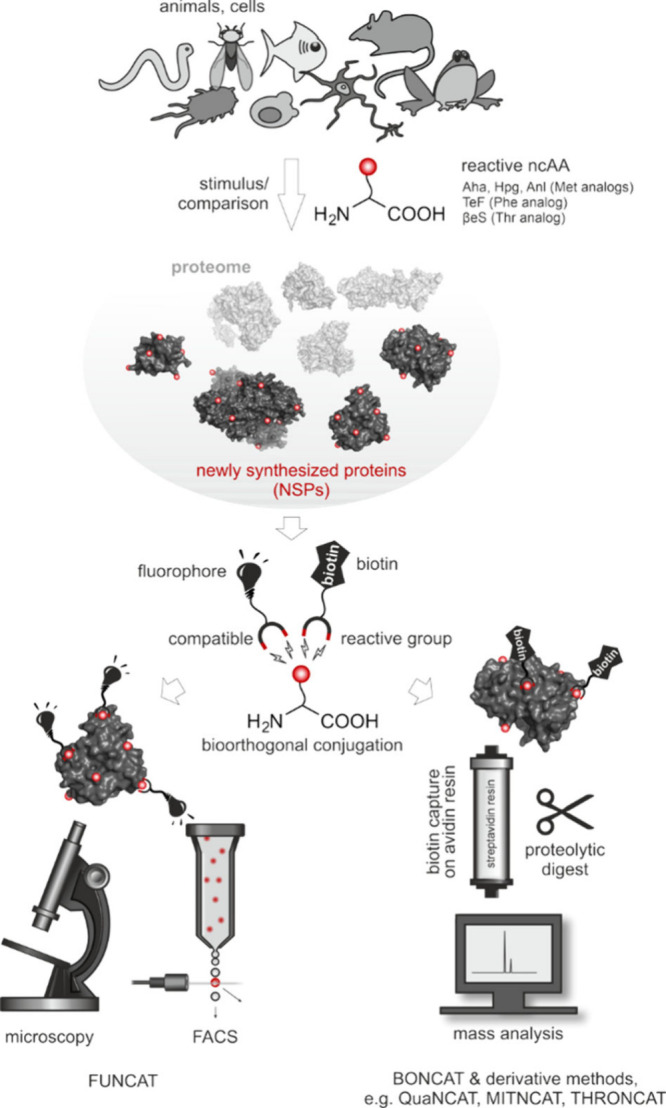
Noncanonical amino acid tagging approaches. Animals or cells are subjected to a stimulus or compared under different conditions in the presence of a reactive ncAA. Proteins that are biosynthesized upon this treatment are residue-specifically labeled with the ncAA throughout the newly synthesized proteome. The proteins are treated with a fluorophore carrying a compatible bioorthogonal reactive group (FUNCAT) for imaging or fluorescence activated cell sorting (FACS). To identify the newly synthesized proteins (BONCAT and related methods, see text and Table 3 for details), they are labeled with biotin, followed by pull-down on avidin resin, proteolytic cleavage, and finally mass analysis.

In addition to bacteria^264,388–392^ and mammalian cells,^157,158,378,393–398^ BONCAT has been applied to a variety of species, such as zebrafish (Danio rerio),^399^ the nematode Caenorhabditis elegans,^400^ the African clawed frog Xenopus laevis,^401^ the fruit fly Drosophila melanogaster,^160,381^ mouse (Mus musculus),^383^ murine tissue and embryos,^163^ and rats^156^ as well as the mouse-ear cress Arabidopsis thaliana.^382,386^ The method has also found applications in microbiome and environmental research.^402,403^

Here, we summarize selected applications of BONCAT, FUNCAT, and their most important derivative methods. Readers interested in a more detailed survey of the topic are referred to more specialized reviews.^384,404,405^ The reviews by Stone et al.,^406^ van Bergen et al.,^407^ and Tang and Chen^408^ survey the ncAA tagging methods in the context of other methods to study nascent proteomes.

Beatty et al. first performed BONCAT slightly differently: Instead of tagging Aha-labeled NSPs with alkyne-biotin, they incorporated Hpg and attached an azide-fluorophore to visualize NSPs in bacteria.^264^ In follow-up studies, they demonstrated the utility of the approach in mammalian cells.^380,409^ The same authors developed a strategy to temporally resolve emerging proteomes in mammalian cells. They incorporated Aha during the first stimulus and Hpg during the second one and then labeled each reactive Met analog with a distinct fluorophore. In this way, they were able to distinguish the proteomes that were induced by the individual stimuli.^397^ A couple of years later, again Dieterich et al. coined the acronym FUNCAT^387^ (Table 3) for the fluorescence labeling of NSPs. They incorporated Aha and Hpg into rat hippocampal neurons to observe the changes in protein synthesis and the fate of the proteins produced in different parts of the cells by fluorescence microscopy.^387^ FACS allows the identification of actively growing microorganisms in soil samples.^402,410^ Similarly, Song et al. incorporated the alkene-Met analog Hag (Figure 7) into the nascent proteome of HeLa cells and labeled the NSPs with a fluorophore by photoinduced alkene-tetrazole cycloaddition reaction for FACS and fluorescence microscopy.^411^ Despite the fact that many studies in the literature use FUNCAT, the term is less common than BONCAT. Often, “BONCAT with fluorescence tagging” describes what is actually a FUNCAT experiment.

The host MetRS that recognizes and charges the Met analogs onto its cognate tRNA_CAU_^Met^ is universally present in the organism involved in a BONCAT experiment. Consequently, the analog is globally incorporated into all NSPs in this organism. In complex heterogeneous environments, such as tissues and multicellular organisms, this can lead to serious problems in protein mapping. To resolve this shortcoming, the Met analog must be selectively incorporated, for instance in the tissue or organ being examined. A mutant MetRS that prefers the analog over Met offers an elegant solution because its selective expression in the cell subpopulation of interest facilitates the incorporation of the Met analog only in these cells. Other cell types that express their wildtype MetRS will not be labeled. As already outlined earlier (Section 4.1.2), the Tirrell group devised the *Ec*MetRS^L13G^ mutant that incorporates Anl^85^ and the *Ec*MetRS^NLL^ mutant that greatly prefers Anl over Met.^102^ Grammel et al. demonstrated the selective incorporation of Anl and the alkyne analog Aoa (Figure 7) in Salmonella typhimurium transformed with expression constructs for the *Ec*MetRS^L13G^ and *Ec*MetRS^NLL^ mutants. The selective incorporation of Anl or Aoa in the proteome of the transformed S. typhimurium cells allowed the visualization and analysis of this pathogen during its infection of mammalian cells.^391^ Truong et al. advanced the method further to selectively label mixed bacteria populations with Anl by the *Ec*MetRS^NLL^ or with Pra using PraRS.^270^ The approach is not limited to bacteria and can be tweaked to selectively label certain positions in NSPs. Ngo et al. introduced E. coli MetRS^NLL^ into HEK293 cells. The bacterial MetRS^NLL^ selectively charged the mammalian initiator tRNA_CAU_^Met^ with Anl. Consequently, Anl was incorporated only at the N-terminal Met positions of NSPs while Met occupied the internal positions.^412^ Evans et al. used the same method to study murine NSPs during memory formation.^413^ Eukaryotic MetRS mutants corresponding to E. coli MetRS^NLL^ are collectively referred to as MetRS^LtoG^. Murine MetRS^LtoG^, *Mu*MetRS^L274G^, allows the analysis of cell-type specific NSPs in response to environmental changes in mouse.^414^ Erdmann et al. demonstrated cell-type-specific BONCAT and FUNCAT in D. melanogaster by the expression of the insect MetRS^LtoG^ mutant, *Dm*MetRS^L262G^.^381^ Similarly, the Tirell group engineered the phenylalanyl-tRNA synthetase from C. elegans, *Ce*PheRS^T412G^, for the cell-type-selective incorporation of 4azF (Figure 9) in the nematode.^415^ Yang et al. devised a corresponding mutant from mouse, *Mu*PheRS^T413G^, and an engineered tyrosyl-tRNA synthetase from S. cerevisiae, *Sc*TyrRS^Y43G^, for the cell-selective incorporation of 4azF and 3azY (Figure 10), respectively, in mammalian cells.^416^

Depending on the biological context and the experimental conditions, the abundance of NSPs can vary greatly. If it is very low, the detection of the NSPs can be very challenging. To facilitate the identification of very lowly expressed NSPs, Eichelbaum et al. combined BONCAT with pulsed supplementation of stable isotope labeled amino acids in the cell culture (pSILAC).^393^ The BONCAT-pSILAC (Table 3) approach facilitated the time-resolved quantification^417^ of low levels of identified NSPs by mass analysis. It revealed its true strength in secretome research. To lower the considerable background caused by serum proteins in BONCAT experiments, fetal bovine serum would be omitted from the cell culture medium. However, Eichelbaum et al. observed that serum-free cultivation altered protein secretion. BONCAT-pSILAC permitted the enrichment of secreted NSPs and their reliable identification as well as quantification in the presence of serum proteins in the medium.^393^ By the same approach, the group was able to characterize the rapid proteomic changes that occur upon lipopolysaccharide-induced macrophage activation.^394^ Bowling et al. used a combination of **BON**CAT with SI**LAC**, which they termed BONLAC (Table 3), to monitor proteomic changes in stimulated intact brain slices.^418^ Howden et al. coined the term “**Qua**ntitative **N**on-**C**anonical **A**mino acid **T**agging” (QuaNCAT) (Table 3) for the combined BONCAT-pSILAC approach. They employed QuaNCAT to study how protein biosynthesis changes when human T cells are subjected to activation stimuli.^419^ Rothenberg and co-workers^420^ elaborated the method further and combined BONCAT and pSILAC with **M**ultiplexed **I**sobaric mass **T**agging (MITNCAT) (Table 3) to detect the response of HeLa cells to stimulation with the epidermal growth factor. They were able to detect changes in protein biosynthesis occurring already within the first 15 min after stimulation. The individual experimental conditions were marked with distinct isobaric tags, which allowed the multiplexed analysis of temporal translation with increased sensitivity.^420^

The efficient and selective enrichment of tagged NSPs is pivotal for the subsequent analysis steps. The classic ncAA tagging methods such as BONCAT, QuaNCAT, and MITNCAT (Table 3) conjugate the azide-labeled NSPs to alkyne-biotin, for instance by CuAAC and employ NeutrAvidin beads to capture the synthetically biotinylated NSPs. To prepare the NSPs for the subsequent MS/MS analysis, the bound proteins are proteolytically digested either directly on the beads or they are first eluted and then proteolyzed. However, the abundance of biotinylated peptides in the complex peptide mixtures originating from the proteolysis of proteomic samples is low. Accordingly low is their chance to be detected and identified by the downstream mass analysis. Schiapparelli et al. elegantly evaded this difficulty by digesting the proteins *before* they enriched the biotin-labeled peptides.^156^ This small alteration of the sample preparation greatly improved the sensitivity of the method. The ratio of biotinylated vs nonbiotinylated peptides was substantially improved, which simplified the discrimination of biotinylated candidates from contaminants. The approach was dubbed “**Di**rect **D**etection of **Bi**o**T**inylated” proteins (DiDBiT) (Table 3). It displayed 200-fold higher sensitivity than traditional methods to enrich biotinylated proteins and facilitated the reliable detection of low abundance NSPs, for instance in HEK293T cells as well as in rat retina. In principle, DiDBiT (as well as the methods using biotin tags) is independent of the biotinylation method, which means that DiDBiT does not necessarily discriminate between biotinylated host proteins and synthetically biotinylated NSPs. Indeed, the removal of naturally biotinylated proteins by treating the protein samples with streptavidin beads *before* the conjugation to alkyne-biotin greatly reduced the number of false positives.^421^ This finding clearly emphasizes the importance of accurate controls to ensure the reliability of BONCAT experiments. In particular, it should be considered that the cultivation conditions and the supplementation of the cells with the ncAAs can affect their metabolism and protein turnover.^422^ The labeling of the NSPs with “**i**sobaric **T**ags for **R**elative and **A**bsolute **Q**uantitation” (iTRAQ) (Table 3) represents another strategy to discriminate between synthetically biotinylated NSPs and biotinylated host proteins.^395,423^ The study and control samples are labeled with distinct tags, which allows the unambiguous identification of biotinylated host proteins.^158^

McClatchy et al. further combined DiDBiT with “**P**ulsed **A**ha **L**abeling in **M**ammals” (PALM) (Table 3).^383^ They substituted Met with Aha in the diet of mice to study the effect of the knockout of liver kinase B1 (LKB1) in mouse livers on the NSP profile. The study confirmed that Aha can be fed to animals to accomplish the *in vivo* labeling of proteins. The NSPs from LKB1 knockout mice were compared to NSPs from wild-type mice by conjugating them with “heavy” stable isotope labeled (^13^C_3_,^15^N)­biotin-alkyne or “light” unlabeled biotin-alkyne for the relative quantification by MS. “**H**eavy **I**sotope **L**abeled **A**zidohomoalanine **Q**uantification” (HILAQ) (Table 3) introduced “heavy” (^13^C_4_,^15^N_2_)­Aha as an alternative to heavy isotope labeled cAAs in pSILAC or PALM with “heavy” and “light” biotin-alkyne described above. This greatly simplified the workflow since peptide enrichment, protein NSP status confirmation, and quantification could all be achieved by supplementing “heavy” or “light” Aha.^424^

The capture of NSPs is not limited to the biotin-streptavidin interaction. Kleinpenning et al. recently introduced phosphonate handles as an alternative to the biotin tag.^425^ They functionalized phosphonic acid with an alkyne-, azide- or DBCO-group for its bioorthogonal conjugation with Aha-labeled NSPs by CuAAC or SpAAC. NSPs carrying such installed phosphonate handles can be enriched using immobilized metal affinity chromatography. Kleinpenning et al. termed their enrichment method PhosID and used a combination of BONCAT-PhosID (Table 3) to study the response of HeLa and Jurkat cells to IFN-γ stimulation.^425^

BONCAT has been used predominantly to study nascent proteomics, nevertheless, it is an excellent method to study protein degradation as well. McShane and colleagues employed it to monitor the degradation kinetics of mouse fibroblast proteins. Basically, they performed a combined BONCAT-pSILAC experiment, but after pulse labeling with Aha the cells were transferred to Aha-free medium. After different intervals of cultivation in Aha-free medium, they isolated the Aha-labeled proteins that had not been degraded until then and identified and quantified them.^426^ McClatchy et al. used a similar approach, which they dubbed “**QU**antification of **A**zidohomoalanine **D**egradation” (QUAD) (Table 3), to evaluate the global protein degradation rates in mouse tissues. To conduct the necessary pulse-chase Aha labeling, they fed the mice on an Aha diet first and then reverted them to a normal diet without Aha. The animals were sacrificed after varying intervals and the remaining Aha-containing proteins were identified and quantified.^427^

BONCAT and all its derivatives described so far (Table 3) use the host MetRS or a selectively expressed mutant to incorporate Met analogs into nascent or degrading proteomes. However, some proteins may not contain any Met residues because their internal sequence lacks Met and the N-terminal Met is cleaved, for instance with the signal peptide during secretion, or by N-terminal Met excision.^173^ Inevitably, such proteins as well as very lowly abundant proteins with only one or two Met residues escape the BONCAT analysis. Threonine is approximately 10 times more abundant than methionine.^246^ The Bonger group found that the threonine analog βeS (Figure 14) was efficiently incorporated into the proteome of prototrophic E. coli, even in complex medium.^428^ The labeling efficiency of the E. coli proteome with βeS was at least comparable to Hpg in a Met auxotrophic E. coli strain background. They successfully employed βeS for proteomic labeling of HeLa and B cells *in vitro* as well as fruit flies *in vivo* and dubbed their new method “**THR**e**O**nine-derived **N**on-**C**anonical **A**mino acid **T**agging” (THRONCAT) (Table 3). THRONCAT is safe (i.e., βeS is not acutely toxic in E. coli, mammalian cells, or D. melanogaster), efficient, and fast and does not require Thr auxotrophic strains or Thr-free media. As such, it appears to be perfectly complementary to classic BONCAT with Met analogs. The combination of both methods might be beneficial when the exhaustive identification of NSPs is necessary, as proteins escaping BONCAT would be detected by THRONCAT and vice versa.^428^

Adelmund et al. developed laBONCAT (Table 3), which is short for light-activated BONCAT.^429^ The method allows the experimenter to precisely control when Aha is admitted to ribosomal translation. To achieve this, Aha carries an N-terminal photocage, 2-(2-nitrophenyl)­propoxycarbonyl-Aha (NPPOC-Aha, phAha) (Figure 7), that is released upon exposure of the compound to near-ultraviolet light. phAha can be taken up by the cells but remains translationally inactive until the photocage is removed. Irradiating their samples through a slitted photomask, Adelmund et al. demonstrated the spatiotemporal control of Aha incorporation into NSPs in HeLa cells encapsulated in hydrogels (“synthetic HeLa tissue”).^429^

Recently, Bassan and colleagues introduced TeF (Figure 9) as a new ncAA to monitor protein biosynthesis by the deep profiling methods mass cytometry and imaging mass cytometry. TeF is so efficiently incorporated (only ∼10–20-fold less efficient than Phe) that labeling in the presence of Phe is possible. TeF is simply added to the culture medium, starvation of Phe or Phe-depleted media are obsolete. In comparison to Tem (see Section 3.4), TeF is stable and only modestly cytotoxic. The authors demonstrated the power of their approach by monitoring protein biosynthesis in different hosts such as E. coli, Jurkat cells, and the human pancreatic cancer cell line PANC-1 as well as in mice. For mice and the cell lines, Phe is an essential amino acid.^26,162^ Unfortunately, they neither indicated which E. coli strain they had used, nor did they reveal its genotype. Given the high incorporation efficiency of TeF, it could have been a prototroph.

#### 4.3.2 Proteome-wide Photo-Cross-Linking

In 2005, Suchanek et al. introduced photoactivatable amino acids to study protein–protein interaction in mammalian cells (Figure 20).^430^ Photo-l-methionine (phM) (Figure 7), photo-l-isoleucine (phI), and photo-l-leucine (phL) (Figure 13) are structurally similar to Met, Leu, and Ile but contain a photoactivatable diazirine ring. When irradiated with UV light (>310 nm), the diazirine ring decomposes into nitrogen and a reactive, short-lived carbene that spontaneously forms a covalent bond with a neighboring molecule. Carbenes can be inserted into C-H and C-O bonds and react with various nucleophiles.^431^ The cross-linking occurs directly without any spacer molecule. Suchanek et al. cultured mammalian fibroblast-like COS7 cells in medium without Met, Leu or Ile, which was supplemented with the photoderivatives of these cAAs. In the presence of phM, phL and phI, the cells grew slower than in complete medium but the photoanalogs neither affected their viability nor their morphology. Most importantly, phM, phL and phI were at least partially incorporated into the cellular proteins. When the cells were irradiated with UV light, photo-cross-linking occurred fast (within minutes) and was specific as it depended on the presence of the photoamino acids containing the photoactivatable diazirine ring.^430^ The method was later extended to protein–protein interaction studies in other mammalian cell lines, e.g., human A549 cells^432^ and HEK293 cells^433^ as well as rat primary hippocampal neurons.^434^ In E. coli, recombinant proteins were also labeled with phM^435–437^ and phL.^438,439^ However, prototrophic host strains were used, which resulted in low to decent incorporation efficiencies (∼30%). Recently, Kohl et al. increased the incorporation efficiency in the prototrophic E. coli host BL21­(DE3) to ∼75% by employing a condensed culture protocol with elevated cell densities.^439^ To study protein–protein interactions, partial labeling with the photo-ncAA is often sufficient.^430,439^ Henderson and Nilles demonstrated the successful application of phM and phL in a multiauxotrophic Yersinia pestis strain to study protein–protein interaction in the type III secretion system,^440^ which is responsible for host cell infection and evasion of host defense mechanisms.^441^

**Figure 20:**
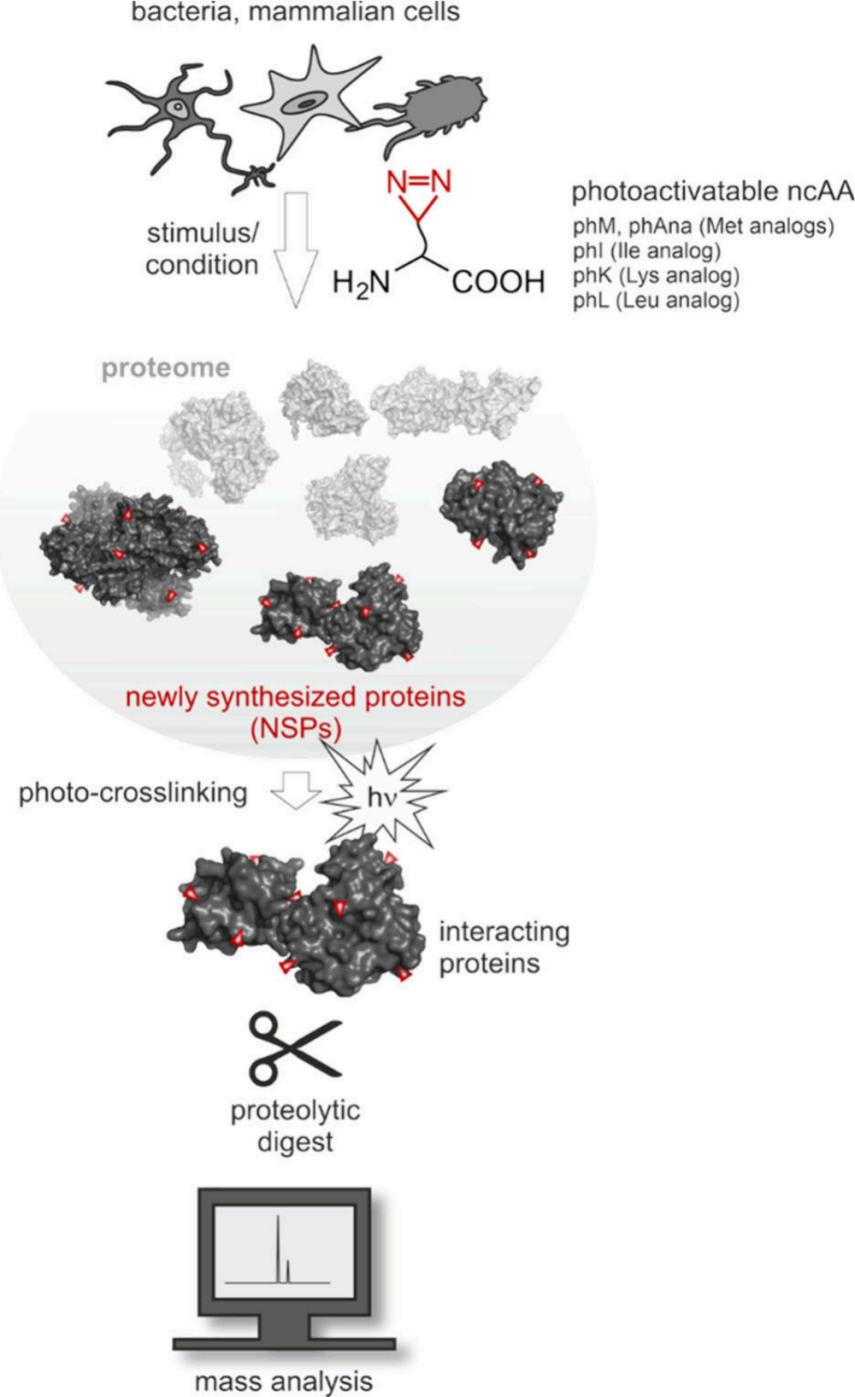
Protein interactome analysis using photoactivatable ncAAs. The photoactivatable ncAA replaces Met, Ile, Leu, or Lys in the proteins that are newly biosynthesized, for instance in response to a stimulus. Irradiation with UV light cross-links interacting proteins which can be identified by mass analysis.

The group of Sinz used a combination of affinity enrichment by photo-cross-linking and mass spectrometry to identify interacting proteins of the human protein kinase D2 (PKD2). They produced PKD2 as a fusion protein with glutathione-S-transferase and immobilized the GDP-PKD2 fusion on glutathione sepharose beads. The beads were then incubated with soluble protein extract of HeLa cells that had been labeled with phM and phL for 24 h in methionine- and leucine-free medium. PKD2-protein complexes were photo-cross-linked by irradiation with UV light. After elution of the photo-cross-linked complexes from the affinity matrix and proteolytic digest, the peptides were mass analyzed to identify the interactors.^442^

Black et al. followed a similar procedure but they expressed the bait protein directly in the cell line in which they intended to study protein–protein interaction. Proteome-wide incorporation of phM in a HEK293 cell line stably expressing the Ca^2+^-binding protein calmodulin (CaM) to study the CaM interactome under different Ca^2+^ conditions, i.e., basal Ca^2+^ concentration, transiently increased intracellular Ca^2+^ concentration and removed extracellular Ca^2+^. CaM contains nine Met residues that are involved in the interaction with target proteins. Proteins interacting with CaM­[phM] were captured by photolysis, the cross-linked complexes were pulled down by an affinity tag fused to CaM­[phM] and the captured proteins were identified by mass analysis after trypsination.^443^

Li and co-workers devised photo-l-lysine (phK) (Figure 13), which is accepted by HeLa cells as a lysine surrogate not only in ribosomal protein synthesis but also for post-translational modifications. They employed phK in HeLa cells to capture proteins that bind to post-translationally modified lysine residues.^444^ Very recently, the same group introduced the multifunctional methionine analog phAna (Figure 7) that combines bioorthogonal functionality employed in BONCAT or FUNCAT with a diazirine group for photo-cross-link. phAna is a substrate of *Ec*MetRS^L13G^ but not of wild-type MetRS, which limits its incorporation to cells or cell types that express this mutant. As such, phAna resembles Anl or Aoa for cell-selective BONCAT (see the previous section). The terminal alkyne moiety in the side chain can be used to attach biotin for streptavidin pull-down or a fluorophore for fluorescence imaging as in BONCAT and FUNCAT. Finally, the photoreactive diazirine group in the side chain can be exploited for protein–protein interaction studies. The Li group demonstrated the versatility and utility of their new methionine analog in the analysis of S. typhimurium infecting HeLa cells. To monitor the dynamic adaptations of the pathogen proteome during the different stages of infection, they used phAna for BONCAT-pSILAC (Table 3). In addition, the photoreactive group of phAna allowed them to dissect the pathogen-host interactome.^445^

## 5 *Quo Vadis*? Challenges for Future Developments

### 5.1 Cellular Uptake of ncAAs and Their Intracellular Fate

Currently, little is known about the cellular uptake mechanisms of ncAAs and their intracellular fate. Nevertheless, these issues can become a primary hurdle for scale-up (discussed in Section 5.4).

For instance, hyperosmotic shock up-regulates proline transporters in E. coli and thus improves the uptake of proline analogs (see also Section 3.3.3).^34^ In a rare example for the systematic assessment of the intracellular uptake of ncAAs, Lilie and co-workers studied the uptake of fluorinated aromatic amino acids in mammalian breast cancer cells.^365^ They treated the human breast cancer cell line MCF-7 with the Phe analogs 2FF, 3FF, and 4FF (Figure 9), the Trp analogs 4FW, 5FW, and 6FW (Figure 11) as well as the Tyr analog 3FY (Figure 10). The fluorinated aromatic amino acids irreversibly inhibited the proliferation of the cancer cells. 4FW, 4FF, and 6FW were most inhibitory with IC_50_ values below 5 μM and at 12 μM and 15 μM, respectively. The IC_50_ values of the other analogs were between 50 and 100 μM. The authors generated radiolabeled derivatives of 4FW and 6FW by enzymatic condensation (see Section 5.2.5.1 for details) of the corresponding indole precursors with ^14^C-l-serine to study the intracellular uptake of the Trp analogs. They found that both radiolabeled analogs accumulated at 70-fold excess inside the cells within ∼20 min, independent of their extracellular supplementation. Nevertheless, higher extracellular supply drove a 70-fold higher intracellular concentration. Competition assays with unlabeled compounds suggested that the Trp analogs were taken up *via* the amino acid transport system L. The authors speculated that the toxic effect of the analogs could have originated from their incorporation into cellular proteins that are involved in cell cycle progression.^365^

Other groups mastered the reluctance of some ncAAs to enter the cell by supplementing them in esterified form^446,447^ or as dipeptides.^448^ In a very recent study, Panke and co-workers presented a rationally designed bacterial import system for sulfonated ncAAs.^449^

### 5.2 Biosynthesis of ncAAs for Residue-Specific Incorporation

The ncAAs’ availability and costs represent severe obstacles for the scale-up of the production of recombinant alloproteins.^110^ Although the commercial availability of ncAAs has improved during the past decade, many compounds are forbiddingly expensive for upscaling.^57^ The chemical synthesis of ncAAs uses harsh reaction conditions and costly, highly volatile, toxic and explosive raw materials, e.g., methylsulfonylchloride or diazomethane.^450–452^ Microbial fermentation can offer an environmental-friendly alternative to the notoriously unsustainable chemical synthesis of ncAAs. While canonical amino acids can be produced from glucose by microbial fermentation,^385^ the biosynthesis of ncAAs from simple sugars is still in its infancy. The biosynthesis pathways of naturally occurring ncAAs are often unknown and their deciphering presents a herculean task.^453^ Artificial biosynthesis routes facilitate the synthesis of ncAAs from cheap precursors and cellular metabolites (Figure 21).^136,140^ Synthetic biology has spurred the design of artificial metabolic pathways yet their efficient integration into the host organism usually requires extensive enzyme-, metabolic- and process engineering.^454–459^ Figure 22 highlights the challenges related to the biosynthesis of ncAAs.

**Figure 21:**
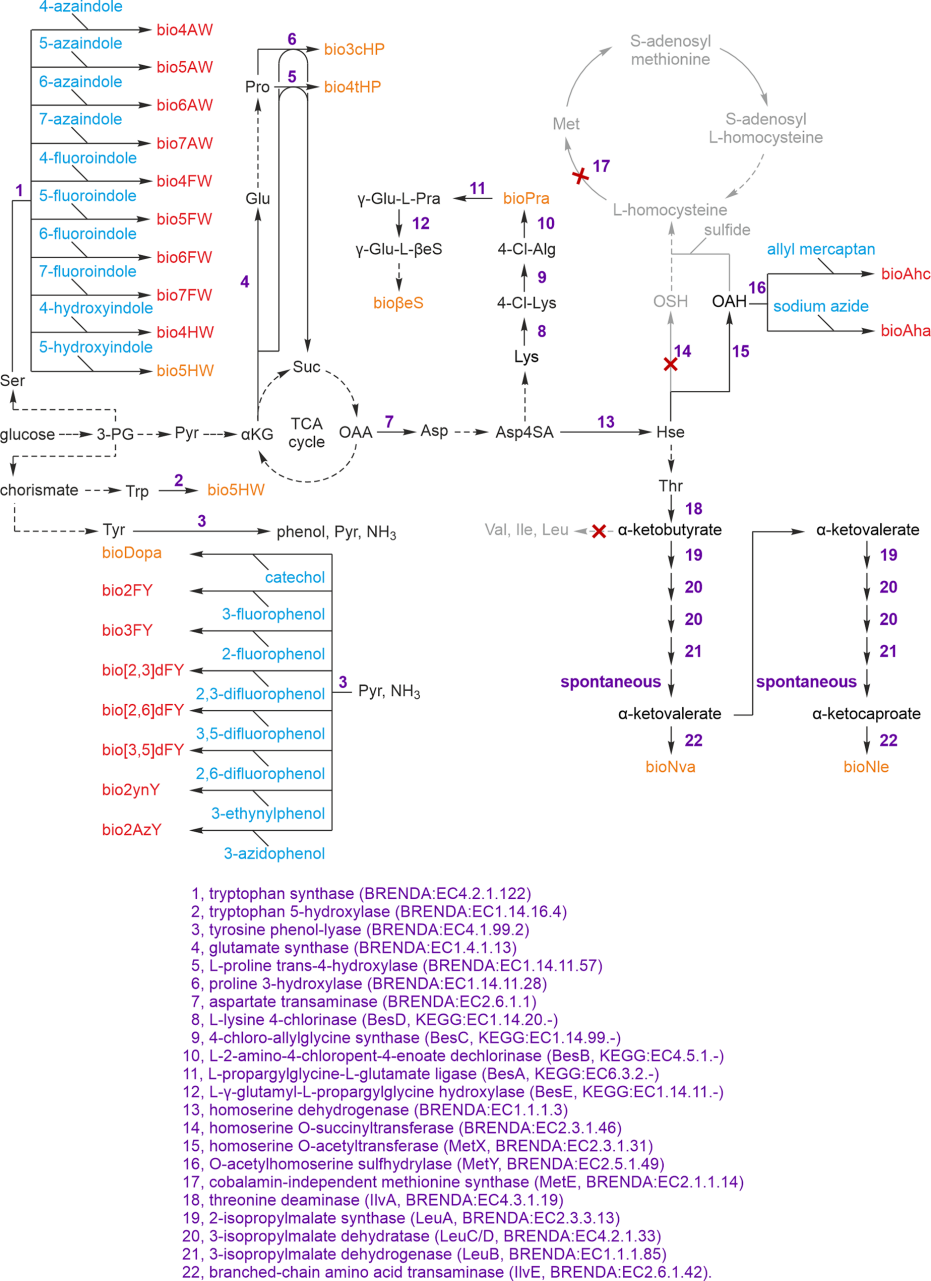
Biosynthesis pathways of noncanonical amino acids for residue-specific incorporation into proteins. In the schematic illustration, naturally occurring ncAAs are indicated in orange while non-natural ones are depicted in red. The prefix “bio” emphasizes that the ncAA is of biosynthetic origin. Precursor molecules added to the medium are highlighted in cyan. Dashed arrows imply that intermediary steps have been omitted for clarity. Blocked competing biosynthesis pathways are shown in gray. Enzymes catalyzing key steps are indicated in purple numbers. Abbreviations: 3-PG, 3-phosphoglycerate; 4-Cl-Alg, 4-chloro-l-allylglycine; 4-Cl-Lys, 4-chloro-l-lysine; Asp, l-aspartic acid; Asp4SA, l-aspartate 4-semialdehyde; bio­[2,3]­dFY, 2,3-difluoro-l-tyrosine; bio­[2,6]­dFY, 2,6-difluoro-l-tyrosine; bio­[3,5]­dFY, 3,5-difluoro-l-tyrosine; bio2AzY, 2-azido-l-tyrosine; bio2FY, 2-fluoro-l-tyrosine; bio2ynY, 2-ethynyl-l-tyrosine; bio3cHP, *cis*-3-hydroxy-l-proline; bio3FY, 3-fluoro-l-tyrosine; bio4AW, 4-aza-l-tryptophan; bio4FW, 4-fluoro-l-tryptophan; bio4HW, 4-hydroxy-l-tryptophan; bio4tHP, *trans*-4-hydroxy-l-proline; bio5AW, 5-aza-l-tryptophan; bio5FW, 5-fluoro-l-tryptophan; bio5HW, 5-hydroxy-l-tryptophan; bio6AW, 6-aza-l-tryptophan; bio7AW, 7-aza-l-tryptophan; bio7FW, 7-fluoro-l-tryptophan; bioAha, l-azidohomoalanine; bioAhc, *S*-allyl-l-homocysteine; bioDopa, 3,4-dihydroxy-l-phenylalanine; bioNle, l-norleucine; bioNva, l-norvaline; bioPra, l-propargylglycine; Glu, l-glutamic acid; Hse, l-homoserine; Ile, l-isoleucine; Leu, l-leucine; Lys, l-lysine; Met, l-methionine; OAA, oxaloacetic acid; OAH, *O*-acetyl-l-homoserine; OSH, *O*-succinyl-l-homoserine; Pro, l-proline; Pyr, pyruvate; Ser, l-serine; Suc, succinate; Thr, l-threonine; Trp, l-tryptophan; Tyr, l-tyrosine; Val, l-valine; αKG, α-ketoglutarate; γ-Glu-l-Pra, γ-l-glutamyl-l-propargylglycine; γ-Glu-l-βeS, γ-l-glutamyl-l-β-ethynylserine.

**Figure 22:**
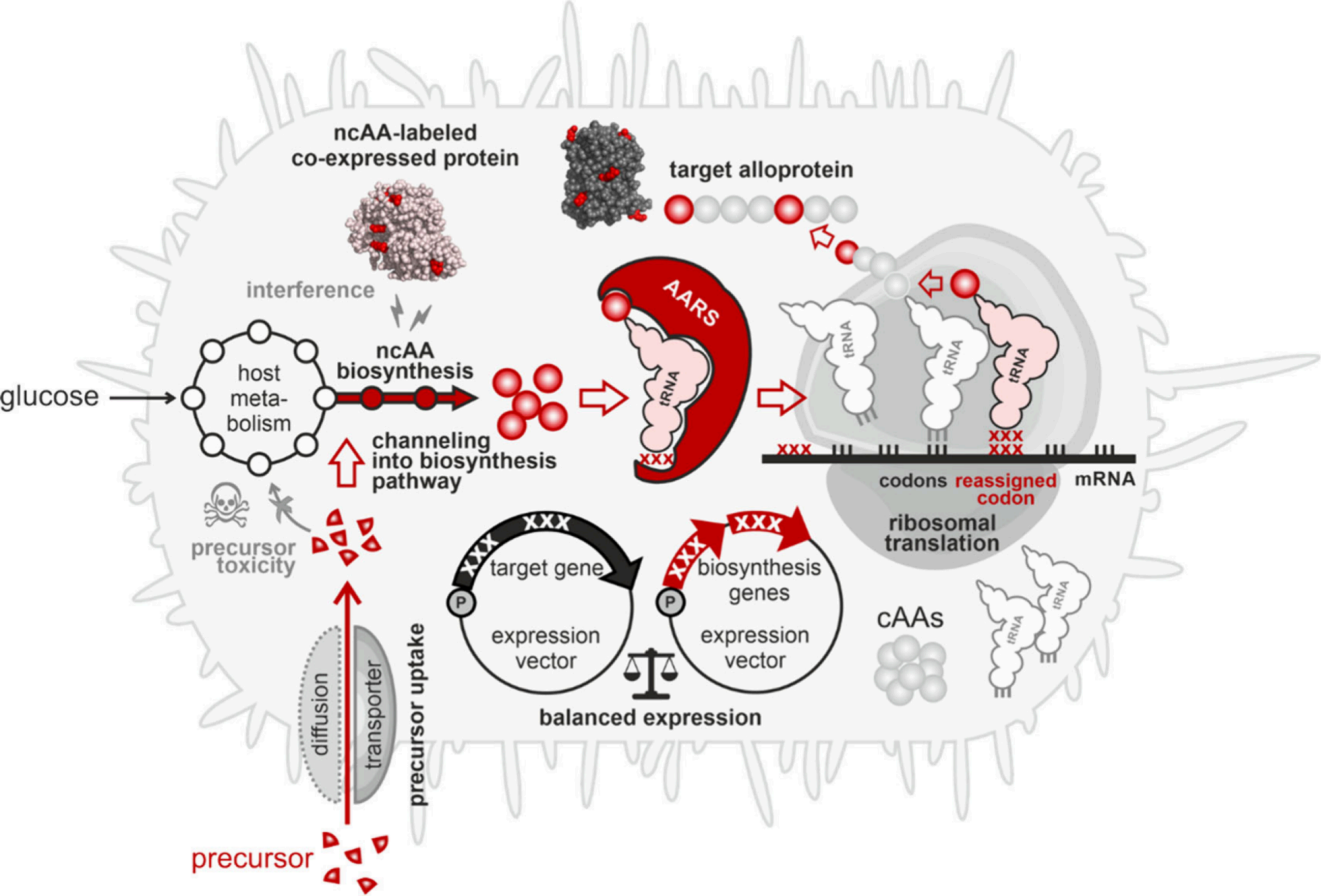
Accidental incorporation of ncAAs into coexpressed proteins and other challenges related to scale-up. NcAAs can be biosynthesized from glucose or precursor molecules. The precursor should not intoxicate the host’s metabolism and should be channeled directly into its biosynthesis pathway. The expression of the target gene and other coexpressed genes, such as those of the ncAA biosynthesis pathway, should be balanced. In this way, the cellular resources are best distributed between biosynthesis of the ncAA and its incorporation into the target protein. The ncAA will be incorporated into any coexpressed proteins, for instance, those of the ncAA biosynthesis pathway or T7 RNA polymerase. This accidental ncAA incorporation can compromise the protein function and thus the expression of the target gene or the biosynthesis of the ncAA.

In this section, we focus on the biosynthesis of ncAAs for the residue-specific incorporation in auxotrophic hosts. A number of recent reviews extend the biosynthesis of ncAAs to a broader perspective.^460–464^

#### 5.2.1 Leucine Analogs

##### 5.2.1.1 l-Norvaline (Nva)

Nva (Figure 7) is a side-product of the branched chain amino acid pathway leading to the biosynthesis of Leu, Val, and Ile (Figure 21) in E. coli. Its biosynthesis starts with α-ketobutyrate (C4-chain), which is elongated to α-ketovalerate (C5-chain) by the *leuABCD* branch of the pathway. Transamination of α-ketovalerate yields Nva. Nva levels increase when cells grow anaerobically on high glucose concentrations. In contrast, Nva is not accumulated in wild-type E. coli K-12 strains during aerobic growth on minimal medium. Soini et al. provoked Nva biosynthesis in the low mM range (∼1 mM Nva) in a 15 L bioreactor culture of E. coli K-12 strain W3110 grown on minimal medium containing excess glucose but with limited oxygen supply.^465^ This observation is relevant when shake flask cultures run into oxygen limitation or in large bioreactor cultures where high glucose concentration and oxygen limitation can occur in feeding zones. Leucyl-tRNA synthase and to a lesser extent methionyl-tRNA synthase can mis-aminoacylate their cognate Leu- and Met-tRNAs with Nva, which leads to the accidental incorporation of Nva at Leu and Met codons.^466^ Apostol et al. observed the stochastic replacement of Leu residues by biosynthetic Nva in recombinant hemoglobin. Hemoglobin is a Leu-rich protein as it contains 72 Leu residues (12%) in a sequence of 575 amino acids.^466^ The high demand for Leu during the production of Leu-rich recombinant proteins such as hemoglobin lowers the intracellular levels of this cAA, which derepresses the branched chain amino acid pathway and promotes the biosynthesis of Nva. To suppress the accidental incorporation of this Leu analog, the growth medium can be supplemented with Leu.

##### 5.2.1.2 5,5,5,5′,5′,5′-Hexafluoro-l-leucine (Hfl)

Hfl (Figure 13) can be incorporated by SPI when the host LeuRS is overexpressed.^467^ Chiu et al. devised a mix of enzymatic and chemical synthesis to produce gram amounts of Hfl. Chemically synthesized hexafluoroleucine pyruvate precursor is turned over to Hfl by l-phenylalanine dehydrogenase (BRENDA:EC1.4.1.20) and formate dehydrogenase (BRENDA:EC1.2.1.2).^468^

#### 5.2.2 Methionine Analogs

##### 5.2.2.1 l-Azidohomoalanine (Aha)

Budisa and co-workers devised a biosynthesis route for Aha and its concurrent incorporation into recombinant proteins in a methionine-auxotrophic E. coli host (Figure 21).^130,469^ To achieve this, they expressed the Corynebacterium glutamicum *metY* gene from a multicopy plasmid in the Met auxotrophic E. coli strain B834­(DE3). *MetY* encodes a pyridoxal 5′-phosphate-dependent *O*-acetylhomoserine sulfhydrylase (cgOAHSS; BRENDA:EC2.5.1.49) that catalyzes the conjugation of *O*-acetyl-l-homoserine with sulfide and thiols, which act as a nucleophile. cgOAHSS accepts sodium azide as a noncanonical substrate nucleophile instead of sulfide, which results in the formation of Aha. The cells were grown in minimal medium containing a limiting amount of Met (0.045 mM) and all other 19 cAA at a concentration of 0.5 mM until Met was exhausted in the midlog phase of growth (*D*_600_ = 0.6–0.8). To prevent misincorporation of the biosynthesized Aha (bioAha) into cgOAHSS, *metY* was expressed from the constitutive *glnS*′ promoter^302^ in the presence of Met. As soon as the cells had consumed all Met, 1 mM *O*-acetyl-l-homoserine and NaN_3_ were added, and the biosynthesis of Aha was allowed to proceed for 1 h, after which the expression of the target gene was induced. In shake flask cultures, Ma et al. were able to produce 5.6 mg/L ψ-b*­[bioAha] (1 Met; Table 2) and similar titers of ψ-b**­[bioAha] (2 Met, see Section 3.1.1; Table 2). Although they did not indicate the titer of bioAha, they reported its incorporation at high efficiency.^130^

The addition of chemically synthesized *O*-acetyl-l-homoserine to the culture medium is not ideal, so the same group extended the pathway for one more reaction appending it to E. coli’s methionine metabolism (Figure 21).^140^ They converted the methionine precursor l-homoserine to *O*-acetyl-l-homoserine using the homoserine acetyltransferase (BRENDA:EC2.3.1.31) from C. glutamicum (cgHSAT, encoded by the *metX* gene) and acetyl-CoA as the acetyl-donor. The resulting *O*-acetyl-l-homoserine then underwent the cgOAHSS catalyzed condensation reaction with NaN_3_ to yield bioAha as described earlier. Basically, bioAha was biosynthesized from an E. coli metabolite and inexpensive NaN_3_ salt.^140^ To divert the flux through the E. coli methionine biosynthesis pathway to the formation of bioAha, Schipp et al. inactivated the *metA* gene in the Met auxotrophic E. coli strain B834­(DE3). *metA* encodes homoserine *O*-succinyltransferase (BRENDA:EC2.3.1.46), which succinylates l-homoserine. The *metA* knockout blocked the succinylation of the intermediate and led to its accumulation for an efficient conversion to *O*-acetyl-l-homoserine with acetyl-CoA by the cgHSAT. The resulting E. coli strain MDS15 was subjected to directed evolution in a turbidostat, which shortened its generation time by 40% but did not improve the tolerance to the toxic NaN_3_. Furthermore, Schipp et al. optimized their shake flask procedure for the biosynthesis of bioAha and its concurrent incorporation into recombinant target proteins with regard to Met source, its depletion, and feeding of NaN_3_ and pantothenic acid (an acetyl-CoA precursor).^140^ They used synthetic minimal medium with a 2.5-fold increased potassium phosphate concentration to elevate its buffer capacity and yeast extract as the Met source. An excess of NaN_3_ interfered with the biosynthesis and/or incorporation of bioAha. To avoid the intoxication of their host cells, they started their feeding regime with 0.8 mM NaN_3_ and 1 mM pantothenic acid three hours after inoculation of the culture and repeated it twice after 75 min each. Eight hours after inoculation, when the growth rate decreased due to the depletion of Met, they induced the target gene expression with 0.5 mM isopropyl β-d-1-thiogalactopyranoside (IPTG). Since *metX* and *metY* were expressed from a multicopy plasmid under the control of the constitutive *glnS*’ promoter, the enzymes were produced continuously during the growth phase in the presence of Met. The intention was to prevent the accidental incorporation of the bioAha which could potentially impair enzyme function and thus hamper its own production.

To demonstrate the versatility of their biosynthesis approach, Schipp et al. incorporated the biosynthesized Aha into the model proteins ψ-b** (2 Met); different mutant fluorescent proteins containing a varying number of Met residues for exchange to bioAha, e.g., GFP-1M (GFP^D134M^, 1 Met), GFP-2M (GFP^T50M D134M^, 2 Met) ECFP-C (5 Met) and ECFP-N (6 Met); as well as the Geobacillus thermoleovorans lipase GTL, which contained 7 Met residues. The maximal production titers ranged between 1.5 mg/L (ψ-b**­[bioAha]) and 36 mg/L (ECFP-N­[bioAha]; all bioAha labeled model proteins and their titers are listed in Table 2) and the incorporation efficiency was higher than 85% except for ψ-b**­[bioAha], where only one of two Met residues was replaced by bioAha. However, even under optimized production conditions, quantitative replacement of Met by bioAha was achieved only with GFP1M­[bioAha] and GFP2M­[bioAha]. These findings emphasize the importance of the efficient depletion of the host for Met before the target gene expression is induced. Finally, Schipp et al. exploited the incorporated bioAha for the postsynthetic modification of selected target proteins with an oligoglycerol dendrimer.^140^

##### 5.2.2.2 *S*-Allyl-l-homocysteine (Ahc)

The *O*-acetyl-l-homoserine sulfhydrylase from C. glutamicum, cgOAHSS, displays a somewhat relaxed substrate specificity. Next to NaN_3_, it also accepts allyl mercaptan as a noncanonical nucleophile substrate. Coupling of allyl mercaptan with *O*-acetyl-l-homoserine by the cgOAHSS yields the unsaturated Met analog Ahc (Figure 21).^136^ Analogous to Ma et al.,^130^ Nojoumi et al. biosynthesized and concurrently incorporated Ahc in the Met auxotrophic E. coli strain B834­(DE3), which was transformed with a multicopy plasmid encoding *metY* under the constitutive *glnS*’ promoter. They used the enhanced synthetic minimal medium described by Schipp et al.^140^ but initiated the Ahc biosynthesis only after the depletion of Met. They supplied synthetic *O*-acetyl-l-homoserine and allyl mercaptan together with pantothenic acid in the growth medium in three consecutive feeds, which were spaced by 90 min intervals. In total, Ahc biosynthesis was allowed to proceed for 8 h before the target gene expression was induced. In contrast to the work of Schipp et al., the coexpression of *metX* encoding the homoserine acetyltransferase from C. glutamicum, cgHSAT, failed to evade the supplementation of the cells with synthetic *O*-acetyl-l-homoserine. The inactive *metA* gene of their strain MDS15 ^140^ allowed the accumulation of *O*-acetyl-l-homoserine. Obviously, in the *metA* proficient B834­(DE3) strain background, the *O*-acetyl-l-homoserine levels produced by cgHSAT were not high enough to biosynthesize sufficient amounts of bioAhc for incorporation. These observations underline the importance of metabolically engineered production hosts for ncAAs.

Nojoumi et al. demonstrated the incorporation of bioAhc in three cysteine-free GFP mutants that differed by the position and number of their Met residues.^136^ The substitution of the single N-terminal Met residue of cfGFPhs1-RM­(M1) by bioAhc occurred at 57% while the single and two internal Met residues of cfGFPhs1-RM­(M134) and cfGFPhs1-RM­(M134:M143) were replaced with 86% and 71% efficiency. Quantitative replacement of Met by bioAhc did not occur. All variants were produced at ∼10 mg/L (Table 2). The approach needs further optimization to allow efficient biosynthesis and concurrent incorporation of Ahc, such as, for instance, the use of a *metAE* double knockout host and full depletion of Met prior to induction of gene expression. Nevertheless, the authors demonstrated the versatility of the desaturated side chain of bioAhc for thiol-ene coupling with thiolated glycan and immobilization on thiol-hydrogels. Deallylation in the presence of a water-soluble palladium catalyst followed by quenching with dithiothreitol transformed the incorporated bioAhc residues into l-homocysteine carrying a thiol moiety in the side chain. Subsequently, the thiol group of l-homocysteine was coupled to small molecules carrying compatible reactive groups for maleimide chemistry and phosphonamidate conjugation.

##### 5.2.2.3 l-Norleucine (Nle)

Like Nva, Nle is a side product of the branched chain amino acid pathway in E. coli (Figure 21). Its biosynthesis also starts with α-ketobutyrate (C4-chain), which is elongated to α-ketovalerate (C5-chain) by the LeuABCD branch of the pathway in a first step. The relaxed substrate specificity of the LeuABCD cascade allows α-ketovalerate to serve as a substrate for another chain elongation, which results in the formation of α-ketocaproate (C_6_ chain). The transamination of this α-keto acid, for instance by the branched chain aminotransferase IlvE (BRENDA:EC2.6.1.42) of E. coli results in the biosynthesis of Nle form glucose. Nle is a hydrophobic Met analog, for instance, its incorporation at eleven positions in the thermophilic lipase TTL greatly enhanced the activity of the enzyme without heat activation.^125^

Wiltschi and co-workers substantially elevated the biosynthesis of Nle in E. coli by increasing the metabolic flux through the LeuABCD pathway.^57^ To accumulate the precursor α-ketobutyrate, they knocked out the *ilvBNIHGM* genes encoding three acetolactate synthase (BRENDA:EC2.2.1.6) isomers in E. coli. In addition, they constitutively expressed the *leuA* ^*fbr*^ *BCD* operon from a multicopy plasmid. LeuA was resistant to feedback-inhibition by free Leu as it carried the G462D mutation.^470,471^ They produced 5 g/L Nle in 1 L fed-batch bioreactor cultures in synthetic minimal medium.

##### 5.2.2.4 l-Propargylglycine (Pra)

Streptomycetes are treasure troves for the biosynthesis of ncAAs. For instance, the Gram-positive soil bacterium Streptomyces cattleya produces the threonine analogs βeS^472^ (Figure 14) and 4-fluoro-l-threonine.^473^ Scannell and co-workers first described the biosynthesis of the terminal-alkyne amino acid Pra (Figure 7) in an unidentified streptomycete. They found that the compound inhibited the growth of B. subtilis in chemically defined medium and that the addition of Leu or Met to the medium reversed the growth inhibition^474^ Recently, the group of Chang deciphered the biosynthesis pathway of Pra and βeS in S. cattleya (Figure 21).^453^ It is encoded by the *besABCDE* gene cluster, where BesBCD are responsible for the biosynthesis of Pra and BesE hydroxylates Pra to βeS. The biosynthesis starts from Lys that is chlorinated at Cγ by the halogenase BesD. The oxidase BesC converts 4-chloro-l-lysine to 4-chloro-allyl-l-glycine, which is transformed to Pra by the pyridoxal 5′-phosphate (PLP)-dependent enzyme BesB. BesA acts as an amino acid ligase and conjugates Pra to Glu to form a γ-Glu-l-Pra dipeptide intermediate. The hydroxylase BesE acts on Pra in the dipeptide intermediate and γ-Glu-l-βeS is generated. Finally, a yet unidentified cellular peptidase releases βeS from the dipeptide.

Marchand et al. reconstituted the *besBCD* pathway for Pra biosynthesis in the Met auxotrophic E. coli strain B834­(DE3) (Table 1).^453^ The coproduction of the chaperones GroEL-GroES was essential for the soluble and functional expression of *besB*. They coexpressed the gene encoding PraRS^270^ to demonstrate the proteome-wide incorporation of bioPra by SPI. Soluble protein extracts were subjected to CuAAC with an azide-dye and then separated by SDS-PAGE. Fluorescence imaging of the SDS gel as well as mass analysis of the cell extracts confirmed the successful incorporation of bioPra in the E. coli B834­(DE3) proteome.

The *besABCDE* pathway is particularly beneficial for the recombinant biosynthesis of ncAAs because virtually all amino acid intermediates can be produced. E. coli BL21 Star (DE3) equipped with an expression plasmid for *besD* produced 4-chloro-l-lysine. Expression of *besC* without *besD* yielded Alg (Figure 7), which can be incorporated into recombinant proteins in E. coli when MetRS is overexpressed.^24^ Although none of these biosynthesized ncAAs were incorporated into recombinant proteins, they could be synthesized in the range of 50–100 μM within 48 h. γ-Glu-l-βeS was biosynthesized *in vitro* with isolated BesA, BesB, BesC, BesD, and BesE.^453^

#### 5.2.3 Proline Analogs

##### 5.2.3.1 *trans*-4-Hydroxy-l-proline (4tHP)

Shibasaki et al. were the first to biosynthesize 4tHP from Pro, α-ketoglutarate and molecular oxygen by l-proline *trans*-4-hydroxylase (P4H; BRENDA:EC1.14.11.57) in recombinant E. coli.^475^ P4H is a α-ketoglutarate-dependent dioxygenase that requires Fe^2+^ as the cofactor. Succinate and CO_2_ are released during the reaction. Since α-ketoglutarate and succinate are intermediates of the TCA cycle of E. coli, the cosubstrate α-ketoglutarate is conveniently recycled by the host. Using an *E. coli putA* mutant strain, they produced 41 g/L 4tHP after 100 h in medium containing Pro and glucose. Quantitative conversion of Pro to 4tHP occurred only after *putA* encoding the bifunctional enzyme proline dehydrogenase/l-glutamate γ-semialdehyde dehydrogenase (BRENDA:EC1.5.5.2/BRENDA:EC1.2.1.88) was inactivated. However, the production of 4tHP was inefficient in medium without supplementation of Pro.^475^

To avoid the supplementation of Pro in the medium, the same group elevated the intracellular levels of this cAA. The first step in Pro biosynthesis is catalyzed by glutamate 5-kinase (BRENDA:EC2.7.2.11, encoded by *proB* in E. coli), which is feedback-inhibited by Pro. In an E. coli *putA* host expressing the *proB74* mutant gene, which encodes a feedback-insensitive glutamate 5-kinase, they were able to produce 25 g/L 4tHP after 96 h in synthetic minimal medium that contained glucose but no Pro. Long et al. further improved the biosynthesis of 4tHP by metabolic engineering of the E. coli host for enhanced Pro biosynthesis and dynamic control of the key enzymes in the TCA cycle.^476^ They obtained up to 55 g/L 4tHP after 60 h in 7.5 L fed-batch bioreactor cultures without the addition of free Pro. Very recently, Gong et al. reported a peak production of up to 90 g/L 4tHP from glucose after 44 h in 5 L fed-batch bioreactor cultures, which yielded to 0.34 g 4tHP per gram glucose. Their production strain was heavily engineered to overexpress the necessary genes and avoid feedback inhibition, and to direct the carbon flux efficiently toward 4tHP. Process engineering enhanced the oxygen transport and ensured the continuous supply of P4H with its cofactor Fe^2+^.^477^ However, the incorporation of biosynthesized 4tHP into recombinant proteins has not yet been shown.

##### 5.2.3.2 *cis*-3-Hydroxy-l-proline (3cHP)

In a similar fashion as 4tHP, 3cHP can be produced from l-proline with α-ketoglutarate and O_2_ using proline 3-hydroxylase (P3H, BRENDA:EC1.14.11.28). The recombinant enzyme was functionally expressed in E. coli,^478^ but the 3cHP biosynthesis needs further improvement.

#### 5.2.4 Tyrosine Analogs

##### 5.2.4.1 Biosynthesis of Tyrosine Analogs from Phenol Precursors

Tyrosine phenol lyase (TPL, BRENDA:EC4.1.99.2) is a PLP-dependent carbon-carbon lyase that biosynthesizes a variety of tyrosine analogs by the condensation of the corresponding phenol precursors with pyruvate and ammonia. TPLs from, e.g., Citrobacter freundii (*Cf*TPL),^479^ Citrobacter intermedius,^480^ or Erwinia herbicola^481^ have been used to biosynthesize monosubstituted fluoro-, chloro-, bromo-, methyl-, iodo-, and methoxy-l-tyrosines^480^ and di- and trifluorinated tyrosine analogs^479^ as well as Dopa if catechol was used as the precursor.^481^

In a recent elaborate study, the group of Yun demonstrated the biosynthesis of a panel of mono- and polyfluorinated tyrosine analogs as well as Dopa and the concurrent incorporation of these ncAAs into selected model proteins.^110^ They employed the Tyr auxotrophic E. coli strain JW2581 (Table 1) to biosynthesize Dopa from the precursor catechol and pyruvate with *Cf*TPL, which was constitutively expressed. The bioDopa was incorporated into GFP-HS.^60,195^ The amino acid sequence of GFP-HS contains 8 Tyr residues, where Tyr66 is present in the fluorophore. The global replacement of Tyr by bioDopa in GFP-HS could be directly monitored as it red-shifted λ_ex max_.^60^ Optimally, 20 mM catechol were supplied in the medium for bioDopa synthesis. The supplementation of the cells with pyruvate in the medium was ineffective as it did not change the level of bioDopa incorporation. The biosynthesis and incorporation procedure differed only marginally from the process where synthetic Dopa was added to the medium. Basically, the cells were first grown in synthetic minimal medium with 0.1 mM Tyr and the other 19 cAA in excess. In midlog, when the cell density reached a *D*_600_ of 0.8–1, the cells were harvested by low-speed centrifugation, washed with 0.9% (w/v) saline and resuspended in fresh synthetic minimal medium containing 19 cAAs but no Tyr. The catechol precursor was added and Dopa biosynthesis allowed to proceed for 30 min at 37 °C before the target gene expression was induced.

GFP-HS­[bioDopa] had the same spectral properties as GFP-HS­[Dopa] that had been produced with synthetic Dopa supplemented in the growth medium. The fluorescence of GFP-HS­[bioDopa] was quenched by Cu^2+^ ions as reported earlier for GFP-HS­[Dopa].^60^ Six to eight Tyr residues were replaced by bioDopa, which corresponds to 75–100% incorporation efficiency, however, the authors did not indicate the protein titer.^110^

In addition to bioDopa, the Yun group also biosynthesized and incorporated into GFP-HS the monofluorinated tyrosine analogs 2-fluoro-l-tyrosine (2FY) (Figure 10; precursor: 3-fluorophenol) and 3-fluoro-l-tyrosine (3FY) (Figure 10; precursor: 2-fluorophenol) as well as the difluorinated tyrosine analogs 2,3-difluoro-l-tyrosine ([2,3]­dFY]) (Figure 10; precursor: 2,3-difluorophenol) and 3,5-difluoro-l-tyrosine ([3,5]­dFY) (Figure 10; precursor: 2,6-difluorophenol). GFP-HS variants with biosynthesized tri- and tetrafluorinated Tyr analogs could not be produced. While the optimal concentration of the catechol precursor was 20 mM as outlined above, the fluorophenole precursors had to be used below 3 mM because they were cytotoxic. Yun and co-workers validated their Tyr analog biosynthesis/incorporation procedure with industrially relevant biocatalysts. They used two ω-transaminases from Sphaerobacter thermophilus containing 14 (ω-TAST-I) and 17 (ω-TAST-II) Tyr residues as well as alanine dehydrogenase (AlaDH, 11 Tyr). Between 5 and ∼10 mg/L bio2FY labeled enzymes were produced (Table 2), which corresponded to 40–60% of the parent proteins. The titers of the other variant proteins were not indicated, but in all cases the monofluorinated Tyr analogs facilitated the production of higher titers than the difluorinated ones: bio2FY ≳ bio3FY > bio­[3,5]­dFY or bio­[2,3]­dFY (very low). bio2FY and bio3FY quantitatively replaced Tyr in all variants, yet the variants with the biosynthesized difluorinated tyrosines were heterogeneously labeled. The enzymes benefitted from the incorporation of the fluorinated Tyr analogs: at elevated temperatures of 60–70 °C, the variant enzymes containing bio2FY retained their activities longer than the corresponding parent proteins and ω-TAST-I­[bio2FY] and ω-TAST-II­[bio2FY] outperformed their parent proteins in a model reaction under elevated temperature conditions.^110^

Similar to the Yun group, Olson et al. also constitutively expressed *Cf*TPL in the Tyr/Phe double auxotrophic E. coli strain DL39­(DE3)^482^ (Table 1) to biosynthesize the monofluorinated analogs 2FY and 3FY (from 3- and 2-fluorophenol as the precursors) and the difluorinated analogs 2,6-difluoro-l-tyrosine ([2,6]­dFY) (Figure 10; precursor 3,5-difluorophenol) and [3,5]­dFY (from 2,6-difluorophenol as the precursor). In addition, they produced two Tyr analogs with reactive side chains, 2-ethynyl-l-tyrosine (2ynY) (Figure 10; precursor: 3-ethynylphenol) and 2-azido-l-tyrosine (2AzY) (Figure 10; precursor: 3-azidophenol).^94^ They quantified the biosynthesized fluorotyrosines by ^19^F NMR and found up to 256 mg/L bio2FY, 236 mg/L bio3FY, and 112 mg/L [2,6]­dFY; bio­[3,5]­dFY was produced only in trace amounts, as were bio2ynY and bio2azY. Although the monofluorinated Tyr analogs were better produced than the difluorinated ones, the overall conversion of the phenols to the corresponding tyrosines was quite low (∼6% at max). The authors speculated that this inefficiency could be caused by the accidental incorporation of the biosynthesized fluorotyrosines into the constitutively expressed *Cf*TPL, which contains 23 Tyr residues in its amino acid sequence. Their biosynthesis and incorporation procedure does not rule out accidental analog incorporation into *Cf*TPL. They grew the Tyr/Phe double auxotrophic expression host first in complex LB medium to the midlog growth phase (*D*_600_ = 0.6–0.8), harvested the cells and resuspended them in defined synthetic medium containing all cAAs except Tyr,^483^ the phenol derivative, pyruvate, PLP and NH_4_Cl. Analog biosynthesis proceeded for 90 min at 37 °C and after 45 min more phenol derivative and pyruvate were added to the medium such that it contained a total of 1.7 mM fluorophenol. The culture was allowed to cool to 20 °C for 30 min before the target gene expression was induced. Since *Cf*TPL was expressed under the control of a medium strength constitutive promoter in combination with a medium strength ribosome binding site, misincorporation of the Tyr analogs into *Cf*TPL could have happened during the biosynthesis- and target protein production phases where the medium lacked Tyr. Unless the enzyme was turned over rapidly, sufficient amounts of functional enzyme might have accumulated, though, during its constitutive production in the growth phase in complex medium containing Tyr.

Indeed, Olson et al. observed an incorporation efficiency of 50–100% of bio2FY and bio3FY in *Cf*TPL. In contrast, incorporation efficiencies of 5–40% bio2FY and ∼20% bio3FY were drastically lower for their target, the bromodomain protein BRD4­(D1). It resulted in inhomogeneous labeling with multiple species of fluorinated protein variants. These results do not reflect a low amenability of BRD4­(D1) to labeling with fluorotyrosines because synthetic analogs are well incorporated (>90% efficiency) by SPI. While *Cf*TPL was expressed from a medium strength constitutive promoter in combination with a medium strength ribosome binding site, BRD4­(D1) was under control of a strong IPTG-inducible T7 promoter in combination with a strong ribosome binding site. From this configuration one would expect higher expression levels of BRD4­(D1) than of *Cf*TPL. However, BRD4­(D1) contained 7 Tyr residues while *Cf*TPL contained 23, which is more than three times as many. Obviously, the medium-level expression of this Tyr-rich protein drained the pool of bio2FY and bio3FY such that too little remained for the production of fluorinated BRD4­(D1). Again, the activity of the T7RNAP could have been impaired by the misincorporation of the Tyr analogs. Clearly, the biosynthesis of Tyr analogs as well as the procedure for their concurrent SPI need further optimization. For instance, to increase the amount of biosynthesized Tyr analogs, *Cf*TPL could be engineered to better accept the phenol derivatives. To prevent the accidental incorporation of the biosynthesized Trp analogs into TPL, an analog with fewer Tyr residues could be chosen or the expression of the gene might be shut down during the incorporation phase.^94^

#### 5.2.5 Tryptophan Analogs

##### 5.2.5.1 Biosynthesis of Tryptophan Analogs from Indole and l-Serine

Tryptophan synthase (TrpS, BRENDA:EC4.2.1.122) is a PLP enzyme that catalyzes the condensation of l-serine with indole (Figure 21).^484^ TrpS (e.g., from S. typhimurium, Salmonella enterica, or E. coli) shows a broad tolerance toward the indole substrate. Many ring-substituted Trp analogs are accessible by *in vitro* and *in vivo* biosynthesis.

The Budisa group biosynthesized 4AW, 5AW, 6AW, and 7AW by adding the corresponding indoles to the synthetic growth medium. Obviously, the indole derivatives were taken up by the cells and condensed with intracellular Ser to form the Trp analogs by the E. coli TrpS.^127,311^ Alternatively, they performed the conversion of the azaindoles with Ser to the Trp analogs *in vitro* using isolated TrpS. This mixture was added to the growth medium without further purification to relieve the cellular burden associated with the biosynthesis and concurrent incorporation of the analogs.^127^ In another study, they equipped their expression host with a multicopy plasmid for the recombinant expression of S. typhimurium TrpS. When supplemented with 4*H*-thieno­[3,2-*b*]­pyrrole, the cells biosynthesized the Trp analog [3,2]­Tpa,^202^ which was incorporated into RiPPs (see Section 3.3.1).^81^

Tobola et al. biosynthesized a panel of fluorinated Trp analogs, bio4FW, bio5FW, bio6FW, and bio7FW (Figure 11) from the corresponding 4-, 5-, 6-, and 7-fluoroindole precursors and cellular Ser by the host TrpS of the Trp auxotrophic E. coli strain BWEC47 Δ*trpC* (Table 1).^142^ The Trp analogs were incorporated into the Ralstonia solanacearum lectin RSL. The lectin is a homotrimer with 7 Trp residues per monomer, 6 of which are involved in carbohydrate binding. The RSL variants containing bio4FW, bio5FW, bio6FW and bio7FW were produced at titers comparable to the parent protein (Table 2), however, the RSL­[bio6FW] variant was entirely insoluble. All biosynthesized Trp analogs were incorporated at ∼85% efficiency. They affected the thermostability of the variant RSLs depending on the position of the fluorine substituent in the indole ring. RSL­[bio5FW] displayed a slightly increased melting temperature in comparison to the parent protein (+1 and + 2 °C with and without d-mannose and α-l-fucopyranoside as the glycan ligands), while the melting temperatures of RSL­[bio4FW] (−9 °C with and without glycan ligand) and RSL­[bio7FW] (−16 °C without glycan; −9 °C with methyl α-l-fucopyranoside and −14 °C with d-mannose) were substantially decreased. The fluorination of the Trp residues at position 7 in the indole weakened the binding of the RSL to blood group B trisaccharides.

In a follow-up study, Tobola et al. extended their Trp analog biosynthesis/incorporation procedure to 12 fluoro-, hydroxyl-, methyl-, and amine-Trp analogs and another lectin, galectin-1 (Gal-1).^144^ In contrast to RSL, Gal-1 contains only a single Trp residue in its glycan binding site. This unique Trp is highly conserved among galectins and plays a key role in the interaction with their carbohydrate ligands. Gal-1­[4FW] was insoluble but the remaining 11 soluble variants were purified and the incorporation efficiency of the Trp analogs was assessed. 4-, 5-, and 6AW as well as 1-methyl-l-tryptophan were not incorporated. Gal-1­[7AW] was produced at ∼60% of the parent protein and Trp was quantitatively replaced by 7AW. The fluorinated Trp analogs were incorporated at ∼85–95% efficiency and the variants amounted to ∼40–70% of the parent protein. While the incorporation efficiencies of the hydroxylated Trp analogs and 4NW were still high (60–80%), these variants were much less abundant than the parent protein (15–20%, see Table 2). By a glycan microarray, the authors assessed the affinity of the Gal-1 variants for >300 glycans. While the glycan binding profiles of most variants largely resembled that of the parent protein, Gal-1­[7FW] and Gal-1­[7AW] showed drastically reduced affinity for 3′-sulfated oligosaccharides. This study not only demonstrated the flexibility of the E. coli Trp biosynthesis machinery for the *in situ* biosynthesis of a variety of Trp analogs from their corresponding indole precursors. It also highlighted the power of atomic mutations to modulate the interaction of binding proteins with their ligands. SPI is particularly suitable, for instance, when a single conserved Trp residue cannot be addressed by classic mutagenesis without compromising the protein’s function.

##### 5.2.5.2 5-Hydroxy-l-tryptophan (5HW)

The direct enzymatic hydroxylation of Trp with tryptophan 5-hydroxylase (T5H, BRENDA:EC1.14.16.4) (Figure 21) represents an alternative to the condensation of 5-hydroxyindole with Ser to 5HW. T5H from humans and animals is a pterin-dependent aromatic amino acid hydroxylase. The reconstitution of this alternative pathway for 5HW in a recombinant host such as E. coli requires the recycling of the cofactor tetrahydrobiopterin (BH4). However, mammalian enzymes can be notoriously difficult to express in E. coli (see Lin et al.^485^ and references therein). Bacteria encode a few phenylalanine 4-hydroxylases (P4Hs, BRENDA:EC1.14.16.1) that belong to same enzyme class as T5H but predominantly hydroxylate Phe. For instance, P4H from Xanthomonas campestris (XcP4H) shows high activity with Phe as the substrate when recombinantly expressed in E. coli but it accepts also Trp to a low extent. Lin et al. engineered the XcP4H^W179F^ mutant that showed a higher preference for Trp than the wild type. They coexpressed the mutant gene together with two genes of the cofactor recycling system from a medium copy plasmid to alleviate the overburdening of the cells by the gene overexpression. To provide sufficient Trp as the substrate for hydroxylation, they expressed a feedback-insensitive version of the *trpE* ^*fbr*^ *DCBA* operon from a low copy plasmid under control of an IPTG-inducible promoter. The recombinant promoter evaded the regulation of the pathway at the transcription level and the *trpE* ^*S40F*^ mutation prevented feedback inhibition by Trp.^486^ They chose a *tnaA* deficient strain background for 5HW biosynthesis and lowered the temperature from 37 to 30 °C to avoid the oxidative degradation of Trp and potential other side reactions. These measures directed the metabolic flux to 5HW, which was produced at a titer of ∼150 mg/L.^485^

More recently, Wang et al. produced recombinant human T5H together with its cofactor recycling system in E. coli. They employed a combinatorial engineering strategy to improve the biosynthesis of 5HW. They engineered the mammalian enzymes for better solubility and manipulated the copy number of their expression plasmid to relieve the metabolic burden on the cells. Finally, transcriptional fine-tuning directed the metabolic flux into the biosynthesis of 5HW such that they were able to produce 1.3 g/L in shake flask cultures and 5.1 g/L in a 7 L fed-batch bioreactor culture.^487^ Replacement of the heavily feedback-inhibited *aroH* and *trpE* genes in the E. coli host genome by feedback resistant *aroH* ^fbr^ and *trpE* ^fbr^ mutant alleles under control of a regulatable *tac* promoter facilitated a small further increase of the 5HW titer to 1.6 g/L in shake flask cultures.^488^ 5HW produced by this pathway has not yet been incorporated into proteins.

### 5.3 Accidental Incorporation of ncAAs into Coexpressed Proteins

As already outlined in Section 2, ncAAs can be accidentally incorporated into proteins that are coexpressed with the target gene (Figure 22). Ayyadurai et al. observed that the Met analog Hpg (Figure 7) impeded the function of T7RNAP.^58^ They expressed their target genes under the control of the T5 and T7 phage promoters. The T5 promoter is recognized by the E. coli RNA polymerase, which is constitutively produced in the host. The production of the enzyme during the growth phase in the presence of Met should produce sufficient functional protein for the transcription of the target gene in the presence of the analog. In contrast, the T7 expression system exploits the T7RNAP, which is orthogonal in E. coli and does not recognize host promoters.^489^ In E. coli DE3 strains such as the Met auxotrophic B834­(DE3) (Table 1), T7RNAP is expressed under the control of the IPTG-inducible *lacUV5* promoter from the E. coli chromosome.^490^ During the growth phase in the presence of Met, only leaky expression of the enzyme may occur under noninducing conditions. However, when the target gene is expressed from an IPTG-inducible T7 promoter, T7RNAP is coexpressed, and the Met analog can be incorporated. If the analog inhibits T7RNAP, the enzyme that was produced during leaky expression may kick in unless it was turned over or the levels are insufficient to efficiently drive the target gene expression.

Ayyadurai et al. tested this hypothesis by the incorporation of Hpg (Figure 7) into the target proteins GFPmut3.1 ^491,492^ and the chimeric scFv against the hepatocyte growth factor/scattering factor c-Met (anti-c-Met cscFv) which contained six and two Met residues, respectively. They expressed both target genes under the control of the T5 and T7 promoters in the Met auxotrophic E. coli strains M15A and B834­(DE3) (Table 1). While the expression under control of the T5 promoter in presence of Hpg was comparable to the positive control with supplemented Met, the expression under control of the T7 promoter equaled the negative control without Hpg or Met added. The expression levels could not be improved by the co-overexpression of *Ec*MetRS under control of its own promoter from an episomal plasmid. T7RNAP contains 25 Met residues, one of them is located in the active site of the enzyme. The experimental evidence suggests that the global replacement of Met by Hpg inactivates T7RNAP.^58^ Obviously, the amount of functional enzyme that was produced by any leaky expression during the growth phase with Met did not suffice for the efficient transcription of the target gene in the presence of Hpg.

Although they did not discuss it in their study,^116^ Al Toma et al. may have experienced the same issue. They introduced Aha and Hpg into a lasso peptide (see Section 3.3.1) and observed low to very low expression levels, respectively. They expressed the genes for the target peptide as well as the maturation helper enzymes from a T7 promoter and employed the standard cAA limitation approach (see Section 2.1). Most probably, Aha and Hpg were not only incorporated into the target peptide but also into the helper enzymes and, more importantly, into the T7RNAP. The putative labeling of the helper enzymes with Aha or Hpg did not substantially impair their function because the mature forms of the lasso peptide variants with Aha and Hpg were found. Dysfunctional T7RNAP­[Hpg] and T7RNAP­[Aha], however, may have caused the low expression levels. Apparently, the incorporation of Aha into T7RNAP has less dramatic effects than Hpg. Their alternative SCS approach to incorporate Lys analogs employed the amber stop codon to encode the ncAAs. Misincorporation of the Lys analogs into T7RNAP by this method is highly unlikely and might explain the substantially higher variant titers.

To avoid this issue, the group of van Hest added a short preinduction step for T7RNAP to the media shift procedure. Once the cells had reached the desired density, they induced T7RNAP expression with IPTG for 15 min in the presence of Met, then the cells were washed to remove the inducer. The cells were resuspended in minimal medium without Met, starved for 10 min and then the production of the target protein with Aha or Hpg was initiated. Obviously, the short preinduction step with IPTG did not produce substantial amounts of unlabeled target proteins since the incorporation efficiencies were good to excellent.^47–50^

Völler et al. took a different approach to tackle a similar problem.^51^ First, they expressed the genes for the biosynthesis of heme under the control of an IPTG-inducible promoter in the Phe/Tyr double auxotrophic host BL21­(DE3)­Δ*tyrApheA* (Table 1). Then they washed the cells, starved them for Phe or Tyr for 4 h and finally induced the expression of the hemeprotein cyt c from an arabinose-inducible promoter in the presence of 4FF (Figure 9) or 3FY (Figure 10). This procedure produced substantially higher titers of ncAA-labeled hemeprotein than a standard SPI experiment where all genes were expressed simultaneously. A similar cross-expression approach was developed by Kuipers and co-workers for the expression of nisin and its PTM enzymes in L. lactis (see Section 4.1.1.1). Ma et al. and Schipp et al. used a combination of constitutive and inducible promoters for the biosynthesis and incorporation of Aha (see Section 5.2.2.1).^130,140^ Olson et al. also employed a constitutive promoter for the expression of their Tyr analog biosynthesis enzyme but they still observed misincorporation of Tyr analogs into it (see Section 5.2.4.1).^94^

### 5.4 Challenges Related to the Scale-Up of SPI

The basis for each residue-specific ncAA incorporation project is the choice of an appropriate auxotrophic protein production host. Since E. coli has been the primary host for SPI of ncAAs (see Section 2), we will focus on this biotechnology work horse here. Nevertheless, most challenges related to establishing an SPI procedure and scale-up of the method should be valid also for other microbial hosts.

A major obstacle is the lack of suitable auxotrophic host strains that are compatible with commonly used expression vectors for the overproduction of recombinant proteins. Starting form the broadly employed E. coli strains C43­(DE3) and BL21­(DE3), Iwasaki et al. generated a set of auxotrophic host strains for amino acid-selective isotope labeling.^493^ These strains are accessible through public strain banks such as Addgene (https://www.addgene.org/; see also Table 1 for sources of auxotrophic hosts) and can be a valuable resource for SPI of ncAAs. Alternatively, strains carrying the desired amino acid auxotrophy can be generated by homologous recombination methods such as λ Red recombineering.^494^ This approach disrupts the target gene by the insertion of an antibiotic resistance cassette *via* flanking 35–50 nt homologies. The cassette carries direct inverted repeats which facilitate its later excision from the new knockout strain by the flippase (FLP) recombinase expressed from the pCP20 vector.^495^ Alternatively, CRISPR/Cas9 can be used to knock out genes without a resistance marker.^496^

Once an auxotrophic expression host is available, it can be transformed with an inducible expression construct for the recombinant protein of choice. Usually, the SPI of one or several amino acid analogs is tested first in shake flask cultures. To allow reliable scale-up to bioreactor-scale cultures, the selected strain should display uncompromised growth in synthetic minimal growth medium, e.g., M9 medium,^149^ when supplemented with sufficient amounts of the corresponding essential cAA. For obvious reasons, media shift protocols with the associated cell harvesting steps cannot be executed in bioreactor cultures. Thus, the demand for the growth-limiting cAA must be determined experimentally, specifically, this is the quantity of cAA that is necessary to form one unit of biomass. Usually, it is expressed in mg cAA per g of cell dry mass or similar. The simultaneous uptake and utilization of amino acids and glucose by E. coli is highly dynamic and the coordination of the corresponding catabolic pathways intricately complex.^497^ If the host cells efficiently take up the ncAA of choice and accumulate it intracellularly, successful high-level SPI of the ncAA can occur. Timely charging of the tRNA(s) with the ncAA and delivery of the ncAA-charged tRNA(s) to the ribosome by the E. coli elongation factor EF-Tu are crucial as well. For instance, for the incorporation of Met analogs, it should be considered that E. coli regulates its Met transport activity by the intracellular Met pool size and possibly by repression processes.^498^ Marin and Krämer reviewed the amino acid transport systems in biotechnologically relevant bacteria.^499^ In addition, the cAA pool size has an impact on the ribosome synthesis rate.^500^ Consequently, the development of robust and scalable processes for the efficient incorporation of ncAAs in E. coli auxotrophs necessitates (i) a comprehensive understanding of the intracellular uptake of ncAAs; (ii) alongside a profound grasp of the catabolic and anabolic pathways related to the cAA for which the host organism exhibits auxotrophy.

The unknowns of recombinant protein production in an auxotrophic strain that is supplemented with cAA analogs are considerable. Presumably, the supplementation of cAA-depleted cells with an ncAA involves interventions at multiple layers in the host cell’s regulatory networks. Typically, amino acid starvation triggers an extensive reaction of the regulatory network, the stringent response.^501,502^ The onset and extent of the stringent response depends on the host strain, the expression vector, the specific protein-of-interest as well as the process control strategy.^503–505^ Auxotrophic E. coli strains are sensitive to imbalances in media composition or process conditions. Often, amino acid auxotrophies are generated by the inactivation of late genes in the corresponding cAA biosynthesis pathways, e.g., the lesion in *metE* in E. coli strain B834­(DE3) inactivates the last step of Met biosynthesis. This leaves earlier steps intact and thus vulnerable to metabolic imbalances. Moreover, the cAA analogs themselves could interfere with the cellular uptake and metabolism and cause unexpected—and in the worst case irremediable—side effects on the efficiency of their own incorporation during ribosomal translation. To date, SPI of ncAAs has been predominantly established in batch cultures. As outlined in Section 2, the incorporation of the ncAA is usually initiated after the depletion of the corresponding cAA and the cells are at the verge to entering the stationary phase. However, this point in time appears to be nonoptimal because the amino acid uptake rates decline, and the ribosome content stagnates.^506–508^ In the light of these considerations and for the reasons outlined above, it might be highly beneficial to stage the cAA depletion during the fed-batch phase of a high-cell density production process. Even more so because the misincorporation of noncanonical side products of the cAA biosynthesis pathways such as Nva and Nle occurs.^466,509^ To study their misincorporation in more detail might guide process outlines for the deliberate, high-level SPI of ncAAs. In addition, tRNA abundance and modification as well as the tRNA charging status of the isoacceptors concerned with ncAA incorporation should be taken into account. Recent methods to determine tRNA abundance, modification^43^ and charging status^510,511^ could provide insight into these rate limiting parameters.

To become applicable for industrial use, the SPI process must be scalable and operatable at bioreactor scale. The key challenge lies in ensuring the ample supply of the desired ncAA. The cellular uptake mechanisms of ncAAs and their possible intracellular degradation have largely been neglected so far. Only a single systematic report scrutinizing the uptake of Trp analogs in mammalian cells is available to date.^365^ A robust yet sensitive method to quantify the extra- as well as intracellular ncAA concentrations presents a basic yet essential prerequisite for the rational development of an SPI bioprocess. It would allow the precise identification, characterization and classification of existing bottlenecks that affect the intracellular availability of an ncAA for ribosomal translation. Most importantly, it might hint at suitable remediation strategies. The intracellular uptake of ncAAs during the different phases of a fed-batch cultivation could be analyzed by ultrahigh performance liquid chromatography (UHPLC)^512^ or by reversed-phase high performance liquid chromatography (RP-HPLC) with derivatization^513^ or without derivatization by LC-MS/MS.^514^ Alignment of the ncAA uptake data with the different process parameters would allow the optimization of the process, e.g., with regard to the ncAA feeding regime, which is crucial for the development of industrially relevant setups. For each ncAA/expression host combination, it will be imperative to develop a knowledge base integrating the specifics of the ncAA uptake, its degradation, and intracellular accumulation as well as the optimal form of supply. In contrast to current SPI methods, which rely mainly on trial and error or educated guess, this strategy avoids overdosing yet allows efficient protein labeling, which will keep the costs as well as a waste of resources at bay.

The challenges of scalable SPI warrant a closer look to the vector for target gene expression. Episomal plasmids carrying antibiotic resistance markers, which are maintained when the cells are supplemented with the antibiotic, are conveniently used in small scale E. coli shake flask cultures. The costs as well as the potential contamination of the recombinant product with the antibiotic prohibit this selection strategy at large scale. The maintenance of plasmids at high copy numbers puts a considerable metabolic burden on the host cells, which may lead to plasmid loss.^515–517^ Plasmid loss is a phenomenon tightly related to the genetic background of the host and the type of plasmid. Eventually, it results in a nonproducing plasmid-free cell population that grows at the expense of producer cells, thus limiting large-scale production.^518–521^ In numerous studies, the Striedner group showed that plasmid-free systems are preferable to conventional plasmid-based systems for the production of recombinant proteins in fed-batch cultivations.^521–524^ The expression cassettes were integrated site specifically into the host genome either *via* λ Red recombineering^494^ or *via* a protocol for fast and antibiotic free integration.^525^ Transcriptomics in combination with a variety of process analytics revealed that the plasmid-free system provoked a moderate stress response with only minor effects on cell growth. In contrast, comprehensive changes were observed in the transcriptome of cells carrying an episomal expression plasmid and the recombinant gene expression heavily impaired cell growth. The comprehensive analysis suggested that in the latter constellation, high levels of recombinant mRNAs competed for a limited number of ribosomes, which led to a significantly reduced translation of host mRNAs.^521^ Genomically integrated expression constructs perform excellently in terms of productivity. A plasmid-free system with only a single copy of the gene of interest integrated into the chromosome produced a Fab antibody fragment at yields ranging from 80 to 300% of the conventional plasmid-based system.^522^ Taken together, the scalable production of recombinant proteins definitely profits from plasmid-free expression systems. Future studies will have to demonstrate their utility for proteins produced by SPI.

A recent report by Karbalaei-Heidari and Budisa demonstrated that the integration of the orthogonal translation system into the host genome for a combined SCS/SPI experiment (Section 4.1.5) had beneficial effects for the number of incorporated ncAAs and the product titer.^526^ For combined SCS/SPI experiments, often two plasmids are used to encode the orthogonal translation system and the gene of interest, which further increases the metabolic burden and triggers plasmid loss,^517^ a very unfavorable situation for scale-up. To reduce the cellular load by the components that are encoded on the plasmid and to improve their segregational stability, it would make sense to integrate the orthogonal translation system into the genome and to keep only the gene of interest on the plasmid. Ultimately, microbial chassis as described by Karbalaei-Heidari and Budisa could facilitate the parallel incorporation of different ncAAs, one by SPI and the other by SCS under scalable production conditions.

An inherent problem of SPI is the unselective incorporation of the ncAA into any newly synthesized protein as soon as the compound accumulates to a level inside the cell that allows its efficient participation in ribosomal translation (Figure 22). This implication has several consequences: First of all, not only the protein of interest (POI) but also the entire proteome are labeled. Substantial proteomic incorporation of the ncAA can lead to major changes in the host cell, which are reflected by restricted growth.^309^ Cells whose growth is impaired by the ncAA are unsuitable as production hosts. To the best of our knowledge, the relative distribution of different ncAAs in the POI vs the proteome has not yet been systematically assessed. How much of the ncAA “disappears” in the proteome and does the extent vary with the target protein? The results obtained with BONCAT and its derivatives imply that proteomic labeling can be quite efficient with Aha and Hpg (see the corresponding Section 4.3.1). Can the expression levels, the expression host, or any other process parameters shift the relative abundance? Can the decoupling of growth and protein production abolish the proteomic incorporation of the ncAA? Growth-decoupled manufacturing approaches separate the accumulation of cell mass from the accumulation of protein product.^527,528^ After the switch from growth to production, the host discontinues the production of its own proteins and is forced to channel the available cellular resources into the production of the recombinant protein. Expectedly, this situation would promote the incorporation of the ncAA into the target protein while the stochastic incorporation into the host proteome would be suppressed protecting it from the toxicity of the accidentally incorporated ncAA. However, if genes for the orthogonal T7 RNA polymerase or ncAA biosynthesis pathway are coexpressed with the target gene, the ncAA can be incorporated accidentally resulting in nonfunctional proteins (see Section 5.3).^58,94^ How can we design a (growth-decoupled) bioprocess that suppresses the accidental incorporation? Are cell-free SPI approaches as demonstrated by Worst et al.^146,529^ the future? These questions call urgently for a systematic analysis of the extent of the unintended or accidental incorporation of ncAAs. On the other hand, incomplete labeling of the target protein with the ncAA leads to inhomogeneous protein mixtures. Partially labeled variant species represent so-called product-related impurities, which are extremely difficult to separate from the desired product variant. Low titers of the desired product variant challenge the downstream processing train because unit operation efficiency decreases, irrespective of the separation principle, when the impurity content is high.^530,531^ Substantial product losses may be anticipated, emphasizing the need for strategies to address these challenges early on. Integrated process models would be very beneficial to tackle this issue. Clearly, upstream- and downstream processing should be integral to the design of the SPI bioprocess to control product quantity and quality, as well as sufficient product purity.

There is hardly any doubt about the exciting potential of ncAAs to considerably expand the scope of protein drugs with their extraordinary side chain chemistries and to advance the biomedical field to unprecedented opportunities.^532,533^ However, there are still some challenges in terms of their application and, particularly, their commercialization in the biopharmaceutical industry. The availability and the costs of ncAAs are still a hurdle, despite the generous profit margins for high value products in this sector.^450^ As outlined earlier, the key enzymes involved in the biosynthetic pathways of ncAAs are still largely unknown. Computational methods such as simulation prediction, retrobiosynthesis and computational design in combination with functional enzyme screening and enzyme engineering^534^ could advance the ncAA biosynthesis field.^453^ The aim are microbial cell factories that enable a sustainable and cost-effective procedure for the production of ncAAs. While economic and environmental considerations advocate for the biosynthesis of ncAAs over chemical methods,^450^ this preference excludes ncAAs that cannot be biosynthesized. In this case, partial enzymatic synthesis from chemical precursors can be a solution (e.g., Aha, Ahc, fluorinated Trp and Tyr analogs; Figure 21). Currently, the ncAAs are biosynthesized and incorporated into the target proteins by SPI in the same cell and in one process (see Section 5.2.2). In one reported case, this approach apparently led to the intoxication of the biosynthesis machinery by its own biosynthesized ncAA (Figure 22).^94^ In such cases, the separation of the biosynthesis of the ncAA and its incorporation could be a solution. Indeed, Trp analogs are well produced by a biocatalytic approach combining isolated or partially purified Trp synthase with Ser and the indole precursors in reaction buffer.^535–538^ The mixture containing the ncAA and innoxious precursors can be added directly to the growth medium.^124^ If this is not possible, for instance because a precursor is too toxic (Figure 22) at the concentration used for biocatalysis, a purification strategy for the ncAA must be devised.

### 5.5 Beyond the Horizon: Applications with Future Potential

NcAAs offer many opportunities for the development of new biomaterials reaching well beyond those described in Section 4.2. For instance, Deepankumar and co-workers proposed GFP­[Dopa] as a new photosensitizer for biosensitized solar cells based on promising initial photoconversion results with GFP­[Dopa] immobilized on TiO_2_ thin films.^68^ We expect more such unusual applications to appear in the future. Protein-based biomaterials often consist of repetitive sequence domains. SPI, particularly in combination with the overexpression of mutant AARSs (Section 4.1.2) represents a straightforward approach to label them globally with ncAAs carrying unorthodox side chains. Depending on their chemistry, the future might see “smart biomaterials” that are, e.g., stimuli-responsive, conductive, or colored. These traits are currently confined to nonprotein (organic) polymers.^539^

Xenobiology, a term originally coined to describe extraterrestrial life forms,^540^ has recently gained new momentum in the context of ncAAs. The modern connotation of xenobiology refers not only to extraterrestrial life forms but also to “human-made” unnatural life forms that are engineered for useful purposes.^541^ In principle, the unnatural life forms differ from extant ones by the use of non-natural building blocks for genetic information storage molecules (xenonucleic acid, XNA), metabolism (neometabolism) or proteins (alloproteins containing ncAAs). A thorough evaluation of the xenobiology field would go far beyond the scope of this review, and the interested reader is referred to a recent special edition on the topic in *ChemBioChem*.^542^ Nevertheless, we highlight here two selected developments that may be game changers for future applications of ncAAs in xenobiology.

The Romesberg group devised a “semisynthetic organism” (SSO), which uses an unnatural base pair (UBP) to encode an ncAA.^543^ The ncAA was incorporated in response to an orthogonal codon containing an unnatural base. The tRNA that was able to read this codon carried the complementary unnatural base in its anticodon (Figure 23). The approach resembles the attempts to incorporate ncAAs in response to translation stop codons or quadruplet codons using orthogonal AARSs and tRNAs. It extends the principle of orthogonality to the DNA and RNA levels and thus improves the selectivity and efficiency of the ncAA incorporation. To prevent the loss of the UBP, the host had to be engineered to eliminate target DNA without UBP^544^ or to avoid its editing.^545^ Several ncAAs were incorporated simultaneously into the same target protein in response to individual orthogonal codons. This SSO was the first organism capable of decoding 67 codons.^546^ The company Sanofi/Synthorx has commercialized the SSO platform for the discovery of novel therapeutic proteins.^547^

**Figure 23:**
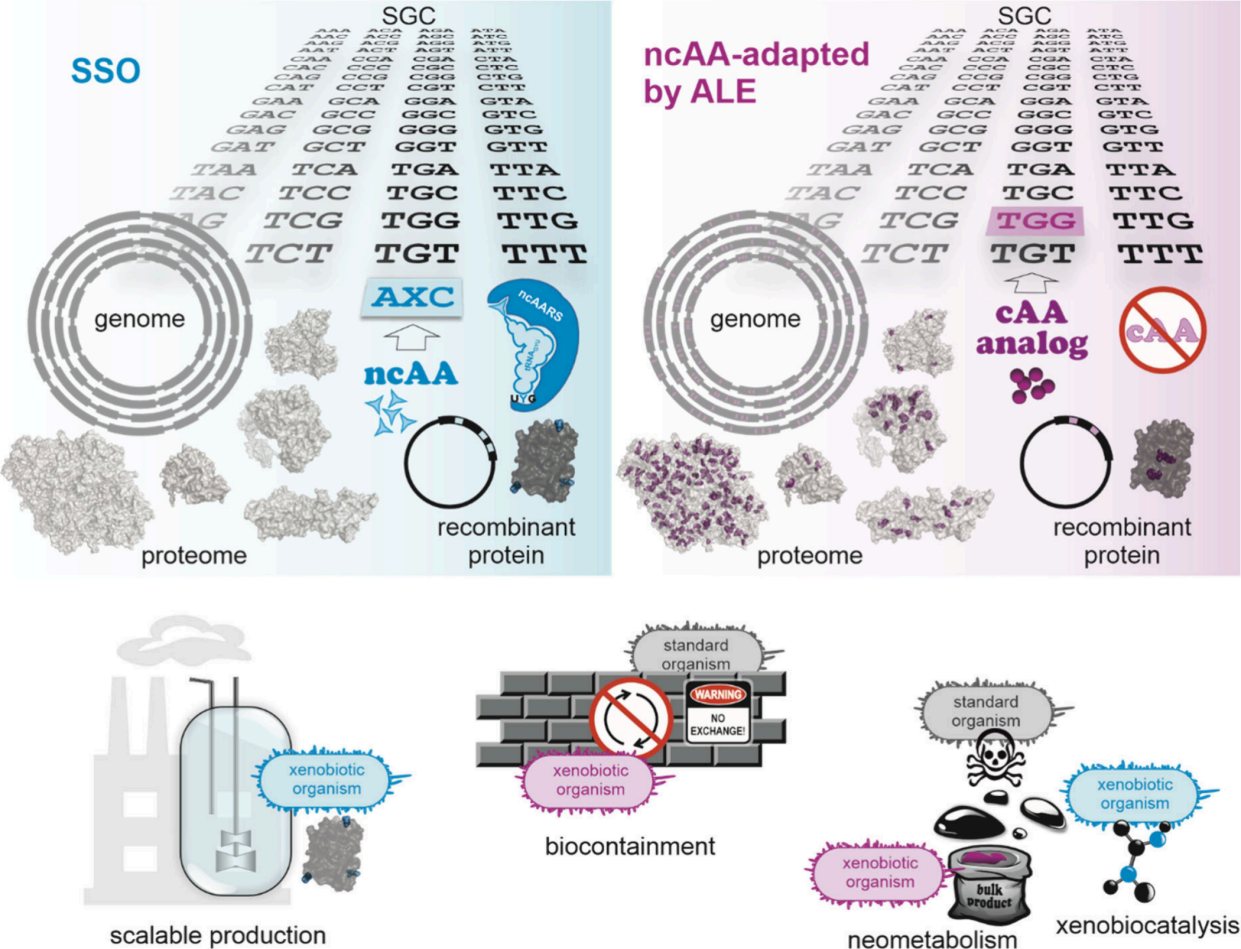
Xenobiotic organisms and their potential future applications. The semisynthetic organism (SSO, top left) was devised by the Romesberg group. It employs orthogonal codons containing unnatural base pairs, e.g., AXC, where X denotes the unnatural base, to encode ncAAs. An ncAA-specific aminoacyl-tRNA synthetase (ncAARS) charges the ncAA onto a tRNA carrying the complementary orthogonal anticodon, here tRNA_GYU_ where Y denotes the complementary unnatural base. The ncAA is incorporated into the recombinant target protein while the ncAA remains absent from the proteome of the SSO. The ncAA is an addition to the standard genetic code. Organisms that are adapted to ncAAs by ALE (top right) incorporate the analog in response to a sense codon in a recombinant target protein as well as the proteome. The organism is addicted to the ncAA, it replaces the corresponding cAA that vanishes from its genetic code. Xenobiotic organisms promise improved production of alloproteins (bottom left) or efficient biocontainment because of incompatible genetic information and/or metabolism (bottom center). Substrates that standard organisms cannot metabolize or which are even toxic for them (black droplets at bottom right) might be used to produce new-to-nature compounds by their neometabolism or when employed as xenobiocatalysts (bottom right).

From the perspective of xenobiology, organisms will become fully synthetic if they thrive on an expanded genetic code that stably accommodates at least one orthogonal codon *in addition* to the standard codons. That is, the orthogonal codon becomes a *new standard codon*, which is faithfully translated with an ncAA at any occurrence(s) in the genome or on recombinant DNA. The SSO platform developed by Romesberg and colleagues comes already quite close to this ideal. The ALE experiments to addict E. coli to an ncAA outlined in section 4.1.6 aim in a similar direction. Evidently, they did not employ an orthogonal codon. Instead, one of the standard codons, here the single Trp codon TGG, was reassigned to a Trp analog in a Trp auxotroph. In other words, the TGG codon of the SGC was interpreted in a nonstandard way. Conversely, this also means that Trp disappeared from the SGC and the entire proteome of the organism (Figure 23). It was replaced by a close structural and/or chemical analog such as [3,2]­Tpa or fluorotryptophan.^202,311,318^ The addition of an orthogonal codon to the SGC, by contrast, allowed the encoding of ncAAs with side chains whose structure and/or chemistry is very different form the cAAs.^543^ The thorough analysis of the organisms resulting from the ALE experiments is an invaluable source of information and inspiration to answer the question why and how all extant life forms on Earth evolved to use 20 cAA and triplets of only four nucleobases as the basic building blocks of life.^548^ The adapted strains encoded the Trp analogs extraordinarily efficiently at multiple occurrences in a target protein so that homogeneously labeled alloproteins resulted.^202,311,318^ However, all isolates were facultative users of the Trp analogs as well as Trp. Future applications will reveal whether they retain their adaptation under scaleup conditions, e.g., for industrial production. Similarly, the anticipated qualification of xenobiotic organisms for biocontainment,^549,550^ i.e., the synthetic organism cannot interfere with the natural ones, or “xenobiocatalysis”^551^ to biosynthesize new-to-nature compounds has yet to be shown (Figure 23).

## 6 Conclusions

The residue-specific incorporation of ncAAs in auxotrophic hosts has come a long way during the last two decades. The introduction of reactive handles into individual proteins has been and still is a central application of the method. It has advanced from the mere demonstration that it is possible to a manifold of implementations: Above all, ncAAs with bioorthogonal reactive side chain chemistries, specifically Aha, Anl and Hpg (Figure 7) have found tremendous use in the “NCAT” methods to study newly synthesized proteomes (Section 4.3.1). Highlights of the artificial post-translational modification of proteins containing reactive ncAAs (Section 3.1.1) include the engineering of biomaterials, specifically silk (Section 4.2); the directed immobilization of sensor proteins to improve the sensitivity of biosensors;^48^ the selective functionalization of proteins for their targeted delivery;^135,180^ the decoration of VLPs, e.g., for the potential use as a vaccine;^98^ or the stabilization of proteins by “stapling” *via* intramolecular cross-links of ncAAs with compatible bioorthogonal groups.^182^ The modulation of the antimicrobial properties of peptides by ncAAs, in particular the structural and chemical diversification of RiPPs represents another exciting strategy (Section 3.3.1). Their future production will require the careful design of production process where PTM helper proteins are reliably produced in their active form with cAAs and the ncAAs are efficiently introduced only into the peptides.

The precise manipulation of residues in the active site of enzymes typically calls for SCS unless the addressed catalytic cAA occurs only once. In the latter case, SPI can be an attractive alternative when the titer of the variant enzyme is equal to the parent’s or even exceeds it and the ncAA incorporation efficiency is high. SPI of ncAAs can alter the physicochemical properties of enzymes, such as substrate specificity, stability and activity (Section 3.3.5). The (poly)­fluorination of enzymes and of proteins in general allows us to influence their folding and stability (Section 3.3.2). Pro analogs play a central role in this application due to the special position of Pro among the cAAs (Section 3.3.3). Pro analog stereoisomers often provoke opposing effects in protein structure and/or folding. Even subtle atomic changes, such as the position of a fluorine atom, e.g., in the Phe ring can have a sever impact on the structure and function of a protein while on the other hand, the incorporation of many ncAAs may have hardly any effect. Currently, the consequences of cAA→ncAA exchanges are hard to predict, in particular when they occur at multiple sites in a protein. For this reason, the decision which ncAA to choose to provoke a desired effect is often an educated guess or even mere trial and error. The choice to tune the therapeutic properties of insulin with hydroxyproline represents an example for a very successful educated guess.^82^ Nevertheless, the field would benefit enormously from systematic efforts to predict the effects of ncAAs and to reveal potential structure/function relationships, possibly even in the context of varying protein structure environments.

SPI of ncAAs has been demonstrated with various amino acid auxotrophic organisms, such as bacteria, yeasts, mammalian cells and animals. However, E. coli is still the workhorse to produce recombinant alloproteins. Proteome-wide ncAA incorporation has focused on mammalian cell lines or animals, e.g., to study NSPs. On the other hand, ALE for the adaptation of entire proteomes to ncAAs and to addict organisms to them has been shown for bacteria so far (Section 4.1.6).

At the moment, we have a rather rudimentary notion how the ncAAs are taken up by the auxotrophic hosts^365^ and what intracellular fate(s) they experience other than being used in ribosomal translation. A comprehensive perception of these processes could guide the engineering of hosts and bioprocesses in a way that the AARSs charge the ncAAs onto their cognate tRNA(s) with an efficiency rivaling the cAAs. Hosts metabolically engineered to biosynthesize ncAAs (Section 5.2) most probably will play a major role in the future. Specifically, the scale-up of the method urgently demands the reduction of the costs for ncAAs as well as their unrestrained availability. Efforts to decipher the biosynthesis pathways of naturally occurring ncAAs, e.g., from streptomycetes^453^ or fungi,^1^ the retrosynthesis or *de novo* design of pathways represent exciting opportunities. In the context of the ncAA biosynthesis, smart strategies to avoid the inactivation of the biosynthesis enzymes by the accidental incorporation of ncAAs, such as the cross-expression technique (Section 5.3) need to be further developed.

The toolbox for the engineering of proteins with ncAAs is ever growing and many of the open questions outlined above do not only apply to the SPI of ncAAs in auxotrophic hosts but also to SCS, the recoding of sense codons and the incorporation of ncAAs using organisms with a compressed and expanded genetic code. A multidisciplinary effort combining concepts from biochemistry, molecular biology, synthetic biology, systems biology as well as computational biology and, last but not least, bioprocess engineering has an excellent chance to find answers and advance the field further.

For the burgeoning community of genetic code expansion afficionados, genetic code engineering may appear obsolete. With regard to its latest achievements and future prospects as outlined in this review, we are convinced that it is not. Neglecting its potentials and timely development, the ncAA incorporation field might miss an excellent opportunity to establish itself as an economically viable tool. The residue-specific incorporation of ncAAs in auxotrophic hosts is a simple, robust and straightforward experimental method. These desirable traits have granted it a long-lasting fixed star position in the “genetic code expansion universe”. It all started nearly 70 years ago with the seminal paper of Cohen and Cowie.^240^ Its way into the future has only just begun.

## Abbreviations

AARS: aminoacyl-tRNA synthetase
acetyl-CoA: acetyl-coenzyme A
ALE: adaptive laboratory evolution
*Bm*: Bombyx mori
BONCAT: bioorthogonal noncanonical amino acid tagging
cAA: canonical amino acid
CAT: chloramphenicol acetyltransferase
*Ce*: Caenorhabditis elegans
CoA: coenzyme A
CuAAC: copper­(I)-catalyzed [3 + 2] azide–alkyne cycloaddition
*D*_600_: attenuance at 600 nm
DADPS: dialkoxydiphenylsilane
DBCO: dibenzocyclooctyne
*Dm*: Drosophila melanogaster
DTT: dithiothreitol
*Ec*: Escherichia coli
EPL: expressed protein ligation
ESI-MS: electrospray ionization mass spectrometry
FACS: fluorescence-activated cell sorting
FBS: fetal bovine serum
FMDV: foot-and-mouth disease virus
FUNCAT: fluorescent noncanonical amino acid tagging
GFP: green fluorescent protein
glyco-MTS: glycomethanethiosulfonate
GSH: reduced glutathione
GST: glutathione S-transferase
IEDDA: inverse electron demand Diels–Alder
IleRS: isoleucyl-tRNA synthetase
IPTG: isopropyl β-d-1-thiogalactopyranoside
LC-MS/MS: liquid chromatography–tandem mass spectrometry
LeuRS: leucyl-tRNA synthetase
*Ll*: Lactococcus lactis
MAD: multiwavelength anomalous diffraction
*Mb*: Methanosarcina barkeri
MetAP: methionine aminopeptidase
MetRS: methionyl-tRNA synthetase
*Mj*: Methanocaldococcus jannashii
*Mm*: Methanosarcina mazei
MRI: magnetic resonance imaging
MRS: magnetic resonance spectroscopy
*Mu*: Mus musculus
ncAA: noncanonical amino acid
NMM: New Minimal Medium
NSP: newly synthesized protein
OCM: olefin cross-metathesis
OxC: oxime coupling
PBS: phosphate-buffered saline
PEG: poly­(ethylene glycol)
PheRS: phenylalanyl-tRNA synthetase
PLP: pyridoxal 5′-phosphate
POI: protein of interest
ProRS: prolyl-tRNA synthetase
PTM: post-translational modification
PylRS: pyrrolysyl-tRNA synthetase
RiPP: post-translationally modified peptide
RLP: resilin-like protein
*Sc*: Saccharomyces cerevisiae
scFv: single chain variable fragment
SCR: sense codon reassignment
SCS: stop codon suppression
SDS-PAGE: sodium dodecyl sulfate polyacrylamide gel electrophoresis
SGC: standard genetic code
SpAAC: strain-promoted [3 + 2] azide–alkyne cycloaddition
SPI: supplementation incorporation
SSO: semisynthetic organism
T7RNAP: T7 RNA polymerase
TEC: thiol–ene click chemistry
*T*_m_: melting temperature
*T*_opt_: optimal temperature
TrpRS: tryptophanyl-tRNA synthetase
TyrRS: tyrosyl-tRNA synthetase
UBP: unnatural base pair
ValRS: valyl-tRNA synthetase
VLP: virus-like particle
